# Enhanced Photoredox Activity in Nitrogen‐Doped Carbon Nitride for Heterogeneous Nitrogen and Oxygen Radical Reactions

**DOI:** 10.1002/advs.202417752

**Published:** 2025-02-22

**Authors:** Lan Qin, Huan Liu, Yi Wei, Lian‐Qing Chen, Xiao‐Qiang Hu

**Affiliations:** ^1^ Key Laboratory of Catalysis and Energy Materials Chemistry of Ministry of Education & Hubei Key Laboratory of Catalysis and Materials Science School of Chemistry and Materials Science South‐Central Minzu University Wuhan 430074 China

**Keywords:** nitrogen radicals, nitrogen‐doped carbon nitride, oxidative reactions, oxygen radicals, photoredox reactions

## Abstract

A nitrogen‐doped carbon nitride (NCN) has been synthesized and utilized for the sustainable generation of reactive nitrogen and oxygen radicals under mild photocatalytic conditions. This material exhibits a significant improvement in the separation and transfer of photoexcited charge carriers, which facilitates the direct activation of N─H and O─H bonds to achieve a variety of radical carboamination, oxyamination and deoxygenation reactions. The NCN catalyst has further demonstrated its robust photoredox activity through several successful applications, including the aerobic oxidation of boronic acids and the controllable oxidation of alcohols to aldehydes and carboxylic acids. Moreover, the NCN catalyst maintains its reactivity and efficiency even after multiple cycles of use, showcasing its durability and potential for practical applications in sustainable chemistry.

## Graphical abstract

A nitrogen‐doped carbon nitride (NCN) has been synthesized and applied for the sustainable generation of reactive nitrogen and oxygen radicals. The NCN catalyst facilitates radical carboamination, oxyamination, and deoxygenation reactions, as well as the oxidation of boronic acids and alcohols, all under mild photocatalytic conditions.

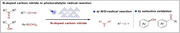

## 1 Introduction

Nitrogen‐containing heterocycles are ubiquitous in natural products, pharmaceuticals, agrochemicals and functional materials. Visible light photocatalytic nitrogen radical reaction has recently emerged as a powerful platform for the assembly of nitrogen heterocycles.^[^ ^1^, ^2^, ^3^ ^]^ Despite the extensive application of carbon radicals, the development of nitrogen radical chemistry has been relatively sluggish, primarily due to the scarcity of versatile and accessible methods for their generation. Known approaches to generate nitrogen radicals predominantly involve the reductive scission of N─X (X = Cl, Br), N─N, N─O and N─S bonds.^[^ ^4^, ^5^, ^6^, ^7^, ^8^, ^9^, ^10^, ^11^, ^12^ ^]^ However, the reaction generality of these strategies is often limited by several intrinsic challenges, such as the laborious preparation of N‐functionalized precursors, poor functional group tolerance and low atomic economy. In stark contrast, the straightforward conversion of N─H bonds into N‐radicals to forge new C─N bonds has been a tantalizing yet formidable project for synthetic chemists. In 2014, Xiao and Chen et al. unveiled a photocatalytic oxidative deprotonation electron transfer (ODET) strategy for the catalytic generation of N‐radicals from hydrazones.^[^ ^13^, ^14^, ^15^ ^]^ The group of Knowles pioneered a proton‐coupled electron transfer (PCET) strategy to transform amide N─H bonds into N‐radicals.^[^ ^16^, ^17^, ^18^ ^]^ A stark increase in the number of N‐radical reactions occurred in the year following the unveiling of ODET and PCET strategies.^[^ ^19^, ^20^, ^21^, ^22^, ^23^, ^24^, ^25^ ^]^ Despite the substantial advancements in the realm of N‐radical chemistry, it is crucial to acknowledge that most of these strategies necessitate non‐recyclable, precious ruthenium and iridium‐based polypyridyl complexes, thereby largely limiting their broader applicability due to production costs and practicality concerns. Consequently, there is an urgent need for the development of innovative photocatalytic systems that utilize recyclable, metal‐free photocatalysts to directly convert N/O─H bonds into nitrogen and oxygen radicals to efficiently construct heterocycles. The discovery of novel reactions within this framework is highly sought after, yet it remains an elusive target at the current stage.

Heterogeneous photocatalysis has been garnering significant interest from the synthetic chemistry community owing to the ease of catalyst separation and their superior recyclability.^[^ ^26^, ^27^ ^]^ In this context, polymeric carbon nitrides (CN) stand out as non‐toxic, structurally tunable that can be readily synthesized from commodity chemicals.^[^ ^28^, ^29^ ^]^ Unlike widely used semiconductor TiO_2_, CN polymers exhibit a narrow band gap (2.7 eV vs 3.2 eV) and strong light absorption in the visible region, making them particularly promising catalysts in water splitting, CO_2_ reduction, environmental remediation and so on.^[^ ^30^, ^31^, ^32^, ^33^, ^34^ ^]^ Despite its immense potential, the application of CN materials in synthetic photocatalysis is largely unexploited due to the rapid recombination of photogenerated holes and electrons that inhibit the expected electron transfer (ET) to molecules. The combination or integration of metal catalysts such as Ni, Cu, Fe, Pd and Ru *etc*. with carbon nitrides has been demonstrated to be particularly beneficial in the context of narrowing band gap and enhancing photocatalytic performance.^[^ ^35^ ^]^ These catalyst systems are capable of enabling a spectrum of traditionally challenging cross‐coupling, hydrogenation, and oxidation reactions that were not feasible with single CN catalyst.^[^ ^36^, ^37^, ^38^, ^39^, ^40^, ^41^, ^42^ ^]^ While impressive progress on CN and metal‐doped CN mediated heterogeneous photocatalysis, a general CN‐catalyzed N‐ and O‐radical reaction via the direct cleavage of N/O─H bonds remains a long‐standing but unsolved project in this field. To date, only two examples have been reported independently by Nocera^[^ ^43^ ^]^ and Wang.^[^ ^44^ ^]^ In these pioneering studies, modified CN photocatalysts with long‐lived triplet excited states or rich in −NH_2_ groups were designed to facilitate N‐radical hydroamination reactions.

The surface characteristics of heterogeneous catalysts play a pivotal role in their catalytic efficiency by affecting the mobility of charge carriers, and the interaction between catalyst and substrate.^[^ ^45^ ^]^ By employing strategies such as doping, nanostructure engineering, and copolymerization to modify the CN catalyst's surface, a robust platform has been established for uncovering innovative catalysts and novel reactions.^[^ ^35^ ^]^ Inspired by these collective studies,^[^ ^35^, ^36^, ^37^, ^38^, ^39^, ^40^, ^41^, ^42^, ^43^, ^44^ ^]^ we hypothesized that the nitrogen‐doped carbon nitride (NCN) with a narrow band gap of 2.20 eV and outstanding photocatalytic performance, could be a promising solution to the enduring challenges in heterogeneous N‐ and O‐radical reactions.^[^ ^46^ ^]^ In this work, we have achieved the photocatalytic conversion of N─H and O─H bonds into nitrogen and oxygen radicals using a readily accessible NCN catalyst for the first time, enabling a range of N/O‐radical carboamination, oxyamination and deoxygenation reactions. Notably, the NCN catalyst showcases remarkable recyclability and maintains its photoactivity across multiple cycles. It also excels in the aerobic oxidation of boronic acids, and controllable oxidation of alcohols, demonstrating its good potential in organic synthesis. The reported catalytic system is highly efficient and sustainable, which marks a significant advance to the limited alternative Ru‐ and Ir‐based procedures requiring the use of activated precursors or stoichiometric oxidants.^[^ ^4^, ^5^, ^6^, ^7^, ^8^, ^9^, ^10^, ^11^, ^12^ ^]^

## 2 Results and Discussions

**Figure 1:**
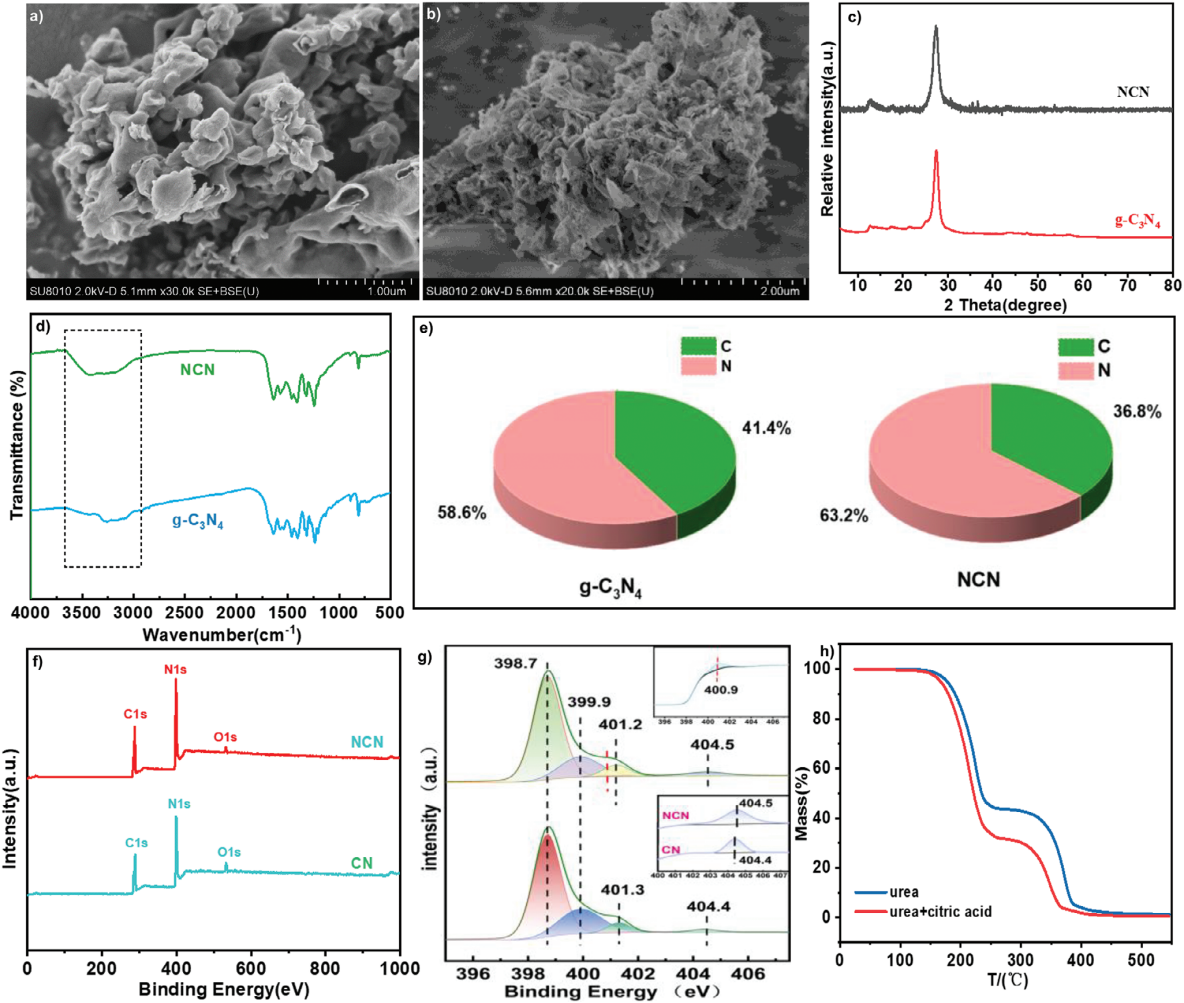
a) Scanning Electron Microscope of g‐C_3_N_4_. **b)** Scanning Electron Microscope of NCN. **c)** X‐Ray Diffraction of g‐C_3_N_4_ and NCN. **d)** Fourier‐Transform Infrared Spectra of g‐C_3_N_4_ and NCN. **e)** Elemental Analysis of g‐C_3_N_4_ and NCN. **f)** X‐ray Photoelectron Spectroscopy of g‐C_3_N_4_ and NCN. **g)** High‐resolution N 1s spectrum of g‐C_3_N_4_ and NCN. **h)** Thermogravimetric of g‐C_3_N_4_ and NCN.

The NCN photocatalyst is synthesized by mixing urea (25 g) with citric acid (25 mg), subsequently calcining the mixture in a crucible with a cover at 550 °C for 4 h with a ramp rate of 2 °C min^−1^ in the air (see SI for more details).^[^ ^47^ ^]^ In g‐C_3_N_4_, two prominent diffraction peaks at 13.0° and 27.3° correspond to the (100) and (002) diffraction planes of the typical g‐C_3_N_4_ phase based on the *tri*‐s‐triazine structural units (i.e., heptazine‐based g‐C_3_N_4_).^[^ ^48^ ^]^ These peaks are attributed to the in‐plane stacking of basal sequences and interlayer stacking of aromatic segments. The XRD characteristic diffraction peaks of NCN and g‐C_3_N_4_ are closely matched, indicating that N‐doping does not disrupt the crystal phase (**Figure** **1**c). The characteristic peaks of NCN and g‐C_3_N_4_ are similar in Fourier transform infrared (FT‐IR) spectroscopy, confirming that g‐C_3_N_4_ framework remains intact upon the introduction of N‐atom (Figure 1d). Elemental analysis (EA) results showed that both catalysts contain only N and C two elements. The nitrogen content in NCN (63.2%) surpasses that in g‐C_3_N_4_ (58.6%), indicating a significant presence of N atom doping during the synthesis of NCN (Figure 1e). The elevated nitrogen content in NCN can be attributed to the release of NH_3_ during the decomposition of urea. To further elucidate the chemical states and elemental composition of NCN, X‐ray photoelectron spectroscopy (XPS) analysis was conducted (Figure 1f). The result showed that the N‐content in NCN is higher than g‐C_3_N_4_, consistent with elemental analysis. Peaks attributed to residual oxygen in NCN and g‐C₃N₄ are observed, as a small portion of the catalyst's edge is inevitably oxidized during its preparation. The high‐resolution C 1s spectra analysis showed that NCN and g‐C_3_N_4_ have two distinct peaks with similar spectra. Peaks at 284.8 and 288.2 eV are attributed to sp^2^‐hybridized carbon in C═C/C═C and the aromatic ring (N─C═N), respectively. The peak at 288.2 eV in NCN is stronger than g‐C_3_N_4_ (Figure S1 in (Supporting Information), 65.14% vs 62.35%).

**Scheme 1:**
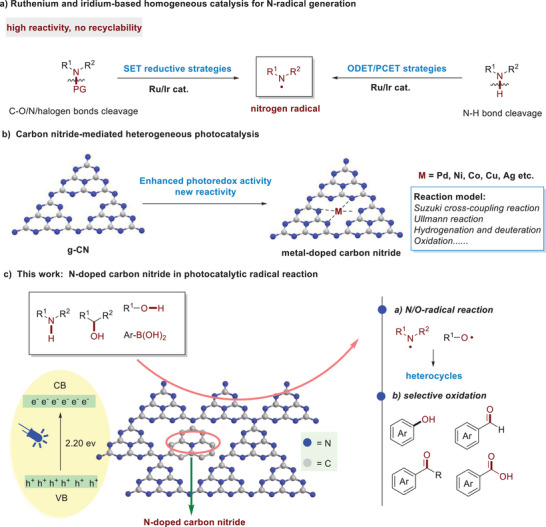
Homogeneous photocatalytic N‐radical reaction, carbon nitrides in heterogeneous catalysis and our reaction design.

The corresponding N 1s spectrum displays four peaks (Figure 1g). The peak at 398.7 eV is attributed to double‐coordinated sp^2^‐hybridized (N_2c_) nitrogen atoms in g‐C_3_N_4_. Peaks at 400.0 and 401.3 eV are assigned to tri‐coordinated (N_3c_) nitrogen atoms and surface amino groups (C─N─H), respectively. The peaks at 404.4 and 404.5 are attributed to the charge effect, while a new peak at 400.9 is observed in the spectrum of NCN, indicating the formation of a nitrogen‐doped graphitic carbon structure as shown in **Scheme** **1**c. This result is consistent with Shi's work.^[^ ^47^, ^49^ ^]^ TG curves disclosed that the addition of citric acid leaded to the more weight loss above the melting point (≈132.7 °C) of urea, demonstrating that reaction/condensation has taken place between citric acid and urea (Figure 1h). Given the reactivity of citric acid's carboxyl groups with urea's amine groups, it is anticipated that during the calcination process, a copolymerization reaction will occur between citric acid and urea. This reaction is expected to form N‐doped graphitic carbon. The resulting material could either be anchored onto the surface or integrated within the *tris*‐triazine framework of g‐C_3_N_4_, thereby enhancing its structural and functional properties.

**Figure 2:**
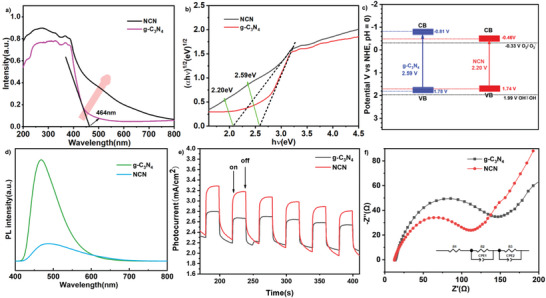
a) UV–Vis diffuse reflectance spectra of g‐C_3_N_4_ and NCN. **b)** Band gap maps of g‐C_3_N_4_ and NCN. **c)** Summarizes the band structures of g‐C_3_N_4_ and NCN. **d)** Photoluminescence spectra of g‐C_3_N_4_ and NCN. **e)** Transient photocurrent response maps g‐C_3_N_4_ and NCN. **f)** Nyquist plots are displayed for pure g‐C_3_N_4_ and NCN.

As shown in the UV–vis absorption features (**Figure** **2**a), g‐C_3_N_4_ exhibits a distinct absorption band at 464 nm. In comparison to g‐C_3_N_4_, NCN displays a noticeable redshift in its absorption edge, exhibiting strong absorption in the visible light range due to the multiple scattering effects resulting from its porous structure. To further investigate the optical properties of NCN, we calculated the band gaps of each sample using the formula (αhυ)^1/2^ = A (hυ‐Eg) (1), as shown in Figure 2b. The band gap of g‐C_3_N_4_ is 2.59 eV. After N doping, the band gap of NCN decreases to 2.20 eV. For the NCN sample, nitrogen doping on the g‐C_3_N_4_ matrix may induce the release of π electrons in this conjugated system due to the presence of lone pair electrons on nitrogen atoms, potentially contributing to another enhancement in light absorption. As shown in Figure S2 in (Supporting Information), the Mott‐Schottky (MS) plots of g‐C_3_N_4_ and NCN samples all exhibit positive slopes, indicating *n*‐type semiconductor characteristics. The flat band potentials of g‐C_3_N_4_ and NCN are −1.26 and −0.91 V (vs SCE, pH = 7), converted to −0.61, and −0.26 V (vs RHE). Generally, the conduction band potential (CB) of n‐type semiconductors is 0–0.2 V lower than their flat band potentials. Therefore, by calculation, the CB values for g‐C_3_N_4_ and NCN are −0.81 and −0.46 V, respectively. Combining with the band gaps determined by UV–Visible spectroscopy and using the formula (EVB = ECB + Eg), the valence band (VB) potentials of g‐C_3_N_4_ and NCN are calculated as 1.78, and 1.74 V, respectively (Figure 2c). According to the results of photoluminescence (PL) spectrum in Figure 2d, the pristine g‐C_3_N_4_ exhibits a high emission peak at a wavelength of 470 nm, primarily attributed to the severe recombination of photogenerated electrons and holes in g‐C_3_N_4_. Upon mixing and calcination with citric acid, the intensity of the emission peak significantly decreases, indicating that N‐doping can effectively quench the fluorescence, suggesting a higher efficiency in the separation of photogenerated charge carriers.

To further investigate the migration of charge carriers and the interface transfer/recombination rate, transient photocurrent responses and electrochemical impedance spectroscopy (EIS) were measured. As depicted in Figure 2e, the photocurrent intensity sharply increases and decreases when the light is turned on and off, indicating the generation of light‐induced electrons under illumination. For g‐C_3_N_4_, the photocurrent intensity is lower due to the substantial recombination of photogenerated electrons and holes. The photocurrent intensity exhibited by NCN significantly outperforms that of g‐C_3_N_4_, underscoring its exceptional capability in the efficient separation of photogenerated electron‐hole pairs. Furthermore, Nyquist plots are displayed for g‐C_3_N_4_ and NCN (Figure 2f). Generally, the smaller arc radius in the electrochemical impedance spectroscopy (EIS) plot suggests the higher efficiency of charge transfer. The arc radius of NCN is the smallest, indicating that NCN can achieve more effective separation of photogenerated charge carriers, consistent with the results of transient photocurrent responses. Taken together, The NCN photocatalyst demonstrates superior separation efficiency for photogenerated electrons and holes, coupled with a more rapid charge transfer rate compared to g‐C_3_N_4_. These attributes significantly extend the lifespan of photogenerated charge carriers and bolster its photoredox capabilities, making it particularly effective for radical reactions.

To test the photocatalytic activity of the obtained NCN in radical reaction, we initiated our investigation by exploring the N‐radical cascade reaction between β, γ‐unsaturated hydrazone **1** and nitrosobenzene **2** in the presence of NCN photocatalyst under irradiation by 405 nm LEDs. To our delight, an unprecedented 4,5‐dihydropyrazole‐based nitrone product **3** was obtained with excellent selectivity (**Table** **1**). Typically, the addition of an alkyl radical to nitrosoarene directly gives rise to the hydroxylamine product.^[^ ^50^ ^]^ The intriguing nitrone products that obtained in our reaction system not only reveal an alternative reaction mechanism but also highlight the NCN catalyst's potential in discovering novel chemical transformations. It is important to note that 4,5‐dihydropyrazoles serve as crucial structural components in a variety of bioactive molecules,^[^ ^51^ ^]^ and nitrones act as highly versatile synthons in 1,3‐dipolar cycloaddition reactions.^[^ ^52^ ^]^ The integration of dihydropyrazole and nitrone structures into a unified molecular framework offers a promising platform for the synthesis and exploration of compounds with significant biologically applications. After a systematic exploration of reaction parameters, the optimal conditions have been demonstrated by using NCN (5.0 mg), K_2_CO_3_ (1.5 equiv) as base in CH_3_CN under the irradiation by 405 nm LEDs for 12 hours at room temperature, delivering nitrone product **3** in 83% isolated yield (entry 1). The simple g‐C_3_N_4_ resulted in a lower yield (entry 2, 22%). The reaction can also proceed in other solvents (THF or DCM), or upon the irradiation by 450 or 390 nm LEDs, providing product **3** in moderate yields (entries 3–6). To verify the necessity for each component involved in this reaction, a series of control experiments were conducted. These results clearly revealed that NCN photocatalyst, visible light irradiation, and K_2_CO_3_ were all critical to the reaction (entries 7–9). In addition, the larger surface area of the catalyst may contribute to enhanced catalytic efficiency (see SI for more details).

**Table 1:** Optimization of reaction conditions^a)^.

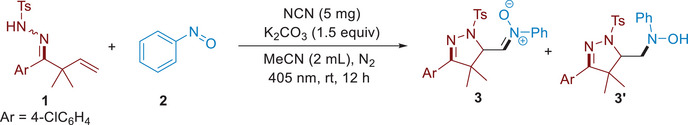						
Entry	Catalyst	Base	Light	Solvent	Yield of 3 (%)^b)^	Yield of 3′ (%)^b)^
1	NCN	K_2_CO_3_	405 nm	CH_3_CN	83	trace
2	g‐C_3_N_4_	K_2_CO_3_	405 nm	CH_3_CN	22	trace
3	NCN	K_2_CO_3_	405 nm	THF	60	trace
4	NCN	K_2_CO_3_	405 nm	DCM	61	trace
5	NCN	K_2_CO_3_	456 nm	CH_3_CN	48	trace
6	NCN	K_2_CO_3_	390 nm	CH_3_CN	65	trace
7	–	K_2_CO_3_	405 nm	CH_3_CN	0	0
8	NCN	–	405 nm	CH_3_CN	0	0
9	NCN	K_2_CO_3_	–	CH_3_CN	0	0

^a)^ Reaction conditions: **1** (0.1 mmol), **2** (0.3 mmol), photocatalyst (5 mg), K_2_CO_3_ (1.5 equiv), solvent (2 mL) under N_2_ at room temperature for 12 h upon the irradiation by 5 W 405 nm LEDs.

^b)^ Isolated yields based on **1**.

**Scheme 2:**
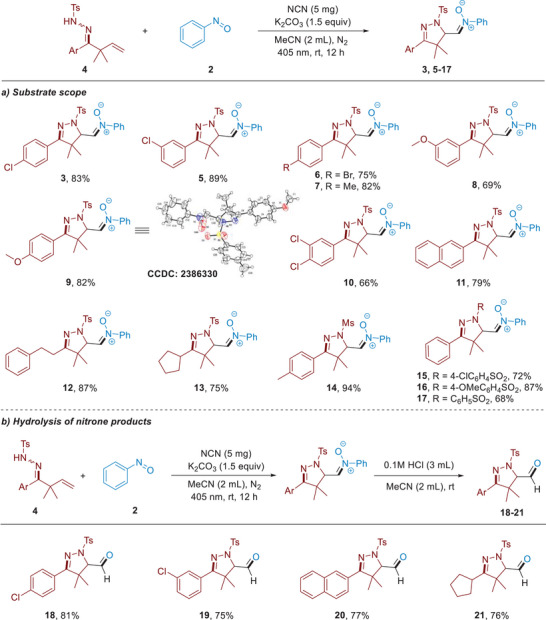
Reaction scope of carboamination (Reactions were run with **4** (0.1 mmol), **2** (0.3 mmol), K_2_CO_3_ (0.15 mmol), NCN (5 mg), in MeCN (2 mL) under N_2_ at room temperature for 12 h under the irradiation by 405 nm LEDs.) and hydrolysis of nitrone products (0.1 M HCl (3 mL), MeCN (2 mL) at room temperature for 12 h).

With the optimized conditions in hand, we then evaluated the generality of this N‐radical cascade reaction with respect to diversely substituted β, γ‐unsaturated hydrazones. As shown in **Scheme** **2**a, the electronic properties and substitution patterns of aromatic rings in β, γ‐unsaturated hydrazones were systematically examined. Accordingly, the hydrazones bearing electron‐withdrawing (Cl, Br, 3,4‐2Cl) or electron‐donating substituents (Me, MeO) at the *para*‐ or *meta*‐positions of phenyl rings performed well, furnishing the expected products **5–10** in 66–89% yield. 2‐Naphthyl substituted β, γ‐unsaturated hydrazone was well‐tolerated. Alkyl groups such as phenylethyl and cyclopentyl substituted hydrazones proceeded smoothly in this reaction to give **12** and **13** in 87% and 75%, respectively. Moreover, the replacement of tosyl group by methanesulfonyl (Ms), *p*‐chlorosulfonyl, *p*‐methoxysulfonyl, and sulfonyl groups had no significant influence on the reaction efficiency, providing the corresponding product **14–17** in generally good yields (68‐94%). To underscore the practical utility of this approach in the synthesis of valuable molecular structures, we have attempted to convert the nitrones into aldehydes under mild conditions. As outlined in Scheme 2b, under acidic conditions, a range of nitrones can be easily converted into aldehydes with good yields (**18‐21**, 75% to 81%).

**Scheme 3:**
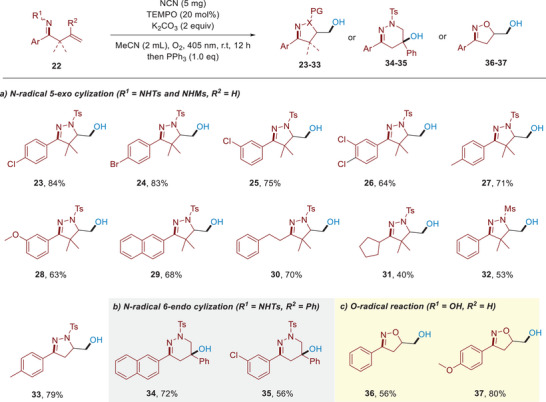
Reaction scope of oxyamination and deoxygenation of alkenes (Reactions were run with **22** (0.2 mmol), K_2_CO_3_ (0.4 mmol), TEMPO (0.04 mmol, 20 mol%), NCN (5 mg), in MeCN (2 mL) under a balloon of O_2_ under the irradiation by 405 nm LEDs.

To further explore the photoredox activity of NCN catalyst, we next sought to extend this new NCN‐mediated radical reaction to oxyamination and deoxygenation of alkenes.^[^ ^53^, ^54^ ^]^ Herein, we designed a novel, dual NCN/TEMPO catalytic system that enabled the efficient generation of N/O‐radicals for oxyamination and deoxygenation reactions using molecular oxygen (O_2_) as a green terminal oxidant and oxygen source. As shown in **Scheme** **3**a, the N‐radical 5‐*exo*‐oxyamination reactions proceeded smoothly to afford the expected products in generally high yields (**23‐33**, 40–84%). Moreover, we are delighted to find that a highly selective N‐radical 6‐*endo*‐oxyamination can be achieved by introducing a phenyl group at the end of alkenes to access biologically important pyridazine derivatives in high efficiency (Scheme 3b, **34%** and **35%**, 72**%** and 56%), showing synthetic potential of this protocol. Meanwhile, we set out to apply this unified photocatalytic platform for the direct activation of O–H of oximes to generate oxygen radicals, thus achieving radical deoxygenation of β, γ‐unsaturated oximes. To our delight, the deoxygenation reaction proceeded smoothly to give isoxazoline products in satisfying yields (Scheme 3c, **36** and **37**).

**Scheme 4:**
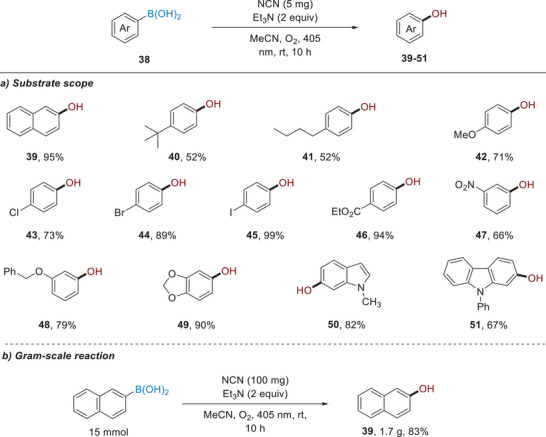
Photocatalytic oxidation of boronic acids (Reactions were run with **38** (0.2 mmol), NCN (5 mg), TEA (2 equiv), CH_3_CN (2 mL) at room temperature for 10 h under a balloon of O_2_ upon the irradiation by 405 nm LEDs.

The photocatalytic activation of molecular oxygen to its highly reactive species such as singlet oxygen (^1^O_2_) and superoxide radical anion provides a powerful and sustainable platform for photooxidative reactions.^[^ ^55^ ^]^ Phenols are particularly important building blocks and versatile intermediates with wide application in chemical and pharmaceutical industries. In 2012, the group of Xiao first reported a [Ru(bpy)_3_Cl_2_]·6H_2_O catalyzed aerobic oxidative hydroxylation of arylboronic acids to phenols under mild photocatalytic conditions.^[^ ^56^ ^]^ In order to avoid the use of expensive Ru‐complex, we intended to utilize heterogeneous NCN catalysts to achieve the oxidation of arylboronic acids. Under slightly modified conditions, we were pleased to find that a wide range of structurally unbiased arylboronic acids can be smoothly transformed into phenols with good to excellent yields (**Scheme** **4**a). For example, the ester group, halogen atoms (Cl, Br, I) and indole‐ and carbazole‐based boronic acids are compatible with this oxidative hydroxylation process. It is worth mentioning that the reaction can be readily scaled up to 15 mmol without significant decrease in the product yield (Scheme 4b).

**Scheme 5:**
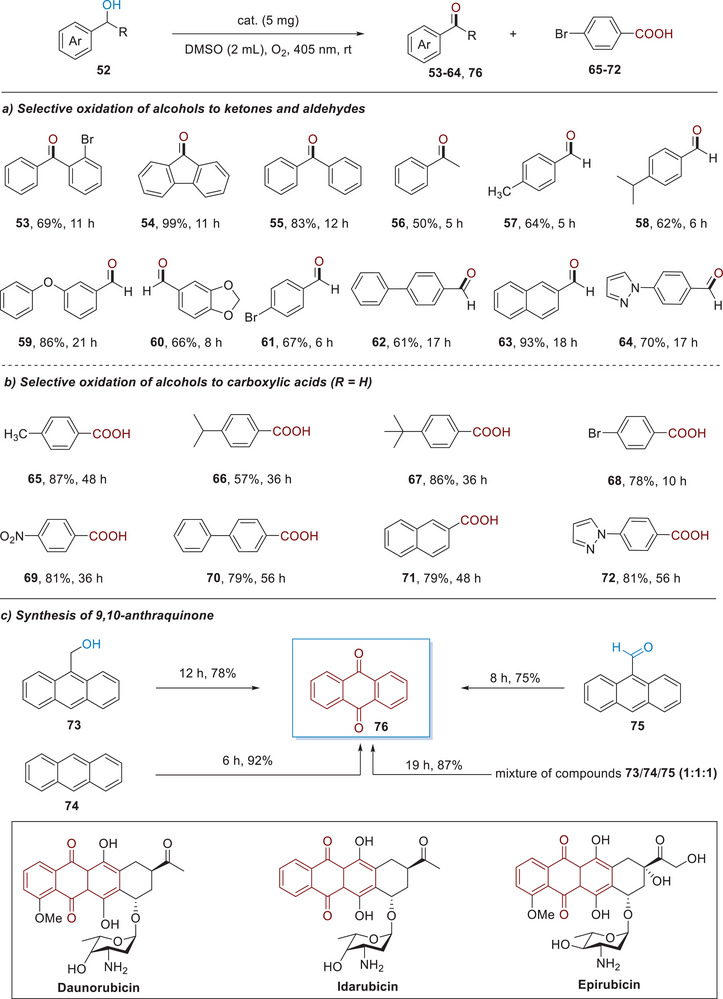
Selective oxidation of alcohols and synthesis of 9,10‐anthraquinone (Reactions were run with **1a** (0.2 mmol), NCN (5 mg) in DMSO (2 mL) at room temperature under a balloon of O_2_ upon the irradiation by 405 nm LEDs.

To further demonstrate the utility of this photocatalytic activation protocol, we turned our attention to the aerobic oxidation of alcohols, which is a fundamental transformation in organic chemistry and industrial science.^[^ ^57^ ^]^ Accordingly, we developed a general catalytic method for alcohol oxidation via the combination of NCN catalyst, a 405 nm LED light source and O_2_ as a green oxidant in DMSO. As shown in **Scheme** **5**a, we were gratified to find that a broad array of electron‐rich and ‐deficient alcohols were readily oxidized to ketones and aldehydes with good yields and excellent chemoselectivity (53% to 64, 50**%** to 93%). Direct oxidation of alcohols to carboxylic acids has posed a general synthetic challenge, with existing methods displaying low chemoselectivity, poor functional group tolerance and limited scope due to the harsh reaction conditions and the requirement of strong oxidants such as KMnO_4_, K_2_Cr_2_O_7_ *etc*. in acidic conditions. Upon investigating the reaction scope, we were delighted to find that benzoic acids could be exclusively obtained by prolonging the reaction time (Scheme 5b), highlighting the versatility and robustness of this photooxidative protocol. To the best of our knowledge, this represents an example of the use of heterogeneous photocatalysis to achieve controlled oxidation of alcohols, thereby selectively producing aldehydes and benzoic acids. Furthermore, under this mild oxidative conditions, 9‐anthracenemethanol (**73**), anthracene (**74**), 9‐anthraldehyde (**75**) can be efficiently transformed into 9, 10‐anthraquinone (75**%** to 92%), which are ubiquitous in organic dyes and Food and Drug Administration (FDA) approved drugs such as daunorubicin, idarubicin and epirubicin *etc*.^[^ ^58^ ^]^ The reaction of the mixture of **73**, **74** and **75** also proceeded smoothly to give 9, 10‐anthraquinone in as the sole product in 87% yield.

**Scheme 6:**
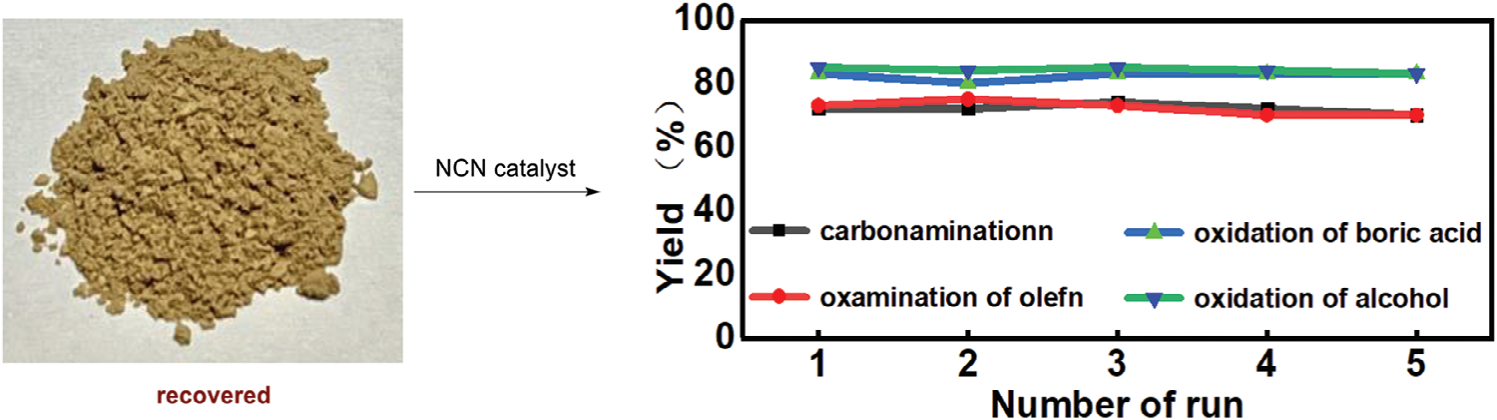
The recyclability of NCN catalyst.

To our delight, the NCN catalyst can be reused at least five times (**Scheme** **6**) without the loss of activity. These results clearly demonstrate the robustness and high efficiency of the NCN photocatalyst in the developed N‐ and O‐radical reactions.

**Scheme 7:**
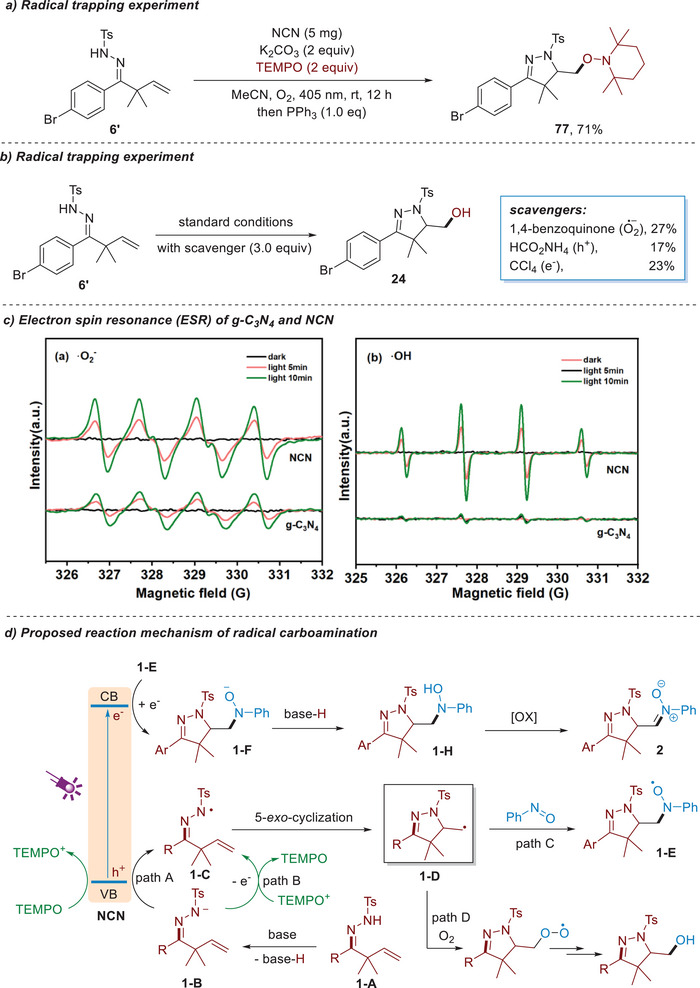
Mechanistic studies and proposed mechanism.

To gain insight into the reaction mechanism, a C‐radical trapping experiment has been first carried out using TEMPO as a radical scavenger, and the corresponding TEMPO adduct **77** was obtained in 71% yield (**Scheme** **7**a), which indicated the intermediacy of a carbon‐centered radical through 5‐*exo*‐cyclization of N‐radical. Moreover, the addition of superoxide radical (·O_2_^−^) scavenger 1,4‐benzoquinone, hole (h^+^) scavenger (HCOONH_4_) or electron (e^−^) scavenger (CCl_4_) significantly suppressed the reactions, suggesting the involvement of these reactive species in our reaction (Scheme 7b).^[^ ^59^ ^]^ Using 5,5‐dimethyl‐1‐pyrroline N‐oxide (DMPO) as a radical trapping agent, the generation of superoxide radical (·O_2_^−^) and hydroxyl radical (·OH) were detected by electron spin resonance (ESR) through the photoactivation of O_2_ by NCN and g‐C_3_N_4_. As shown in Scheme 7c, NCN records stronger DMPO‐·OH and DMPO‐·O_2_^−^ signals than g‐C_3_N_4_, indicating that NCN possesses strong redox capability to activate O_2_. These results demonstrated the electron‐hole separation efficiency of NCN is higher than g‐C_3_N_4_, allowing produce ·OH and ·O_2_^−^ species in a higher efficiency for the oxidation reactions. Moreover, NCN catalyst (E^ox^_1/2_ = +1.74 V) is likely to be sufficiently oxidizing the nitrogen anion (E_p_^red^ = 0.56 V vs SCE) and TEMPO (E_1/2_ = +0.62 V vs Ag/AgCl) to generate N‐radical and TEMPO^+^ species, respectively. On basis of these results, a possible mechanism of radical carboamination is proposed in Scheme 7d. First, upon the irradiation by 405 nm LEDs, NCN is excited to produce electron‐hole pairs, facilitating their separation and thereby triggering redox reactions. Under basic conditions, the deprotonation of hydrazone generates nitrogen anion **1‐ B**, which transfers an electron to the hole to form N‐radical **1‐C** (Path A). Then, a 5‐*exo*‐cylizatiuon of **1‐C** provides C‐based radical **1‐D** that can be readily trapped by nitrosobenzene to deliver O‐centered radical **1‐E** (Path C). A second electron transfer from CB to **1‐E** to form oxygen anion and recover the NCN catalyst for next catalytic cycle. The protonation of **1‐E** produces hydroxylamine **1‐H** that can be further oxidized to nitrone product. For TEMPO‐mediated oxyamination reaction, TEMPO^+^ is first generated through the oxidation of TEMPO by hole, which then oxidizes the nitrogen anion **1‐B** to N‐radical **1‐C** (Path B). The resulting radical **1‐C** can be trapped by O_2_ or ·O_2_^−^ to deliver peroxide species (Path D), which undergoes a reduction process to give the alcohol products.

## 3 Conclusion

In conclusion, we have developed an efficient and robust heterogeneous photocatalytic system for the direct transformation of N─H and O─H bonds into nitrogen and oxygen radicals by a readily available NCN catalyst. This NCN catalyst exhibits a significant improvement in the separation and transfer of photoexcited charge carriers, achieving a range of N/O‐radical carboamination, oxyamination and deoxygenation of alkenes under mild conditions. Remarkably, the NCN catalyst can be further applied for the aerobic oxidation of boronic acids and controllable oxidation of alcohols to aldehydes and carboxylic acids, demonstrating its robust photoredox activity. Additionally, the catalyst exhibits exceptional durability, with its reactivity and efficiency remaining steadfast through multiple utilization cycles. This consistent performance not only underscores its robustness but also signals its significant potential in practical synthesis.

## 4 Experimental Section

### General Procedure for Radical Carboamination Reaction

To an over dried bottle equipped with a magnetic stir bar, β,γ‐unsaturated hydrazone (37.6 mg, 0.1 mmol), nitrosobenzene (0.3 mmol), K_2_CO_3_ (20.7 mg, 0.15 mmol), NCN (5 mg) were added. After that, MeCN (2 mL) was added to the bottle in the glovebox. The mixture was stirred under 405 nm LED irradiation for 12 h at room temperature. The crude product was purified by column chromatography (SiO_2_, petroleum ether/ethyl acetate = 15:1 to 3:1) to give the pure desired product.

### General Procedure for Hydrolysis of Nitrones

To an over‐dried bottle equipped with a magnetic stir bar, nitrone (47.3 mg, 0.098 mmol), 0.1 M HCl (3 mL), MeCN (2 mL) were added. After that, the mixture was stirred under air for 12 h at room temperature. The crude product was purified by column chromatography (SiO_2_, petroleum ether/ethyl acetate = 30:1 to 10:1) to give the pure desired product.

### General Procedure for the Oxyamination and Deoxygenation Reactions

To an over‐dried bottle equipped with a magnetic stir bar, β,γ‐unsaturated hydrazone (75.2 mg, 0.2 mmol), TEMPO (6.25 mg, 0.04 mmol), K_2_CO_3_ (55.3 mg, 0.4 mmol), NCN (5 mg) were added. After that, MeCN (2 mL) was added to the bottle under a balloon of O_2_. The mixture was stirred under 405 nm LED irradiation for 12 h at room temperature. The crude product was purified by column chromatography (SiO_2_, petroleum ether/ethyl acetate = 15:1 to 3:1) to give the pure desired product.

### General Procedure for the Oxidation of Boronic Acids

To an over‐dried bottle equipped with a magnetic stir bar, boronic acids (34.4 mg, 0.2 mmol), NCN (5 mg), Et_3_N (40.5 mg, 0.4 mmol) were added. After that, MeCN (2 mL) was added to the bottle connected with an O_2_ balloon. The mixture was stirred under 405 nm LED irradiation for 10 h at room temperature. The crude product was purified by column chromatography (SiO_2_, petroleum ether/ethyl acetate = 20:1) to give the pure desired product.

### General Procedure for Selective Oxidation of Alcohols

To an over‐dried bottle equipped with a magnetic stir bar, alcohols (36.4 mg, 0.2 mmol), NCN (5 mg) were added. After that, DMSO (2 mL) was added to the bottle connected with an O_2_ balloon. The mixture was stirred under 405 nm LED irradiation for 10 h at room temperature. The crude product was purified by column chromatography (SiO_2_, petroleum ether/ethyl acetate = 20:1) to give the pure desired product.

## Conflict of Interest

The authors declare no conflict of interest.

## Supporting information



Supporting Information

## Data Availability

The data that support the findings of this study are available in the supplementary material of this article.
