# Supplementary material for: Enhanced Photoredox Activity in Nitrogen‐Doped Carbon Nitride for Heterogeneous Nitrogen and Oxygen Radical Reactions
Source: Adv Sci (Weinh). 2025 Feb 22;12(15):2417752. doi: 10.1002/advs.202417752 (PMC12005771; doi:10.1002/advs.202417752)

ADVANCED SCIENCE
Open Access

Supporting Information

for *Adv. Sci.*, DOI 10.1002/advs.202417752

Enhanced Photoredox Activity in Nitrogen-Doped Carbon Nitride for Heterogeneous Nitrogen and Oxygen Radical Reactions

Lan Qin, Huan Liu, Yi Wei, Lian-Qing Chen and Xiao-Qiang Hu**

Supporting Information for

Enhanced Photoredox Activity in Nitrogen-Doped Carbon Nitride for Heterogeneous Nitrogen and Oxygen Radical Reactions

Lan Qin, Huan Liu, Yi Wei, Lian-Qing Chen* and Xiao-Qiang Hu*

Key Laboratory of Catalysis and Energy Materials Chemistry of Ministry of Education & Hubei Key Laboratory of Catalysis and Materials Science, School of Chemistry and Materials Science, South-Central Minzu University, Wuhan 430074, China.

hxq071303127@126.com

Table of Contents

1. General information 3

2. Supplementary Figures and Tables....................4

2.1 The high-resolution C 1s spectra and Mott-Schottky (MS) plots 4

2.2 Nitrogen absorption and desorption analysis 5

2.3 TPRL of catalysts 6

2.4 The reaction of catalysts with different amounts of citric acid in various reactions 6

2.5 XPS characterization of recovered catalyst 7

3. General Procedure for Photocatalytic Reactions and Characterization of Products 8

3.1 Experimental setup....................8

3.2 General procedure for preparation of catalysts....................8

3.3 General procedure for preparation of products 9

3.3 Procedure for Gram-Scale Reaction 43

4. X-Ray crystallographic data of compound 9....................44

5. Mechanistic Studies....................46

5.1 UV-vis spectrum....................46

5.2 TEMPO Trapping Experiment 46

5.3 Determination of Quantum Yield 48

6. NMR Spectra of products 50

1. General information

Unless otherwise noted, materials were purchased from commercial suppliers (Alfa, TCI, Sigma-Aldrich and J&K Scientific etc.), and used without further purification. All the solvents were treated according to general methods. All reactions were monitored by thin-layer chromatography (TLC) on silica gel plates using UV light as visualizing agent (if applicable). Flash column chromatography was performed using 200-300 mesh silica gel. ^{1}H NMR spectra were recorded on 400 MHz spectrophotometers. Chemical shifts are reported in delta (δ (ppm)) units in parts per million (ppm) relative to the singlet (0 ppm) for tetramethylsilane (TMS). Data are reported as follows: chemical shift, multiplicity (s = singlet, d = doublet, t = triplet, q = quartet, dd = doublet of doublets, m = multiplet), coupling constants (Hz) and integration. ^{13}C NMR spectra were recorded on Varian Mercury 100 MHz with complete proton decoupling spectrophotometers ($CDCl_3$: 77.0 ppm). The high-resolution mass spectra (HRMS) were measured on a Shimadzu LCMS-IT-TOF mass spectrometer or DIONEX UltiMate 3000 & Bruker Compact TOF mass spectrometer by ESI. Measured values are reported to 4 decimal places of the calculated value. The calculated values are based on the most abundant isotope.

Instrument information is given in the table 1.

Table S1 Instrument information

Instrument name	Instrument model	Manufacture
Muffle	KSL-1200X	Hefei Kejing Material Technology Co., LTD
ultraviolet and visible spectrophotometer	ShimadzuUV2600	Shimadzu Corporation
Field emission electron microscope	SU8010	Babcock Hitachi
scanning transmission electron microscopy	TALOS-F200X	Thermo Fisher Technology finite formula
X-ray photoelectron spectrometer	EscalabXi+	Thermo Fisher Technology finite formula
fluorescence spectrophotometer	LS-55	American PE companies
fourier infrared spectrometer	Nexus470	Nicoli Instruments
X-ray diffractometer	D8-advance	Bruck

2. Supplementary Figures and Tables

2.1 The high-resolution C 1s spectra and Mott-Schottky (MS) plots

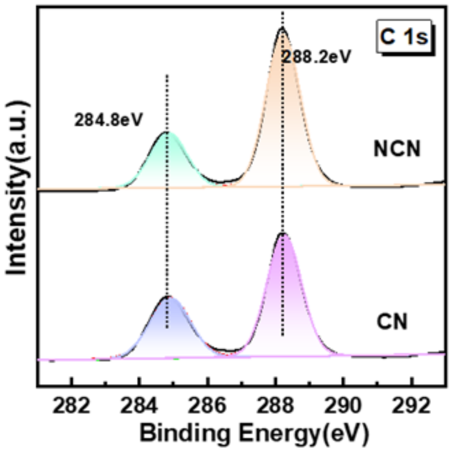

Figure S1 The high-resolution C 1s spectra of g-C_3N_4 and NCN

The high-resolution C 1s XPS data of the original g-C_3N_4 and NCN are shown in Figure S1, fitted with two distinct peaks with similar spectra. Peaks at 284.8 and 288.2 eV are attributed to sp^2-hybridized carbon in C-C/C=C and the aromatic ring (N-C=N), respectively. Importantly, the peak at 288.2 eV for NCN is stronger compared to g-C_3N_4 (65.14% vs. 62.35%).

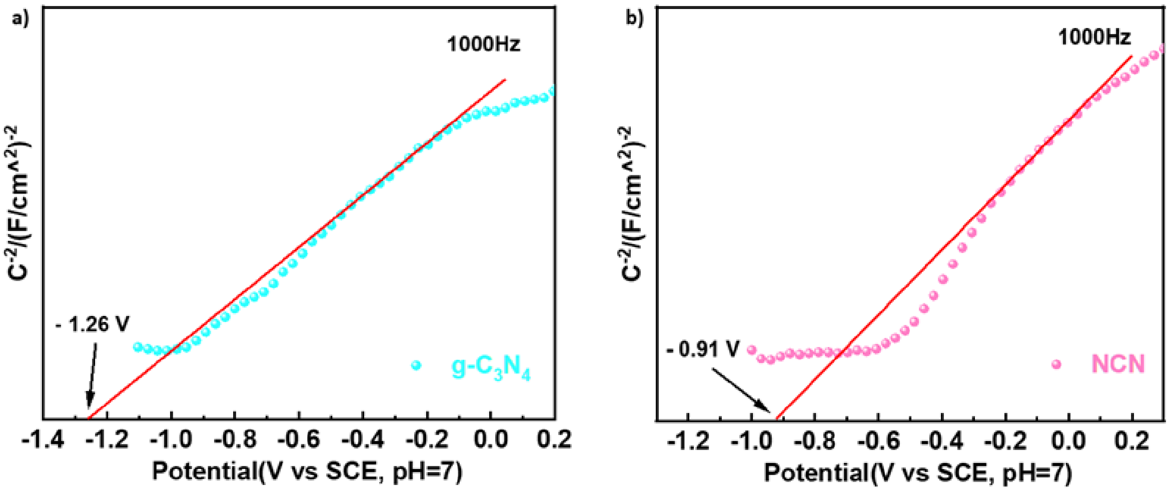

Figure S2 The Mott-Schottky (MS) plots of a) g-C_3N_4 and b) NCN

As shown in Figure S2 a and b, the Mott-Schottky (MS) plots of g-C_3N_4 and NCN samples all exhibit positive slopes, indicating n-type semiconductor characteristics. The flat band potentials of g-C_3N_4 and

NCN are -1.26V and -0.91V (vs SCE, pH=7), converted to -0.61V, and -0.26V (vs RHE). Generally, the conduction band potential (CB) of n-type semiconductors is 0-0.2V lower than their flat band potentials. Therefore, by calculation, the CB values for g-C_3N_4 and NCN are -0.81V and -0.46V, respectively. Combining with the band gaps determined by UV-Visible spectroscopy and using the formula (EVB = ECB + Eg), the valence band (VB) potentials of g-C_3N_4 and NCN are calculated as 1.78V, and 1.74V, respectively.

2.2 Nitrogen absorption and desorption analysis

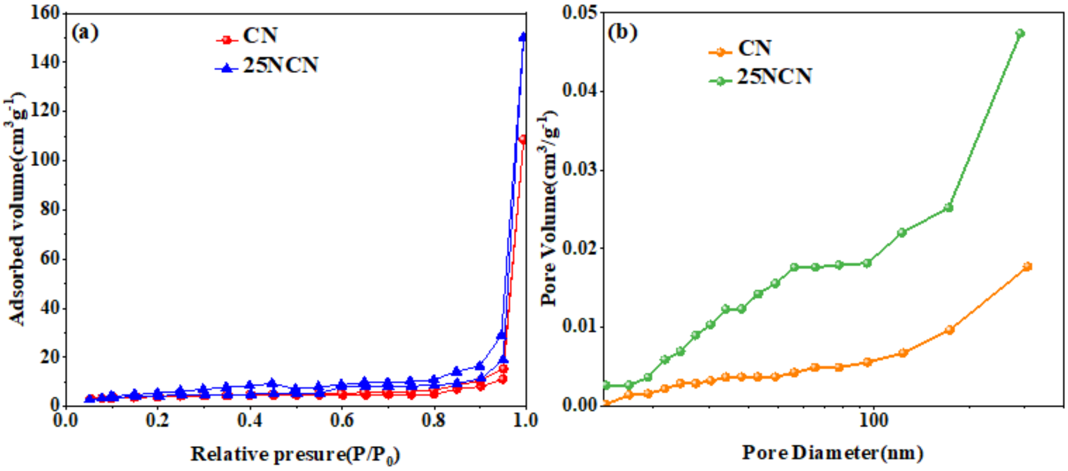

Figure S3 N_2 ad-desorption isotherms and pore size distributions of CN and NCN catalysts.

Table S2 The BET of CN and NCN catalysts.

sample	BET area （m^2/g）	average pore diameter (nm)	pore volume (cm^3/g)
CN	13.68	48.76	0.169
NCN-25	16.84	58.96	0.238
NCN-15	14.98	55.69	0.231
NCN-10	15.64	57.76	0.216

To better understand the specific surface area and pore size distribution of NCN catalysts, nitrogen adsorption-desorption isotherm analysis on as-prepared NCN catalysts were performed. As shown in Figure S3, the BET test indicated that NCN catalysts had mesoporous structures. The specific surface area (16.84 m^2/g) and pore volume (0.238 cm^3/g) of NCN-25 were slightly larger than those of CN (13.68 m^2/g) and pore volume (0.169 cm^3/g), which was shown in Table S2. The increase of pore diameter and specific surface area favored the exposure of more active sites, which enhanced the adsorption of the reactants and thus improved the photocatalytic performance.

2.3 TPRL of catalysts

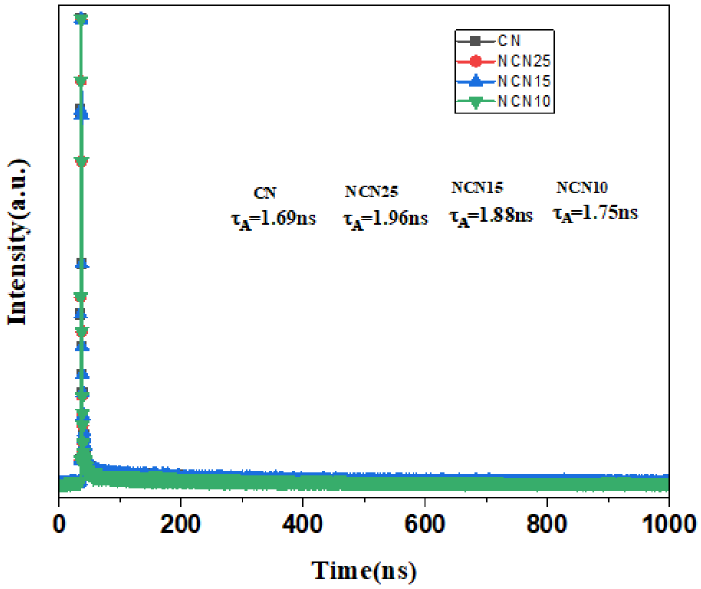

Figure S4 TPRL of CN and NCN catalysts

As shown in Figure S4, NCN catalyst had the smallest arc radius, so it had the minimum charge transfer resistance and superior charge migration. According to the following equation: τA = (A1τ12+ A1τ22)/(A1τ1+ A1τ2), A is the prefactor and τ is the radiation lifetime, it was obtained that NCN catalysts had the longest average fluorescence lifetime which were attributed to the strong interaction with reactants that promoted the transfer of photogenerated carriers.

2.4 The effect of NCN with varying levels of defects

We adjusted the amount of citric acid (10, 15, and 25 mg) to synthesize NCN with varying levels of defects, designated as NCN-10, NCN-15, and NCN-25 (as used in the manuscript). The catalysts were tested in three types of reactions. In the carbamination and oxyamination reactions, NCN-25 demonstrated higher efficiency compared to NCN-10 and NCN-15. Similar results were observed for boronic oxidation. We conducted nitrogen absorption and desorption analysis on g-C_3N_4, NCN-25 (used in our manuscript), NCN-10, and NCN-15 to compare their surface areas (Table S2), with NCN-25 exhibiting the largest specific surface area. In our experiments, NCN-25 demonstrated higher efficiency than both NCN-10 and NCN-15. These results suggest that a larger surface area of the catalyst may contribute to enhanced catalytic efficiency.

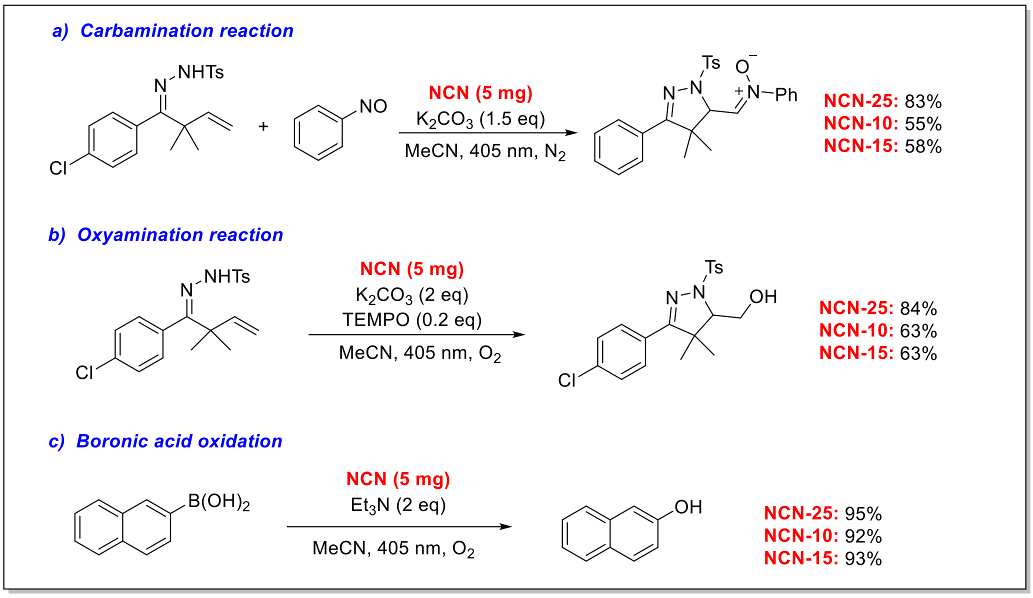

Figure S5 The reaction of NCN catalyst with varying levels of defects

2.5 XPS characterization of recovered catalyst

From the perspective of the full spectrum of XPS, the recovered catalyst oxygen content increased, the C1s spectrum increased by a small peak of 286.6, the other peaks did not change, and the displacement of the peak in the N1s spectrum did not change, indicating that the overall structure of the catalyst did not change, but a small part of carbon was oxidized at the edge to form a C=O bond.

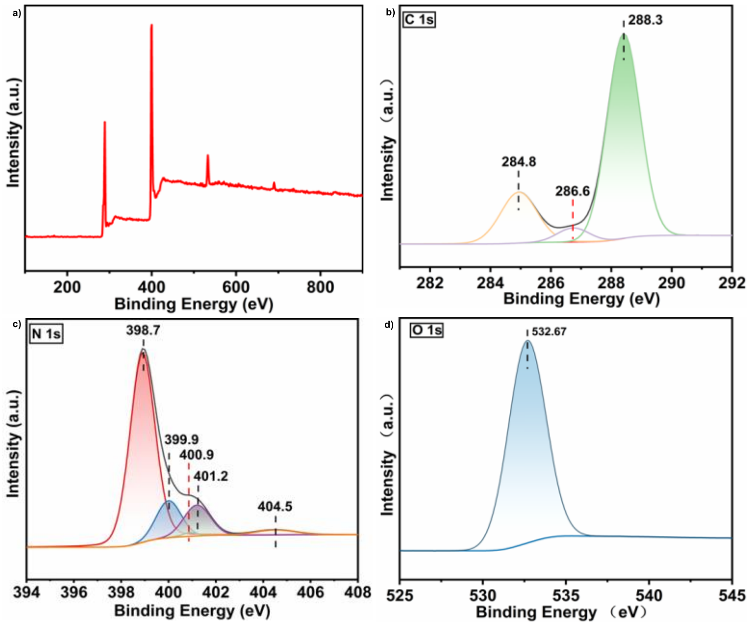

Figure S6 XPS characterization of recovered catalyst

3. General Procedure for Photocatalytic Reactions and Characterization of Products

3.1 Experimental setup

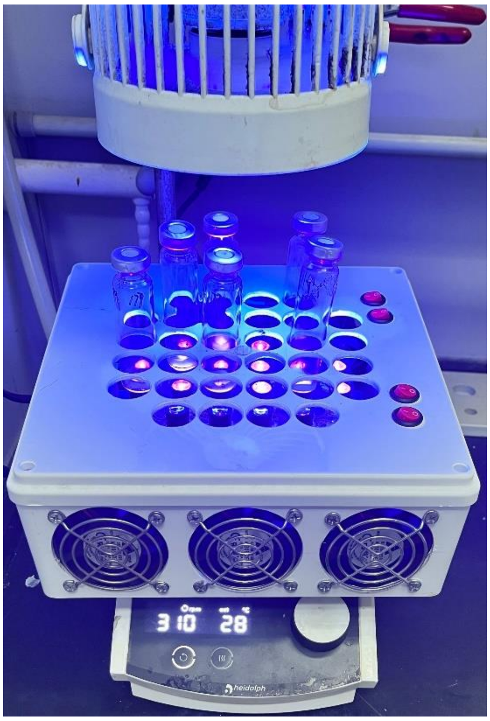

Figure S7 The setting-up reactions.

We used a commercially available 405 nm LEDs lamp (5W) (For more details: Wuhan Jinbo Tianhua instrument equipment Co., LTD). The material of the reaction vessels are regular borosilicate glass.

3.2 General procedure for preparation of catalysts

Experimental procedure for the synthesis of NCN:

Urea (25 g) was mixed with 25 mg citric acid, dissolved in 80ml deionized water, ultrasonic at 80℃, rotary steaming, and then calcined in the air at 2 °C min^{-1} temperature in a covered crucible at 550℃ for 4h to prepare NCN (as used in the manuscript). [19] We also adjusted the amount of citric acid (10 mg and 15 mg) to synthesize NCN with varying levels of defects, designated as NCN-10 and NCN-15.

Experimental procedure for the synthesis of CN:

The pure g-C_3N_4 was prepared by directly heating urea (20 g) at 550 °C for 4 h, with a ramp rate of 2 °C min^{-1} in air. The product denoted as CN. [19]

3.3 General procedure for preparation of products

Product 3 (unknown compound)

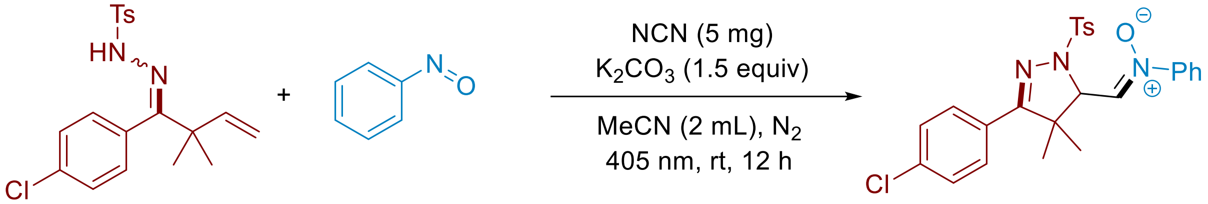

3' **2** **3**

To an over dried bottle equipped with a magnetic stir bar, **3'** (37.6 mg, 0.1 mmol), **2** (32.1 mg, 0.3 mmol), K_2CO_3 (20.7 mg, 0.15 mmol), NCN (5 mg) were added. After that, MeCN (2 mL) was added to the bottle in glovebox. The mixture was stirred under 405 nm LED irradiation for 12 h at room temperature. The product **3** was purified by column chromatography (SiO_2, petroleum ether/ethyl acetate = 15:1 to 3:1), yielding **3** as light brown solid (40 mg, 83% yield). In a nuclear magnetic tube, 40 mg of pure product was dissolved in 0.5 mL of deuterated chloroform and tested by 400M NMR.

^{1}H NMR (400 MHz, $CDCl_3$) δ = 7.86 – 7.77 (m, 4H), 7.62 – 7.54 (m, 3H), 7.49-7.47 (m, 3H), 7.35-7.31 (m, 4H), 4.92 (d, J = 6.6 Hz, 1H), 2.40 (s, 3H), 1.46 (s, 3H), 1.39 (s, 3H). **^{13}C NMR (101 MHz, $CDCl_3$)** δ = 163.5, 147.0, 144.9, 136.3, 134.4, 130.6, 130.1, 129.6, 129.2, 129.0, 128.8, 128.7, 128.2, 121.8, 69.1, 54.6, 25.5, 21.6, 21.3. HRMS (ESI-TOF) m/z: $[M + H]^+$ Calcd for $C_{25}H_{24}ClN_3O_3S$ 482.1300; Found 482.1300.

Product 5 (unknown compound)

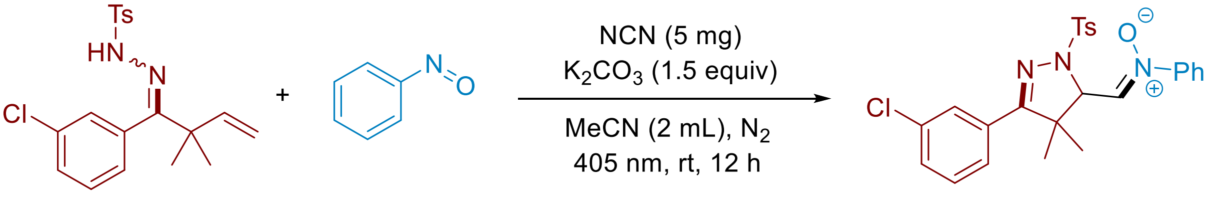

5' **2** **5**

To an over dried bottle equipped with a magnetic stir bar, **5'** (37.6 mg, 0.1 mmol), **2** (32.1 mg, 0.3 mmol), K_2CO_3 (20.7 mg, 0.15 mmol), NCN (5 mg) were added. After that, MeCN (2 mL) was added to the bottle in glovebox. The mixture was stirred under 405 nm LED irradiation for 12 h at room temperature. The product **5** was purified by column chromatography (SiO_2, petroleum ether/ethyl acetate = 15:1 to 3:1), yielding **5** as light brown solid (42.8 mg, 89% yield). In a nuclear magnetic tube, 42.8 mg of pure product was dissolved in 0.5 mL of deuterated chloroform and tested by 400M NMR.

^{1}H NMR (400 MHz, $CDCl_3$) δ = 7.88 – 7.78 (m, 4H), 7.63 – 7.58 (m, 2H), 7.53 – 7.47 (m, 4H), 7.40 –

7.35 (m, 3H), 7.30 (t, J = 7.9 Hz, 1H), 4.93 (d, J = 6.7 Hz, 1H), 2.44 (s, 3H), 1.48 (s, 3H), 1.40 (s, 3H). **^{13}C NMR (101 MHz, CDCl3)** δ = 163.5, 147.2, 145.1, 134.6, 134.4, 131.7, 130.7, 130.3, 130.2, 129.8, 129.8, 129.3, 129.2, 127.7, 125.6, 121.9, 69.2, 54.7, 25.6, 21.7, 21.4. HRMS (ESI-TOF) m/z: $[M + H]^+$ Calcd for $C_{25}H_{24}ClN_3O_3S$ 482.1300; Found 482.1298.

Product 6 (unknown compound)

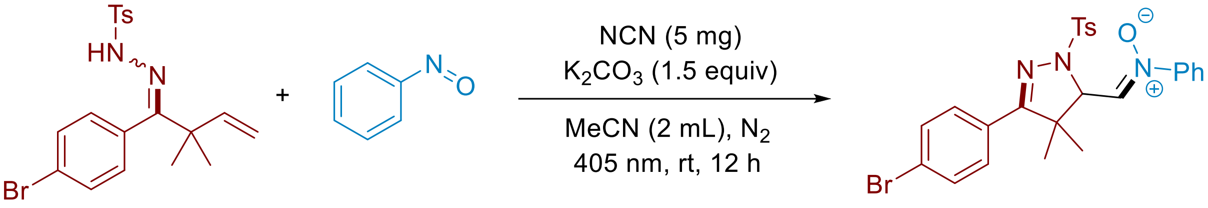

6' 2 6

To an over dried bottle equipped with a magnetic stir bar, **6’** (42.1 mg, 0.1 mmol), **2** (32.1 mg, 0.3 mmol), K_2CO_3 (20.7 mg, 0.15 mmol), NCN (5 mg) were added. After that, MeCN (2 mL) was added to the bottle in glovebox. The mixture was stirred under 405 nm LED irradiation for 12 h at room temperature. The product **6** was purified by column chromatography (SiO_2, petroleum ether/ethyl acetate = 15:1 to 3:1), yielding **6** as light brown solid (39.5 mg, 75% yield). In a nuclear magnetic tube, 39.5 mg of pure product was dissolved in 0.5 mL of deuterated chloroform and tested by 400M NMR.

^{1}H NMR (400 MHz, $CDCl_3$) δ = 7.84 (d, J = 8.3 Hz, 2H), 7.82 – 7.77 (m, 2H), 7.60 (d, J = 6.6 Hz, 1H), 7.51-7.49 (m, 7H), 7.35 (d, J = 8.0 Hz, 2H), 4.91 (d, J = 6.6 Hz, 1H), 2.42 (s, 3H), 1.47 (s, 3H), 1.39 (s, 3H). **^{13}C NMR (101 MHz, $CDCl_3$)** δ = 163.7, 147.1, 145.0, 134.5, 131.8, 130.7, 130.1, 129.7, 129.2, 129.1, 129.0, 128.7, 124.8, 121.9, 69.2, 54.6, 25.6, 21.7, 21.4. HRMS (ESI-TOF) m/z: $[M + H]^+$ Calcd for $C_{25}H_{24}BrN_3O_3S$ 526.0795; Found 526.0797.

Product 7 (unknown compound)

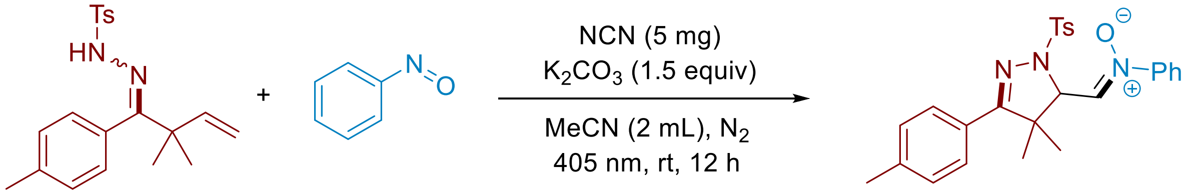

7' 2 7

To an over dried bottle equipped with a magnetic stir bar, **7’** (35.6 mg, 0.1 mmol), **2** (32.1 mg, 0.3 mmol), **K_2CO_3** (20.7 mg, 0.15 mmol), **NCN** (5 mg) were added. After that, MeCN (2 mL) was added to the bottle in glovebox. The mixture was stirred under 405 nm LED irradiation for 12 h at room temperature. The product **7** was purified by column chromatography (SiO_2, petroleum ether/ethyl acetate = 15:1 to 3:1),

yielding **7** as light brown solid (38 mg, 82% yield). In a nuclear magnetic tube, 38 mg of pure product was dissolved in 0.5 mL of deuterated chloroform and tested by 400M NMR.

^{1}H NMR (400 MHz, CDCl$_3$) δ = 7.86 (d, *J* = 8.3 Hz, 2H), 7.83 – 7.78 (m, 2H), 7.61 (d, *J* = 6.7 Hz, 1H), 7.56 – 7.48 (m, 5H), 7.35 (d, *J* = 8.0 Hz, 2H), 7.17 (d, *J* = 8.0 Hz, 2H), 4.89 (d, *J* = 6.7 Hz, 1H), 2.42 (s, 3H), 2.36 (s, 3H), 1.47 (s, 3H), 1.41 (s, 3H). **^{13}C NMR (101 MHz, CDCl$_3$)** δ = 164.7, 147.2, 144.8, 140.6, 135.0, 130.7, 130.2, 129.6, 129.2, 129.2, 127.5, 127.0, 121.9, 69.1, 54.8, 25.7, 21.7, 21.5, 21.4. HRMS (ESI-TOF) m/z: [M + H]$^+$ Calcd for $C_{26}H_{27}N_3O_3S$ 426.1846; Found 462.1819.

Product 8 (unknown compound)

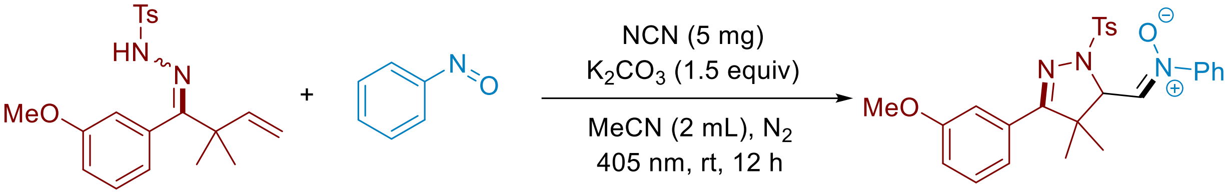

8' **2** **8**

To an over dried bottle equipped with a magnetic stir bar, **8'** (18.6 mg, 0.05 mmol), **2** (16.0 mg, 0.15 mmol), K_2CO_3 (10.4 mg, 0.075 mmol), NCN (2.5 mg) were added. After that, MeCN (1 mL) was added to the bottle in glovebox. The mixture was stirred under 405 nm LED irradiation for 12 h at room temperature. The product **8** was purified by column chromatography (SiO_2, petroleum ether/ethyl acetate = 15:1 to 3:1), yielding **8** as light brown solid (16.9 mg, 69% yield). In a nuclear magnetic tube, 16.9 mg of pure product was dissolved in 0.5 mL of deuterated chloroform and tested by 400M NMR.

^{1}H NMR (400 MHz, CDCl$_3$) δ = 7.88 – 7.78 (m, 4H), 7.61 (d, *J* = 6.7 Hz, 1H), 7.53 – 7.47 (m, 3H), 7.35 (d, *J* = 8.1 Hz, 2H), 7.29 (d, *J* = 7.9 Hz, 1H), 7.20 – 7.14 (m, 2H), 6.96-6.93 (m, 1H), 4.91 (d, *J* = 6.6 Hz, 1H), 3.83 (s, 3H), 2.43 (s, 3H), 1.47 (s, 3H), 1.41 (s, 3H). **^{13}C NMR (101 MHz, CDCl$_3$)** δ = 164.7, 159.5, 147.2, 144.9, 134.7, 131.1, 130.7, 130.2, 129.7, 129.5, 129.3, 129.2, 121.9, 120.0, 115.7, 113.4, 69.2, 55.3, 54.9, 25.7, 21.7, 21.5. HRMS (ESI-TOF) m/z: [M + H]$^+$ Calcd for $C_{26}H_{27}N_3O_4S$ 478.1795; Found 478.1794.

Product 9 (unknown compound)

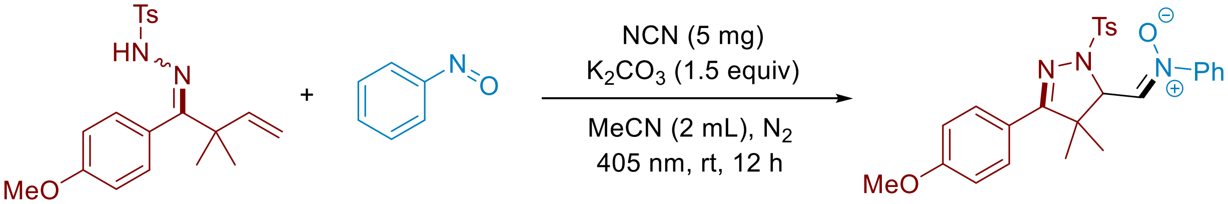

9' **2** **9**

To an over dried bottle equipped with a magnetic stir bar, **9'** (37.2 mg, 0.1 mmol), **2** (32.1 mg, 0.3 mmol),

K_2CO_3 (20.7 mg, 0.15 mmol), NCN (5 mg) were added. After that, MeCN (2 mL) was added to the bottle in glovebox. The mixture was stirred under 405 nm LED irradiation for 12 h at room temperature. The product **9** was purified by column chromatography (SiO_2, petroleum ether/ethyl acetate = 15:1 to 3:1), yielding **9** as light brown solid (39 mg, 82% yield). In a nuclear magnetic tube, 39 mg of pure product was dissolved in 0.5 mL of deuterated chloroform and tested by 400M NMR.

^{1}H NMR (400 MHz, $CDCl_3$) δ = 7.86 (d, J = 8.3 Hz, 2H), 7.83 – 7.76 (m, 2H), 7.64 – 7.57 (m, 3H), 7.52 – 7.44 (m, 3H), 7.34 (d, J = 8.1 Hz, 2H), 6.88 (d, J = 8.9 Hz, 2H), 4.89 (d, J = 6.7 Hz, 1H), 3.82 (s, 3H), 2.42 (s, 3H), 1.48 (s, 3H), 1.41 (s, 3H). **^{13}C NMR (101 MHz, $CDCl_3$)** δ = 164.3, 161.1, 147.2, 144.8, 134.9, 130.6, 130.2, 129.6, 129.2, 129.1, 122.2, 121.9, 113.9, 69.0, 55.3, 54.7, 25.7, 21.6, 21.5. HRMS (ESI-TOF) m/z: $[M + H]^+$ Calcd for $C_{26}H_{27}N_3O_4S$ 478.1795; Found 478.1794.

Product 10 (unknown compound)

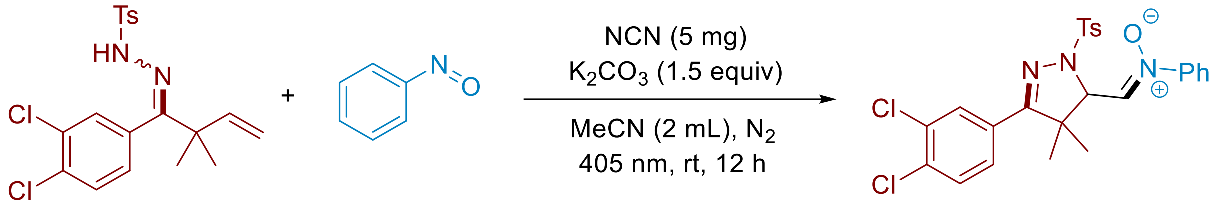

10' **2** **10**

To an over dried bottle equipped with a magnetic stir bar, **10'** (41.0 mg, 0.1 mmol), **2** (32.1 mg, 0.3 mmol), K_2CO_3 (20.7 mg, 0.15 mmol), NCN (5 mg) were added. After that, MeCN (2 mL) was added to the bottle in glovebox. The mixture was stirred under 405 nm LED irradiation for 12 h at room temperature. The product **10** was purified by column chromatography (SiO_2, petroleum ether/ethyl acetate = 15:1 to 3:1), yielding **10** as light brown solid (34 mg, 66% yield). In a nuclear magnetic tube, 34 mg of pure product was dissolved in 0.5 mL of deuterated chloroform and tested by 400M NMR.

^{1}H NMR (400 MHz, $CDCl_3$) δ = 7.87 – 7.82 (m, 2H), 7.82 – 7.76 (m, 2H), 7.72 (d, J = 1.9 Hz, 1H), 7.59 (d, J = 6.6 Hz, 1H), 7.53 – 7.49 (m, 3H), 7.48 – 7.41 (m, 2H), 7.36 (d, J = 8.0 Hz, 2H), 4.93 (d, J = 6.7 Hz, 1H), 2.43 (s, 3H), 1.48 (s, 3H), 1.39 (s, 3H). **^{13}C NMR (101 MHz, $CDCl_3$)** δ = 162.4, 147.1, 145.2, 134.6, 134.2, 133.0, 130.8, 130.6, 130.2, 129.8, 129.8, 129.4, 129.3, 129.1, 126.6, 121.9, 69.3, 54.6, 25.7, 21.7, 21.3. HRMS (ESI-TOF) m/z: $[M + H]^+$ Calcd for $C_{25}H_{23}Cl_2N_3O_3S$ 516.0910; Found 516.0911.

Product 11 (unknown compound)

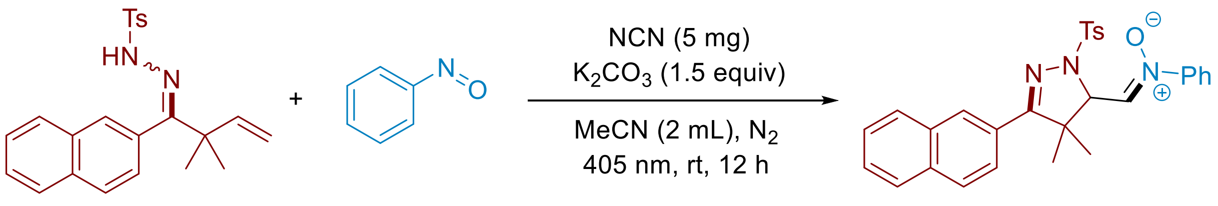

To an over dried bottle equipped with a magnetic stir bar, **11'** (39.2 mg, 0.1 mmol), **2** (32.1 mg, 0.3 mmol), K_2CO_3 (20.7 mg, 0.15 mmol), NCN (5 mg) were added. After that, MeCN (2 mL) was added to the bottle in glovebox. The mixture was stirred under 405 nm LED irradiation for 12 h at room temperature. The product **11** was purified by column chromatography (SiO_2, petroleum ether/ethyl acetate = 15:1 to 3:1), yielding **11** as light brown solid (39.2 mg, 79% yield). In a nuclear magnetic tube, 39.2 mg of pure product was dissolved in 0.5 mL of deuterated chloroform and tested by 400M NMR.

^{1}H NMR (400 MHz, $CDCl_3$) δ= 8.05 (s, 1H), 7.91 (d, J = 8.3 Hz, 2H), 7.84 – 7.82 (m, 6H), 7.65 (d, J = 6.7 Hz, 1H), 7.56 – 7.45 (m, 5H), 7.36 (d, J = 8.1 Hz, 2H), 4.98 (d, J = 6.7 Hz, 1H), 2.42 (s, 3H), 1.60 (s, 3H), 1.51 (s, 3H). **^{13}C NMR (101 MHz, $CDCl_3$)** δ = 164.5, 147.2, 144.9, 134.8, 133.9, 132.6, 130.7, 130.2, 129.7, 129.2, 129.2, 128.5, 128.3, 127.7, 127.4, 127.3, 127.2, 126.7, 124.8, 121.9, 69.2, 54.9, 25.9, 21.7.HRMS (ESI-TOF) m/z: $[M + H]^+$ Calcd for $C_{29}H_{27}N_3O_3S$ 498.1846; Found 498.1822.

Product 12 (unknown compound)

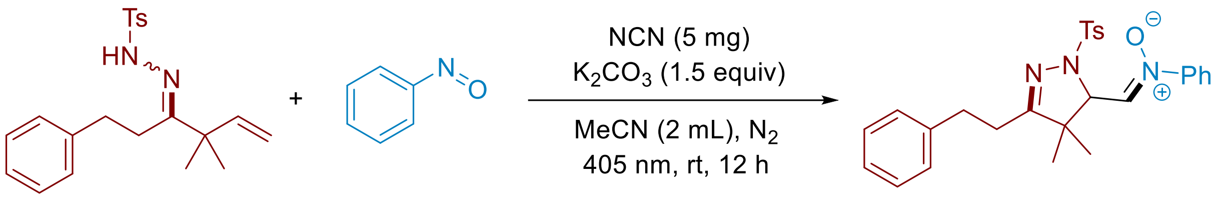

To an over dried bottle equipped with a magnetic stir bar, **12'** (37.0 mg, 0.1 mmol), **2** (32.1 mg, 0.3 mmol), K_2CO_3 (20.7 mg, 0.15 mmol), NCN (5 mg) were added. After that, MeCN (2 mL) was added to the bottle in glovebox. The mixture was stirred under 405 nm LED irradiation for 12 h at room temperature. The product **12** was purified by column chromatography (SiO_2, petroleum ether/ethyl acetate = 15:1 to 3:1), yielding **12** as light brown solid (41.5 mg, 87% yield). In a nuclear magnetic tube, 41.5 mg of pure product was dissolved in 0.5 mL of deuterated chloroform and tested by 400M NMR.

^{1}H NMR (400 MHz, $CDCl_3$) δ = 7.82 – 7.73 (m, 4H), 7.57 – 7.45 (m, 4H), 7.33 (d, J = 8.0 Hz, 2H), 7.29 – 7.18 (m, 3H), 7.14 – 7.09 (m, 2H), 4.69 (d, J = 6.4 Hz, 1H), 3.04 – 2.97 (m, 1H), 2.87 – 2.80 (m, 1H), 2.51 – 2.37 (m, 5H), 1.15 (s, 3H), 1.10 (s, 3H). **^{13}C NMR (101 MHz, $CDCl_3$)** δ = 168.5, 147.0, 144.7,

140.9, 135.6, 130.6, 130.2, 129.5, 129.2, 129.2, 128.5, 128.3, 126.3, 121.8, 67.8, 55.2, 32.3, 27.7, 24.8, 21.7, 20.3. HRMS (ESI-TOF) m/z: $[M + H]^+$ Calcd for $C_{27}H_{29}N_3O_3S$ 476.2002; Found 476.2002.

Product 13 (unknown compound)

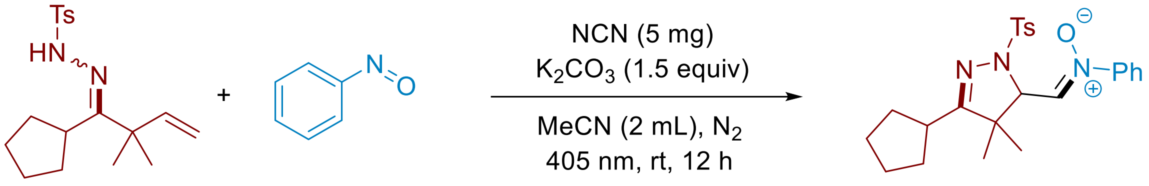

13' **2** **13**

To an over dried bottle equipped with a magnetic stir bar, **13’** (33.4 mg, 0.1 mmol), **2** (32.1 mg, 0.3 mmol), K_2CO_3 (20.7 mg, 0.15 mmol), NCN (5 mg) were added. After that, MeCN (2 mL) was added to the bottle in glovebox. The mixture was stirred under 405 nm LED irradiation for 12 h at room temperature. The product **13** was purified by column chromatography (SiO_2, petroleum ether/ethyl acetate = 15:1 to 3:1), yielding **13** as light brown solid (33.1 mg, 75% yield). In a nuclear magnetic tube, 33.1 mg of pure product was dissolved in 0.5 mL of deuterated chloroform and tested by 400M NMR.

^{1}H NMR (400 MHz, $CDCl_3$) δ = 7.80 – 7.72 (m, 4H), 7.55-7.53 (m, 1H), 7.50-7.46 (m, 3H), 7.32 (d, *J* = 8.0 Hz, 2H), 4.62 (d, *J* = 6.3 Hz, 1H), 2.43 (s, 4H), 1.89 – 1.69 (m, 6H), 1.62 – 1.48 (m, 3H), 1.20 (s, 3H), 1.13 (s, 3H). **^{13}C NMR (101 MHz, $CDCl_3$)** δ 173.4, 147.0, 144.6, 136.0, 130.6, 129.9, 129.3, 129.2, 121.8, 68.1, 55.5, 36.9, 32.7, 25.7, 25.3, 25.2, 21.7, 20.7. HRMS (ESI-TOF) m/z: $[M + H]^+$ Calcd for $C_{18}H_{24}N_2O_3S$ 349.1580; Found 349.1580.

Product 14 (unknown compound)

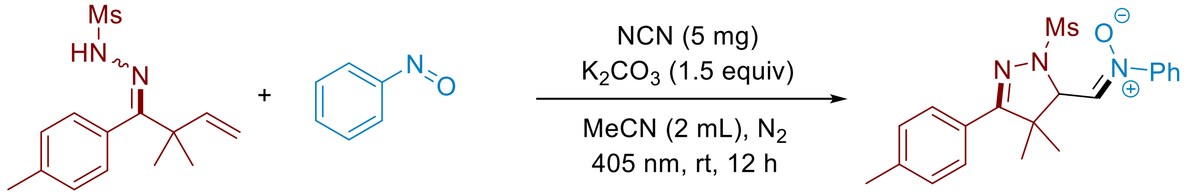

14' **2** **14**

To an over dried bottle equipped with a magnetic stir bar, **14’** (28 mg, 0.1 mmol), **2** (32.1 mg, 0.3 mmol), K_2CO_3 (20.7 mg, 0.15 mmol), NCN (5 mg) were added. After that, MeCN (2 mL) was added to the bottle in glovebox. The mixture was stirred under 405 nm LED irradiation for 12 h at room temperature. The product **14** was purified by column chromatography (SiO_2, petroleum ether/ethyl acetate = 15:1 to 3:1), yielding **14** as light brown solid (36.3 mg, 94% yield). In a nuclear magnetic tube, 36.3 mg of pure product was dissolved in 0.5 mL of deuterated chloroform and tested by 400M NMR.

^{1}H NMR (400 MHz, CDCl$_3$) δ = 7.79 – 7.70 (m, 2H), 7.68 – 7.61 (m, 2H), 7.53 (d, *J* = 6.5 Hz, 1H), 7.50 – 7.43 (m, 3H), 7.22 (d, *J* = 8.0 Hz, 2H), 5.32 (d, *J* = 6.5 Hz, 1H), 3.09 (s, 3H), 2.39 (s, 3H), 1.84 (s, 3H), 1.44 (s, 3H). **^{13}C NMR (101 MHz, CDCl$_3$)** δ = 165.0, 147.1, 140.8, 134.6, 130.6, 129.3, 129.2, 127.7, 126.8, 121.8, 68.7, 55.3, 34.6, 25.9, 21.4, 21.3. HRMS (ESI-TOF) m/z: [M + H]$^+$ Calcd for $C_{20}H_{23}N_3O_3S$ 386.1533; Found 386.1522.

Product 15 (unknown compound)

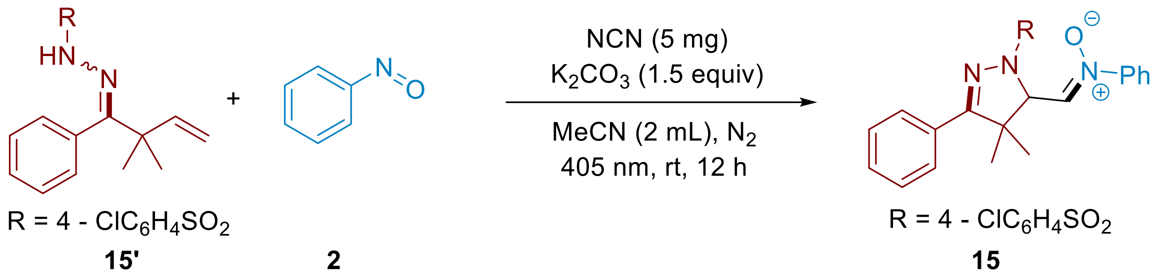

To an over dried bottle equipped with a magnetic stir bar, **15'** (36.2 mg, 0.1 mmol), **2** (32.1 mg, 0.3 mmol), K_2CO_3 (20.7 mg, 0.15 mmol), NCN (5 mg) were added. After that, MeCN (2 mL) was added to the bottle in glovebox. The mixture was stirred under 405 nm LED irradiation for 12 h at room temperature. The product **15** was purified by column chromatography (SiO_2, petroleum ether/ethyl acetate = 15:1 to 3:1), yielding **15** as light brown solid (33.4 mg, 72% yield). In a nuclear magnetic tube, 33.4 mg of pure product was dissolved in 0.5 mL of deuterated chloroform and tested by 400M NMR.

^{1}H NMR (400 MHz, CDCl$_3$) δ = 7.96 – 7.89 (m, 2H), 7.83 – 7.77 (m, 2H), 7.63 – 7.59 (m, 3H), 7.57 – 7.48 (m, 5H), 7.43 – 7.35 (m, 3H), 4.93 (d, *J* = 6.8 Hz, 1H), 1.50 (s, 3H), 1.42 (s, 3H). **^{13}C NMR (101 MHz, CDCl$_3$)** δ = 165.3, 147.2, 140.6, 134.2, 131.7, 130.8, 130.6, 130.4, 129.6, 129.4, 129.3, 128.6, 127.6, 121.9, 68.9, 54.8, 25.5, 21.4. HRMS (ESI-TOF) m/z: [M + H]$^+$ Calcd for $C_{24}H_{22}ClN_3O_3S$ 468.1143; Found 468.1142.

Product 16 (unknown compound)

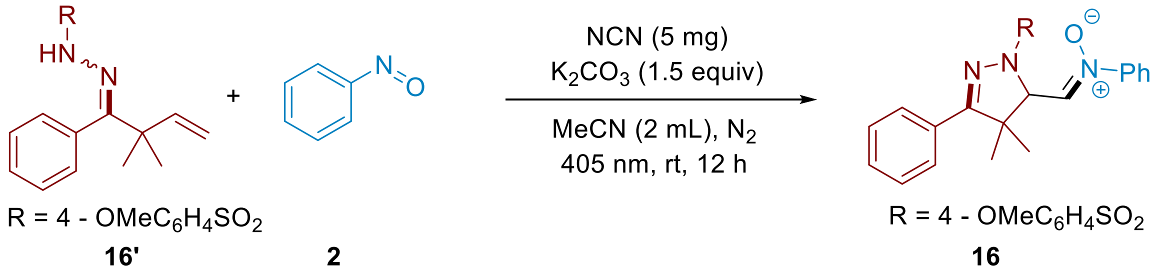

To an over dried bottle equipped with a magnetic stir bar, **16'** (35.8 mg, 0.1 mmol), **2** (32.1 mg, 0.3 mmol),

K_2CO_3 (20.7 mg, 0.15 mmol), NCN (5 mg) were added. After that, MeCN (2 mL) was added to the bottle in glovebox. The mixture was stirred under 405 nm LED irradiation for 12 h at room temperature. The product **16** was purified by column chromatography (SiO_2, petroleum ether/ethyl acetate = 15:1 to 3:1), yielding **16** as light brown solid (40.3 mg, 87% yield). In a nuclear magnetic tube, 40.3 mg of pure product was dissolved in 0.5 mL of deuterated chloroform and tested by 400M NMR.

^{1}H NMR (400 MHz, $CDCl_3$) δ = 7.95 – 7.88 (m, 2H), 7.84 – 7.77 (m, 2H), 7.62 (d, J = 6.8 Hz, 3H), 7.52 – 7.49 (m, 3H), 7.43 – 7.34 (m, 3H), 7.05 – 6.99 (m, 2H), 4.91 (d, J = 6.7 Hz, 1H), 3.86 (s, 3H), 1.49 (s, 3H), 1.42 (s, 3H). **^{13}C NMR (101 MHz, $CDCl_3$)** δ = 164.8, 163.9, 147.2, 134.8, 131.4, 130.7, 130.2, 129.9, 129.2, 128.5, 127.6, 124.7, 121.9, 114.2, 69.1, 55.6, 54.8, 25.6, 21.5. HRMS (ESI-TOF) m/z: $[M + H]^+$ Calcd for $C_{25}H_{25}N_3O_4S$ 464.1639; Found 464.1638.

Product 17 (unknown compound)

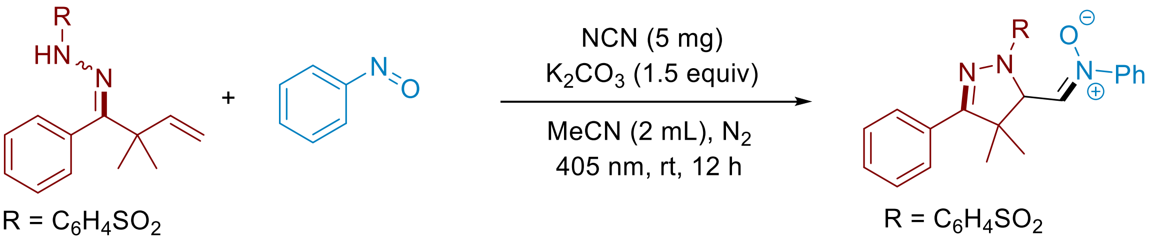

17' **2** **17**

To an over dried bottle equipped with a magnetic stir bar, **17'** (32.8 mg, 0.1 mmol), **2** (32.1 mg, 0.3 mmol), K_2CO_3 (20.7 mg, 0.15 mmol), NCN (5 mg) were added. After that, MeCN (2 mL) was added to the bottle in glovebox. The mixture was stirred under 405 nm LED irradiation for 12 h at room temperature. The product **17** was purified by column chromatography (SiO_2, petroleum ether/ethyl acetate = 15:1 to 3:1), yielding **17** as light brown solid (29.5 mg, 68% yield). In a nuclear magnetic tube, 29.5 mg of pure product was dissolved in 0.5 mL of deuterated chloroform and tested by 400M NMR.

^{1}H NMR (400 MHz, $CDCl_3$) δ = 8.06 – 7.93 (m, 2H), 7.84 – 7.77 (m, 2H), 7.67 – 7.55 (m, 6H), 7.54 – 7.45 (m, 3H), 7.43 – 7.34 (m, 3H), 4.93 (d, J = 6.6 Hz, 1H), 1.44 (d, J = 14.2 Hz, 6H). **^{13}C NMR (101 MHz, $CDCl_3$)** δ = 165.0, 147.2, 134.7, 133.9, 133.2, 130.7, 130.3, 129.8, 129.2, 129.2, 129.0, 128.5, 127.6, 121.9, 69.1, 54.9, 25.7, 21.4. HRMS (ESI-TOF) m/z: $[M + H]^+$ Calcd for $C_{24}H_{23}N_3O_3S$ 434.1533; Found 434.1533.

Product 18 (unknown compound)

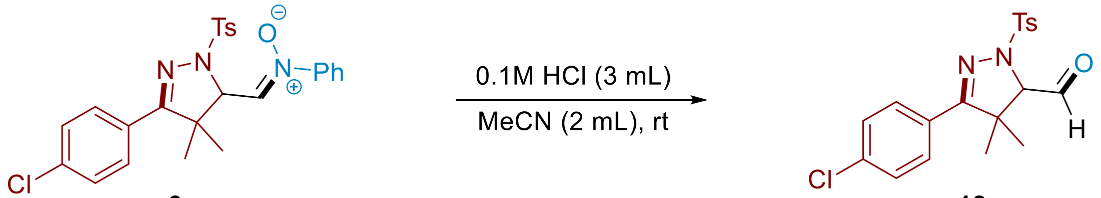

3 18

To an over dried bottle equipped with a magnetic stir bar, **3** (47.3 mg, 0.098 mmol), MeCN (2 mL) were added. After that, the mixture was stirred under air for 12 h at room temperature. The product **18** was purified by column chromatography (SiO_2, petroleum ether/ethyl acetate = 30:1 to 10:1), yielding **18** as white solid (31.2 mg, 81% yield). In a nuclear magnetic tube, 31.2 mg of pure product was dissolved in 0.5 mL of deuterated chloroform and tested by 400M NMR.

^{1}H NMR (400 MHz, CDCl$_3$) δ = 9.93 (d, J = 3.8 Hz, 1H), 7.77 (d, J = 8.4 Hz, 2H), 7.54 (d, J = 8.7 Hz, 2H), 7.36 – 7.33 (m, 4H), 3.41 (d, J = 3.8 Hz, 1H), 2.43 (s, 3H), 1.38 (s, 3H), 1.31 (s, 3H). **^{13}C NMR (101 MHz, CDCl$_3$)** δ = 197.8, 162.7, 145.3, 136.7, 130.4, 129.8, 129.0, 129.0, 128.8, 127.8, 77.4, 53.7, 24.8, 21.7, 20.2. HRMS (ESI-TOF) m/z: $[M + H]^+$ Calcd for $C_{19}H_{19}ClN_2O_3S$ 391.0878; Found 391.0876.

Product 19 (unknown compound)

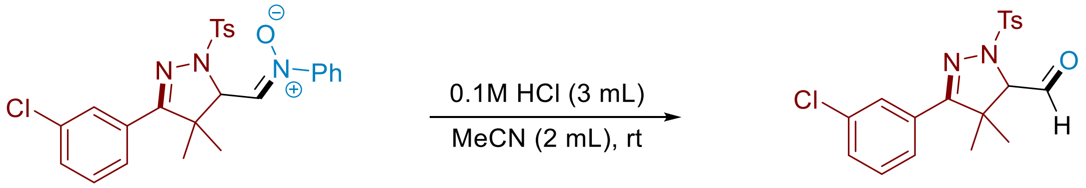

5 19

To an over dried bottle equipped with a magnetic stir bar, **5** (34.5 mg, 0.071 mmol), MeCN (2 mL) were added. After that, the mixture was stirred under air for 12 h at room temperature. The product **19** was purified by column chromatography (SiO_2, petroleum ether/ethyl acetate = 30:1 to 10:1), yielding **19** as white solid (21.9 mg, 75% yield). In a nuclear magnetic tube, 21.9 mg of pure product was dissolved in 0.5 mL of deuterated chloroform and tested by 400M NMR.

^{1}H NMR (400 MHz, CDCl$_3$) δ = 9.93 (d, J = 3.8 Hz, 1H), 7.78 (d, J = 8.3 Hz, 2H), 7.57 (t, J = 1.9 Hz, 1H), 7.48 – 7.45 (m, 1H), 7.42 – 7.27 (m, 4H), 3.43 (d, J = 3.8 Hz, 1H), 2.44 (s, 3H), 1.38 (s, 3H), 1.32 (s, 3H). **^{13}C NMR (101 MHz, CDCl$_3$)** δ = 197.8, 162.5, 145.4, 134.7, 131.1, 130.5, 130.4, 129.9, 129.8, 129.0, 127.6, 125.5, 77.4, 53.8, 24.7, 21.7, 20.2. HRMS (ESI-TOF) m/z: $[M + H]^+$ Calcd for $C_{19}H_{19}ClN_2O_3S$ 391.0878; Found 391.0876.

Product 20 (unknown compound)

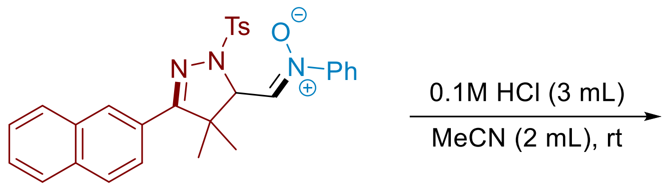

To an over dried bottle equipped with a magnetic stir bar, **11** (42.7 mg, 0.086 mmol), MeCN (2 mL) were added. After that, the mixture was stirred under air for 12 h at room temperature. The product **20** was purified by column chromatography (SiO_2, petroleum ether/ethyl acetate = 30:1 to 10:1), yielding **20** as white solid (25.4 mg, 77% yield). In a nuclear magnetic tube, 25.4 mg of pure product was dissolved in 0.5 mL of deuterated chloroform and tested by 400M NMR.

^{1}H NMR (400 MHz, $CDCl_3$) δ = 9.98 (d, *J* = 3.9 Hz, 1H), 8.01 (d, *J* = 1.6 Hz, 1H), 7.88 – 7.74 (m, 6H), 7.59 – 7.48 (m, 2H), 7.36 (d, *J* = 8.1 Hz, 2H), 3.46 (d, *J* = 3.9 Hz, 1H), 2.42 (s, 3H), 1.49 (s, 3H), 1.44 (s, 3H). **^{13}C NMR (101 MHz, $CDCl_3$)** δ = 198.1, 163.6, 145.2, 134.0, 132.6, 130.4, 129.7, 129.1, 128.5, 128.5, 127.7, 127.6, 127.3, 126.8, 126.8, 124.7, 77.5, 54.0, 25.1, 21.7, 20.5. HRMS (ESI-TOF) m/z: $[M + H]^+$ Calcd for $C_{23}H_{22}N_2O_3S$ 407.1424; Found 407.1417.

Product 21 (unknown compound)

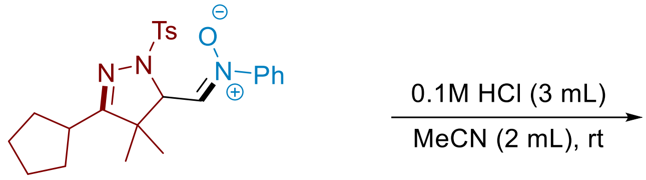

To an over dried bottle equipped with a magnetic stir bar, **13** (31.7 mg, 0.072 mmol), MeCN (2 mL) were added. After that, the mixture was stirred under air for 12 h at room temperature. The product **21** was purified by column chromatography (SiO_2, petroleum ether/ethyl acetate = 30:1 to 10:1), yielding **21** as white solid (22.7 mg, 76% yield). In a nuclear magnetic tube, 22.7 mg of pure product was dissolved in 0.5 mL of deuterated chloroform and tested by 400M NMR.

^{1}H NMR (400 MHz, $CDCl_3$) δ = 9.84 (d, *J* = 3.7 Hz, 1H), 7.71 (d, *J* = 8.3 Hz, 2H), 7.32 (d, *J* = 8.0 Hz, 2H), 3.18 (d, *J* = 3.7 Hz, 1H), 2.44 (s, 4H), 1.91 – 1.64 (m, 5H), 1.57 – 1.53 (m, 2H), 1.48 – 1.35 (m, 1H), 1.12 (s, 3H), 1.03 (s, 3H). **^{13}C NMR (101 MHz, $CDCl_3$)** δ = 198.8, 172.2, 144.8, 130.1, 129.3, 129.2, 77.0, 76.5, 54.4, 36.8, 32.5 (d, *J* = 13.1 Hz), 25.4 (d, *J* = 40.4 Hz), 24.2, 21.7, 19.7. HRMS (ESI-TOF) m/z: $[M + H]^+$ Calcd for $C_{18}H_{24}N_2O_3S$ 349.1580; Found 349.1580.

Product 23 (known compound CAS: 1973493-24-1)[1]

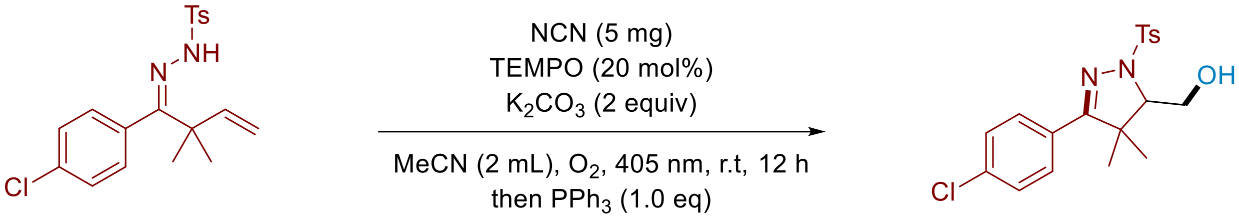

To an over dried bottle equipped with a magnetic stir bar, **3’** (75.2 mg, 0.2 mmol), TEMPO (6.25 mg, 0.04 mmol), K_2CO_3 (55.3 mg, 0.4 mmol), NCN (5 mg) were added. After that, MeCN (2 mL) was added to the bottle under a balloon of O_2. The mixture was stirred under 405 nm LED irradiation for 12 h at room temperature. The product **23** was purified by column chromatography (SiO_2, petroleum ether/ethyl acetate = 15:1 to 3:1), yielding **23** as light yellow solid (65.7 mg, 84% yield). In a nuclear magnetic tube, 65.7 mg of pure product was dissolved in 0.5 mL of deuterated chloroform and tested by 400M NMR.

^{1}H NMR (400 MHz, CDCl$_3$) δ = 7.80 (d, J = 8.3 Hz, 2H), 7.56 – 7.51 (m, 2H), 7.35 – 7.31 (m, 4H), 4.15 – 4.11 (m, 1H), 3.91 (d, J = 12.9 Hz, 1H), 3.46 (s, 1H), 3.21 – 3.19 (m, 1H), 2.42 (s, 3H), 1.26 (s, 3H), 1.14 (s, 3H). **^{13}C NMR (101 MHz, CDCl$_3$)** δ = 163.6, 144.9, 136.2, 130.6, 129.6, 128.8, 128.8, 128.7, 128.6, 74.6, 60.7, 51.0, 25.1, 21.6, 19.1.

Product 24 (unknown compound)

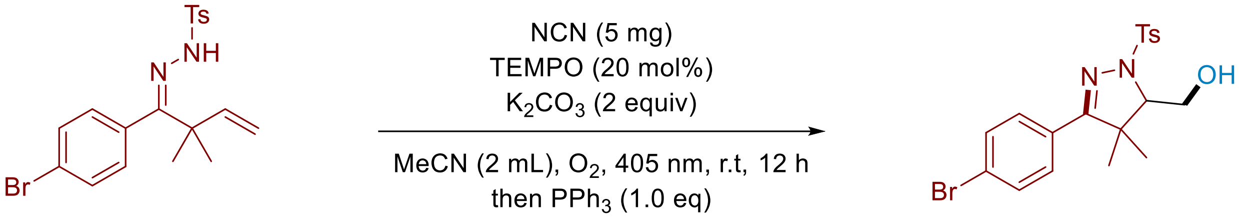

To an over dried bottle equipped with a magnetic stir bar, **6’** (84 mg, 0.2 mmol), TEMPO (6.25 mg, 0.04 mmol), K_2CO_3 (55.3 mg, 0.4 mmol), NCN (5 mg) were added. After that, MeCN (2 mL) was added to the bottle under a balloon of O_2. The mixture was stirred under 405 nm LED irradiation for 12 h at room temperature. The product **24** was purified by column chromatography (SiO_2, petroleum ether/ethyl acetate = 15:1 to 3:1), yielding **24** as light yellow solid (72.7 mg, 83% yield). In a nuclear magnetic tube, 72.7 mg of pure product was dissolved in 0.5 mL of deuterated chloroform and tested by 400M NMR.

^{1}H NMR (400 MHz, CDCl$_3$) δ = 7.79 (d, J = 8.4 Hz, 2H), 7.52 – 7.41 (m, 4H), 7.33 (d, J = 8.1 Hz, 2H), 4.13 (dd, J = 12.7, 6.0 Hz, 1H), 3.90 (d, J = 12.7 Hz, 1H), 3.46 (s, 1H), 3.20 (dd, J = 6.0, 2.8 Hz, 1H), 2.42

(s, 3H), 1.25 (s, 3H), 1.13 (s, 3H).**^{13}C NMR (101 MHz, CDCl$_3$)** δ = 163.7, 144.9, 131.7, 130.6, 129.6, 129.1, 129.0, 128.8, 124.6, 74.6, 60.7, 51.0, 25.1, 21.6, 19.1. HRMS (ESI-TOF) m/z: $[M + H]^+$ Calcd for $C_{19}H_{21}BrN_2O_3S$ 437.0529; Found 437.0524.

Product 25 (unknown compound)

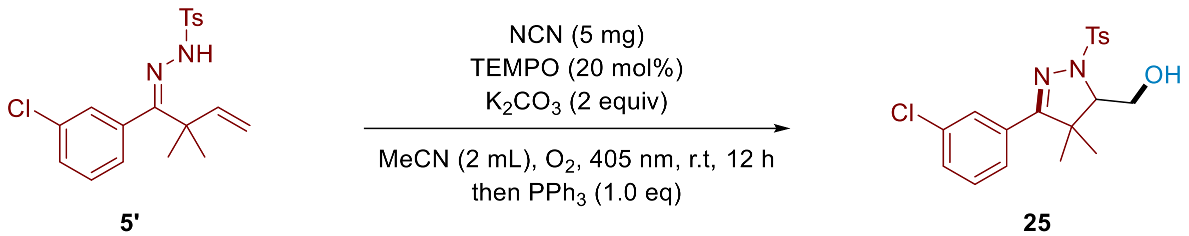

To an over dried bottle equipped with a magnetic stir bar, **5'** (75.2 mg, 0.2 mmol), TEMPO (6.25 mg, 0.04 mmol), K_2CO_3 (55.3 mg, 0.4 mmol), NCN (5 mg) were added. After that, MeCN (2 mL) was added to the bottle under a balloon of O_2. The mixture was stirred under 405 nm LED irradiation for 12 h at room temperature. The product **25** was purified by column chromatography (SiO_2, petroleum ether/ethyl acetate = 15:1 to 3:1), yielding **25** as light yellow solid (55 mg, 75% yield). In a nuclear magnetic tube, 55 mg of pure product was dissolved in 0.5 mL of deuterated chloroform and tested by 400M NMR.

^{1}H NMR (400 MHz, CDCl$_3$) δ = 7.81 (d, J = 8.4 Hz, 2H), 7.56 (t, J = 1.9 Hz, 1H), 7.47 – 7.44 (m, 1H), 7.39 – 7.32 (m, 3H), 7.29 (t, J = 7.9 Hz, 1H), 4.15 – 4.11 (m, 1H), 3.98 – 3.84 (m, 1H), 3.45 (s, 1H), 3.22 – 3.20 (m, 1H), 2.43 (s, 3H), 1.26 (s, 3H), 1.14 (s, 3H). **^{13}C NMR (101 MHz, CDCl$_3$)** δ = 163.5, 144.9, 134.5, 132.0, 130.6, 130.1, 129.7, 129.6, 128.8, 127.6, 125.5, 74.6, 60.7, 51.0, 25.1, 21.6, 19.1. HRMS (ESI-TOF) m/z: $[M + K]^+$ Calcd for $C_{19}H_{21}ClN_2O_3S$ 431.0593; Found 431.0590.

Product 26 (unknown compound)

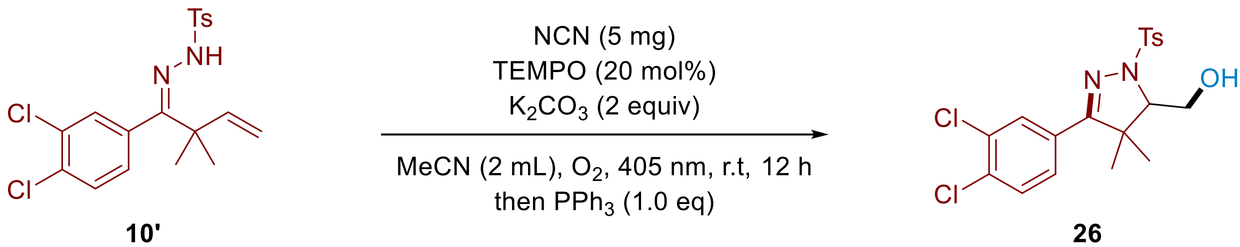

To an over dried bottle equipped with a magnetic stir bar, **10'** (82 mg, 0.2 mmol), TEMPO (6.25 mg, 0.04 mmol), K_2CO_3 (55.3 mg, 0.4 mmol), NCN (5 mg) were added. After that, MeCN (2 mL) was added to the bottle under a balloon of O_2. The mixture was stirred under 405 nm LED irradiation for 12 h at room temperature. The product **26** was purified by column chromatography (SiO_2, petroleum ether/ethyl acetate

= 15:1 to 3:1), yielding **26** as light yellow solid (55 mg, 64% yield). In a nuclear magnetic tube, 55 mg of pure product was dissolved in 0.5 mL of deuterated chloroform and tested by 400M NMR.

^{1}H NMR (400 MHz, $CDCl_3$) δ = 7.80 (d, *J* = 8.3 Hz, 2H), 7.68 (s, 1H), 7.43 (s, 2H), 7.35 (d, *J* = 8.1 Hz, 2H), 4.15 – 4.10 (m, 1H), 3.95 – 3.88 (m, 1H), 3.40 – 3.37 (m, 1H), 3.23 – 3.21 (m, 1H), 2.43 (s, 3H), 1.26 (s, 3H), 1.15 (s, 3H). **^{13}C NMR (101 MHz, $CDCl_3$)** δ = 162.5, 145.0, 134.4, 132.9, 130.6, 130.5, 130.1, 129.7, 129.3, 128.8, 126.6, 74.7, 60.6, 50.9, 25.2, 21.7, 19.1. HRMS (ESI-TOF) m/z: $[M + H]^+$ Calcd for $C_{19}H_{20}Cl_2N_2O_3S$ 427.0644; Found 427.0641.

Product 27 (known compound CAS: 1973493-23-0)[1]

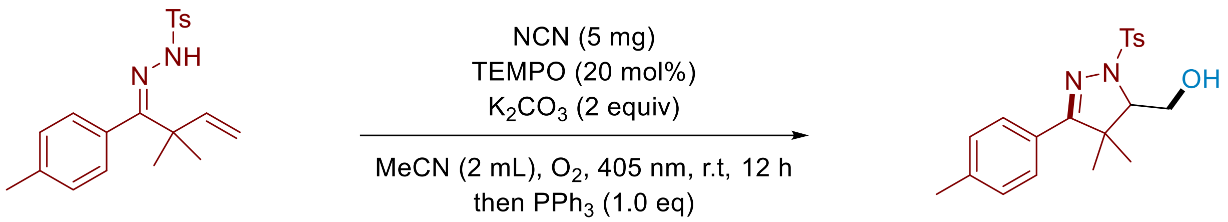

To an over dried bottle equipped with a magnetic stir bar, **7'** (35.6 mg, 0.1 mmol), **TEMPO** (3.1 mg, 0.02 mmol), **K_2CO_3** (27.6 mg, 0.2 mmol), **NCN** (5 mg) were added. After that, MeCN (2 mL) was added to the bottle under a balloon of O_2. The mixture was stirred under 405 nm LED irradiation for 12 h at room temperature. The product **27** was purified by column chromatography (SiO_2, petroleum ether/ethyl acetate = 15:1 to 3:1), yielding **27** as light yellow solid (26.5 mg, 71% yield). In a nuclear magnetic tube, 26.5 mg of pure product was dissolved in 0.5 mL of deuterated chloroform and tested by 400M NMR.

^{1}H NMR (400 MHz, $CDCl_3$) δ = 7.82 (d, *J* = 8.3 Hz, 2H), 7.50 (d, *J* = 8.3 Hz, 2H), 7.33 (d, *J* = 8.0 Hz, 2H), 7.16 (d, *J* = 8.0 Hz, 2H), 4.13 (dd, *J* = 12.7, 6.2 Hz, 1H), 3.94 – 3.85 (m, 1H), 3.52 – 3.50 (m, 1H), 3.17 (dd, *J* = 6.3, 2.5 Hz, 1H), 2.42 (s, 3H), 2.36 (s, 3H), 1.26 (s, 3H), 1.14 (s, 3H). **^{13}C NMR (101 MHz, $CDCl_3$)** δ = 164.6, 144.7, 140.4, 130.7, 129.5, 129.2, 129.0, 127.5, 127.4, 74.7, 60.8, 51.1, 25.1, 21.6, 21.3, 19.2.

Product 28 (unknown compound)

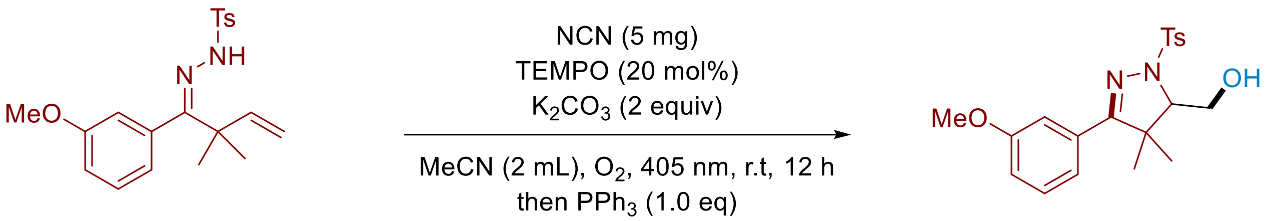

8' **28**

To an over dried bottle equipped with a magnetic stir bar, **8'** (74.4 mg, 0.2 mmol), TEMPO (6.25 mg, 0.04 mmol), K_2CO_3 (55.3 mg, 0.4 mmol), NCN (5 mg) were added. After that, MeCN (2 mL) was added to the bottle under a balloon of O_2. The mixture was stirred under 405 nm LED irradiation for 12 h at room temperature. The product **28** was purified by column chromatography (SiO_2, petroleum ether/ethyl acetate = 15:1 to 3:1), yielding **28** as light yellow solid (49.4 mg, 63% yield). In a nuclear magnetic tube, 49.4 mg of pure product was dissolved in 0.5 mL of deuterated chloroform and tested by 400M NMR.

^{1}H NMR (400 MHz, CDCl$_3$) δ = 7.83 (d, J = 8.3 Hz, 2H), 7.35 (d, J = 8.1 Hz, 2H), 7.31 – 7.26 (m, 1H), 7.18 – 7.10 (m, 2H), 6.96 – 6.93 (m, 1H), 4.17 – 4.12 (m, 1H), 3.93 (s, 1H), 3.83 (s, 3H), 3.53 (s, 1H), 3.21 – 3.19 (m, 1H), 2.43 (s, 3H), 1.27 (s, 3H), 1.15 (s, 3H). **^{13}C NMR (101 MHz, CDCl$_3$)** δ = 164.7, 159.4, 144.8, 131.4, 130.6, 129.5, 129.4, 128.9, 119.8, 115.5, 113.3, 74.6, 60.8, 55.3, 51.1, 25.1, 21.6, 19.2. HRMS (ESI-TOF) m/z: $[M + H]^+$ Calcd for $C_{20}H_{24}N_2O_4S$ 389.1530; Found 389.1526.

Product 29 (known compound CAS: 1973493-26-3)[1]

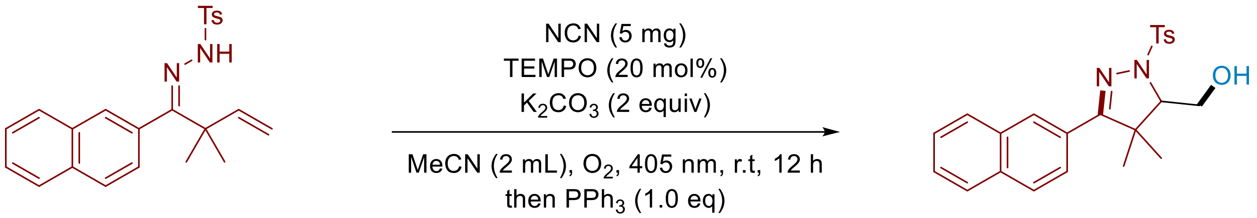

11' **29**

To an over dried bottle equipped with a magnetic stir bar, **11'** (78.4 mg, 0.2 mmol), TEMPO (6.25 mg, 0.04 mmol), K_2CO_3 (55.3 mg, 0.4 mmol), NCN (5 mg) were added. After that, MeCN (2 mL) was added to the bottle under a balloon of O_2. The mixture was stirred under 405 nm LED irradiation for 12 h at room temperature. The product **29** was purified by column chromatography (SiO_2, petroleum ether/ethyl acetate = 15:1 to 3:1), yielding **29** as light yellow solid (55.5 mg, 68% yield). In a nuclear magnetic tube, 55.5 mg of pure product was dissolved in 0.5 mL of deuterated chloroform and tested by 400M NMR.

^{1}H NMR (400 MHz, CDCl$_3$) δ = 8.03 (d, J = 1.7 Hz, 1H), 7.89 – 7.76 (m, 6H), 7.55 – 7.47 (m, 2H), 7.35

(d, J = 8.1 Hz, 2H), 4.20 – 4.16 (m, 1H), 4.01 – 3.90 (m, 1H), 3.53 (s, 1H), 3.27 – 3.25 (m, 1H), 2.42 (s, 3H), 1.37 (s, 3H), 1.27 (s, 3H). **^{13}C NMR (101 MHz, $CDCl_3$)** δ = 164.4, 144.8, 133.8, 132.6, 130.6, 129.6, 129.0, 128.5, 128.3, 127.7, 127.6, 127.3, 127.2, 126.6, 124.8, 74.8, 60.8, 51.2, 25.3, 21.6, 19.4.

Product 30 (unknown compound)

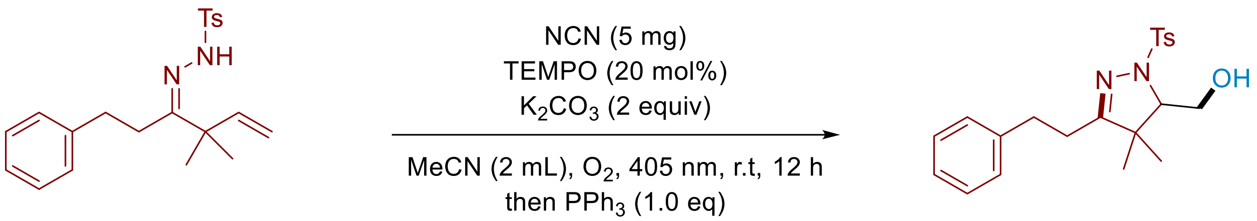

To an over dried bottle equipped with a magnetic stir bar, **12'** (74.03 mg, 0.2 mmol), TEMPO (6.25 mg, 0.04 mmol), K_2CO_3 (55.3 mg, 0.4 mmol), NCN (5 mg) were added. After that, MeCN (2 mL) was added to the bottle under a balloon of O_2. The mixture was stirred under 405 nm LED irradiation for 12 h at room temperature. The product **30** was purified by column chromatography (SiO_2, petroleum ether/ethyl acetate = 15:1 to 3:1), yielding **30** as light yellow solid (54 mg, 70% yield). In a nuclear magnetic tube, 54 mg of pure product was dissolved in 0.5 mL of deuterated chloroform and tested by 400M NMR.

^{1}H NMR (400 MHz, $CDCl_3$) δ = 7.75 (d, J = 8.3 Hz, 2H), 7.32 (d, J = 8.0 Hz, 2H), 7.28 – 7.17 (m, 3H), 7.11 – 7.06 (m, 2H), 4.04 – 4.01 (m, 1H), 3.84 – 3.79 (m, 1H), 3.43 – 3.40 (m, 1H), 3.08 – 3.05 (m, 1H), 3.02 – 2.94 (m, 1H), 2.83 – 2.76 (m, 1H), 2.50 – 2.41 (m, 4H), 2.37 – 2.29 (m, 1H), 0.99 (s, 3H), 0.77 (s, 3H). **^{13}C NMR (101 MHz, $CDCl_3$)** δ = 168.5, 144.6, 140.9, 130.7, 129.4, 129.0, 128.4, 128.3, 126.2, 72.7, 61.2, 51.4, 32.2, 27.9, 24.2, 21.6, 18.3. HRMS (ESI-TOF) m/z: $[M + H]^+$ Calcd for $C_{18}H_{24}N_2O_3S$ 349.1580; Found 349.1580. HRMS (ESI-TOF) m/z: $[M + K]^+$ Calcd for $C_{21}H_{26}N_2O_3S$ 425.1296; Found 425.1295.

Product 31 (unknown compound)

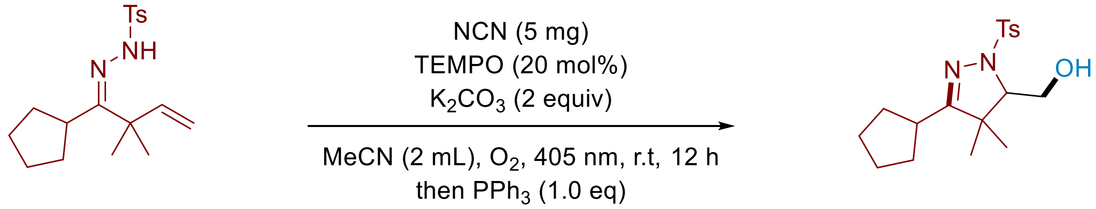

To an over dried bottle equipped with a magnetic stir bar, **13'** (66.8 mg, 0.2 mmol), TEMPO (6.25 mg, 0.04 mmol), K_2CO_3 (55.3 mg, 0.4 mmol), NCN (5 mg) were added. After that, MeCN (2 mL) was added to the bottle under a balloon of O_2. The mixture was stirred under 405 nm LED irradiation for 12 h at room

temperature. The product **31** was purified by column chromatography (SiO_2, petroleum ether/ethyl acetate = 15:1 to 3:1), yielding **31** as light yellow solid (27.8 mg, 40% yield). In a nuclear magnetic tube, 27.8 mg of pure product was dissolved in 0.5 mL of deuterated chloroform and tested by 400M NMR.

^{1}H NMR (400 MHz, $CDCl_3$) δ = 7.73 (d, *J* = 8.3 Hz, 2H), 7.30 (d, *J* = 8.1 Hz, 2H), 4.02 – 3.98 (m, 1H), 3.82 – 3.78 (m, 1H), 3.39 (s, 1H), 3.01 – 2.99 (m, 1H), 2.43 (s, 4H), 1.87 – 1.63 (m, 6H), 1.55 – 1.46 (m, 2H), 1.02 (s, 3H), 0.82 (s, 3H). **^{13}C NMR (101 MHz, $CDCl_3$)** δ = 173.2, 144.5, 130.3, 129.2, 129.1, 73.0, 61.3, 51.7, 37.1, 32.5, 32.5, 25.6, 25.3, 24.9, 21.6, 18.7. HRMS (ESI-TOF) m/z: $[M + H]^+$ Calcd for $C_{18}H_{26}N_2O_3S$ 351.1737; Found 351.1736.

Product 32 (known compound CAS: 1973493-29-6)[1]

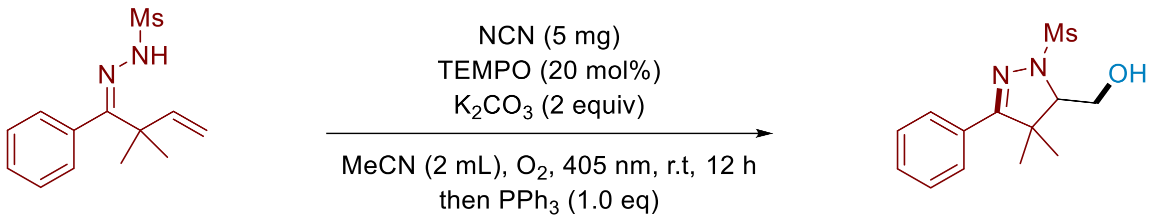

14' **32**

To an over dried bottle equipped with a magnetic stir bar, **32'** (53.2 mg, 0.2 mmol), TEMPO (6.25 mg, 0.04 mmol), K_2CO_3 (55.3 mg, 0.4 mmol), NCN (5 mg) were added. After that, MeCN (2 mL) was added to the bottle under a balloon of O_2. The mixture was stirred under 405 nm LED irradiation for 12 h at room temperature. The product **32** was purified by column chromatography (SiO_2, petroleum ether/ethyl acetate = 15:1 to 3:1), yielding **32** as light yellow solid (30 mg, 53% yield). In a nuclear magnetic tube, 30 mg of pure product was dissolved in 0.5 mL of deuterated chloroform and tested by 400M NMR.

^{1}H NMR (400 MHz, $CDCl_3$) δ = 7.73 – 7.67 (m, 2H), 7.45 – 7.38 (m, 3H), 4.06 (dd, *J* = 12.8, 5.9 Hz, 1H), 3.92 (dd, *J* = 12.8, 2.7 Hz, 1H), 3.72 (dd, *J* = 5.9, 2.7 Hz, 1H), 3.20 (s, 1H), 3.17 (s, 3H), 1.49 (s, 3H), 1.30 (s, 3H). **^{13}C NMR (101 MHz, $CDCl_3$)** δ = 165.2, 130.3, 130.1, 128.6, 127.7, 74.0, 60.6, 51.5, 36.3, 25.1, 19.0.

Product 33 (known compound CAS: 1973493-10-5)[1]

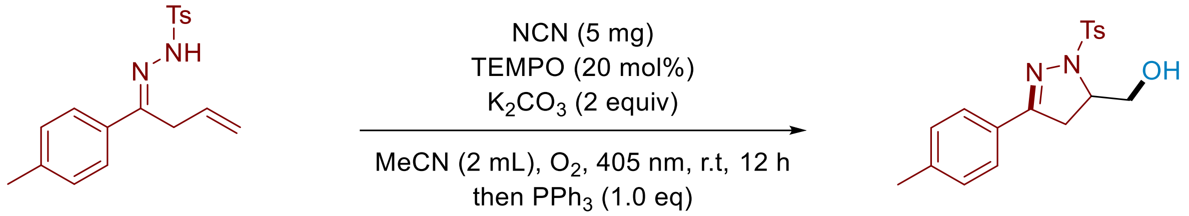

33' **33**

To an over dried bottle equipped with a magnetic stir bar, **33'** (65.6 mg, 0.2 mmol), TEMPO (6.25 mg, 0.04 mmol), K_2CO_3 (55.3 mg, 0.4 mmol), NCN (5 mg) were added. After that, MeCN (2 mL) was added to the bottle under a balloon of O_2. The mixture was stirred under 405 nm LED irradiation for 12 h at room temperature. The product **33** was purified by column chromatography (SiO_2, petroleum ether/ethyl acetate = 15:1 to 3:1), yielding **33** as light yellow solid (54.5 mg, 79% yield). In a nuclear magnetic tube, 54.5 mg of pure product was dissolved in 0.5 mL of deuterated chloroform and tested by 400M NMR.

^{1}H NMR (400 MHz, $CDCl_3$) δ = 7.71 (d, J = 8.3 Hz, 2H), 7.48 (d, J = 8.3 Hz, 2H), 7.20 (d, J = 8.1 Hz, 2H), 7.11 (d, J = 8.0 Hz, 2H), 4.02 – 3.98 (m, 1H), 3.93 – 3.76 (m, 2H), 3.11 – 2.93 (m, 2H), 2.30 (d, J = 6.3 Hz, 6H). **^{13}C NMR (101 MHz, $CDCl_3$)** δ = 158.6, 144.5, 141.2, 131.4, 129.6, 129.3, 128.6, 127.7, 126.9, 64.3, 63.5, 36.5, 21.6, 21.4.

Product 34 (unknown compound)

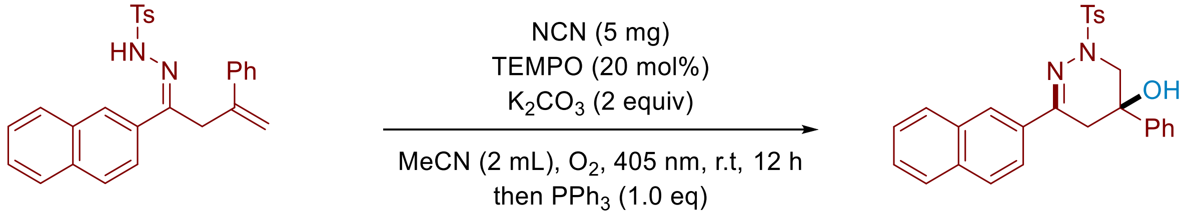

34' **34**

To an over dried bottle equipped with a magnetic stir bar, **34'** (42.6 mg, 0.1 mmol), TEMPO (6.25 mg, 0.02 mmol), K_2CO_3 (27.6 mg, 0.2 mmol), NCN (5 mg) were added. After that, MeCN (2 mL) was added to the bottle under a balloon of O_2. The mixture was stirred under 405 nm LED irradiation for 12 h at room temperature. The product **34** was purified by column chromatography (SiO_2, petroleum ether/ethyl acetate = 15:1 to 3:1), yielding **34** as light yellow solid (32.8 mg, 72% yield). In a nuclear magnetic tube, 32.8 mg of pure product was dissolved in 0.5 mL of deuterated chloroform and tested by 400M NMR.

^{1}H NMR (400 MHz, $CDCl_3$) δ = 8.11 – 8.08 (m, 1H), 7.96 (d, J = 1.9 Hz, 1H), 7.91 – 7.77 (m, 5H), 7.54 – 7.40 (m, 6H), 7.39 – 7.30 (m, 3H), 4.10 – 4.02 (m, 1H), 3.12 – 3.00 (m, 3H), 2.88 (s, 1H), 2.41 (s, 3H).

^{13}C NMR (101 MHz, $CDCl_3$) δ = 150.1, 144.5, 142.7, 133.9, 133.6, 132.8, 129.7, 128.8, 128.5, 128.2, 128.1, 127.7, 127.0, 126.5, 125.4, 124.8, 122.9, 68.3, 53.1, 38.2, 21.6. HRMS (ESI-TOF) m/z: $[M + H]^+$ Calcd for $C_{27}H_{24}N_2O_3S$ 457.1580; Found 457.1579.

Product 35 (unknown compound)

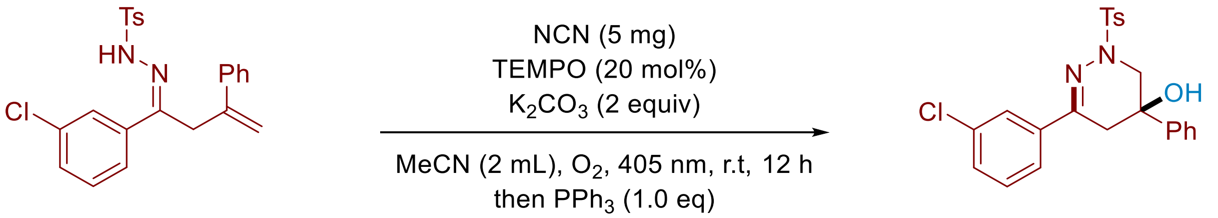

35' **35**

To an over dried bottle equipped with a magnetic stir bar, **35'** (84.8 mg, 0.2 mmol), TEMPO (6.25 mg, 0.04 mmol), K_2CO_3 (55.3 mg, 0.4 mmol), NCN (5 mg) were added. After that, MeCN (2 mL) was added to the bottle under a balloon of O_2. The mixture was stirred under 405 nm LED irradiation for 12 h at room temperature. The product **35** was purified by column chromatography (SiO_2, petroleum ether/ethyl acetate = 15:1 to 3:1), yielding **35** as light yellow solid (49.1 mg, 56% yield). In a nuclear magnetic tube, 49.1 mg of pure product was dissolved in 0.5 mL of deuterated chloroform and tested by 400M NMR.

^{1}H NMR (400 MHz, $CDCl_3$) δ = 7.84 (d, J = 8.2 Hz, 2H), 7.69 – 7.68 (m, 1H), 7.60 – 7.57 (m, 1H), 7.47 – 7.38 (m, 4H), 7.37 – 7.27 (m, 5H), 4.05 – 4.01 (m, 1H), 3.04 – 3.01 (m, 1H), 2.98 – 2.56 (m, 3H), 2.42 (s, 3H). **^{13}C NMR (101 MHz, $CDCl_3$)** δ = 148.9, 144.6, 142.5, 138.0, 134.6, 132.9, 129.7, 129.7, 129.7, 128.8, 128.4, 128.2, 125.7, 124.7, 123.6, 68.2, 52.9, 38.2, 21.6. HRMS (ESI-TOF) m/z: $[M + H]^+$ Calcd for $C_{23}H_{21}ClN_2O_3S$ 441.1034; Found 441.1032.

Product 36 (known compound CAS: 1569-94-4)[1]

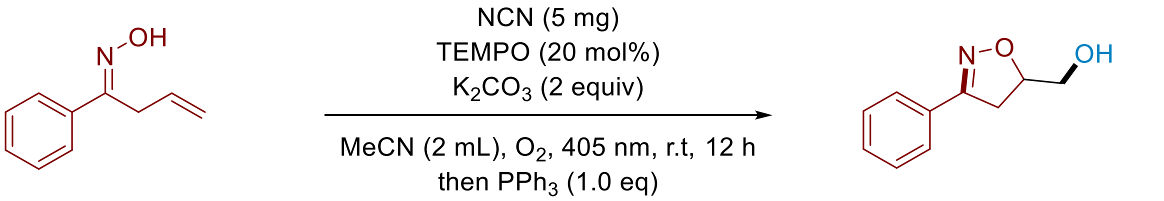

36' **36**

To an over dried bottle equipped with a magnetic stir bar, **36'** (46.9 mg, 0.29 mmol), TEMPO (6.25 mg, 0.04 mmol), K_2CO_3 (55.3 mg, 0.4 mmol), NCN (5 mg) were added. After that, MeCN (2 mL) was added to the bottle under a balloon of O_2. The mixture was stirred under 405 nm LED irradiation for 12 h at room temperature. The product **36** was purified by column chromatography (SiO_2, petroleum ether/ethyl acetate = 15:1 to 3:1), yielding **36** as light yellow solid (29.9 mg, 56% yield). In a nuclear magnetic tube, 29.9 mg

of pure product was dissolved in 0.5 mL of deuterated chloroform and tested by 400M NMR.

^{1}H NMR (400 MHz, CDCl₃) δ = 7.77 – 7.56 (m, 2H), 7.43 – 7.36 (m, 3H), 4.90 – 4.83 (m, 1H), 3.92 – 3.63 (m, 2H), 3.44 – 3.22 (m, 2H), 2.31 (d, *J* = 6.4 Hz, 1H). **^{13}C NMR (101 MHz, CDCl₃)** δ = 157.1, 130.2, 129.2, 128.7, 126.7, 81.2, 63.6, 36.3.

Product 37 (known compound CAS: 206055-84-7)[2]

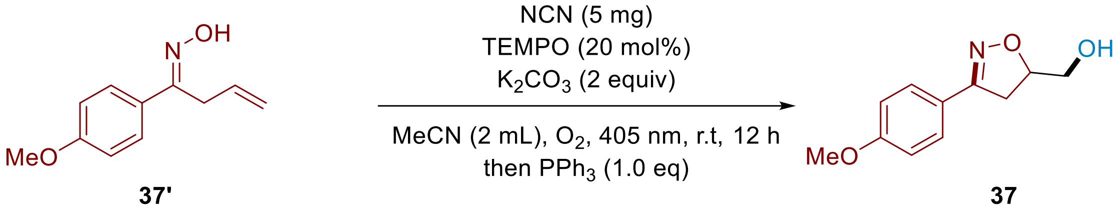

To an over dried bottle equipped with a magnetic stir bar, **37’** (36.2 mg, 0.2 mmol), TEMPO (6.25 mg, 0.04 mmol), K_2CO_3 (55.3 mg, 0.4 mmol), NCN (5 mg) were added. After that, MeCN (2 mL) was added to the bottle under a balloon of O_2. The mixture was stirred under 405 nm LED irradiation for 12 h at room temperature. The product **37** was purified by column chromatography (SiO_2, petroleum ether/ethyl acetate = 15:1 to 3:1), yielding **37** as light yellow solid (31.6 mg, 80% yield). In a nuclear magnetic tube, 31.6 mg of pure product was dissolved in 0.5 mL of deuterated chloroform and tested by 400M NMR.

^{1}H NMR (400 MHz, CDCl₃) δ = 7.70 – 7.52 (m, 2H), 7.01 – 6.82 (m, 2H), 4.87 – 4.81 (m, 1H), 3.84 (s, 4H), 3.72 – 3.62 (m, 1H), 3.41 – 3.20 (m, 2H), 2.03 (s, 1H). **^{13}C NMR (101 MHz, CDCl₃)** δ = 161.1, 156.6, 128.2, 121.8, 114.1, 80.9, 63.7, 55.3, 36.6.

Product 39 (known compound, CAS: 135-19-3)[3]

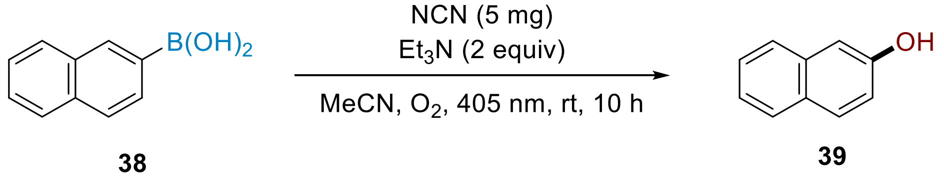

To an over dried bottle equipped with a magnetic stir bar, **39'** (34.4 mg, 0.2 mmol), NCN (5 mg), Et_3N (40.5 mg, 0.4 mmol) were added. After that, MeCN (2 mL) was added to the bottle connected with a O_2 balloon. The mixture was stirred under 405 nm LED irradiation for 10 h at room temperature. The product **39** was purified by column chromatography (SiO_2, petroleum ether/ethyl acetate = 20:1), yielding **39** as light brown solid (27.5 mg, 95%). In a nuclear magnetic tube, 27.5 mg of pure product was dissolved in 0.5 mL of deuterated chloroform and tested by 400M NMR.

^{1}H NMR (400 MHz, $CDCl_3$) δ = 7.80 – 7.76 (m, 2H), 7.70 – 7.68 (m, 1H), 7.47 – 7.43 (m, 1H), 7.37 – 7.33 (m, 1H), 7.19 – 7.09 (m, 2H), 5.37 (s, 1H). **^{13}C NMR (101 MHz, $CDCl_3$)** δ = 153.2, 134.5, 129.8, 128.9, 127.7, 126.5, 126.3, 123.6, 117.7, 109.5.

Product 40 (known compound, CAS: 98-54-4) [4]

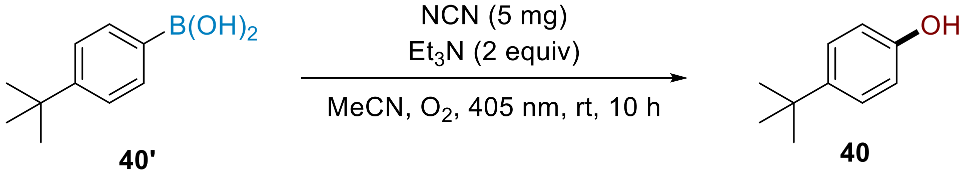

To an over dried bottle equipped with a magnetic stir bar, **40’** (57.4 mg, 0.2 mmol), NCN (5 mg), Et_3N (40.5 mg, 0.4 mmol) were added. After that, MeCN (2 mL) was added to the bottle connected with a O_2 balloon. The mixture was stirred under 405 nm LED irradiation for 10 h at room temperature. The product **40** was purified by column chromatography (SiO_2, petroleum ether/ethyl acetate = 20:1), yielding **40** as white solid (15.2 mg, 52%). In a nuclear magnetic tube, 15.2 mg of pure product was dissolved in 0.5 mL of deuterated chloroform and tested by 400M NMR.

^{1}H NMR (400 MHz, $CDCl_3$) δ = 7.30 – 7.20 (m, 2H), 6.79 – 6.75 (m, 2H), 4.62 (s, 1H), 1.29 (s, 9H). **^{13}C NMR (101 MHz, $CDCl_3$)** δ = 153.1, 143.5, 126.4, 114.7, 34.1, 31.5.

Product 41 (known compound, CAS: 1638-22-8)[5]

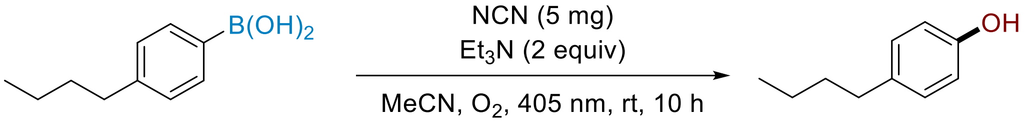

To an over dried bottle equipped with a magnetic stir bar, **41’** (35.6 mg, 0.2 mmol), NCN (5 mg), Et_3N (40.5 mg, 0.4 mmol) were added. After that, MeCN (2 mL) was added to the bottle connected with a O_2 balloon. The mixture was stirred under 405 nm LED irradiation for 10 h at room temperature. The product **41** was purified by column chromatography (SiO_2, petroleum ether/ethyl acetate = 20:1), yielding **41** as colourless oil (15.7 mg, 52%). In a nuclear magnetic tube, 15.7 mg of pure product was dissolved in 0.5 mL of deuterated chloroform and tested by 400M NMR.

^{1}H NMR (400 MHz, $CDCl_3$) δ = 7.05 (d, J = 8.5 Hz, 2H), 6.75 (d, J = 8.4 Hz, 2H), 4.68 (s, 1H), 2.59 – 2.49 (m, 2H), 1.61 – 1.50 (m, 2H), 1.37 – 1.30 (m, 2H), 0.92 (t, J = 7.3 Hz, 3H). **^{13}C NMR (101 MHz, $CDCl_3$)** δ = 153.3, 135.2, 129.4, 115.0, 34.7, 33.9, 22.3, 14.0.

Product 42 (known compound, CAS: 150-76-5)[4]

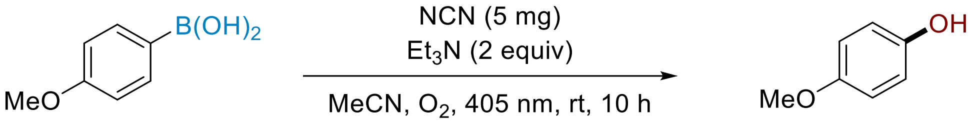

42' **42**

To an over dried bottle equipped with a magnetic stir bar, **42'** (30.4 mg, 0.2 mmol), NCN (5 mg), Et_3N (40.5 mg, 0.4 mmol) were added. After that, MeCN (2 mL) was added to the bottle connected with a O_2 balloon. The mixture was stirred under 405 nm LED irradiation for 10 h at room temperature. The product **42** was purified by column chromatography (SiO_2, petroleum ether/ethyl acetate = 20:1), yielding **42** as brown liqiud (17.6 mg, 71%). In a nuclear magnetic tube, 17.6 mg of pure product was dissolved in 0.5 mL of deuterated chloroform and tested by 400M NMR.

^{1}H NMR (400 MHz, $CDCl_3$) δ = 7.00 – 6.49 (m, 4H), 5.13 (s, 1H), 3.77 (s, 3H). **^{13}C NMR (101 MHz, $CDCl_3$)** δ = 153.6, 149.4, 116.0, 114.9, 55.8.

Product 43 (known compound, CAS: 106-48-9)[6]

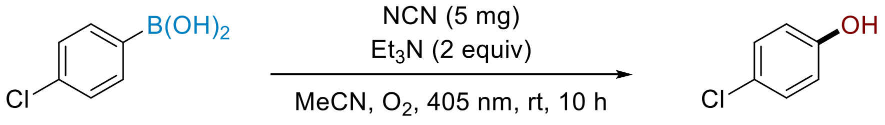

43' **43**

To an over dried bottle equipped with a magnetic stir bar, **43'** (31.3 mg, 0.2 mmol), NCN (5 mg), Et_3N (40.5 mg, 0.4 mmol) were added. After that, MeCN (2 mL) was added to the bottle connected with a O_2 balloon. The mixture was stirred under 405 nm LED irradiation for 10 h at room temperature. The product **43** was purified by column chromatography (SiO_2, petroleum ether/ethyl acetate = 20:1), yielding **43** as light yellow solid (18.9 mg, 73%). In a nuclear magnetic tube, 18.9 mg of pure product was dissolved in 0.5 mL of deuterated chloroform and tested by 400M NMR.

^{1}H NMR (400 MHz, $CDCl_3$) δ = 7.22 – 7.16 (m, 2H), 6.85 – 6.69 (m, 2H), 4.92 (s, 1H). **^{13}C NMR (101 MHz, CDCl3)** δ = 154.0, 129.5, 125.7, 116.6.

Product 44 (known compound, CAS: 106-41-2)[4]

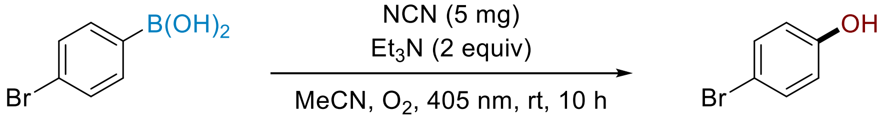

44' **44**

To an over dried bottle equipped with a magnetic stir bar, **44’** (40.2 mg, 0.2 mmol), NCN (5 mg), Et_3N (40.5 mg, 0.4 mmol) were added. After that, MeCN (2 mL) was added to the bottle connected with a O_2 balloon. The mixture was stirred under 405 nm LED irradiation for 10 h at room temperature. The product **44** was purified by column chromatography (SiO_2, petroleum ether/ethyl acetate = 20:1), yielding **44** as light brown solid (30.6 mg, 89%). In a nuclear magnetic tube, 30.6 mg of pure product was dissolved in 0.5 mL of deuterated chloroform and tested by 400M NMR.

^{1}H NMR (400 MHz, $CDCl_3$) δ = 7.37 – 7.30 (m, 2H), 6.76 – 6.67 (m, 2H), 4.94 (s, 1H). **^{13}C NMR (101 MHz, $CDCl_3$)** δ = 154.6, 132.5, 117.2, 112.9.

Product 45 (known compound, CAS: 540-38-5)[4]

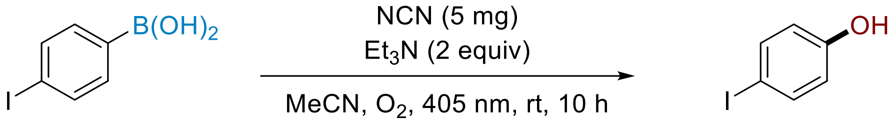

45' **45**

To an over dried bottle equipped with a magnetic stir bar, **45’** (49.6 mg, 0.2 mmol), NCN (5 mg), Et_3N (40.5 mg, 0.4 mmol) were added. After that, MeCN (2 mL) was added to the bottle connected with a O_2 balloon. The mixture was stirred under 405 nm LED irradiation for 10 h at room temperature. The product **45** was purified by column chromatography (SiO_2, petroleum ether/ethyl acetate = 20:1), yielding **45** as light brown solid (47.3 mg, 99%). In a nuclear magnetic tube, 47.3 mg of pure product was dissolved in 0.5 mL of deuterated chloroform and tested by 400M NMR.

^{1}H NMR (400 MHz, $CDCl_3$) δ = 7.63 – 7.35 (m, 2H), 6.67 – 6.55 (m, 2H), 5.02 (s, 1H). **^{13}C NMR (101 MHz, $CDCl_3$)** δ = 155.2, 138.4, 117.8, 82.7.

Product 46 (known compound, CAS: 120-47-8) [7]

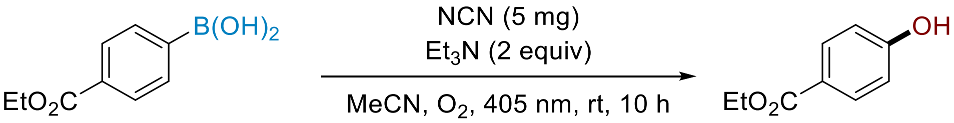

46' **46**

To an over dried bottle equipped with a magnetic stir bar, **46’** (38.8 mg, 0.2 mmol), NCN (5 mg), Et_3N (40.5 mg, 0.4 mmol) were added. After that, MeCN (2 mL) was added to the bottle connected with a O_2 balloon. The mixture was stirred under 405 nm LED irradiation for 10 h at room temperature. The product **46** was purified by column chromatography (SiO_2, petroleum ether/ethyl acetate = 20:1), yielding **46** as

white solid (31.2 mg, 94%). In a nuclear magnetic tube, 31.2 mg of pure product was dissolved in 0.5 mL of deuterated chloroform and tested by 400M NMR.

^{1}H NMR (400 MHz, CDCl$_3$) δ = 8.03 – 7.88 (m, 2H), 6.92 – 6.83 (m, 2H), 6.53 (s, 1H), 4.36 (q, J = 7.1 Hz, 2H), 1.38 (t, J = 7.1 Hz, 3H). **^{13}C NMR (101 MHz, CDCl$_3$)** δ = 167.2, 167.1, 167.1, 160.4, 160.4, 160.3, 131.9, 122.4, 122.3, 115.2, 115.2, 61.0, 61.0, 14.3.

Product 47 (known compound, CAS: 554-84-7)[3]

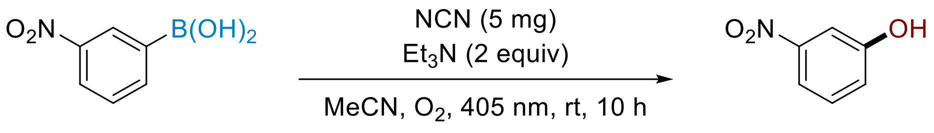

47' **47**

To an over dried bottle equipped with a magnetic stir bar, **47'** (33.4 mg, 0.2 mmol), NCN (5 mg), Et_3N (40.5 mg, 0.4 mmol) were added. After that, MeCN (2 mL) was added to the bottle connected with a O_2 balloon. The mixture was stirred under 405 nm LED irradiation for 10 h at room temperature. The product **47** was purified by column chromatography (SiO_2, petroleum ether/ethyl acetate = 20:1), yielding **47** as light yellow solid (18.3 mg, 66%). In a nuclear magnetic tube, 18.3 mg of pure product was dissolved in 0.5 mL of deuterated chloroform and tested by 400M NMR.

^{1}H NMR (400 MHz, CDCl$_3$) δ = 7.84 – 7.77 (m, 1H), 7.71 – 7.70 (m, 1H), 7.40 (t, J = 8.2 Hz, 1H), 7.20 – 7.17 (m, 1H). **^{13}C NMR (101 MHz, CDCl$_3$)** δ = 156.3, 149.1, 130.3, 122.0, 115.8, 110.5.

Product 48 (known compound, CAS: 3769-41-3)[8]

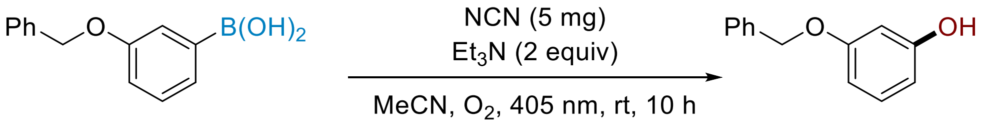

48' **48**

To an over dried bottle equipped with a magnetic stir bar, **48'** (45.6 mg, 0.2 mmol), NCN (5 mg), Et_3N (40.5 mg, 0.4 mmol) were added. After that, MeCN (2 mL) was added to the bottle connected with a O_2 balloon. The mixture was stirred under 405 nm LED irradiation for 10 h at room temperature. The product **48** was purified by column chromatography (SiO_2, petroleum ether/ethyl acetate = 20:1), yielding **48** as white solid (31.5 mg, 79%). In a nuclear magnetic tube, 31.5 mg of pure product was dissolved in 0.5 mL of deuterated chloroform and tested by 400M NMR.

^{1}H NMR (400 MHz, CDCl$_3$) δ = 7.46 – 7.35 (m, 4H), 7.35 – 7.28 (m, 1H), 7.13 (t, J = *8.2 Hz*, 1H), 6.57

(m, 1H), 6.48 (t, J = *2.3 Hz*, 1H), 6.43 (m, 1H), 5.02 (s, 2H). **^{13}C NMR (101 MHz, $CDCl_3$)** δ = 160.1, 156.6, 136.8, 130.2, 128.6, 128.0, 127.5, 108.0, 107.3, 102.4, 70.0.

Product 49 (known compound, CAS: 533-31-3)[5]

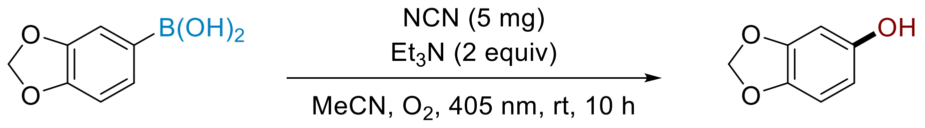

49' **49**

To an over dried bottle equipped with a magnetic stir bar, **49'** (23.2 mg, 0.14 mmol), NCN (5 mg), Et_3N (40.5 mg, 0.4 mmol) were added. After that, MeCN (2 mL) was added to the bottle connected with a O_2 balloon. The mixture was stirred under 405 nm LED irradiation for 10 h at room temperature. The product **49** was purified by column chromatography (SiO_2, petroleum ether/ethyl acetate = 20:1), yielding **49** as white solid (17.4 mg, 90%). In a nuclear magnetic tube, 17.4 mg of pure product was dissolved in 0.5 mL of deuterated chloroform and tested by 400M NMR.

^{1}H NMR (400 MHz, $CDCl_3$) δ = 6.65 (d, J = 8.3 Hz, 1H), 6.43 (d, J = 2.6 Hz, 1H), 6.25 (dd, J = 8.3, 2.5 Hz, 1H), 5.91 (s, 2H), 5.04 – 5.03 (m, 1H). **^{13}C NMR (101 MHz, $CDCl_3$)** δ = 150.5, 148.2, 141.5, 108.1, 106.6, 101.1, 98.3.

Product 50 (known compound, CAS: 13523-92-7) [9]

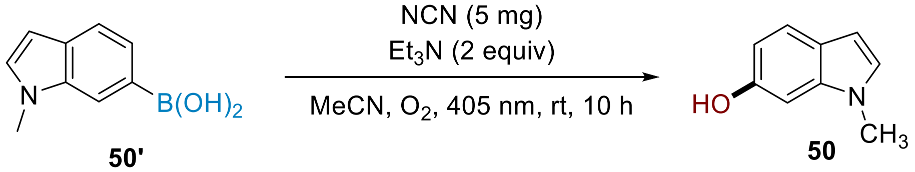

50' **50**

To an over dried bottle equipped with a magnetic stir bar, **50'** (35 mg, 0.2 mmol), NCN (5 mg), Et_3N (40.5 mg, 0.4 mmol) were added. After that, MeCN (2 mL) was added to the bottle connected with a O_2 balloon. The mixture was stirred under 405 nm LED irradiation for 10 h at room temperature. The product **50** was purified by column chromatography (SiO_2, petroleum ether/ethyl acetate = 20:1), yielding **50** as white solid (24.2 mg, 82%). In a nuclear magnetic tube, 24.2 mg of pure product was dissolved in 0.5 mL of deuterated chloroform and tested by 400M NMR.

^{1}H NMR (400 MHz, $CDCl_3$) δ = 7.18 (d, J = 8.7 Hz, 1H), 7.03 (t, J = 3.2 Hz, 2H), 6.82 – 6.79 (m, 1H), 6.36 – 6.35 (m, 1H), 4.60 (s, 1H), 3.76 (s, 3H). **^{13}C NMR (101 MHz, $CDCl_3$)** δ = 149.3, 132.3, 129.7, 129.0, 111.3, 109.8, 105.1, 100.0, 33.0.

Product 51 (known compound, CAS: 876472-35-4) [10]

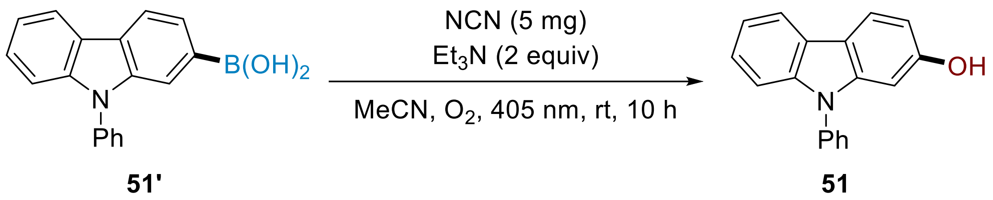

To an over dried bottle equipped with a magnetic stir bar, **50’** (57.4 mg, 0.2 mmol), NCN (5 mg), Et_3N (40.5 mg, 0.4 mmol) were added. After that, MeCN (2 mL) was added to the bottle connected with a O_2 balloon. The mixture was stirred under 405 nm LED irradiation for 10 h at room temperature. The product **51** was purified by column chromatography (SiO_2, petroleum ether/ethyl acetate = 20:1), yielding **51** as white solid (34.6 mg, 67%). In a nuclear magnetic tube, 34.6 mg of pure product was dissolved in 0.5 mL of deuterated chloroform and tested by 400M NMR.

^{1}H NMR (400 MHz, $CDCl_3$) δ = 8.02 (d, J = 7.6 Hz, 1H), 7.96 (d, J = 8.3 Hz, 1H), 7.59 (dd, J = 8.2, 7.1 Hz, 2H), 7.55 – 7.52 (m, 2H), 7.48 – 7.43 (m, 1H), 7.35 – 7.30 (m, 2H), 7.27 – 7.22 (m, 2H), 6.84 – 6.77 (m, 2H), 4.95 (s, 1H). **^{13}C NMR (101 MHz, $CDCl_3$)** δ = 154.8, 142.3, 141.0, 137.5, 129.9, 127.5, 127.0, 124.7, 123.5, 121.2, 120.0, 119.4, 117.4, 109.5, 108.9, 96.0.

Product 53 (known compound, CAS: 13047-06-8) [11]

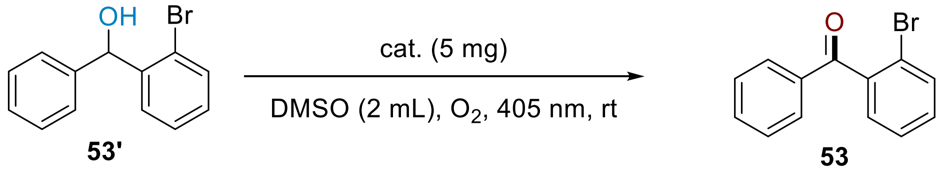

To an over dried bottle equipped with a magnetic stir bar, **53’** (42.6 mg, 0.2 mmol), NCN (5 mg), were added. After that, DMSO (2 mL) was added to the bottle connected with a O_2 balloon. The mixture was stirred under 405 nm LED irradiation for 11 h at room temperature. The product **53** was purified by column chromatography (SiO_2, petroleum ether/ethyl acetate = 20:1), yielding **53** as white solid (36.2 mg, 69%). In a nuclear magnetic tube, 36.2 mg of pure product was dissolved in 0.5 mL of deuterated chloroform and tested by 400M NMR.

^{1}H NMR (400 MHz, $CDCl_3$) δ = 7.85 – 7.77 (m, 2H), 7.67 – 7.58 (m, 2H), 7.50 – 7.32 (m, 5H). **^{13}C NMR (101 MHz, $CDCl_3$)** δ = 195.9, 140.6, 136.1, 133.7, 133.2, 131.1, 130.2, 129.0, 128.6, 127.2, 119.5.

Product 54 (known compound, CAS: 486-25-9) [11]

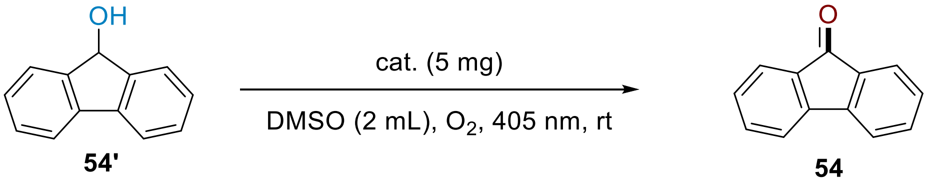

To an over dried bottle equipped with a magnetic stir bar, **54’** (36.4 mg, 0.2 mmol), NCN (5 mg), were added. After that, DMSO (2 mL) was added to the bottle connected with a O_2 balloon. The mixture was stirred under 405 nm LED irradiation for 11 h at room temperature. The product **54** was purified by column chromatography (SiO_2, petroleum ether/ethyl acetate = 20:1), yielding **54** as white solid (39 mg, 99%). In a nuclear magnetic tube, 39 mg of pure product was dissolved in 0.5 mL of deuterated chloroform and tested by 400M NMR.

^{1}H NMR (400 MHz, $CDCl_3$) δ = 7.67 – 7.65 (m, 2H), 7.54 – 7.45 (m, 4H), 7.31 – 7.26 (m, 2H). **^{13}C NMR (101 MHz, $CDCl_3$)** δ = 193.9, 144.4, 134.7, 134.1, 129.1, 124.3, 120.3.

Product 55 (known compound, CAS: 119-61-9) [11]

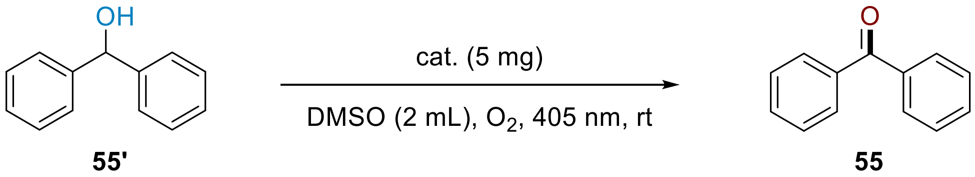

To an over dried bottle equipped with a magnetic stir bar, **55’** (36.8 mg, 0.2 mmol), NCN (5 mg), were added. After that, DMSO (2 mL) was added to the bottle connected with a O_2 balloon. The mixture was stirred under 405 nm LED irradiation for 12 h at room temperature. The product **55** was purified by column chromatography (SiO_2, petroleum ether/ethyl acetate = 20:1), yielding **55** as white solid (30.4 mg, 83%). In a nuclear magnetic tube, 30.4 mg of pure product was dissolved in 0.5 mL of deuterated chloroform and tested by 400M NMR.

^{1}H NMR (400 MHz, $CDCl_3$) δ = 7.87 – 7.72 (m, 4H), 7.62 – 7.55 (m, 2H), 7.50 – 7.46 (m, 4H). **^{13}C NMR (101 MHz, $CDCl_3$)** δ = 196.7, 137.5, 132.4, 130.0, 128.2.

Product 56 (known compound, CAS: 98-86-2) [11]

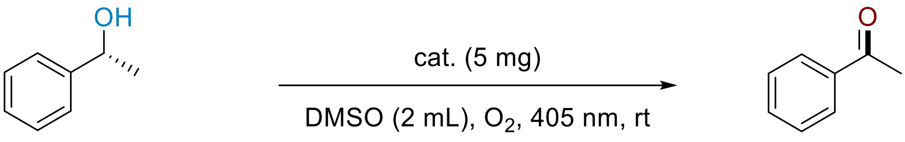

56' **56**

To an over dried bottle equipped with a magnetic stir bar, **56’** (36.8 mg, 0.2 mmol), NCN (5 mg), were added. After that, DMSO (2 mL) was added to the bottle connected with a O_2 balloon. The mixture was stirred under 405 nm LED irradiation for 5 h at room temperature. The product **56** was purified by column chromatography (SiO_2, petroleum ether/ethyl acetate = 20:1), yielding **56** as colourless oil (12.1 mg, 50%). In a nuclear magnetic tube, 12.1 mg of pure product was dissolved in 0.5 mL of deuterated chloroform and tested by 400M NMR.

^{1}H NMR (400 MHz, $CDCl_3$) δ = 7.96 – 7.79 (m, 2H), 7.54 – 7.43 (m, 1H), 7.43 – 7.29 (m, 2H), 2.52 – 2.51 (m, 3H). **^{13}C NMR (101 MHz, $CDCl_3$)** δ = 197.8, 136.8, 132.8, 128.3, 128.0, 26.3.

Product 57 (known compound, CAS: 104-87-0) [11]

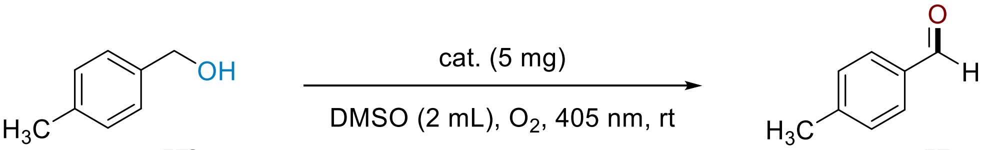

57' **57**

To an over dried bottle equipped with a magnetic stir bar, **57’** (24.4 mg, 0.2 mmol), NCN (5 mg), were added. After that, DMSO (2 mL) was added to the bottle connected with a O_2 balloon. The mixture was stirred under 405 nm LED irradiation for 5 h at room temperature. The product **9f** was purified by column chromatography (SiO_2, petroleum ether/ethyl acetate = 20:1), yielding **9f** as colourless oil (15.4 mg, 64%). In a nuclear magnetic tube, 15.4 mg of pure product was dissolved in 0.5 mL of deuterated chloroform and tested by 400M NMR.

^{1}H NMR (400 MHz, $CDCl_3$) δ = 9.96 (s, 1H), 7.81 – 7.75 (m, 2H), 7.33 (d, *J* = 7.8 Hz, 2H), 2.44 (s, 3H). **^{13}C NMR (101 MHz, $CDCl_3$)** δ = 192.0, 145.5, 134.2, 129.8, 129.7, 21.9.

Product 58 (known compound, CAS: 122-03-2)[12]

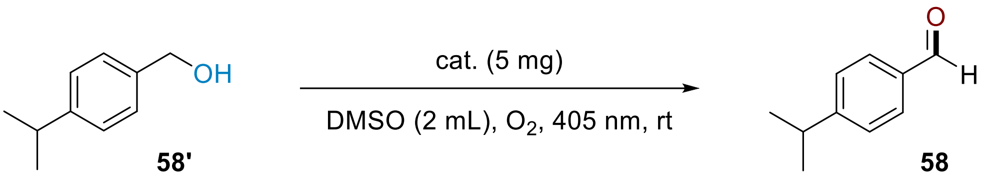

58' 58

To an over dried bottle equipped with a magnetic stir bar, **58'** (30 mg, 0.2 mmol), NCN (5 mg), were added. After that, DMSO (2 mL) was added to the bottle connected with a O_2 balloon. The mixture was stirred under 405 nm LED irradiation for 6 h at room temperature. The product **58** was purified by column chromatography (SiO_2, petroleum ether/ethyl acetate = 20:1), yielding **58** as colourless oil (18.4 mg, 62%). In a nuclear magnetic tube, 18.4 mg of pure product was dissolved in 0.5 mL of deuterated chloroform and tested by 400M NMR.

^{1}H NMR (400 MHz, $CDCl_3$) δ = 9.97 (s, 1H), 7.81 (d, *J* = 8.3 Hz, 2H), 7.38 (d, *J* = 8.3 Hz, 2H), 3.02 – 2.05 (m, 1H), 1.28 (d, *J* = 6.9 Hz, 6H). **^{13}C NMR (101 MHz, $CDCl_3$)** δ = 192.0, 156.2, 134.5, 130.0, 127.1, 34.4, 23.6.

Product 59 (known compound, CAS: 67-36-7) [13]

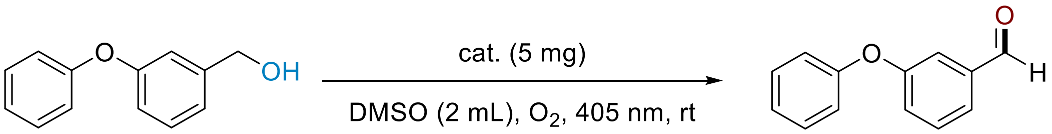

59' 59

To an over dried bottle equipped with a magnetic stir bar, **59'** (40 mg, 0.2 mmol), NCN (5 mg), were added. After that, DMSO (2 mL) was added to the bottle connected with a O_2 balloon. The mixture was stirred under 405 nm LED irradiation for 21 h at room temperature. The product **59** was purified by column chromatography (SiO_2, petroleum ether/ethyl acetate = 20:1), yielding **59** as colourless oil (34.2 mg, 86%). In a nuclear magnetic tube, 34.2 mg of pure product was dissolved in 0.5 mL of deuterated chloroform and tested by 400M NMR.

^{1}H NMR (400 MHz, $CDCl_3$) δ = 9.95 (s, 1H), 7.61 – 7.58 (m, 1H), 7.52 – 7.45 (m, 2H), 7.40 – 7.35 (m, 2H), 7.30 – 7.27 (m, 1H), 7.19 – 7.14 (m, 1H), 7.06 – 7.02 (m, 2H). **^{13}C NMR (101 MHz, $CDCl_3$)** δ = 191.6, 158.4, 156.1, 138.0, 130.4, 130.0, 124.7, 124.5, 124.1, 119.4, 118.1.

Product 60 (known compound, CAS: 120-57-0) [11]

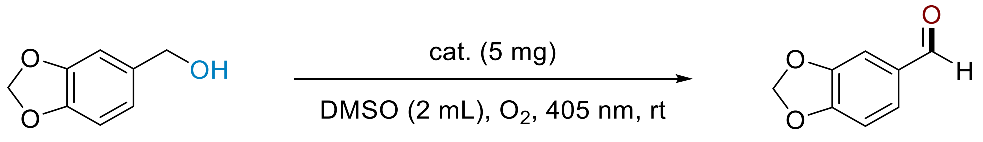

To an over dried bottle equipped with a magnetic stir bar, **60’** (31.6 mg, 0.2 mmol), NCN (5 mg), were added. After that, DMSO (2 mL) was added to the bottle connected with a O_2 balloon. The mixture was stirred under 405 nm LED irradiation for 8 h at room temperature. The product **60** was purified by column chromatography (SiO_2, petroleum ether/ethyl acetate = 20:1), yielding **60** as colourless oil (19.7 mg, 66%). In a nuclear magnetic tube, 19.7 mg of pure product was dissolved in 0.5 mL of deuterated chloroform and tested by 400M NMR.

^{1}H NMR (400 MHz, CDCl$_3$) δ = 9.80 (s, 1H), 7.42 – 7.39 (m, 1H), 7.32 (t, *J* = 1.4 Hz, 1H), 6.92 (dd, *J* = 8.0, 1.0 Hz, 1H), 6.07 (d, *J* = 1.1 Hz, 2H). **^{13}C NMR (101 MHz, CDCl$_3$)** δ = 190.3, 153.1, 148.7, 131.8, 128.6, 108.3, 106.9, 102.1.

Product 61 (known compound, CAS: 1122-91-4) [12]

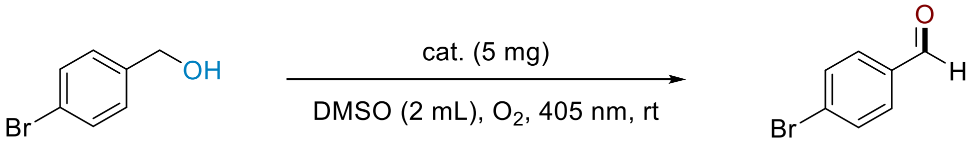

To an over dried bottle equipped with a magnetic stir bar, **61’** (37.4 mg, 0.2 mmol), NCN (5 mg), were added. After that, DMSO (2 mL) was added to the bottle connected with a O_2 balloon. The mixture was stirred under 405 nm LED irradiation for 6 h at room temperature. The product **61** was purified by column chromatography (SiO_2, petroleum ether/ethyl acetate = 20:1), yielding **61** as white solid (24.8 mg, 67%). In a nuclear magnetic tube, 24.8 mg of pure product was dissolved in 0.5 mL of deuterated chloroform and tested by 400M NMR.

^{1}H NMR (400 MHz, CDCl$_3$) δ = 9.97 (s, 1H), 7.75 (d, *J* = 8.0 Hz, 2H), 7.6.9 (d, *J* = 8.0 Hz, 2H). **^{13}C NMR (101 MHz, CDCl$_3$)** δ = 191.1, 135.0, 132.4, 130.9, 129.8.

Product 62 (known compound, CAS: 3218-36-8) [14]

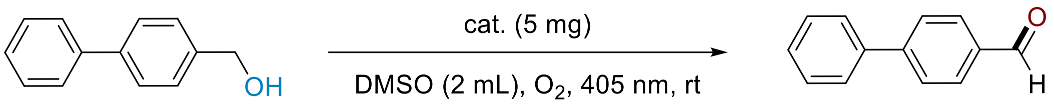

62' **62**

To an over dried bottle equipped with a magnetic stir bar, **62’** (36.8 mg, 0.2 mmol), NCN (5 mg), were added. After that, DMSO (2 mL) was added to the bottle connected with a O_2 balloon. The mixture was stirred under 405 nm LED irradiation for 17 h at room temperature. The product **62** was purified by column chromatography (SiO_2, petroleum ether/ethyl acetate = 20:1), yielding **62** as white solid (22.1 mg, 61%). In a nuclear magnetic tube, 22.1 mg of pure product was dissolved in 0.5 mL of deuterated chloroform and tested by 400M NMR.

^{1}H NMR (400 MHz, $CDCl_3$) δ = 10.06 (s, 1H), 7.96 (d, *J* = 8.3 Hz, 2H), 7.76 (d, *J* = 8.3 Hz, 2H), 7.66 – 7.63 (m, 2H), 7.51 – 7.47 (m, 2H), 7.45 – 7.40 (m, 1H). **^{13}C NMR (101 MHz, $CDCl_3$)** δ = 191.9, 147.2, 139.7, 135.2, 130.3, 129.0, 128.5, 127.7, 127.4.

Product 63 (known compound, CAS: 66-99-9) [11]

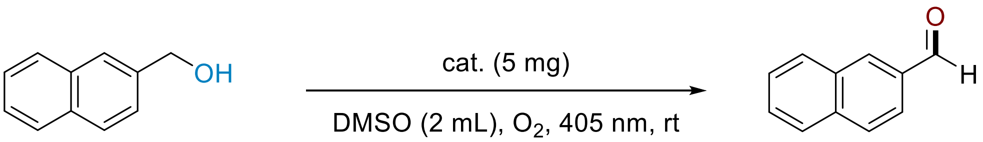

63' **63**

To an over dried bottle equipped with a magnetic stir bar, **63’** (31.6 mg, 0.2 mmol), NCN (5 mg), were added. After that, DMSO (2 mL) was added to the bottle connected with a O_2 balloon. The mixture was stirred under 405 nm LED irradiation for 18 h at room temperature. The product **63** was purified by column chromatography (SiO_2, petroleum ether/ethyl acetate = 20:1), yielding **63** as white solid (29 mg, 93%). In a nuclear magnetic tube, 29 mg of pure product was dissolved in 0.5 mL of deuterated chloroform and tested by 400M NMR.

^{1}H NMR (400 MHz, $CDCl_3$) δ = 10.16 (s, 1H), 8.34 (d, *J* = 1.5 Hz, 1H), 8.02 – 7.88 (m, 4H), 7.67 – 7.57 (m, 2H). **^{13}C NMR (101 MHz, $CDCl_3$)** δ = 192.3, 136.4, 134.5, 134.1, 132.6, 129.5, 129.1, 129.1, 128.0, 127.1, 122.7.

Product 64 (known compound, CAS: 99662-34-7)[15]

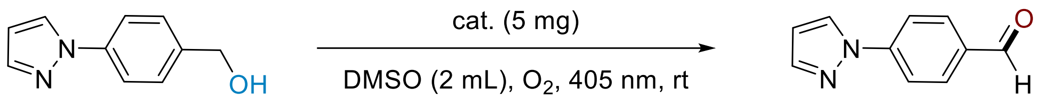

64' **64**

To an over dried bottle equipped with a magnetic stir bar, **64'** (34.4 mg, 0.2 mmol), NCN (5 mg), were added. After that, DMSO (2 mL) was added to the bottle connected with a O_2 balloon. The mixture was stirred under 405 nm LED irradiation for 17 h at room temperature. The product **64** was purified by column chromatography (SiO_2, petroleum ether/ethyl acetate = 20:1), yielding **64** as light yellow solid (24 mg, 70%). In a nuclear magnetic tube, 24 mg of pure product was dissolved in 0.5 mL of deuterated chloroform and tested by 400M NMR.

^{1}H NMR (400 MHz, CDCl$_3$) δ = 10.01 (s, 1H), 8.04 – 7.95 (m, 3H), 7.91 – 7.86 (m, 2H), 7.78 (d, *J* = 1.8 Hz, 1H), 6.53 (t, *J* = 2.2 Hz, 1H). **^{13}C NMR (101 MHz, CDCl$_3$)** δ = 190.9, 144.2, 142.3, 134.0, 131.3, 126.9, 118.8, 108.8.

Product 65 (known compound, CAS: 99-94-5) [16]

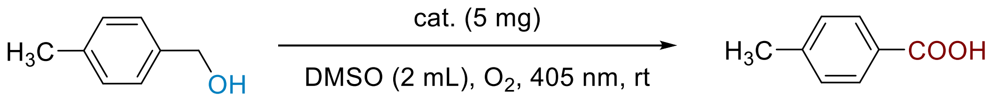

65' **65**

To an over dried bottle equipped with a magnetic stir bar, **65'** (24.4 mg, 0.2 mmol), NCN (5 mg), were added. After that, DMSO (2 mL) was added to the bottle connected with a O_2 balloon. The mixture was stirred under 405 nm LED irradiation for 48 h at room temperature. The product **65** was purified by column chromatography (SiO_2, petroleum ether/ethyl acetate = 10:1), yielding **65** as white solid (23.8 mg, 87%). In a nuclear magnetic tube, 23.8 mg of pure product was dissolved in 0.5 mL of deuterated chloroform and tested by 400M NMR.

^{1}H NMR (400 MHz, CDCl$_3$) δ = 8.01 (d, *J* = 8.3 Hz, 2H), 7.27 (d, *J* = 8.0 Hz, 2H), 2.43 (s, 3H). **^{13}C NMR (101 MHz, CDCl$_3$)** δ = 172.5, 144.6, 130.2, 129.2, 126.6, 21.7.

Product 66 (known compound, CAS: 536-66-3) [17]

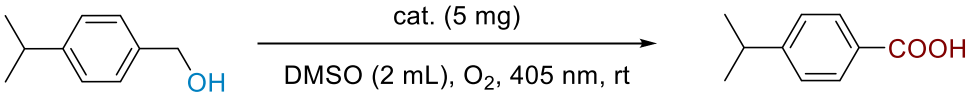

66' **66**

To an over dried bottle equipped with a magnetic stir bar, **66'** (30 mg, 0.2 mmol), NCN (5 mg), were added.

After that, DMSO (2 mL) was added to the bottle connected with a O_2 balloon. The mixture was stirred under 405 nm LED irradiation for 36 h at room temperature. The product **66** was purified by column chromatography (SiO_2, petroleum ether/ethyl acetate = 10:1), yielding **66** as white solid (18.6 mg, 57%). In a nuclear magnetic tube, 18.6 mg of pure product was dissolved in 0.5 mL of deuterated chloroform and tested by 400M NMR.

^{1}H NMR (400 MHz, $CDCl_3$) δ = 8.03 (d, J = 8.3 Hz, 2H), 7.32 (d, J = 8.2 Hz, 2H), 3.02 – 2.93 (m, 1H), 1.27 (d, J = 6.9 Hz, 6H). **^{13}C NMR (101 MHz, $CDCl_3$)** δ = 171.9, 155.3, 130.4, 126.9, 126.6, 34.3, 23.7.

Product 67 (known compound, CAS: 98-73-7) [17]

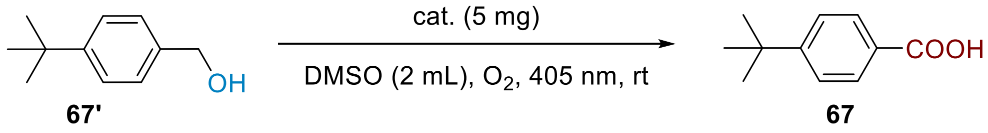

To an over dried bottle equipped with a magnetic stir bar, **67’** (32.8 mg, 0.2 mmol), NCN (5 mg), were added. After that, DMSO (2 mL) was added to the bottle connected with a O_2 balloon. The mixture was stirred under 405 nm LED irradiation for 36 h at room temperature. The product **67** was purified by column chromatography (SiO_2, petroleum ether/ethyl acetate = 10:1), yielding **67** as white solid (30.6 mg, 86%). In a nuclear magnetic tube, 30.6 mg of pure product was dissolved in 0.5 mL of deuterated chloroform and tested by 400M NMR.

^{1}H NMR (400 MHz, $CDCl_3$) δ = 8.09 – 8.02 (m, 2H), 7.52 – 7.47 (m, 2H), 1.36 (s, 9H). **^{13}C NMR (101 MHz, $CDCl_3$)** δ = 171.9, 157.6, 130.1, 126.5, 125.5, 35.2, 31.1.

Product 68 (known compound, CAS: 586-76-5) [17]

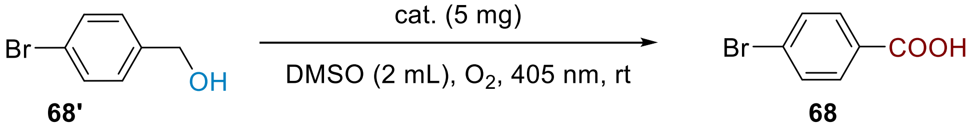

To an over dried bottle equipped with a magnetic stir bar, **68’** (37.4 mg, 0.2 mmol), NCN (5 mg), were added. After that, DMSO (2 mL) was added to the bottle connected with a O_2 balloon. The mixture was stirred under 405 nm LED irradiation for 10 h at room temperature. The product **68** was purified by column chromatography (SiO_2, petroleum ether/ethyl acetate = 10:1), yielding **68** as white solid (31.8 mg, 78%). In a nuclear magnetic tube, 31.8 mg of pure product was dissolved in 0.5 mL of deuterated chloroform and tested by 400M NMR.

¹H NMR (400 MHz, DMSO-d6) δ = 13.19 (s, 1H), 7.86 (d, *J* = 8.5 Hz, 2H), 7.70 (d, *J* = 8.5 Hz, 2H). **¹³C NMR (101 MHz, DMSO-d6)** δ = 166.6, 131.7, 131.3, 130.0, 126.9.

Product 69 (known compound, CAS: 62-23-7) [17]

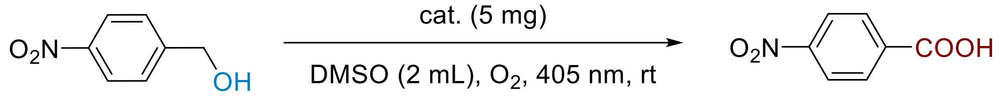

69' **69**

To an over dried bottle equipped with a magnetic stir bar, **69’** (30.6 mg, 0.2 mmol), NCN (5 mg), were added. After that, DMSO (2 mL) was added to the bottle connected with a O_2 balloon. The mixture was stirred under 405 nm LED irradiation for 36 h at room temperature. The product **69** was purified by column chromatography (SiO_2, petroleum ether/ethyl acetate = 10:1), yielding **69** as light yellow solid (27.1 mg, 81%). In a nuclear magnetic tube, 27.1 mg of pure product was dissolved in 0.5 mL of deuterated chloroform and tested by 400M NMR.

¹H NMR (400 MHz, DMSO-d6) δ = 13.64 (s, 1H), 8.29 (d, *J* = 8.8 Hz, 2H), 8.14 (d, *J* = 8.8 Hz, 2H). **¹³C NMR (101 MHz, DMSO-d6)** δ = 165.8, 150.0, 136.4, 130.7, 123.7.

Product 70 (known compound, CAS: 92-92-2) [17]

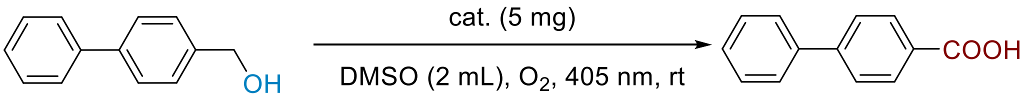

70' **70**

To an over dried bottle equipped with a magnetic stir bar, **70’** (36.8 mg, 0.2 mmol), NCN (5 mg), were added. After that, DMSO (2 mL) was added to the bottle connected with a O_2 balloon. The mixture was stirred under 405 nm LED irradiation for 56 h at room temperature. The product **70** was purified by column chromatography (SiO_2, petroleum ether/ethyl acetate = 10:1), yielding **70** as white solid (31.8 mg, 79%). In a nuclear magnetic tube, 31.8 mg of pure product was dissolved in 0.5 mL of deuterated chloroform and tested by 400M NMR.

¹H NMR (400 MHz, $CDCl_3$) δ = 8.19 (d, *J* = 8.5 Hz, 2H), 7.74 – 7.62 (m, 4H), 7.51 – 7.39 (m, 3H). **¹³C NMR (101 MHz, $CDCl_3$)** δ = 170.7, 146.5, 139.9, 130.7, 129.0, 128.3, 127.9, 127.3, 127.2.

Product 71 (known compound, CAS: 93-09-4) [17]

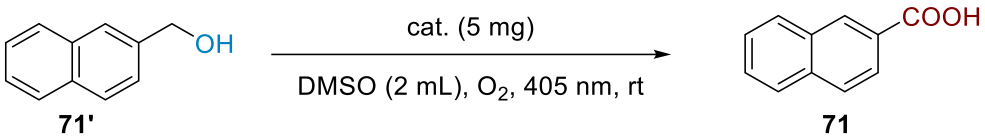

To an over dried bottle equipped with a magnetic stir bar, **71’** (31.6 mg, 0.2 mmol), NCN (5 mg), were added. After that, DMSO (2 mL) was added to the bottle connected with a O_2 balloon. The mixture was stirred under 405 nm LED irradiation for 48 h at room temperature. The product **71** was purified by column chromatography (SiO_2, petroleum ether/ethyl acetate = 10:1), yielding **71** as white solid (27.2 mg, 79%). In a nuclear magnetic tube, 27.2 mg of pure product was dissolved in 0.5 mL of deuterated chloroform and tested by 400M NMR.

^{1}H NMR (400 MHz, CDCl$_3$) δ = 8.74 – 8.73 (m, 1H), 8.14 (dd, *J* = 8.6, 1.7 Hz, 1H), 8.00 (d, *J* = 8.1 Hz, 1H), 7.95 – 7.90 (m, 2H), 7.65 – 7.56 (m, 2H). **^{13}C NMR (101 MHz, CDCl$_3$)** δ = 172.1, 136.0, 132.4, 132.2, 129.5, 128.7, 128.3, 127.8, 126.8, 126.5, 125.4.

Product 72 (known compound, CAS: 16209-00-0)

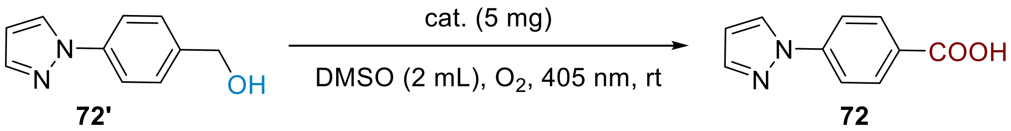

To an over dried bottle equipped with a magnetic stir bar, **72’** (34.4 mg, 0.2 mmol), NCN (5 mg), were added. After that, DMSO (2 mL) was added to the bottle connected with a O_2 balloon. The mixture was stirred under 405 nm LED irradiation for 56 h at room temperature. The product **72** was purified by column chromatography (SiO_2, petroleum ether/ethyl acetate = 10:1), yielding **72** as white solid (30.3 mg, 81%). In a nuclear magnetic tube, 30.3 mg of pure product was dissolved in 0.5 mL of deuterated chloroform and tested by 400M NMR.

^{1}H NMR (400 MHz, DMSO-*d*$_6$) δ = 13.01 (s, 1H), 8.62 (d, *J* = 2.6 Hz, 1H), 8.05 (d, *J* = 8.8 Hz, 2H), 7.98 (d, *J* = 8.8 Hz, 2H), 7.81 (d, *J* = 1.7 Hz, 1H), 6.60 (t, *J* = 2.2 Hz, 1H). **^{13}C NMR (101 MHz, DMSO)** δ = 166.7, 142.7, 141.9, 130.9, 128.2, 128.1, 117.9, 108.6.

Product 76 (known compound, CAS: 681470-56-4) [18]

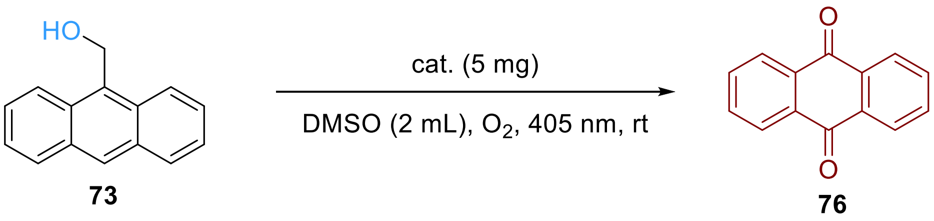

To an over dried bottle equipped with a magnetic stir bar, **73** (41.6 mg, 0.2 mmol), NCN (5 mg), were added. After that, DMSO (2 mL) was added to the bottle connected with a O_2 balloon. The mixture was stirred under 405 nm LED irradiation for 6 h at room temperature. The product **76** was purified by column chromatography (SiO_2, petroleum ether/ethyl acetate = 10:1), yielding **76** as light yellow solid (31.3 mg, 78%). In a nuclear magnetic tube, 31.3 mg of pure product was dissolved in 0.5 mL of deuterated chloroform and tested by 400M NMR.

^{1}H NMR (400 MHz, $CDCl_3$) δ = 8.39 – 8.22 (m, 4H), 7.84 – 7.78 (m, 4H). **^{13}C NMR (101 MHz, $CDCl_3$)** δ = 183.2, 134.1, 133.5, 127.2.

3.3 Procedure for Gram-Scale Reaction

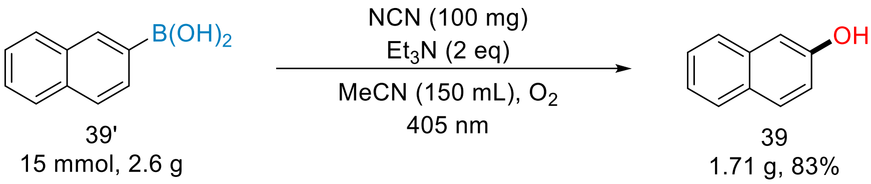

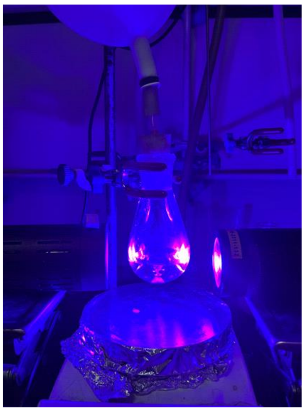

Figure S8 Gram-scale reaction

To an over dried bottle equipped with a magnetic stir bar, **39’** (2.6 g, 15 mmol), NCN (100 mg, 0.3% wt), Et_3N (3.0 g, 30 mmol) were added. After that, MeCN (150 mL) was added to the bottle connected with a O_2 balloon. The mixture was stirred under 405 nm LED irradiation for 12 h at room temperature. The product **39** was purified by column chromatography (SiO_2, petroleum ether/ethyl acetate = 20:1), yielding **39** as light brown solid (1.71 g, 83%).

4. X-Ray crystallographic data of compound 9

Single Crystal X-ray Diffraction

Single Crystal X-ray diffraction data were collected using a Bruker D8 Quest diffractometer (Mo Kα, λ= 0.71073 Å). Indexing and data integration were performed using APEX3 (Difference Vectors method). Absorption correction was performed by multiscan method implemented in SADABS. Space groups were determined using XPREP implemented in APEX3. Structures were solved using SHELXL-2014 (direct methods) and refined using SHELXL-2014 (full-matrix least-squares on F^2) with anisotropic displacement contained in APEX3 program packages. Hydrogen atoms on carbon and nitrogen were calculated in ideal positions with isotropic placement parameters set to 1.2 × U_{eq} of the attached atoms.

Method for crystal growth

The purified compound **9** (28 mg) was dissolved in a mixed solvents of ethyl acetate (0.5 mL) and petroleum ether (5 mL), the solution was placed at minus 18 degrees Celsius. After five days, colourless crystals were crystallized out of the solution.

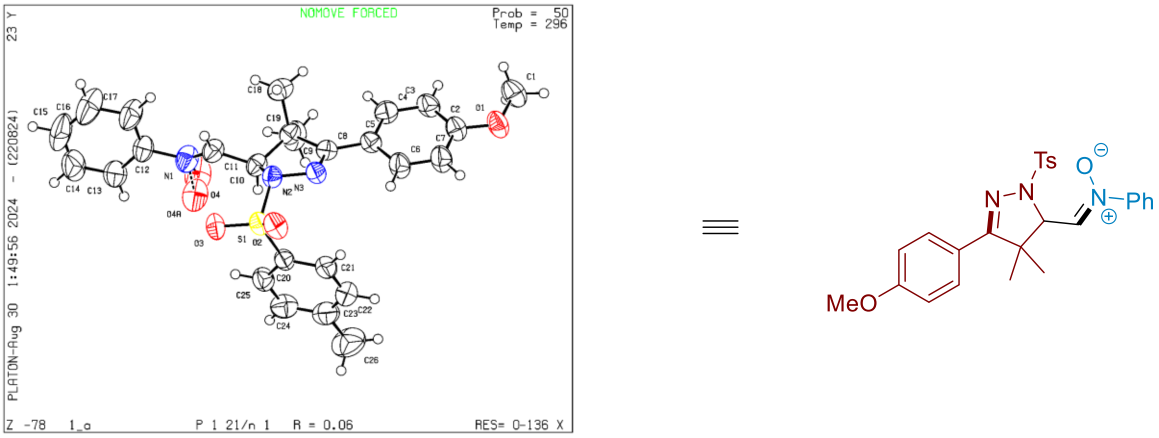

Figure S9 X-ray structure of product **9**

Crystal data and structure refinement for 9.

Identification code	Compound **9**
Empirical formula	$C_{26}H_{27}N_3O_4S$
Formula weight	477.56
Temperatur	296(2) K
Wavelength	0.71073 Å

Crystal system	monoclinic
Space group	$P2_1/n$
Unit cell dimensions	a = 10.4864(10)Å α = 90°.
	b = 20.3859(18) Å β = 112.669°.
	c = 12.3841(10) Å γ = 90°.
Volume	2442.9(4) $Å^3$
Z	4
Density (calculated)	1.298 g/m^3
Absorption coefficient	0.170 mm^{-1}
F(000)	1008
Crystal size	0.22×0.2×0.18 mm^3
Theta range for data collection	4.66 to 50.088°.
Index ranges	-12<=h<=12, -24<=k<=23, -14<=l<=12
Reflections collected	38022
Independent reflections	4297 [R_{int} = 0.0789, R_{sigma} = 0.0573]
Data / restraints / parameters	4297 / 20 / 320
Goodness-of-fit on F^2	1.055
Final R indices [I>2sigma(I)]	R1 = 0.0554, wR2 = 0.0993
R indices (all data)	R1 = 0.1171, wR2 = 0.1220
Largest diff. peak and hole	0.21 and -0.23 e.$Å^{-3}$

5. Mechanistic Studies

5.1 UV-vis spectrum

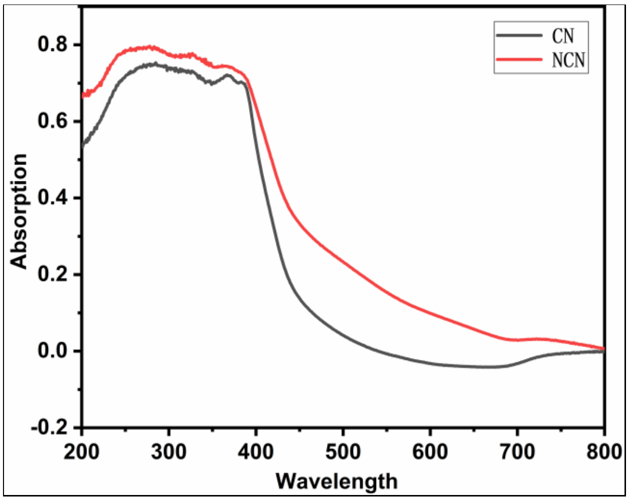

Figure S10 UV−vis absorption spectra.

5.2 TEMPO Trapping Experiment

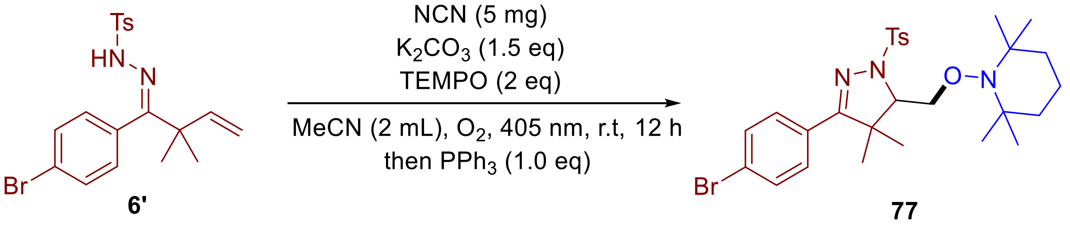

To an over dried bottle equipped with a magnetic stir bar, **6’** (42 mg, 0.1 mmol), TEMPO (31.3 mg, 0.4 mmol), K_2CO_3 (27.6 mg, 0.2 mmol), NCN (5 mg) were added. After that, MeCN (2 mL) was added to the bottle under a balloon of O_2. The mixture was stirred under 405 nm LED irradiation for 12 h at room temperature. The product **77** was purified by column chromatography (SiO_2, petroleum ether/ethyl acetate = 15:1 to 10:1), yielding **77** as white solid (40.6 mg, 71% yield).

Product 77

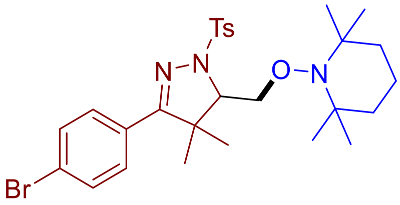

It was purified by column chromatography (SiO_2, petroleum ether/ethyl acetate = 15:1 to 10:1), yielding producy **77** as white solid (71% yield). **^{1}H NMR (400 MHz, $CDCl_3$)** δ = 7.77 (d, J = 8.4 Hz, 2H), 7.49 (s, 4H), 7.31 (d, J = 8.1 Hz, 2H), 4.60 (dd, J = 10.3, 4.6 Hz, 1H), 4.28 (t, J = 10.1

Hz, 1H), 3.35 (dd, J = 9.9, 4.6 Hz, 1H), 2.42 (s, 3H), 1.62 (d, J = 1.4 Hz, 1H), 1.58 – 1.44 (m, 5H), 1.43 (s, 3H), 1.29 – 1.19 (m, 9H), 1.07 (d, J = 11.6 Hz, 6H). **^{13}C NMR (101 MHz, $CDCl_3$)** δ = 164.1, 144.4, 131.6, 130.9, 129.5, 129.4, 129.2, 128.9, 124.3, 75.4, 70.1, 59.8, 51.7, 39.6 (d, *J=15.1 Hz*), 33.0 (d, *J=97.0 Hz*), 26.1, 21.6, 20.2 (d, *J=16.2 Hz*), 19.9, 17.1.

^{1}H NMR (400 MHz, $CDCl_3$) and ^{13}C NMR (101 MHz, $CDCl_3$)

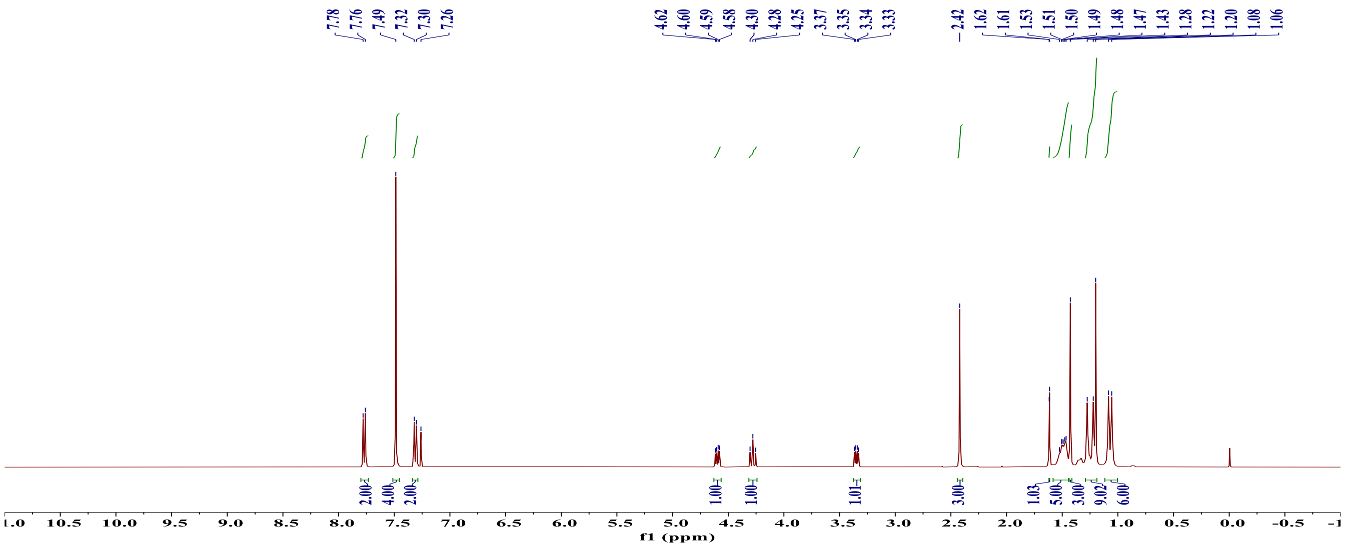

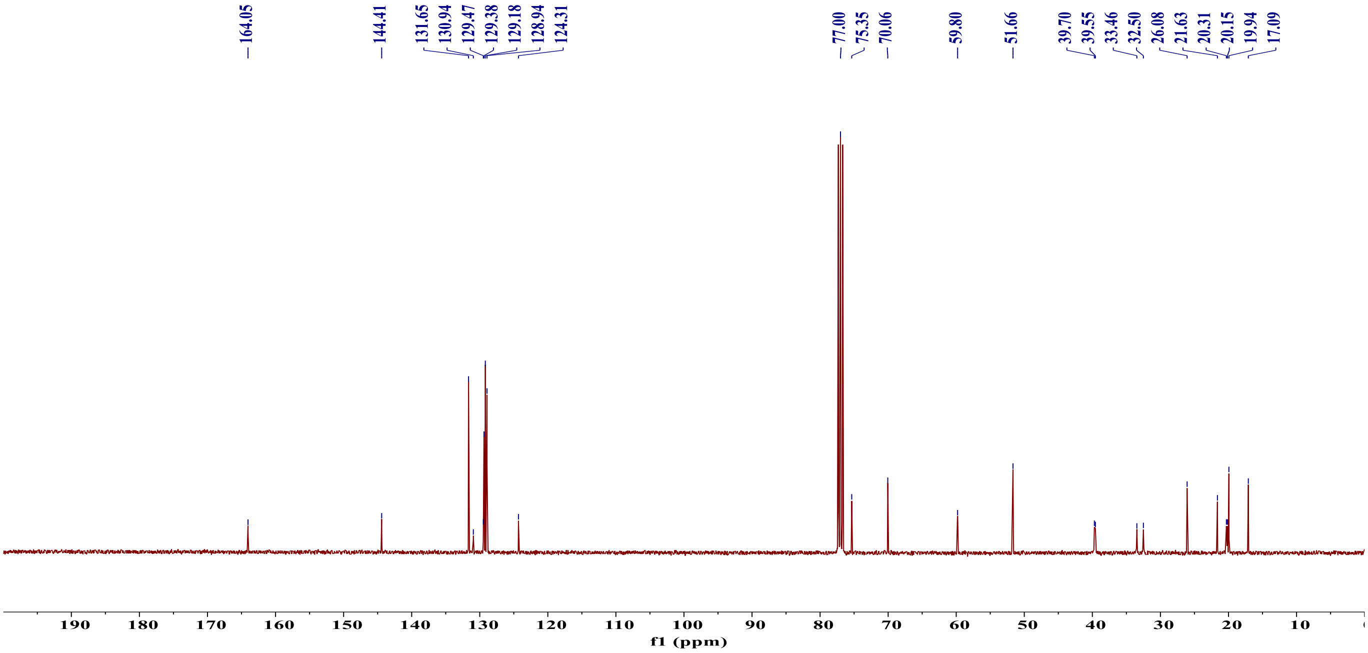

Results: In the presence of TEMPO, the reaction was completely inhibited, and the corresponding TEMPO adduct 77 was obtained in 71% yield, which indicated the intermediacy of a carbon-centered radical through 5-exo-cyclization of N-radical.

5.3 Determination of Quantum Yield

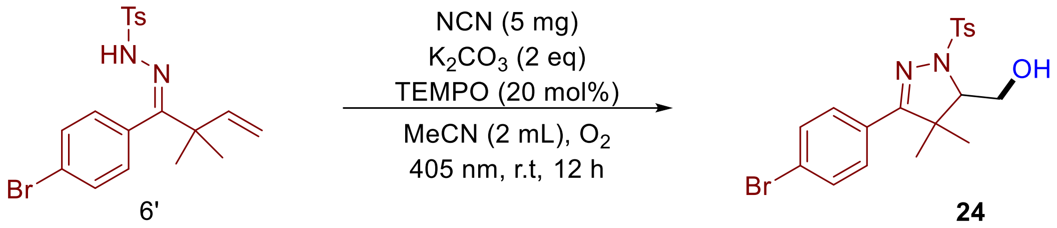

A cuvette was charged with 6' (0.05 mmol), NCN (5 mg), K_2CO_3 (2 eq), TEMPO (20 mol%), and 2.0 mL MeCN. The reaction was irradiated by LEDs (λ = 405 nm, slit width = 3.0 mm, slit height 5.0 mm with intensity of 0.774 mW·cm^{-2}) for 19545 s (5 h 25 min 45 s). The quantum yield was determined as follows.

ϕ = Mole number for product/Mole number for absorption of photons = 0.081

$$\Phi = \frac{nN_A/t}{fP\lambda/hc}$$

n: the mole number of the product 24; t: reaction time (19545 s, 5 h 25 min 45 s); N_A: 6.02 x10^{23} mol^{-1}; f: $1\text{-}10^{-A}$(405 nm, A= 0.47); P: P = F*S (E: illumination intensity, E= 0.7740 mW/cm^2; S: the area thatirradiated S= 0.15 cm^2), wavelength (λ= 4.05 x10^{-7} m); h: planck constant (h = 6.626 x10^{-34} J*s); c: velocityof light (c = 3 x10^8 m/s).

Results:* *This result indicated that the reaction likely proceeded via a sequential photoredox process rather than by a radical chain process.

References:

[1] X. Hu, J. Chen, J. Chen, D. Yan, W. Xiao, *Chemistry A European J.* **2016**, *22*, 14141-14146.

[2] I. Triandafillidi, C. G. Kokotos, *Org. Lett.* **2016**, *19*, 106-109.

[3] T. Xu, W. Lu, X. F. Wu, W. Chen, *Journal of Catalysis* **2019**, *378*, 63-67.

[4] C. Zhu, R. Wang, J. R. Falck, *Org. Lett.* **2012**, *14*, 3494-3497.

[5] P. S. Fier, J. F. Hartwig, *Angew. Chem. Int. Ed.* **2013**, *52*, 2092-2095.

[6] H. Li, C. Shan, B. Pan, *Environ. Sci. Technol.* **2018**, *52*, 2197-2205.

[7] P. Ni, L. Yang, Y. Shen, L. Zhang, Y. Ma, M. Sun, R. Cheng, J. Ye, *J. Org. Chem.* **2022**, *87*, 12677-12687.

[8] H. Zhou, P. Mukherjee, R. Liu, E. Evrard, D. Wang, J. M. Humphrey, T. W. Butler, L. R. Hoth, J. B. Sperry, S. K. Sakata, C. J. Helal, C. W. am Ende, *Org. Lett.* **2018**, *20*, 812-815.

[9] K. Shibatomi, M. Kotozaki, N. Sasaki, I. Fujisawa, S. Iwasa, *Chemistry A European J.* **2015**, *21*, 14095-14098.

[10] D. Luo, Y. Huang, X. Hong, D. Chen, G. Li, X. Huang, W. Gao, M. Liu, Y. Zhou, H. Wu, *Adv. Synth. Catal.* **2019**, *361*, 961-964.

[11] D. Shen, T. Ren, H. Zhang, M. Chao, C. Sun, P. Gong, S. Zhang, Y. M. Lee, S. Fukuzumi, W. Nam, *ACS Catal.* **2024**, *14*, 2162-2172.

[12] J. M. Khurana, S. Kumar, *Tetrahedron Lett.* **2009**, *50*, 4125-4127.

[13] F. Aiello, M. G. Simons, J. W. van Velde, P. Dani, *Molecules* **2021**, *26*, 3811.

[14] N. Kataoka, Q. Shelby, J. P. Stambuli, J. F. Hartwig, Air Stable, *J. Org. Chem.* **2002**, *67*, 5553-5566.

[15] M. Li, J. Huang, K. Xu, S. Gong, Y. Liang, X. Xu, Z. Liu, Z. Wang, S. Wang, *Spectrochimica Acta Part A: Molecular and Biomolecular Spectroscopy* **2024**, *318*, 124476.

[16] W. Cui, R. Kargbo, Z. Sajjadi-Hashemi, F. Ahmed, J. Gauuan, *Synlett* **2012**, *2012*, 247-250.

[17] D. M. Green, I. Goljer, D. S. Andraka, M. Chengalvala, L. Shanno, W. Hurlburt, J. C. Pelletier, *J. Comb. Chem.* **2008**, *11*, 117-125.

[18] S. Li, X. Su, M. Abdullah, Y. Sun, G. Li, X. Cheng, Y. Lin, Y. Cai, Q. Jin, *Int. J. Mol. Sci.* **2018**, *19*, 2273.

[19] Y. Zhou, L. Zhang, W. Huang, Q. Kong, X. Fan, M. Wang, J. Shi, *Carbon* **2016**, *99*, 111-117.

[20] J. Zhang, M. Yan, G. Sun, X. Li, B. Hao, K. Liu, *Chemosphere* **2022**, *304*, 135318.

[21] H. Ashiq, N. Nadeem, A. Mansha, J. Iqbal, M. Yaseen, M. Zahid, I. Shahid, *Journal of Physics and Chemistry of Solids* **2022**, *161*, 110437.

6. NMR Spectra of products

^{1}H NMR (400 MHz, $CDCl_3$), ^{13}C NMR (101 MHz, $CDCl_3$) of product 3

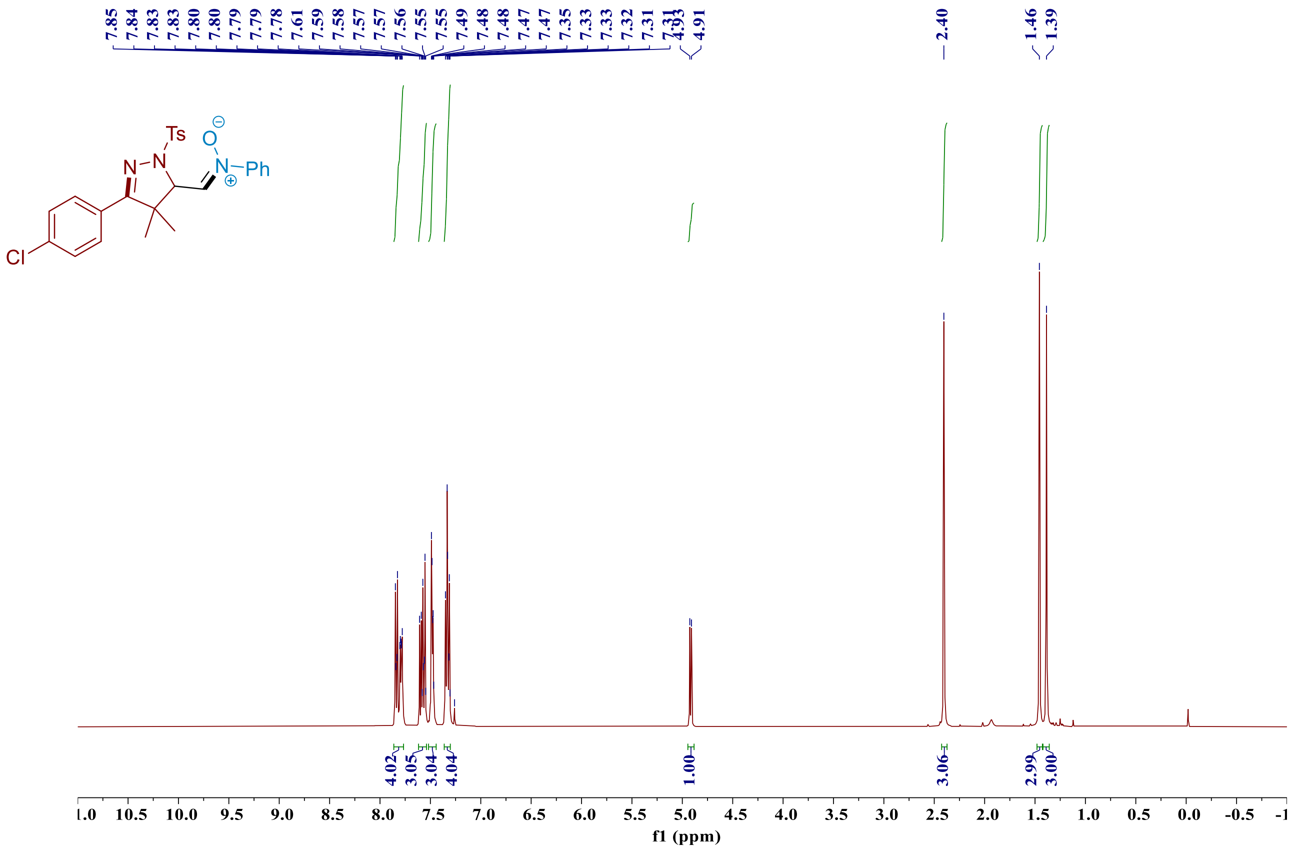

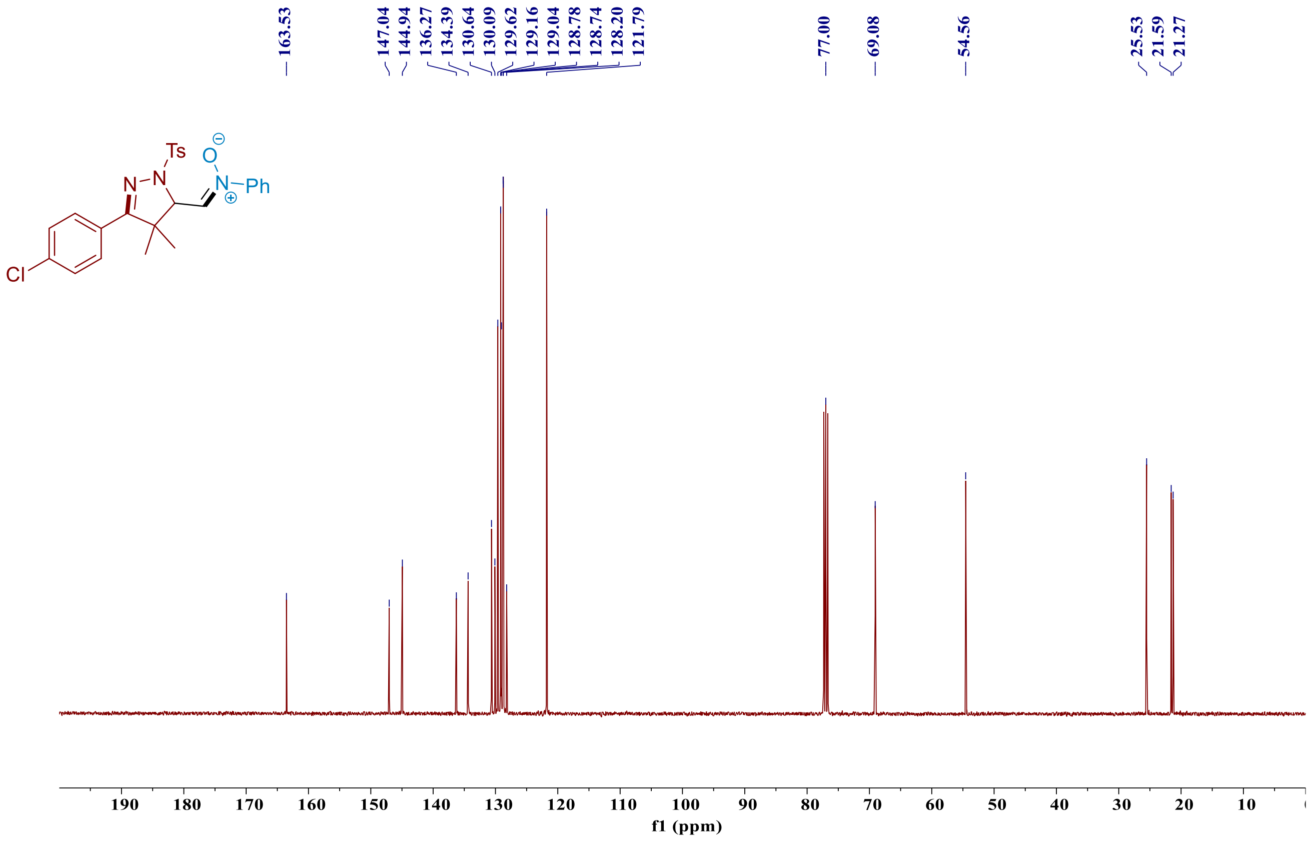

^{1}H NMR (400 MHz, $CDCl_3$), ^{13}C NMR (101 MHz, $CDCl_3$) of product 5

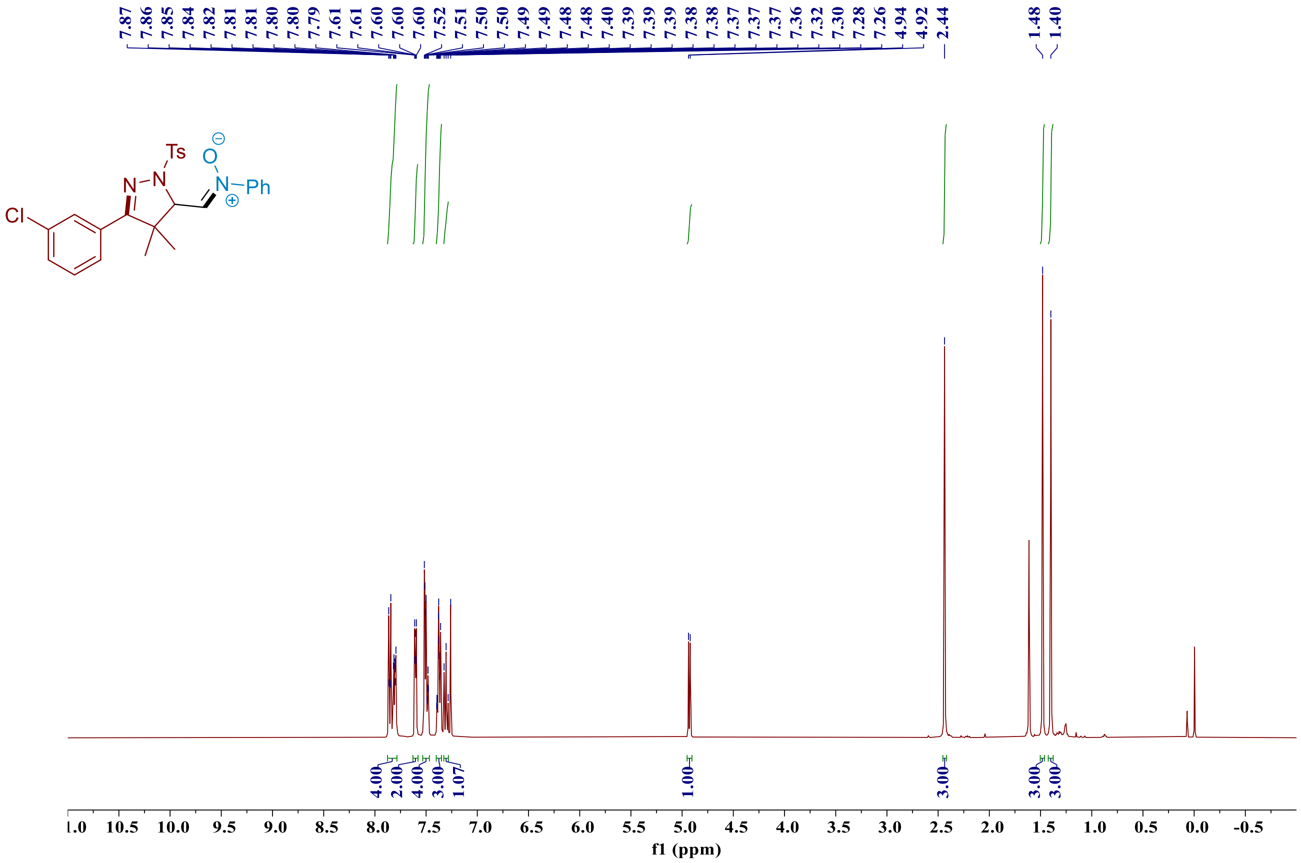

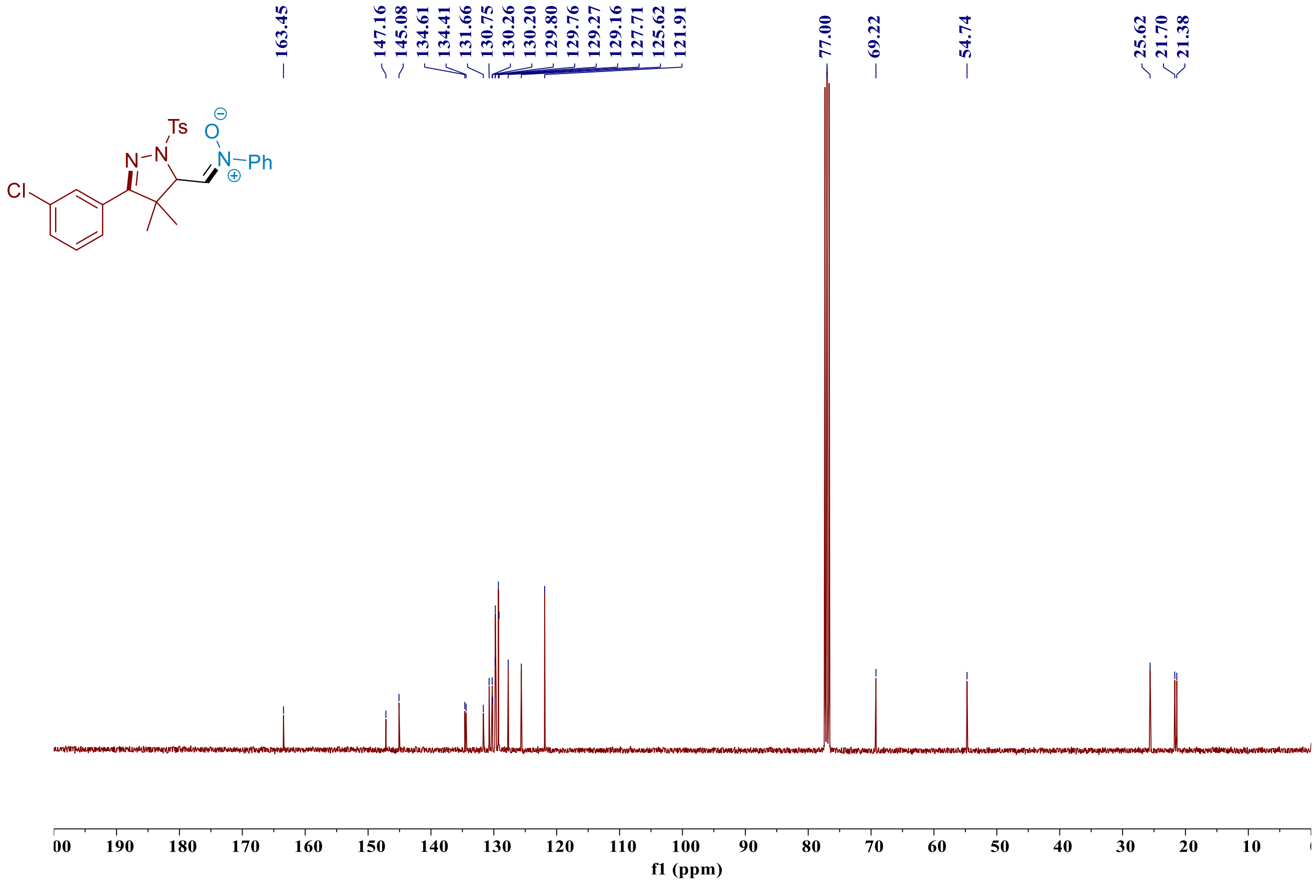

^{1}H NMR (400 MHz, $CDCl_3$), ^{13}C NMR (101 MHz, $CDCl_3$) of product 6

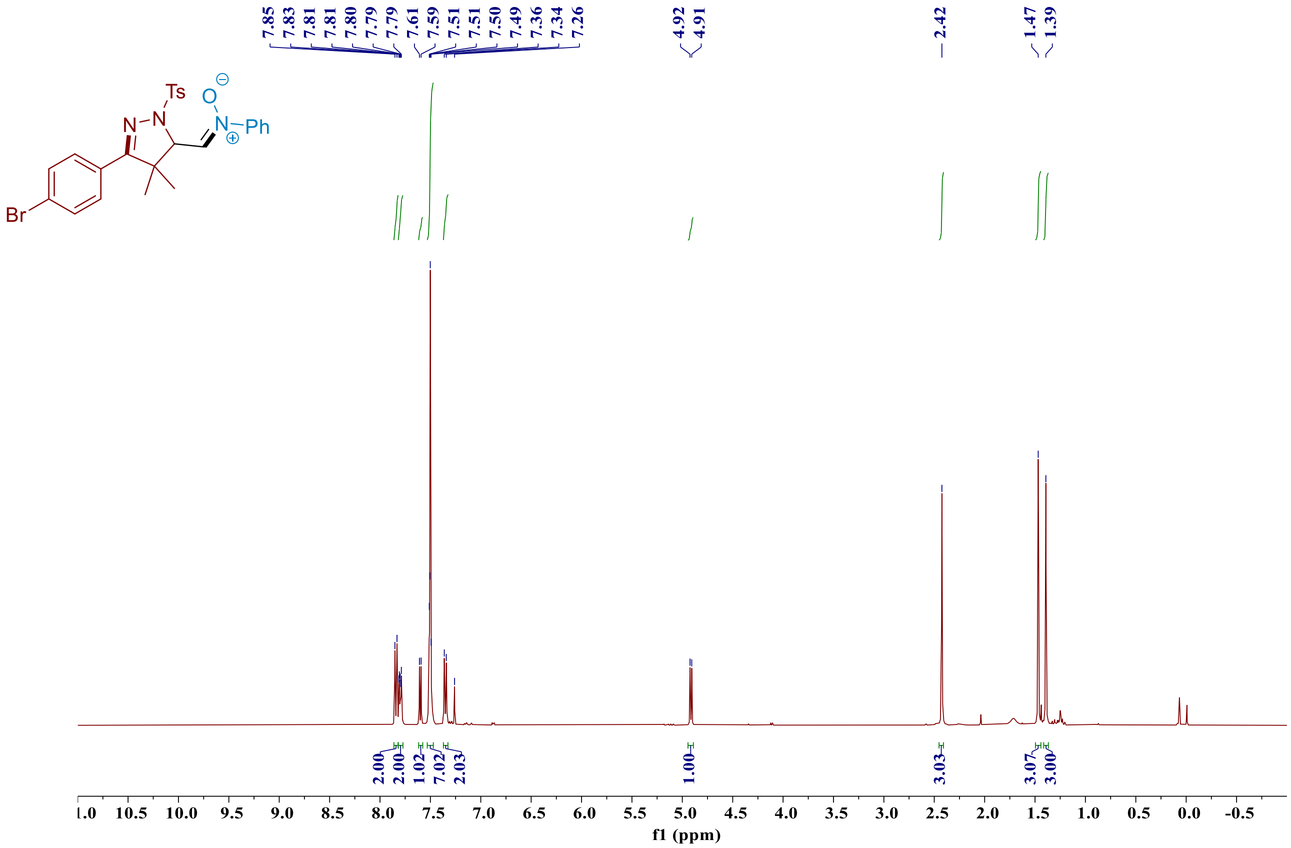

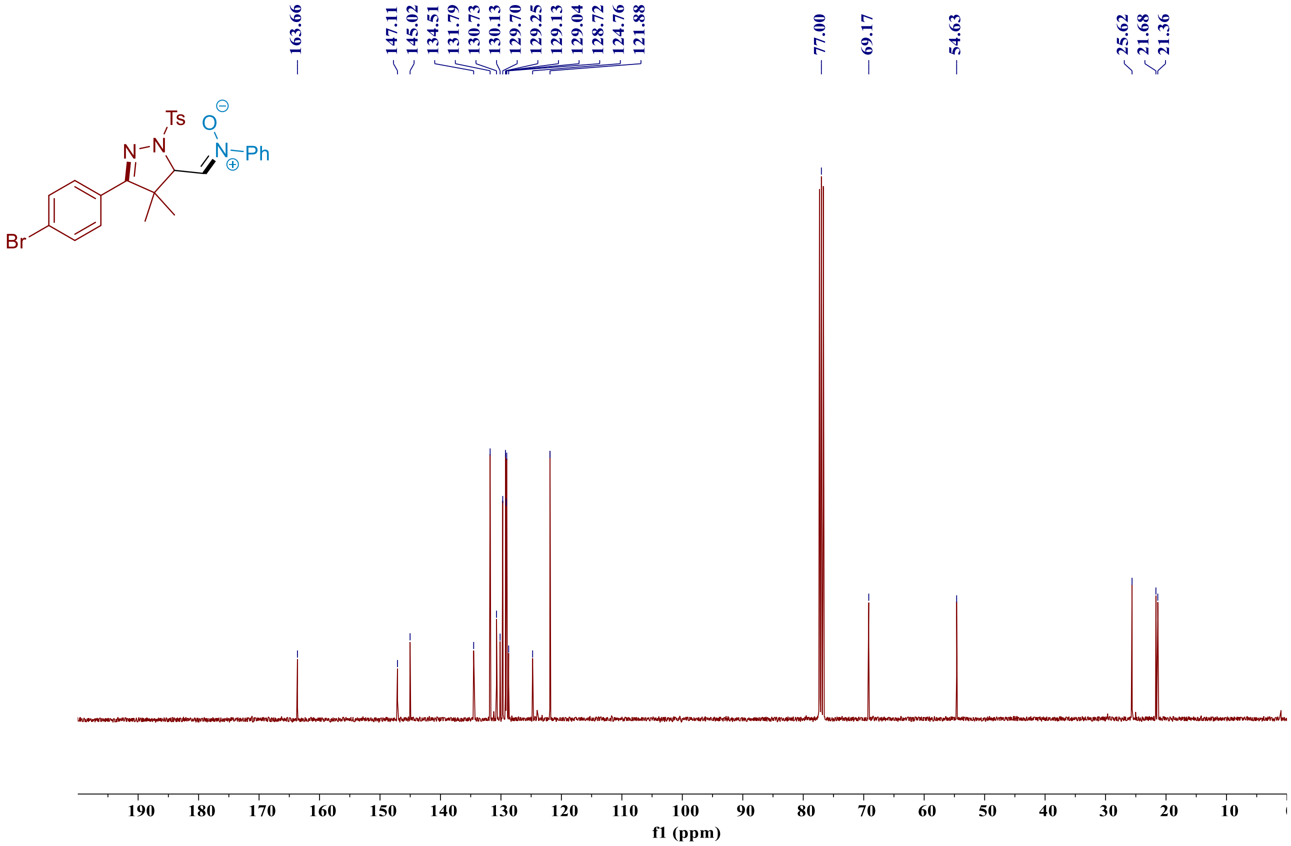

^{1}H NMR (400 MHz, $CDCl_3$), ^{13}C NMR (101 MHz, $CDCl_3$) of product 7

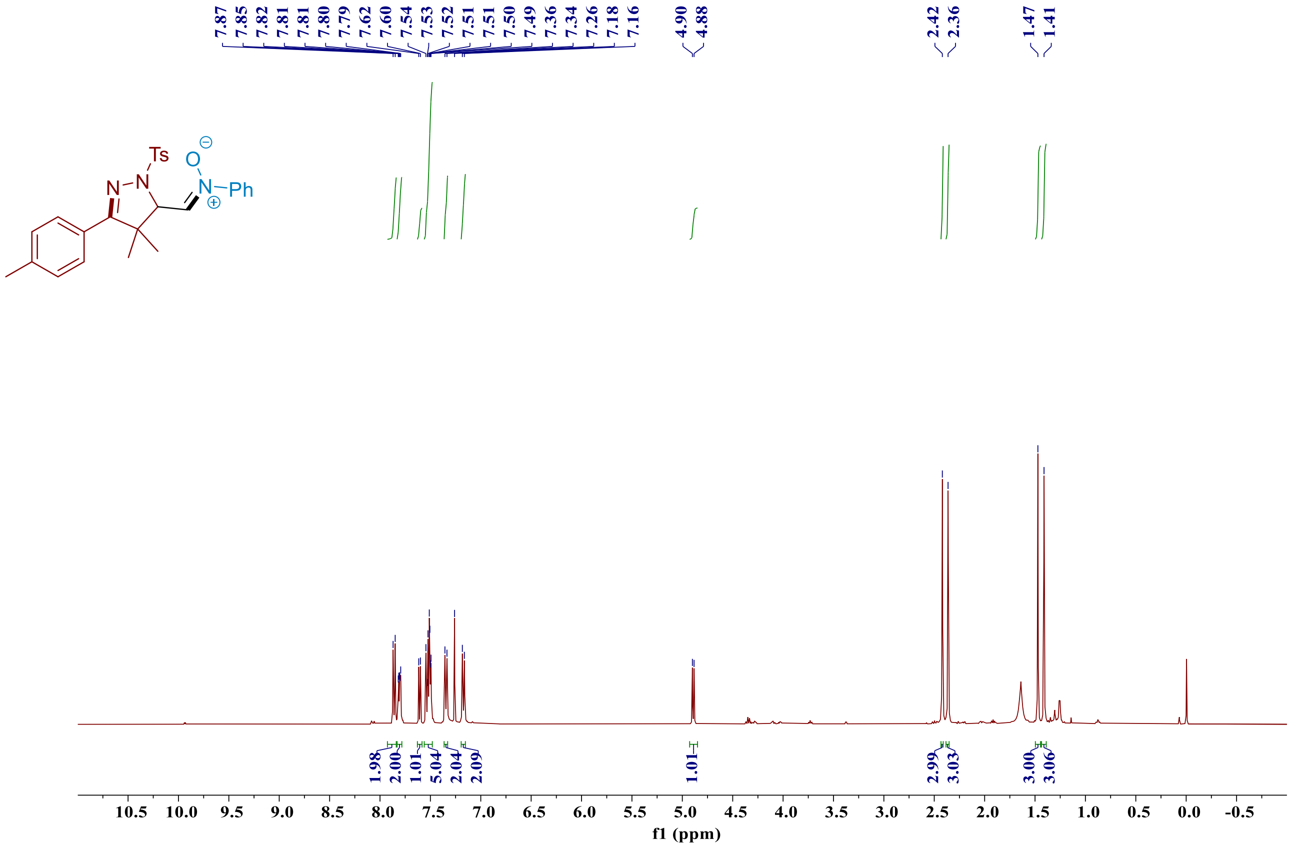

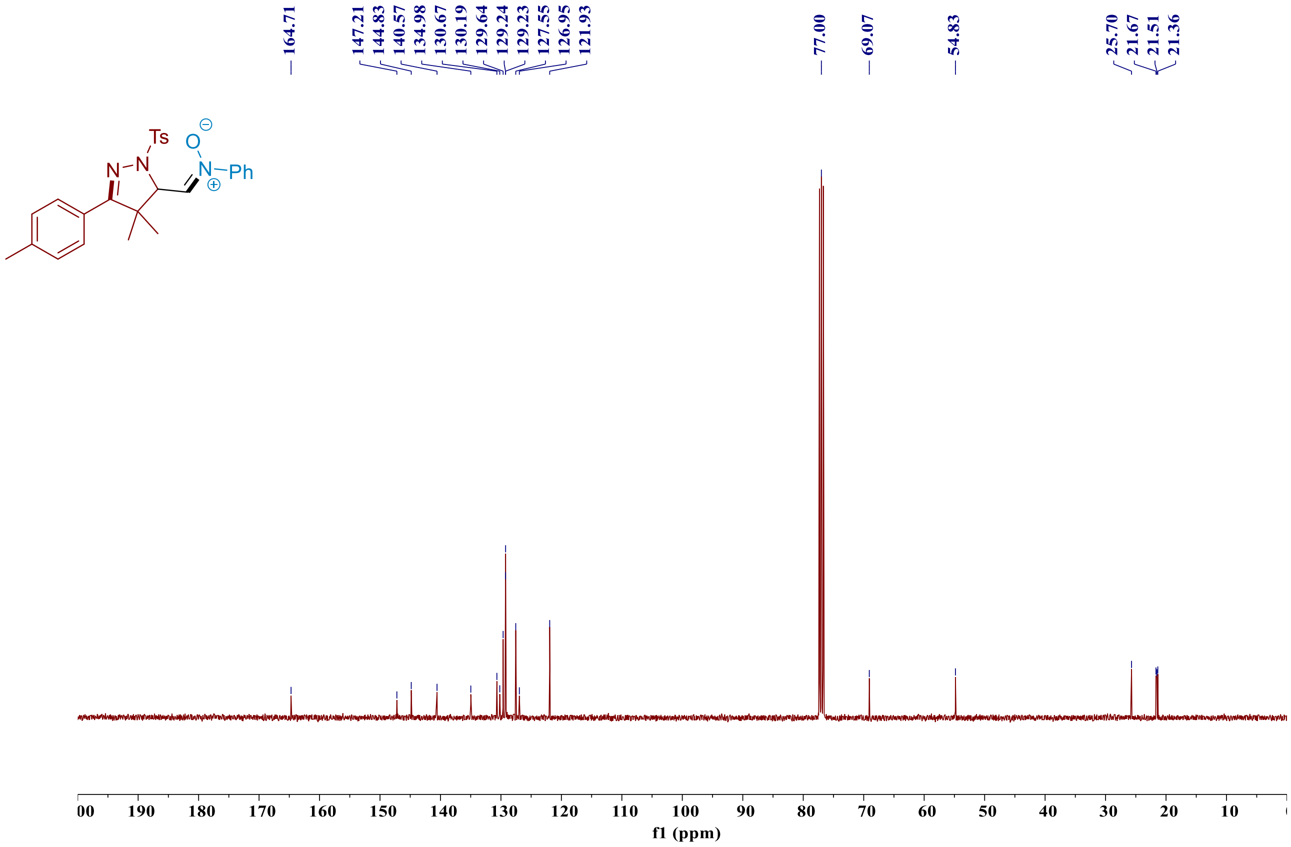

^{1}H NMR (400 MHz, $CDCl_3$), ^{13}C NMR (101 MHz, $CDCl_3$) of product 8

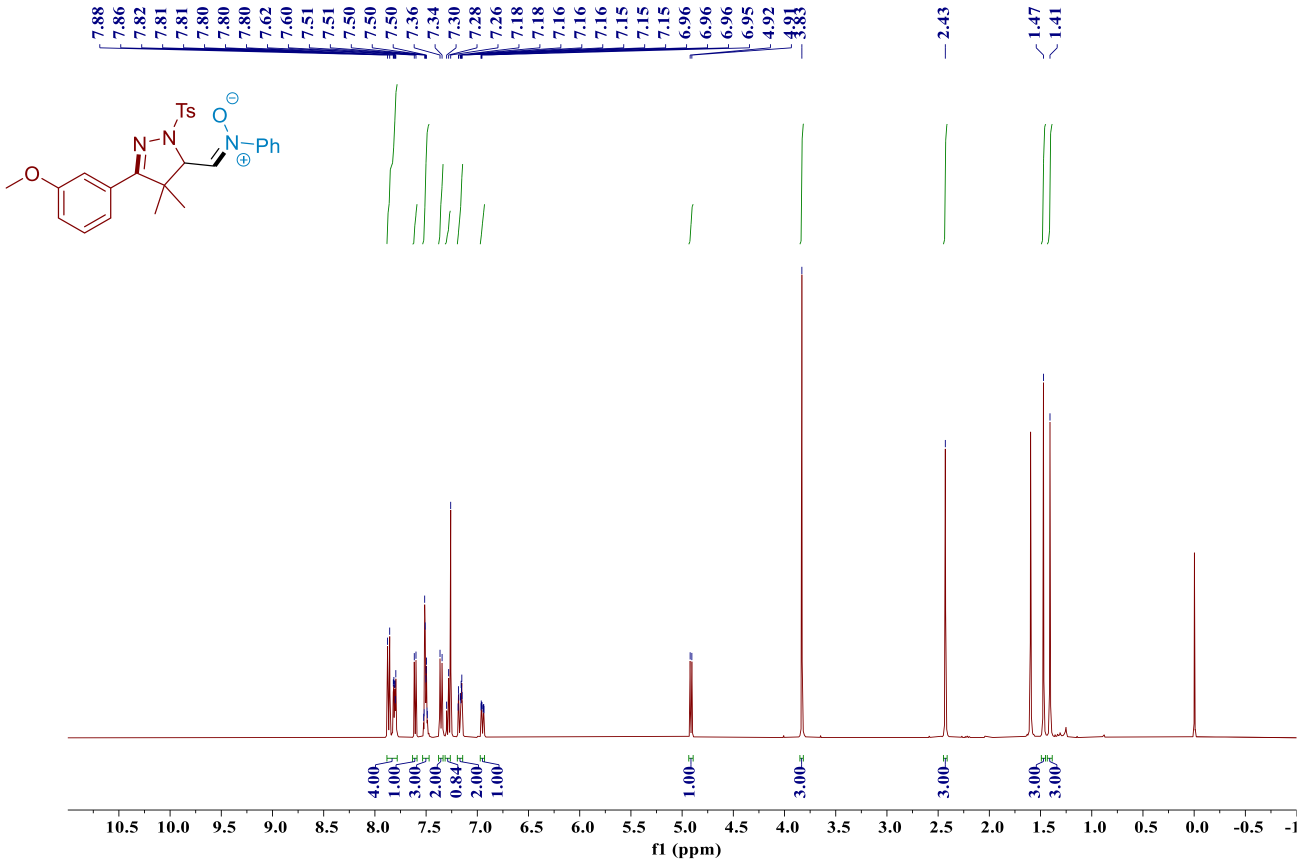

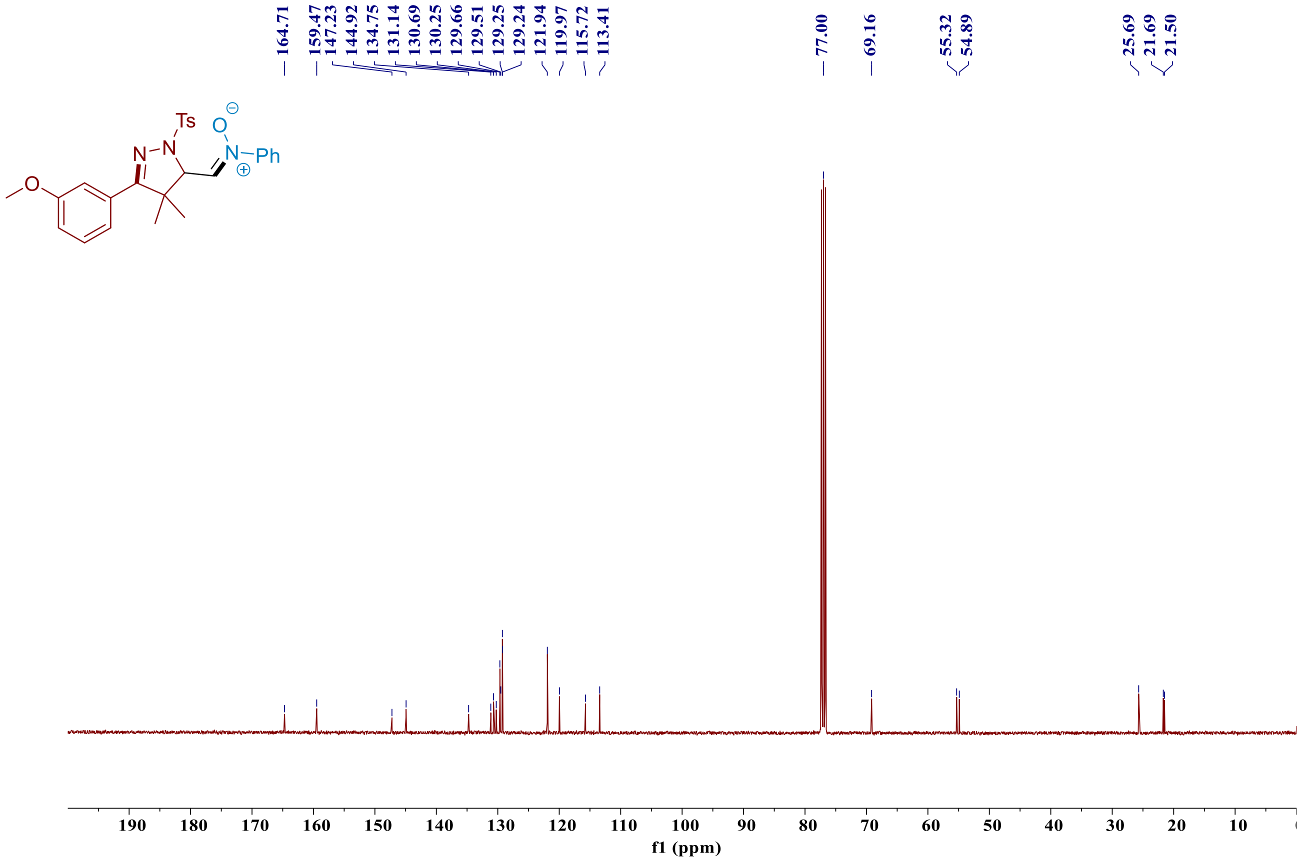

^{1}H NMR (400 MHz, $CDCl_3$), ^{13}C NMR (101 MHz, $CDCl_3$) of product 9

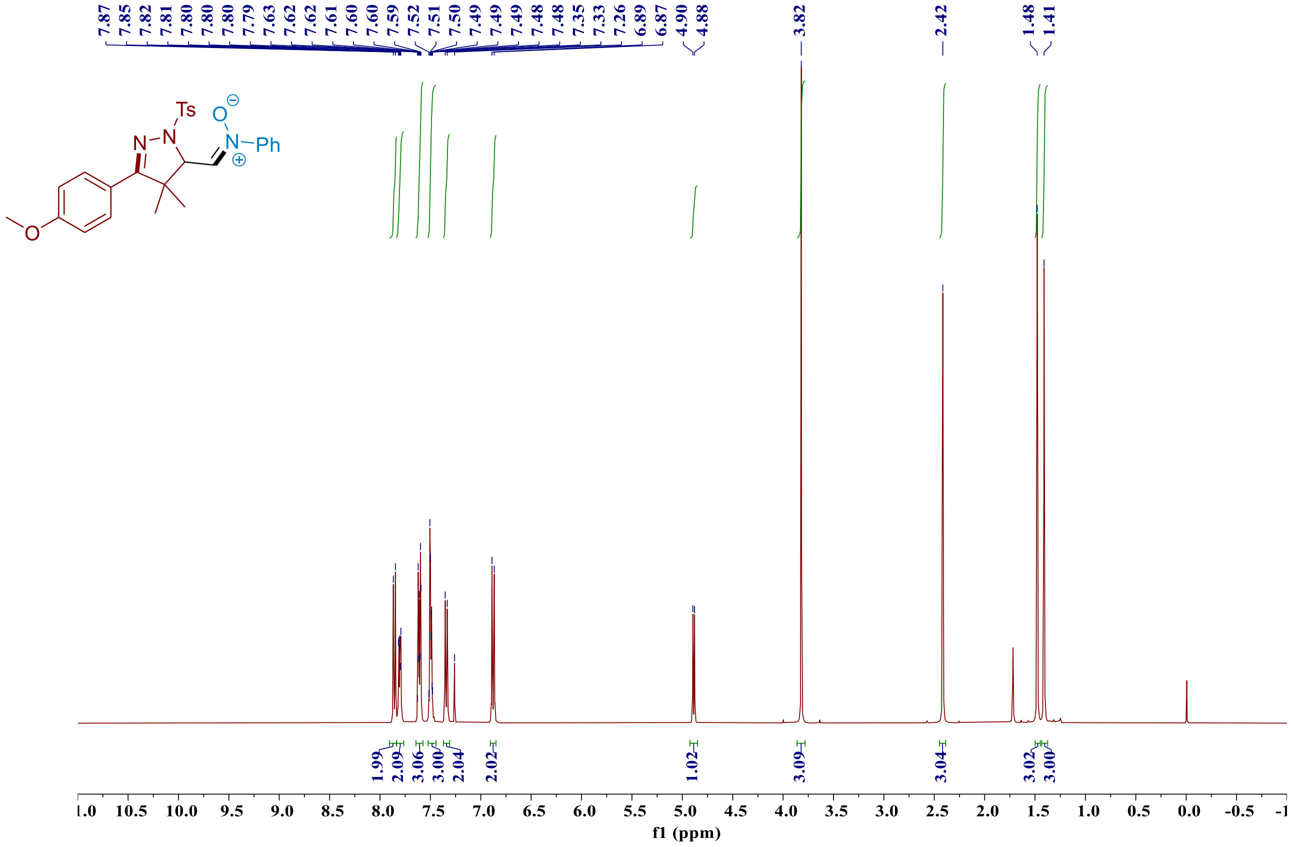

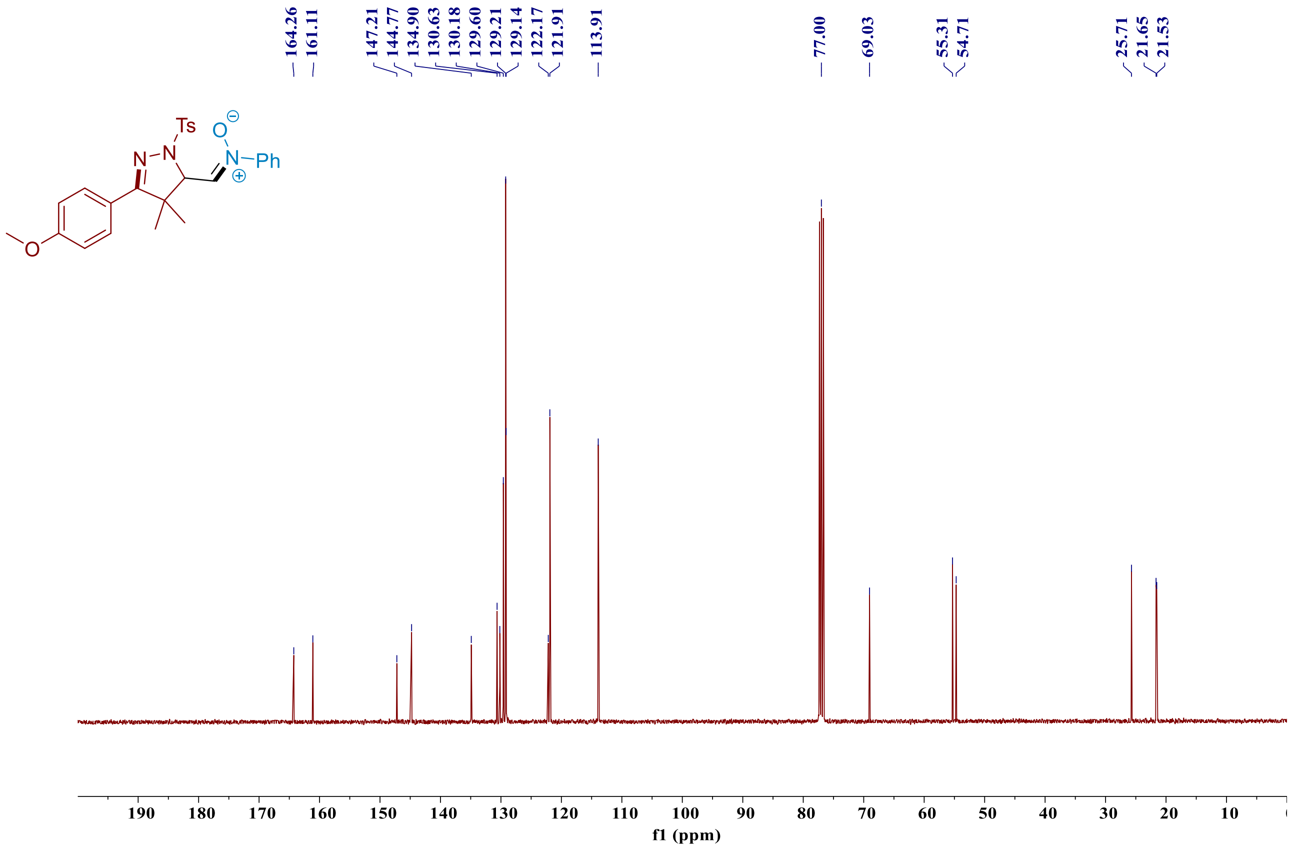

^{1}H NMR (400 MHz, $CDCl_3$), ^{13}C NMR (101 MHz, $CDCl_3$) of product 10

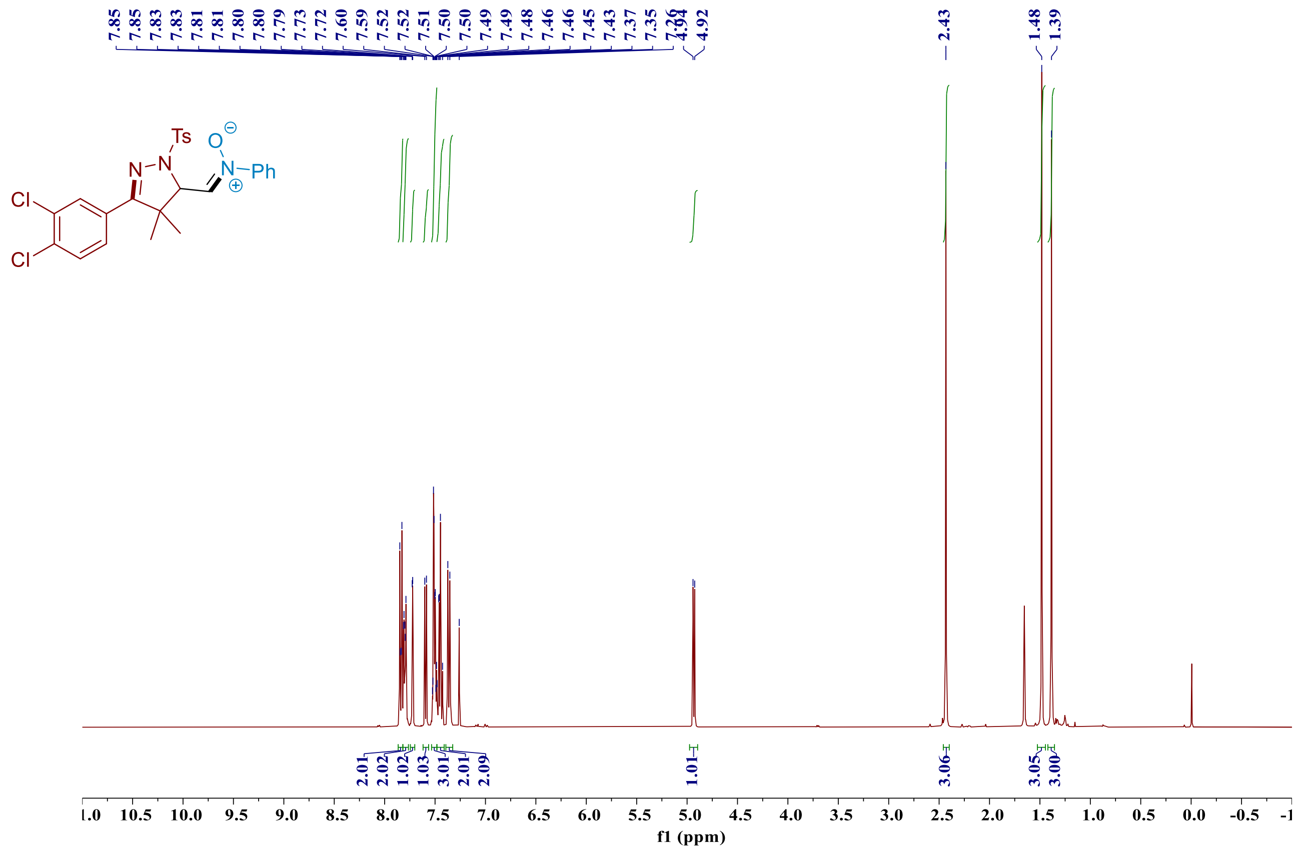

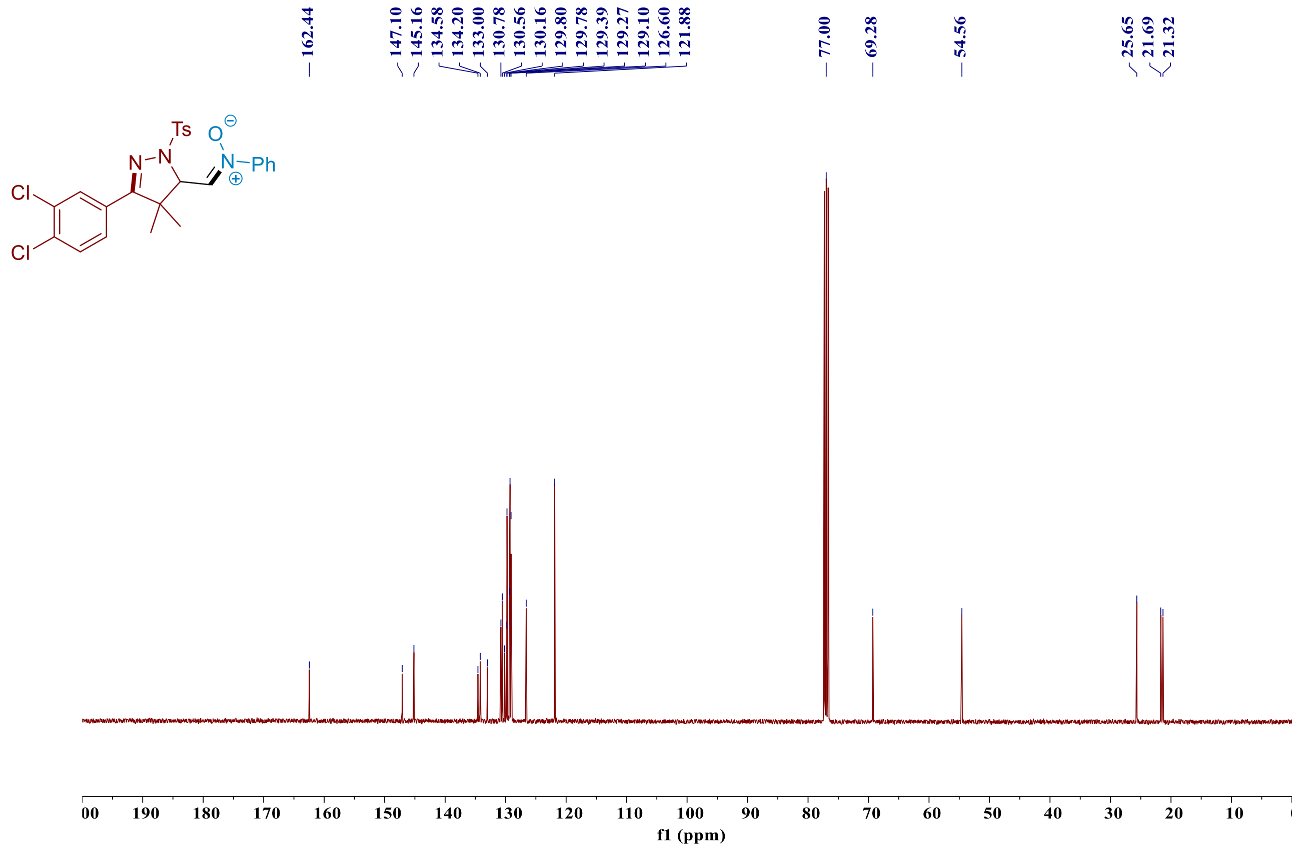

^{1}H NMR (400 MHz, $CDCl_3$), ^{13}C NMR (101 MHz, $CDCl_3$) of product 11

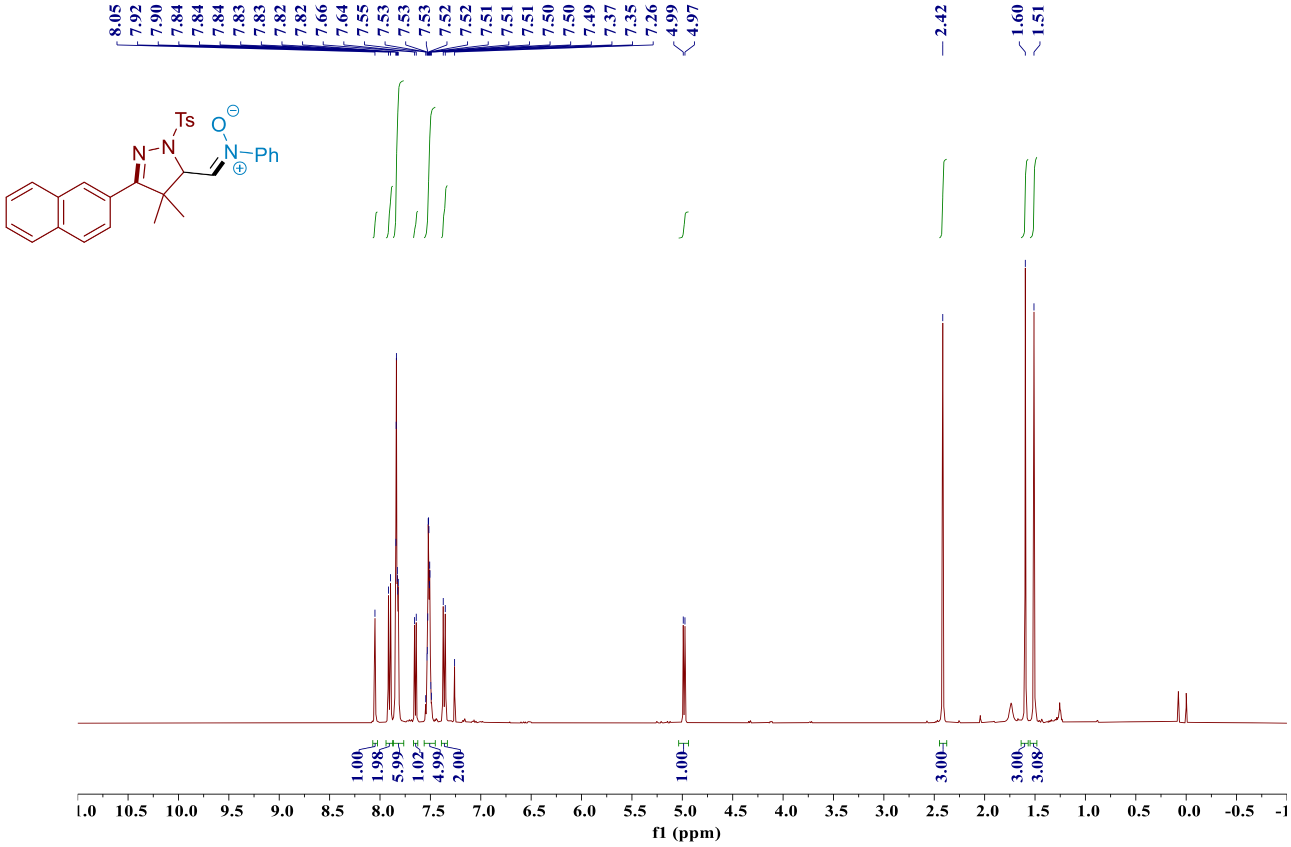

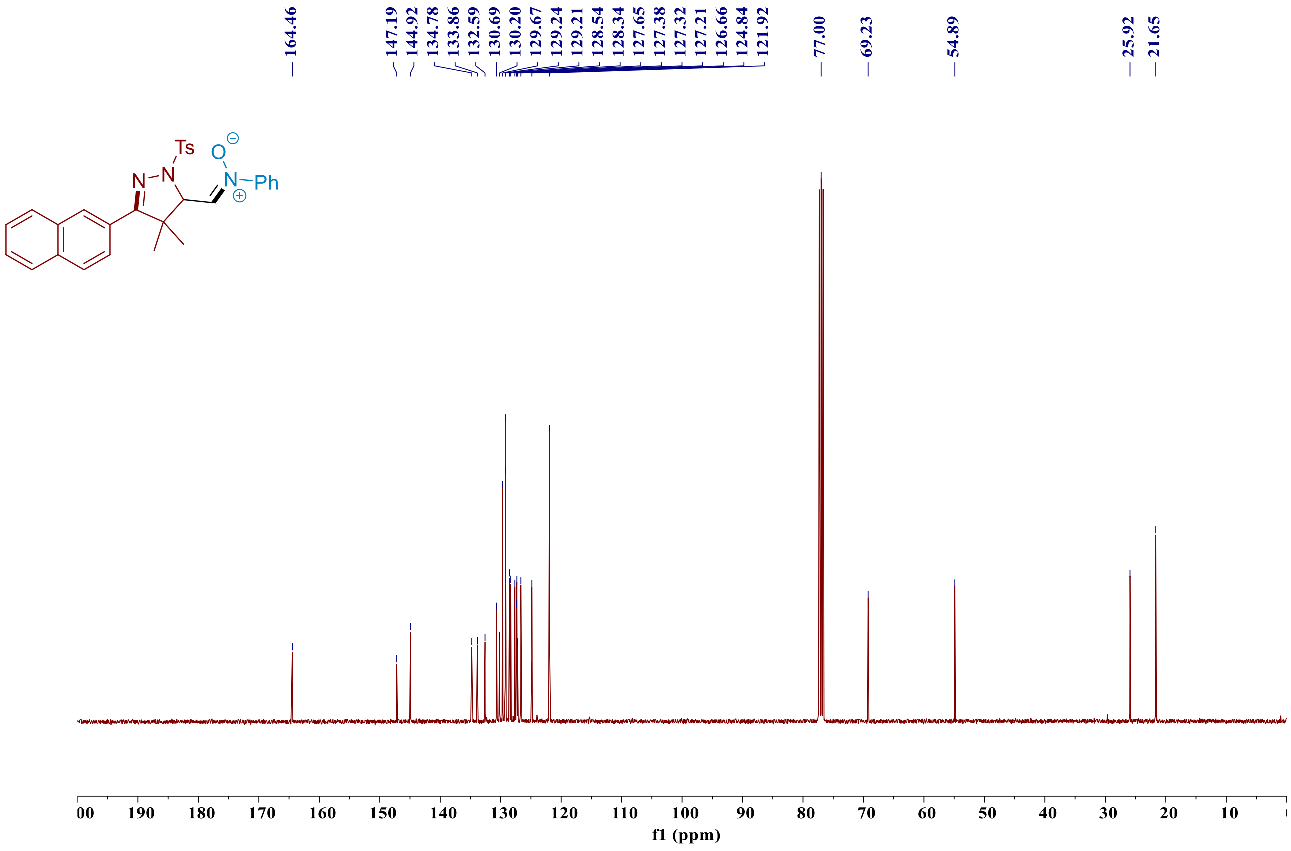

^{1}H NMR (400 MHz, $CDCl_3$), ^{13}C NMR (101 MHz, $CDCl_3$) of product 12

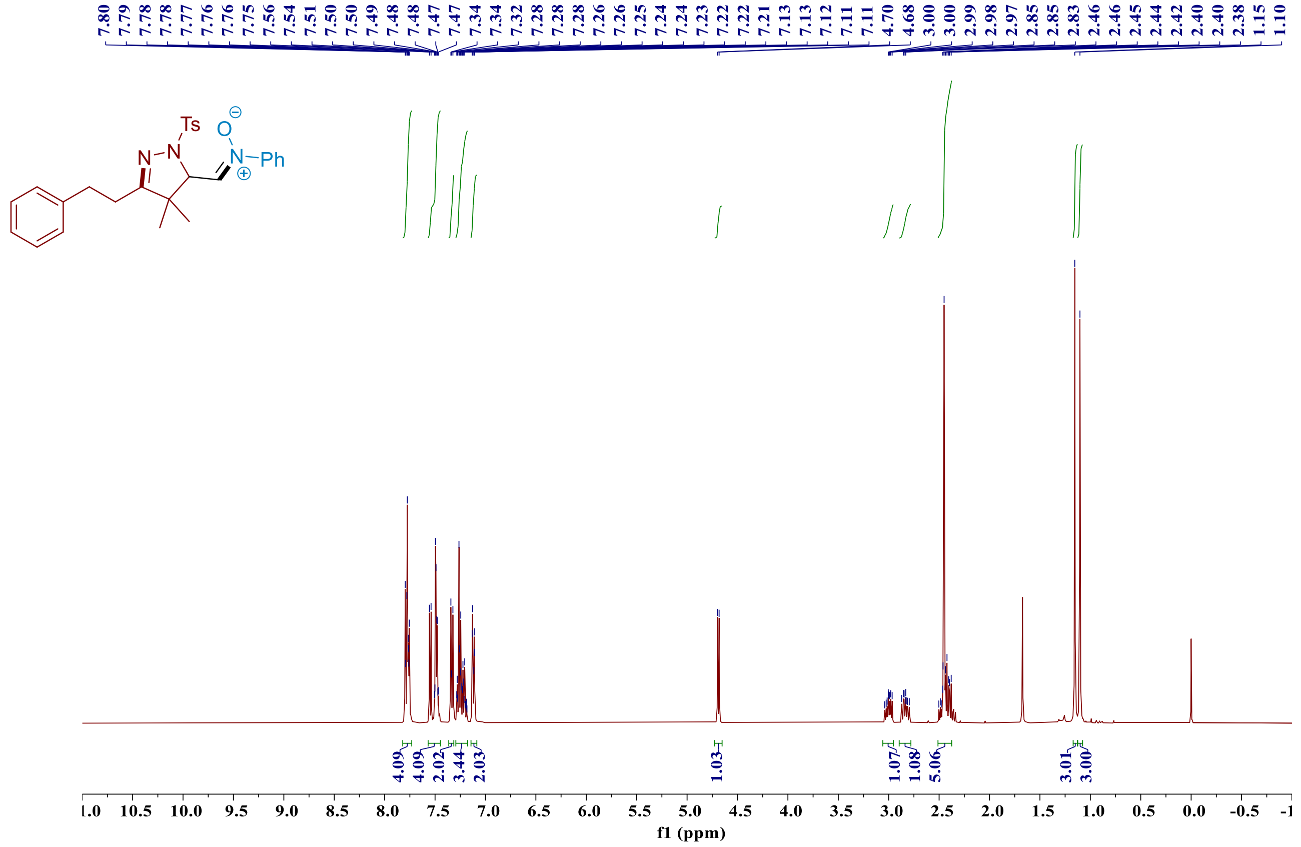

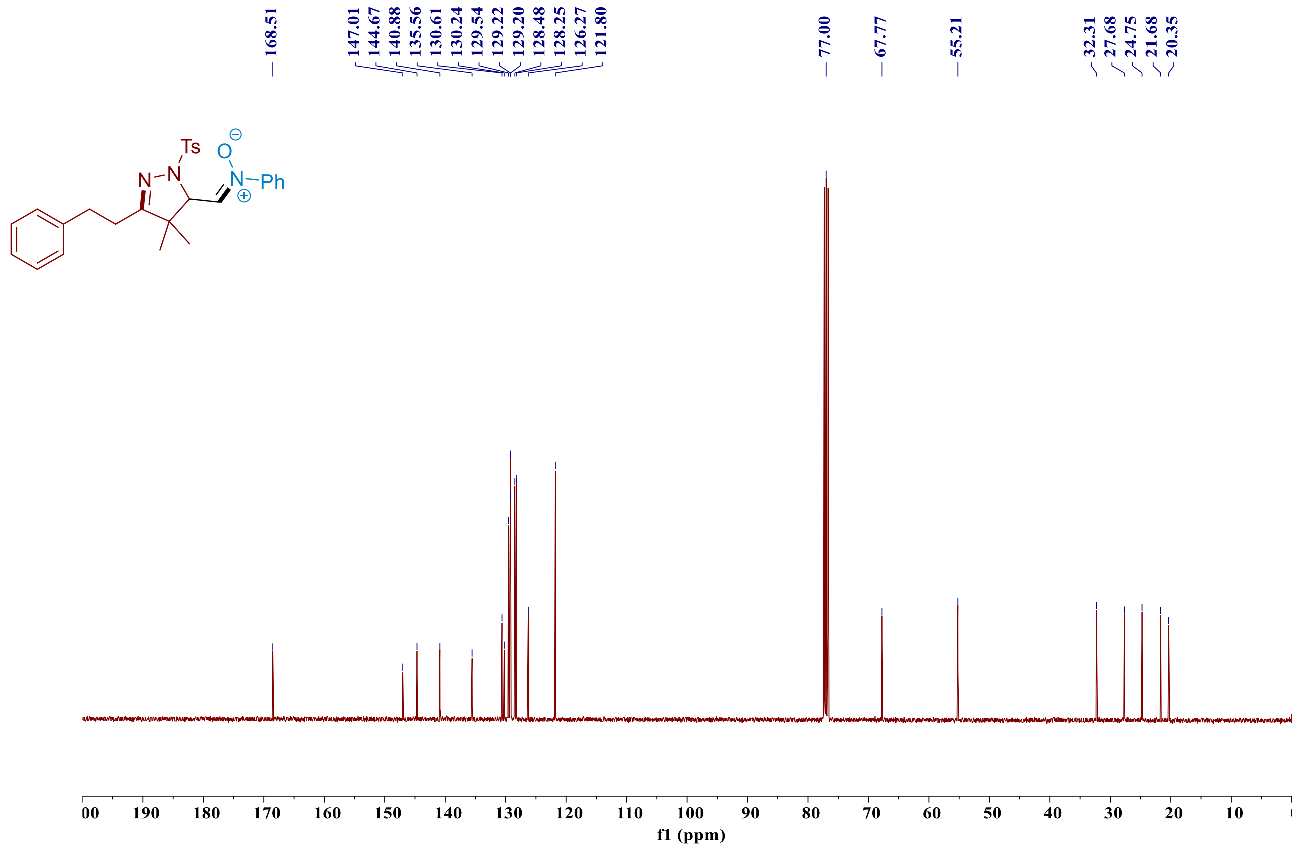

^{1}H NMR (400 MHz, CDCl$_3$), ^{13}C NMR (101 MHz, CDCl$_3$) of product 13

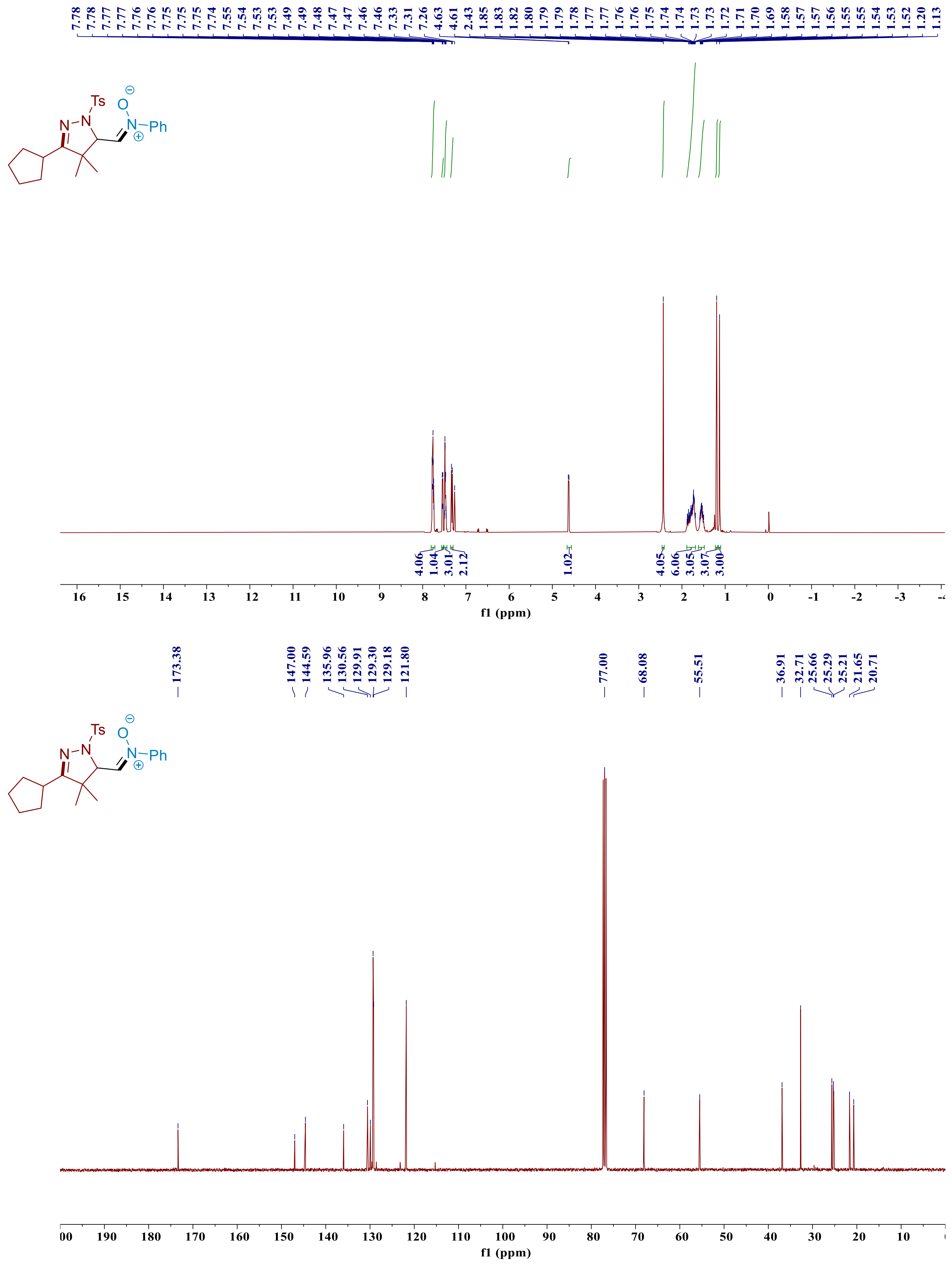

^{1}H NMR (400 MHz, $CDCl_3$), ^{13}C NMR (101 MHz, $CDCl_3$) of product 14

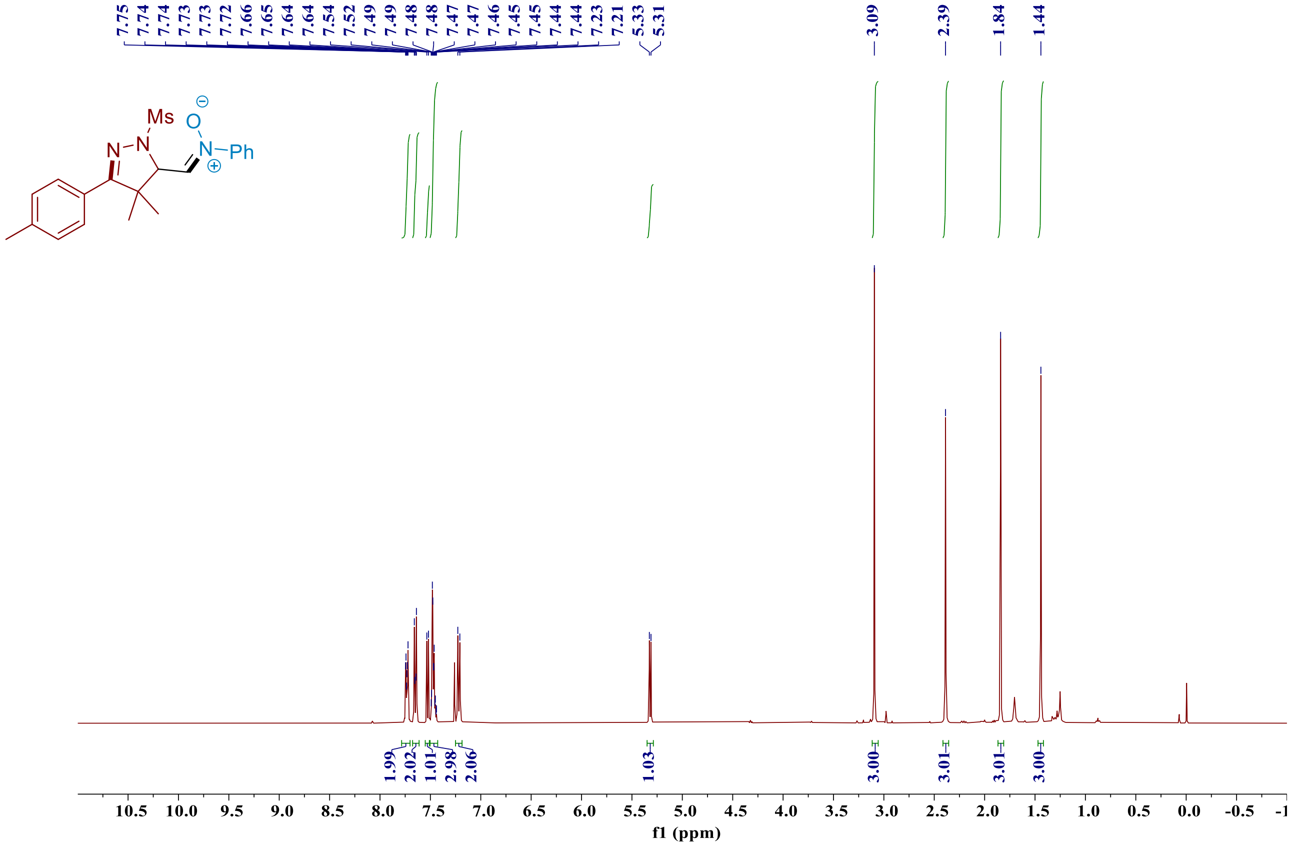

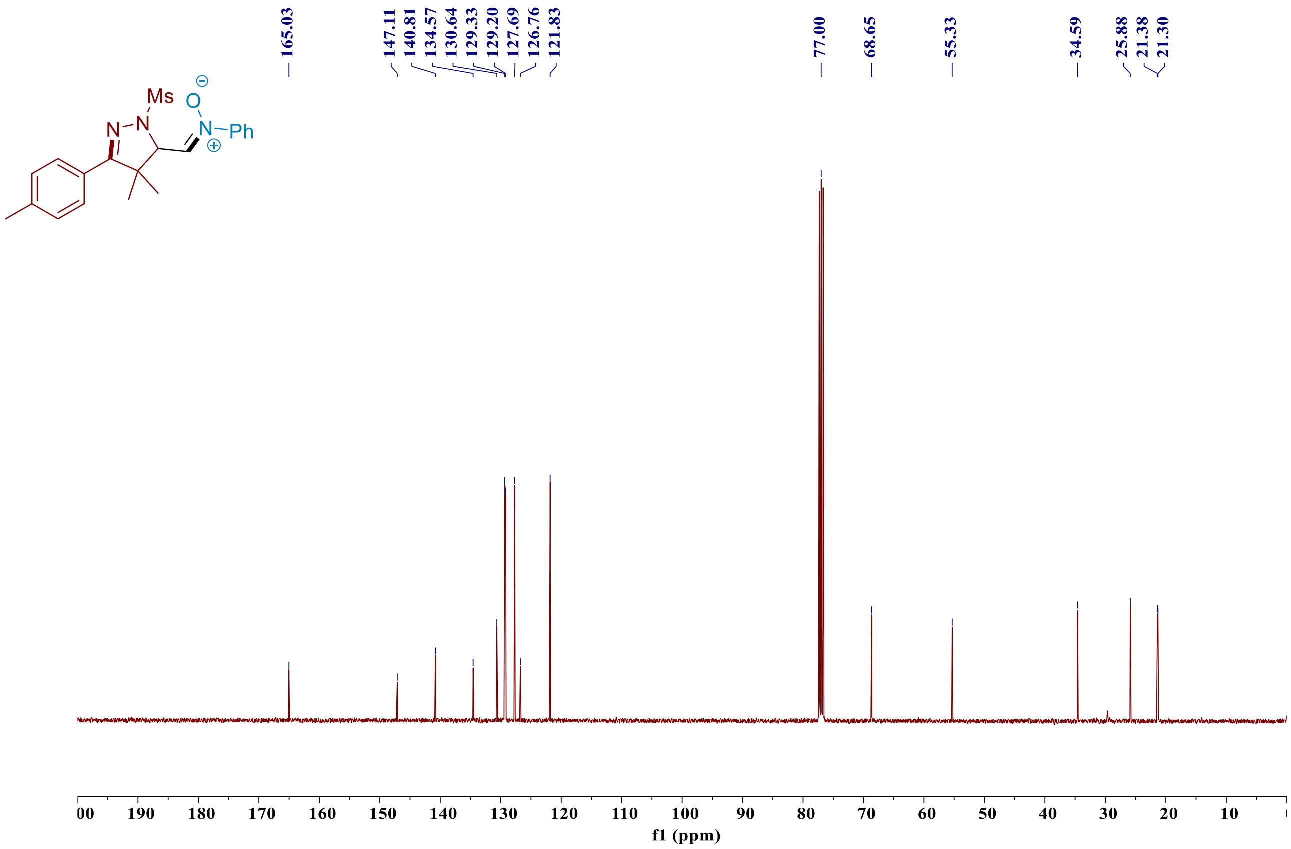

^{1}H NMR (400 MHz, $CDCl_3$), ^{13}C NMR (101 MHz, $CDCl_3$) of product 15

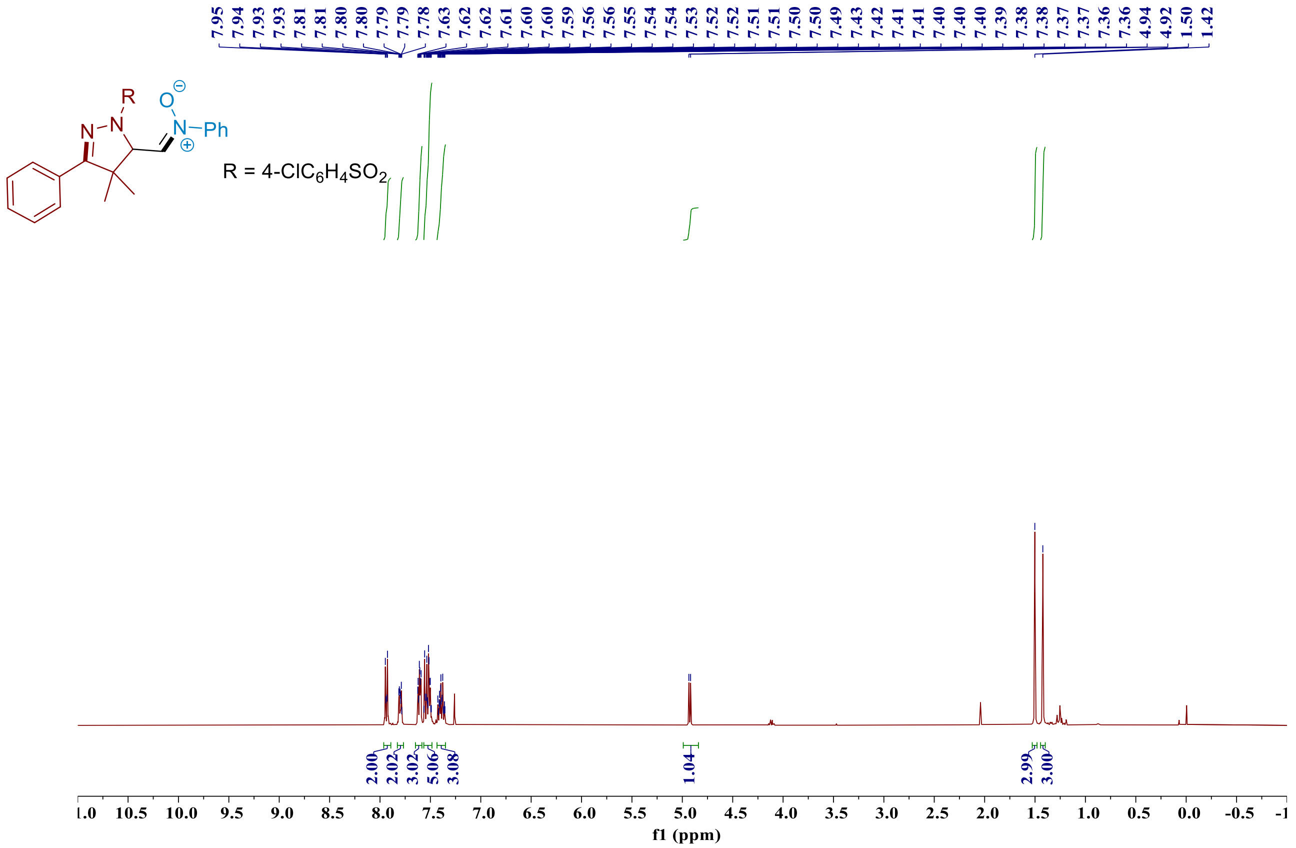

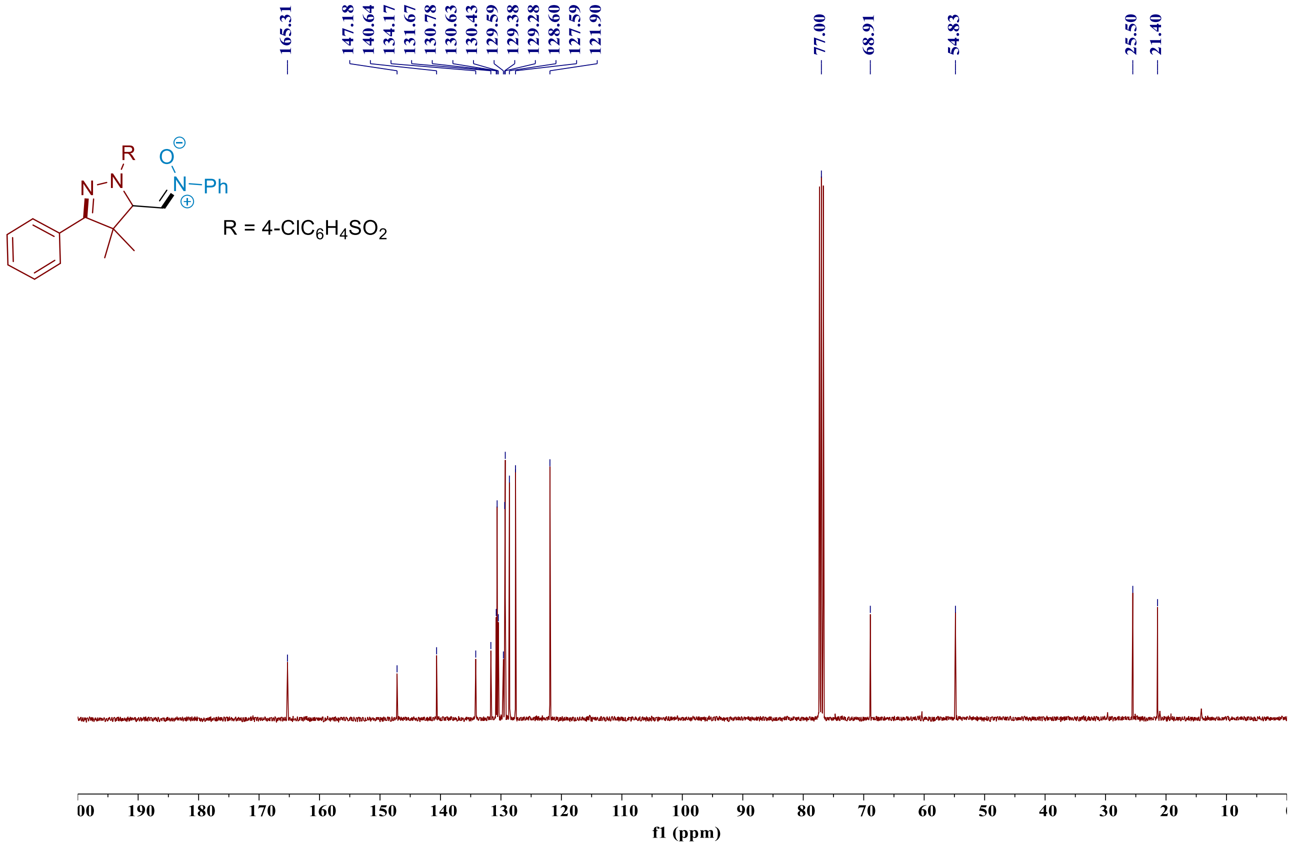

^{1}H NMR (400 MHz, $CDCl_3$), ^{13}C NMR (101 MHz, $CDCl_3$) of product 16

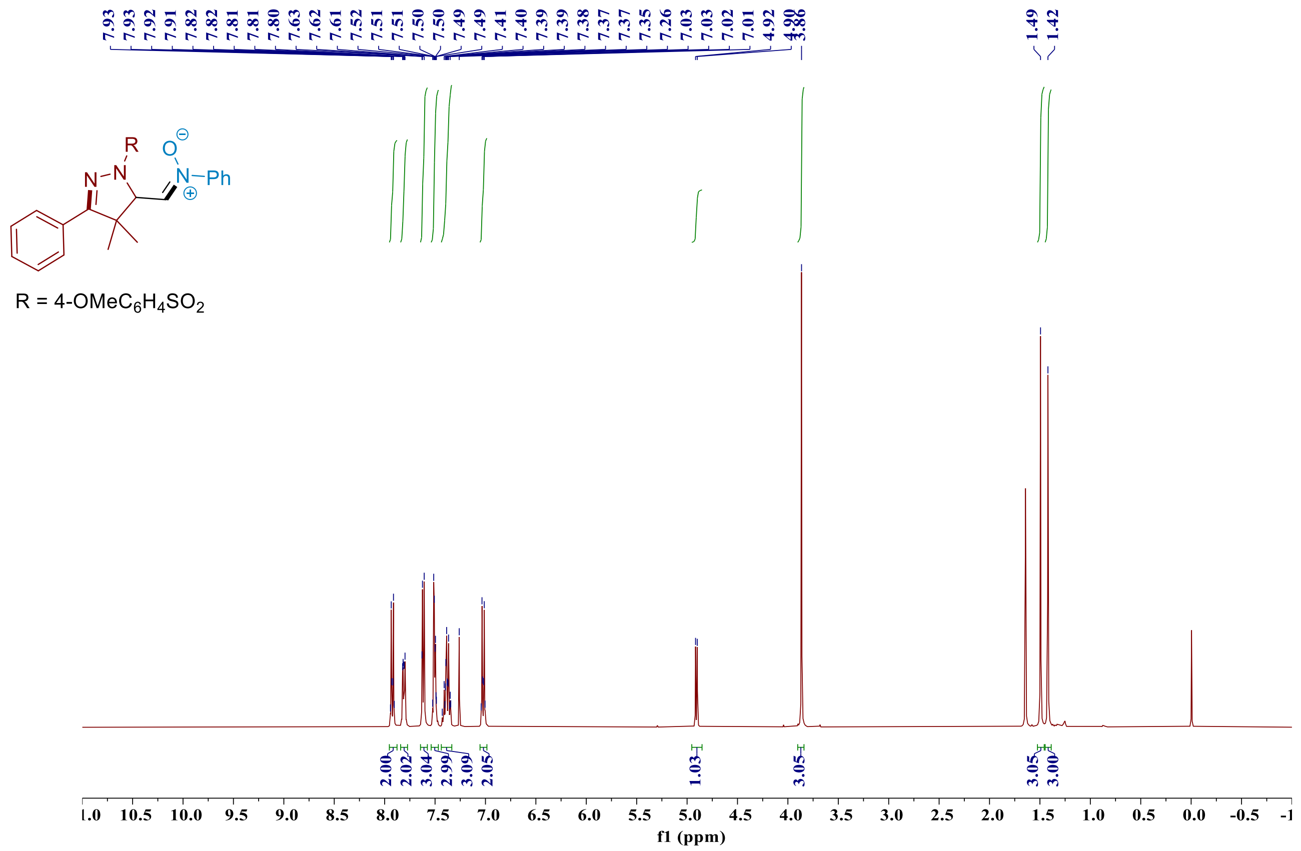

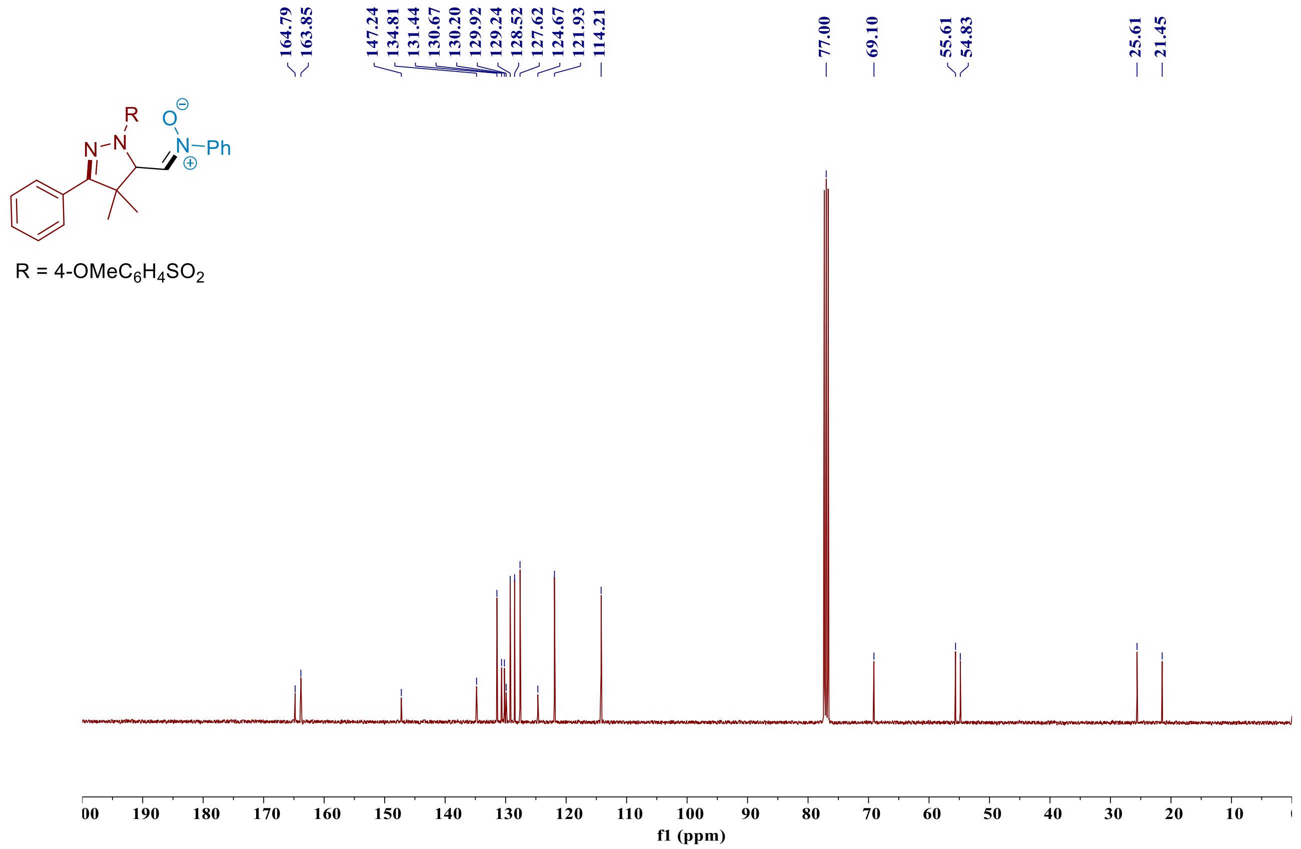

^{1}H NMR (400 MHz, $CDCl_3$), ^{13}C NMR (101 MHz, $CDCl_3$) of product 17

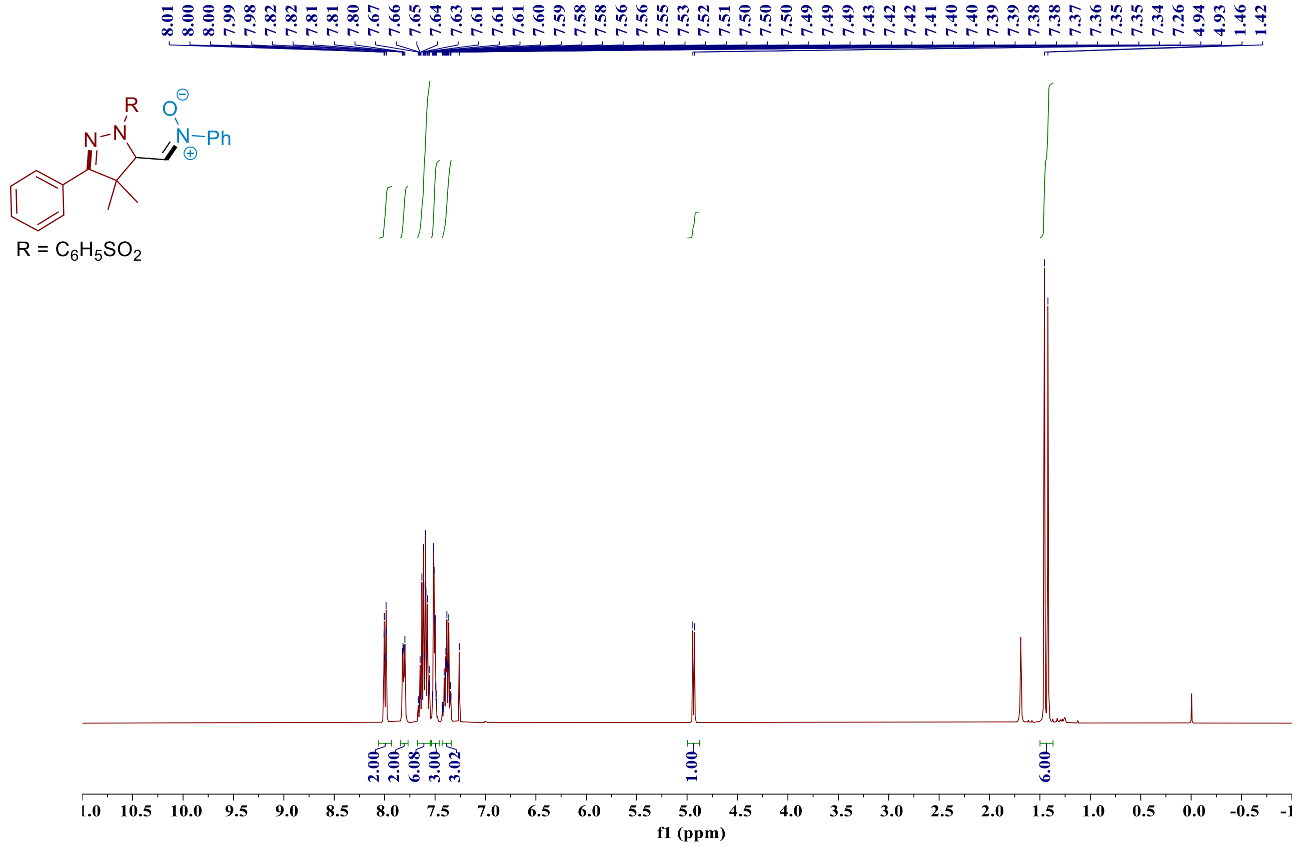

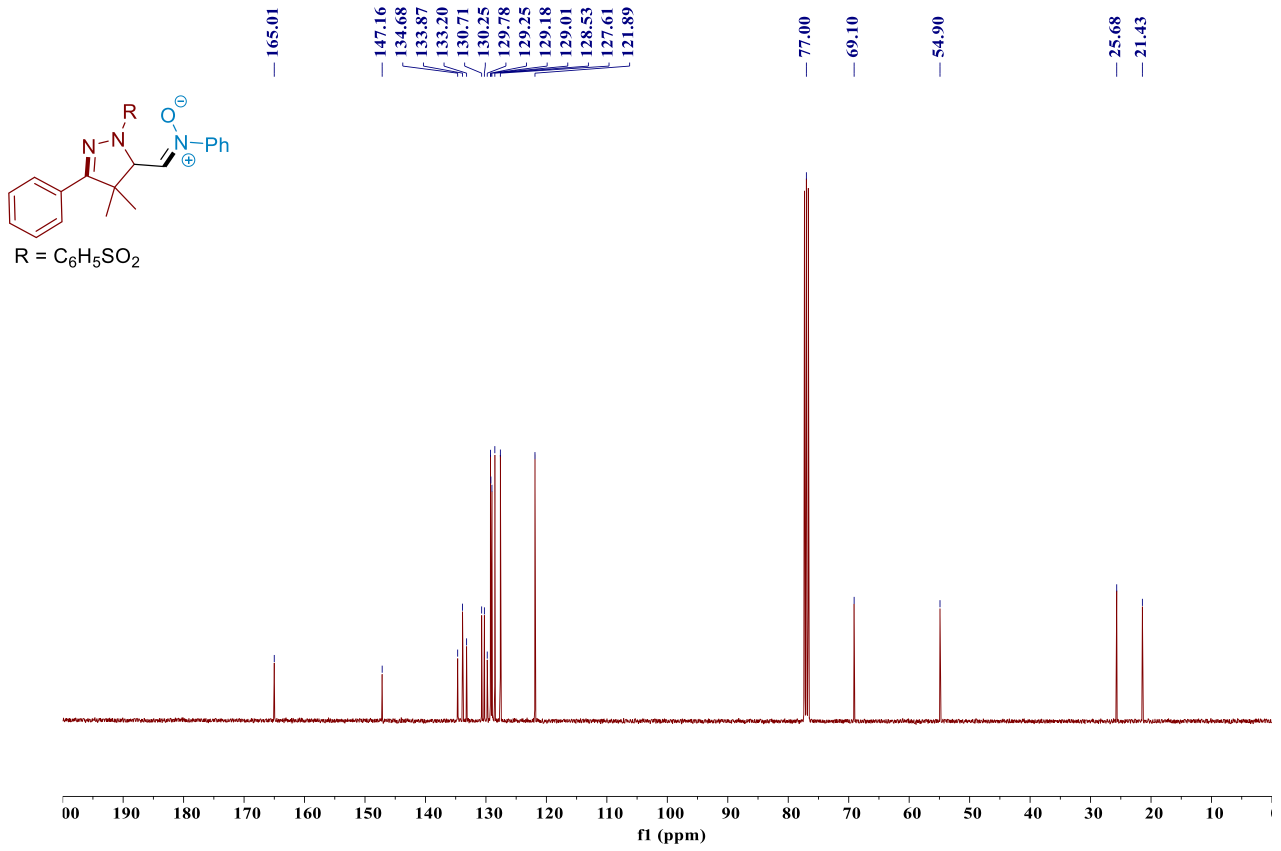

^{1}H NMR (400 MHz, $CDCl_3$), ^{13}C NMR (101 MHz, $CDCl_3$) of product 18

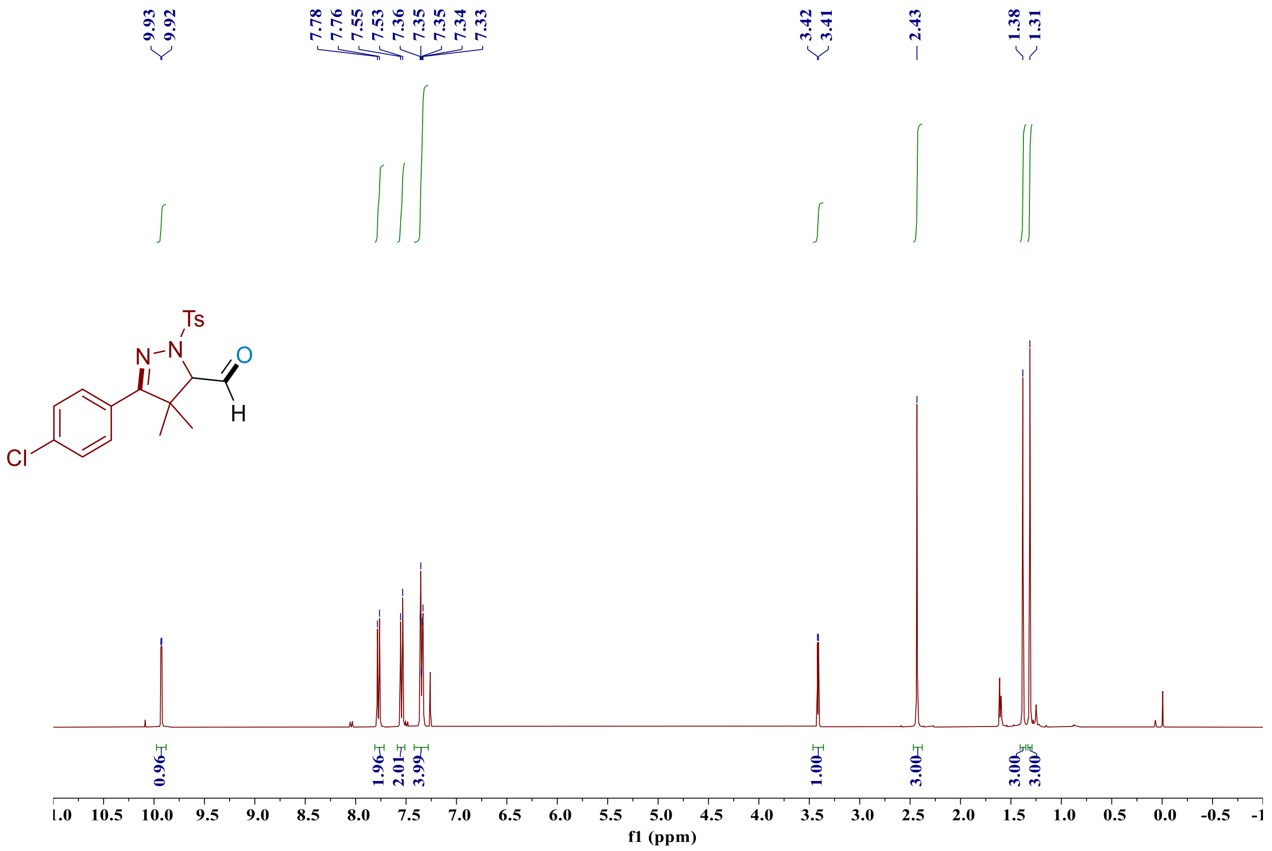

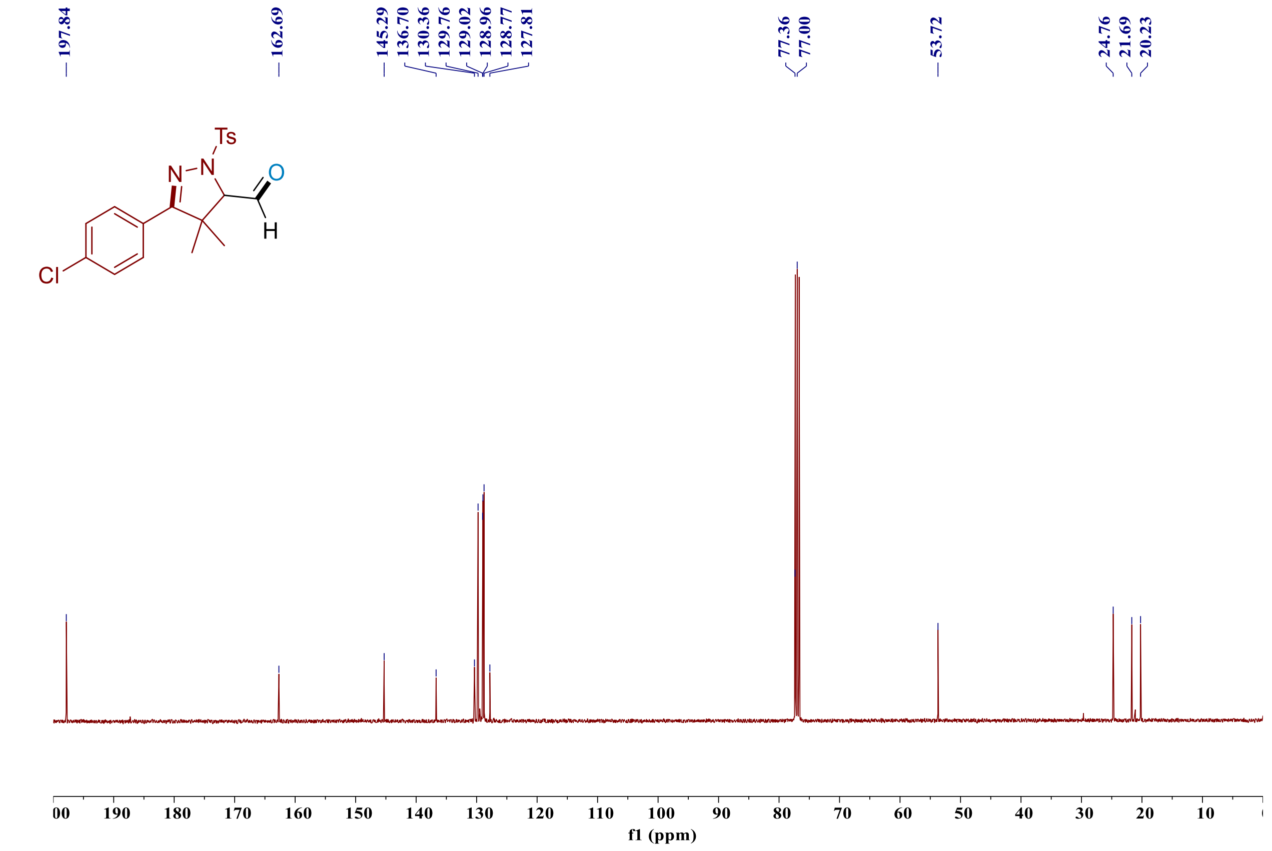

^{1}H NMR (400 MHz, $CDCl_3$), ^{13}C NMR (101 MHz, $CDCl_3$) of product 19

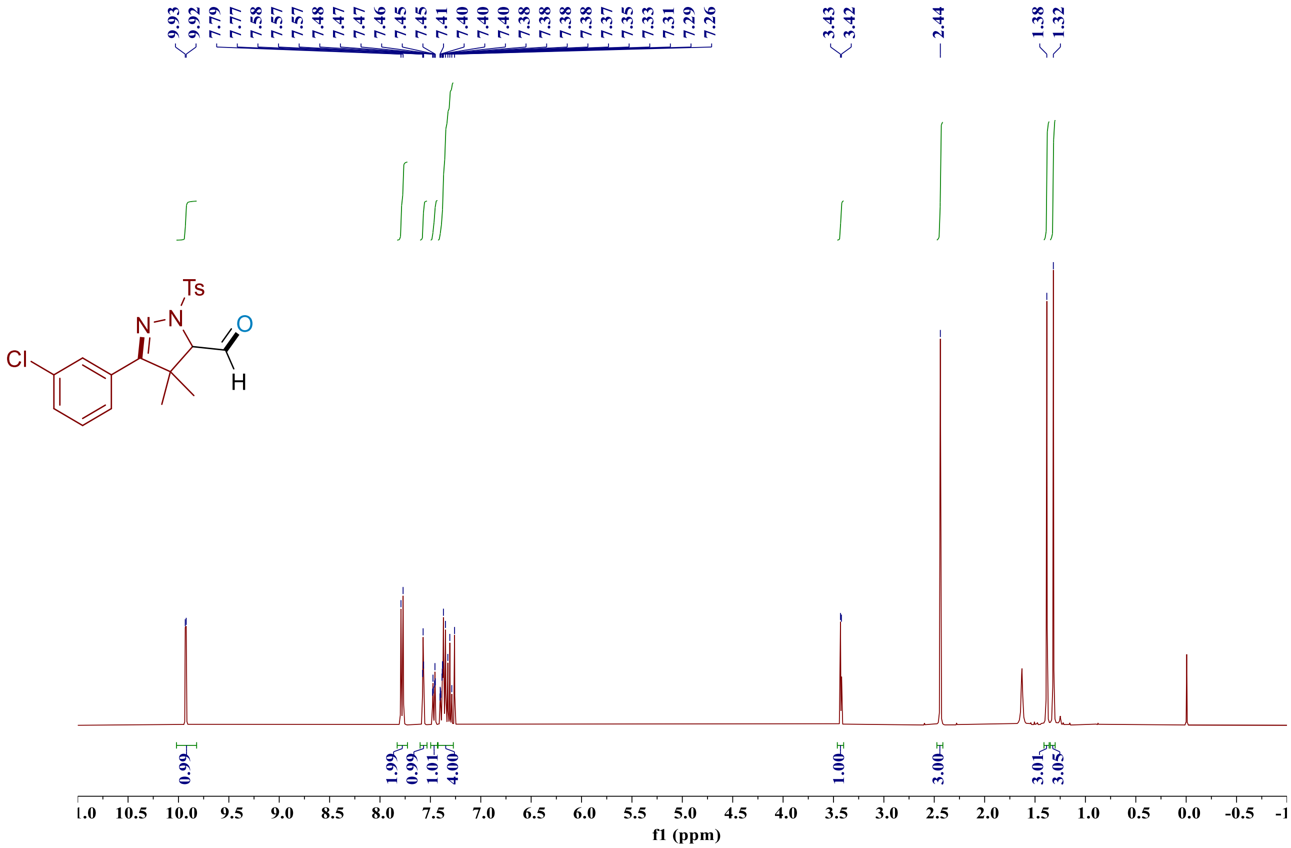

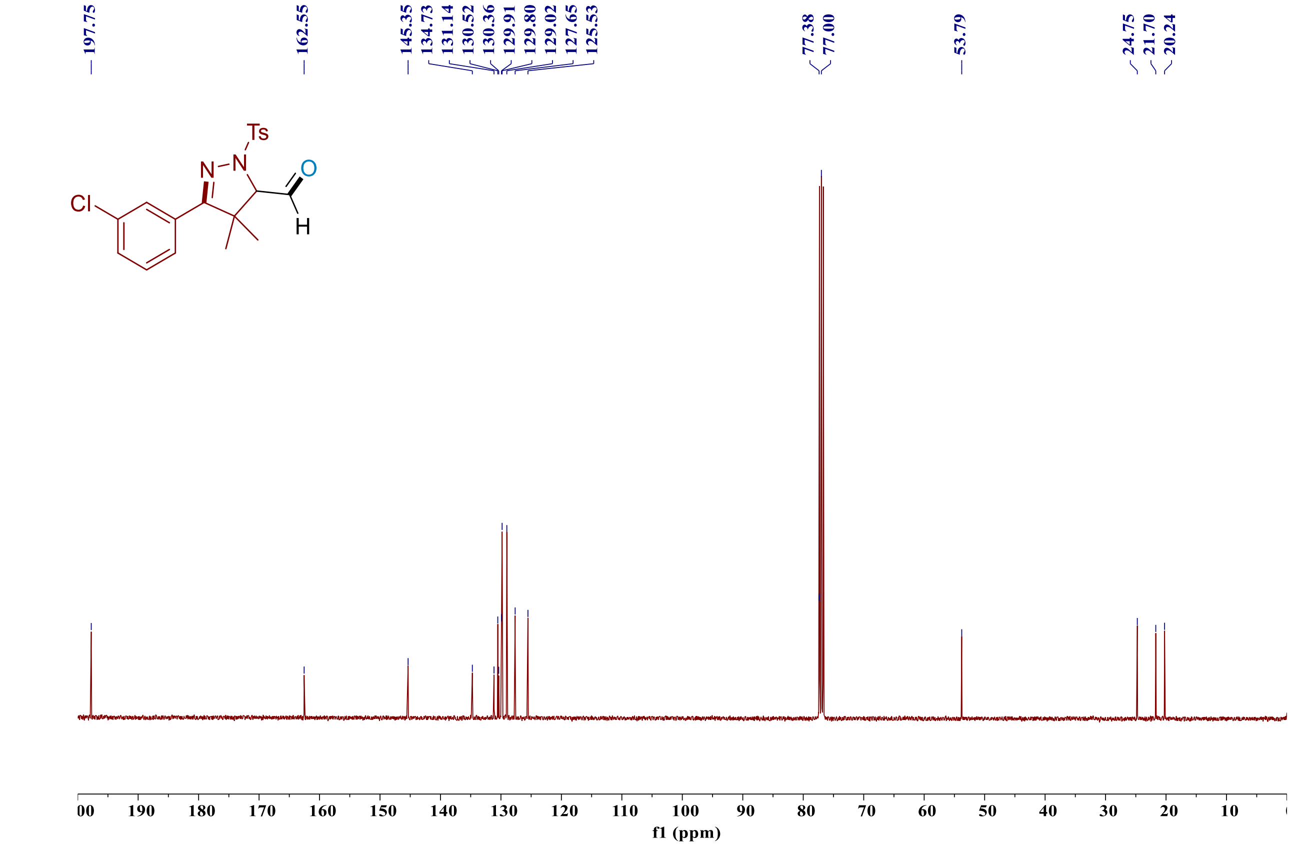

^{1}H NMR (400 MHz, $CDCl_3$), ^{13}C NMR (101 MHz, $CDCl_3$) of product 20

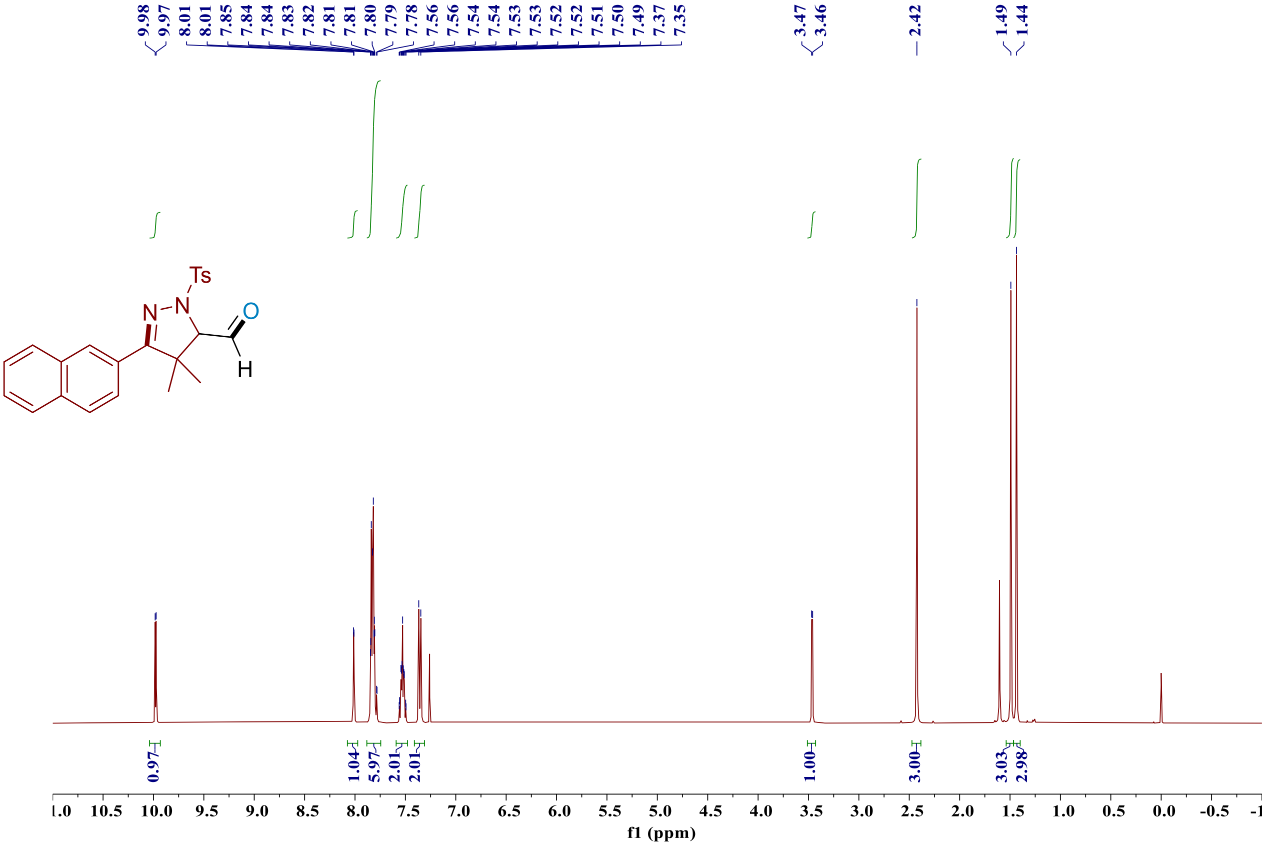

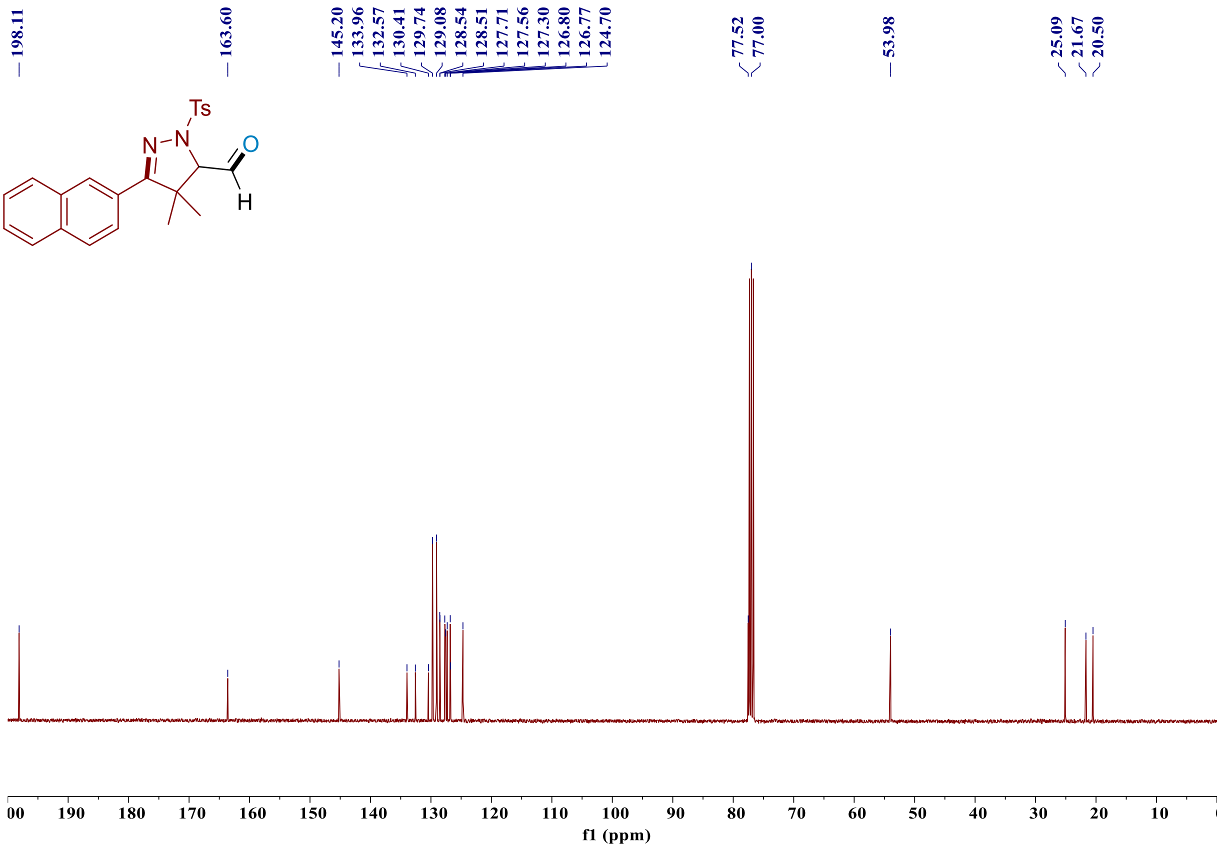

^{1}H NMR (400 MHz, $CDCl_3$), ^{13}C NMR (101 MHz, $CDCl_3$) of product 21

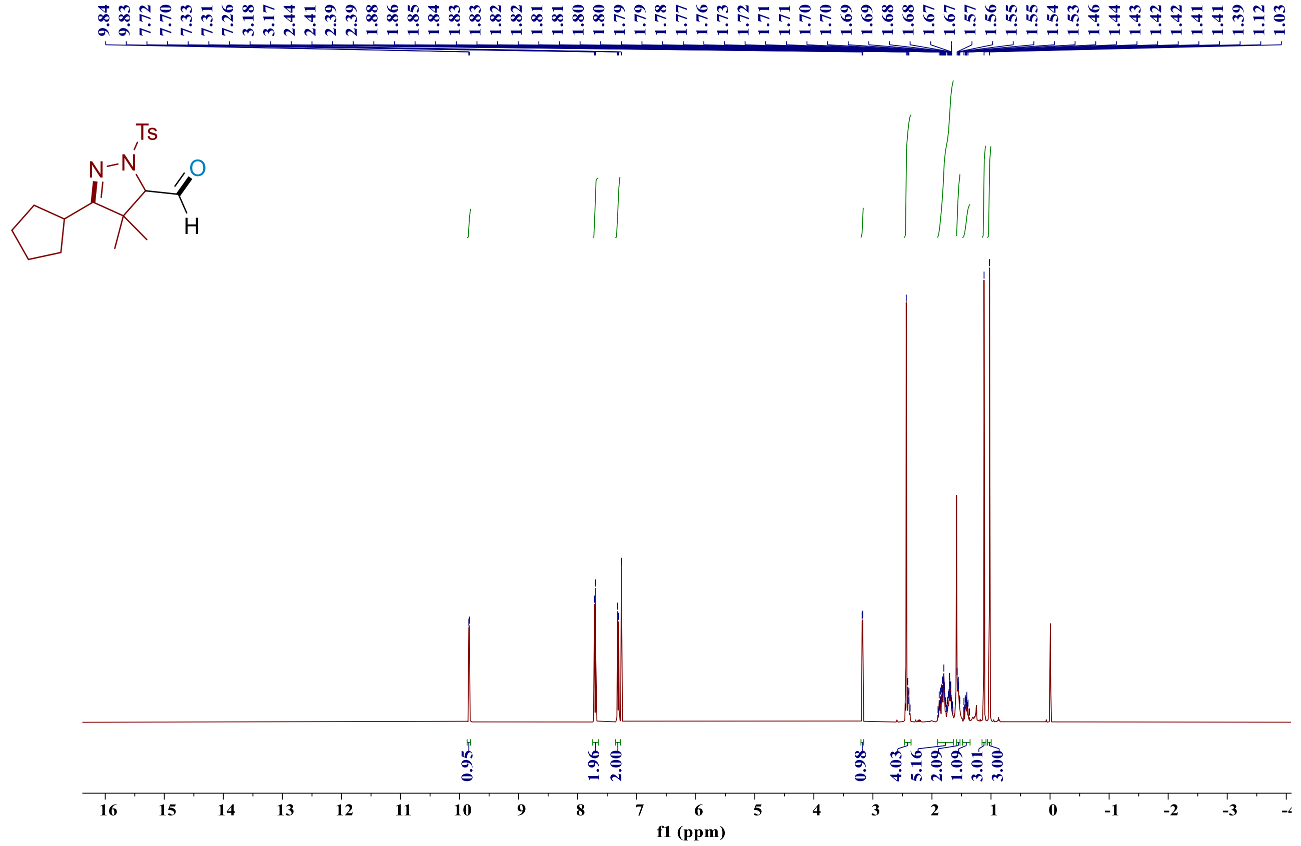

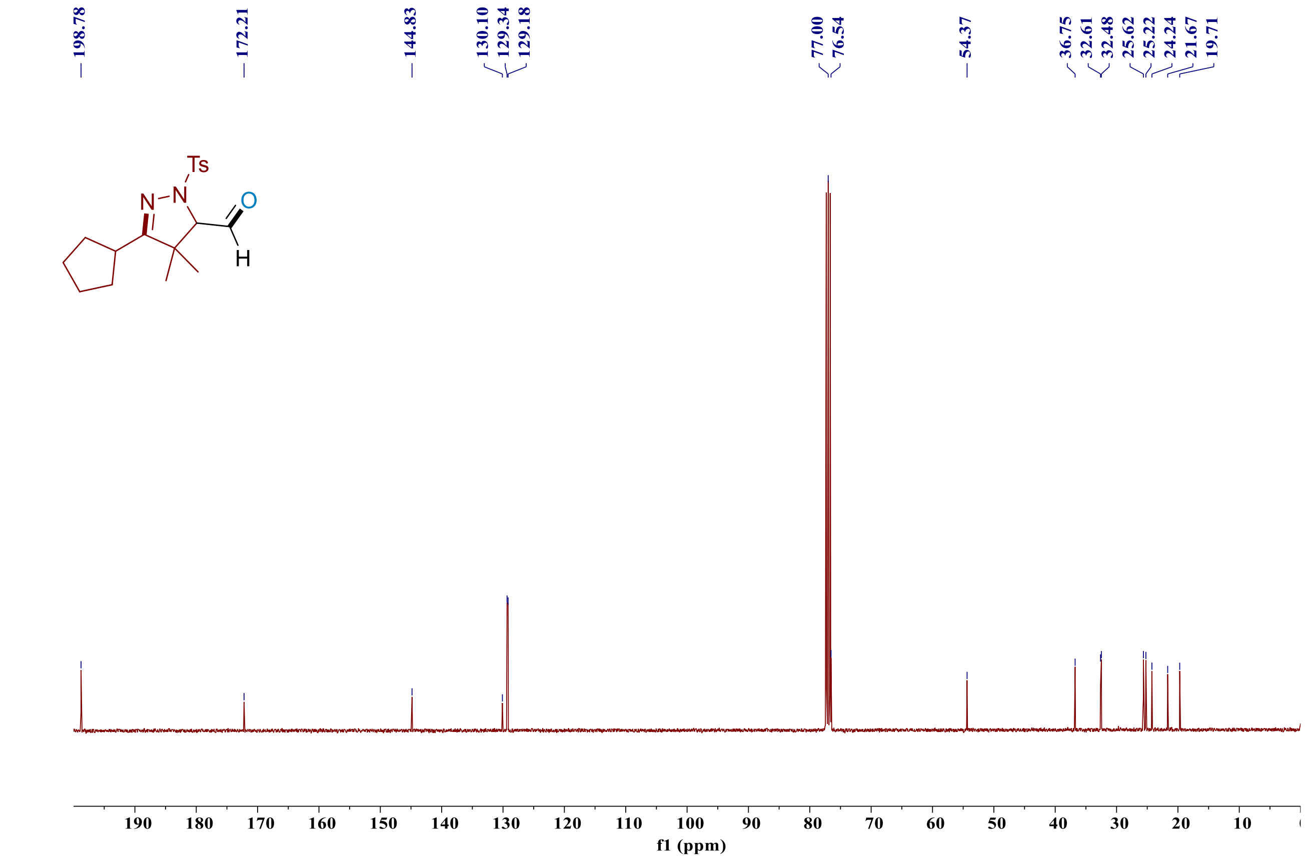

^{1}H NMR (400 MHz, CDCl$_3$), ^{13}C NMR (101 MHz, CDCl$_3$) of product 23

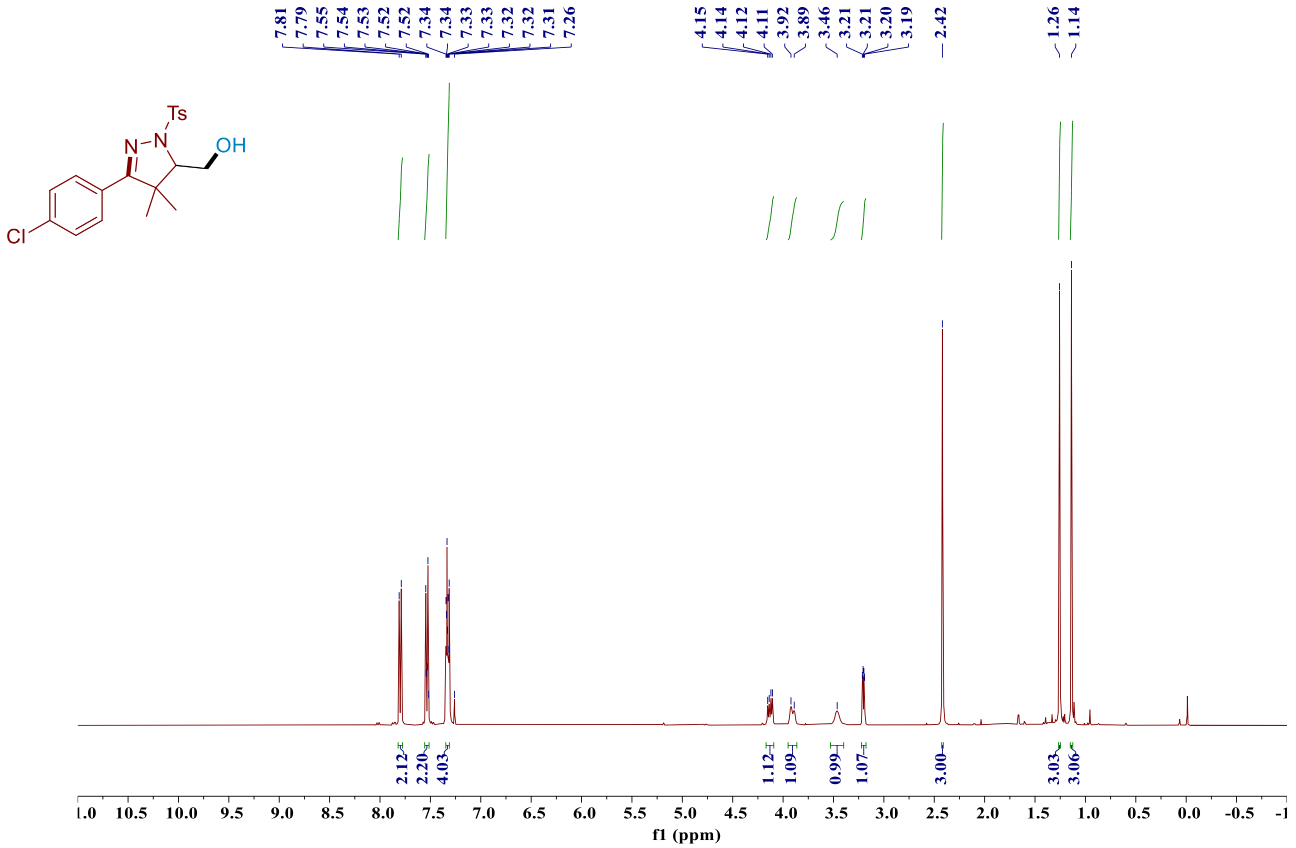

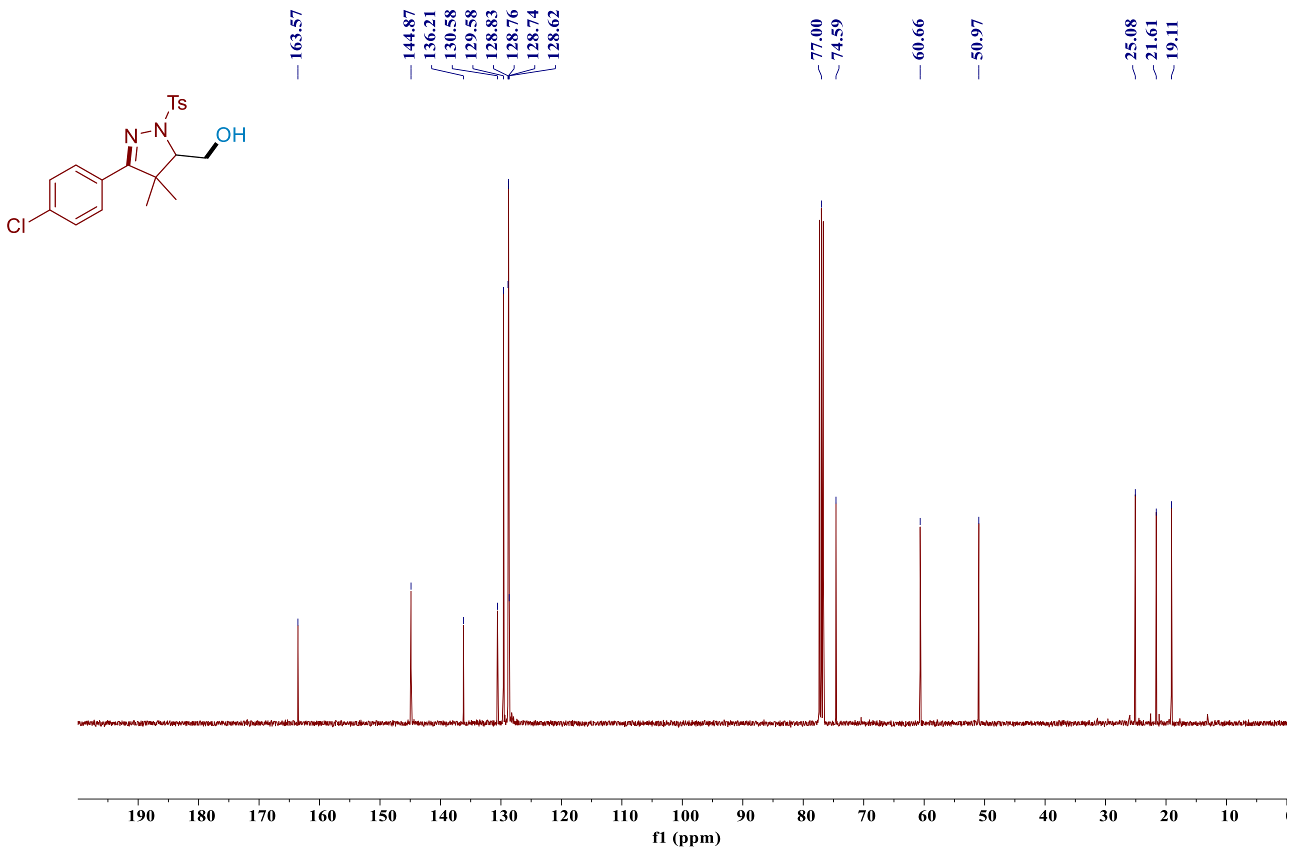

^{1}H NMR (400 MHz, CDCl$_3$), ^{13}C NMR (101 MHz, CDCl$_3$) of product 24

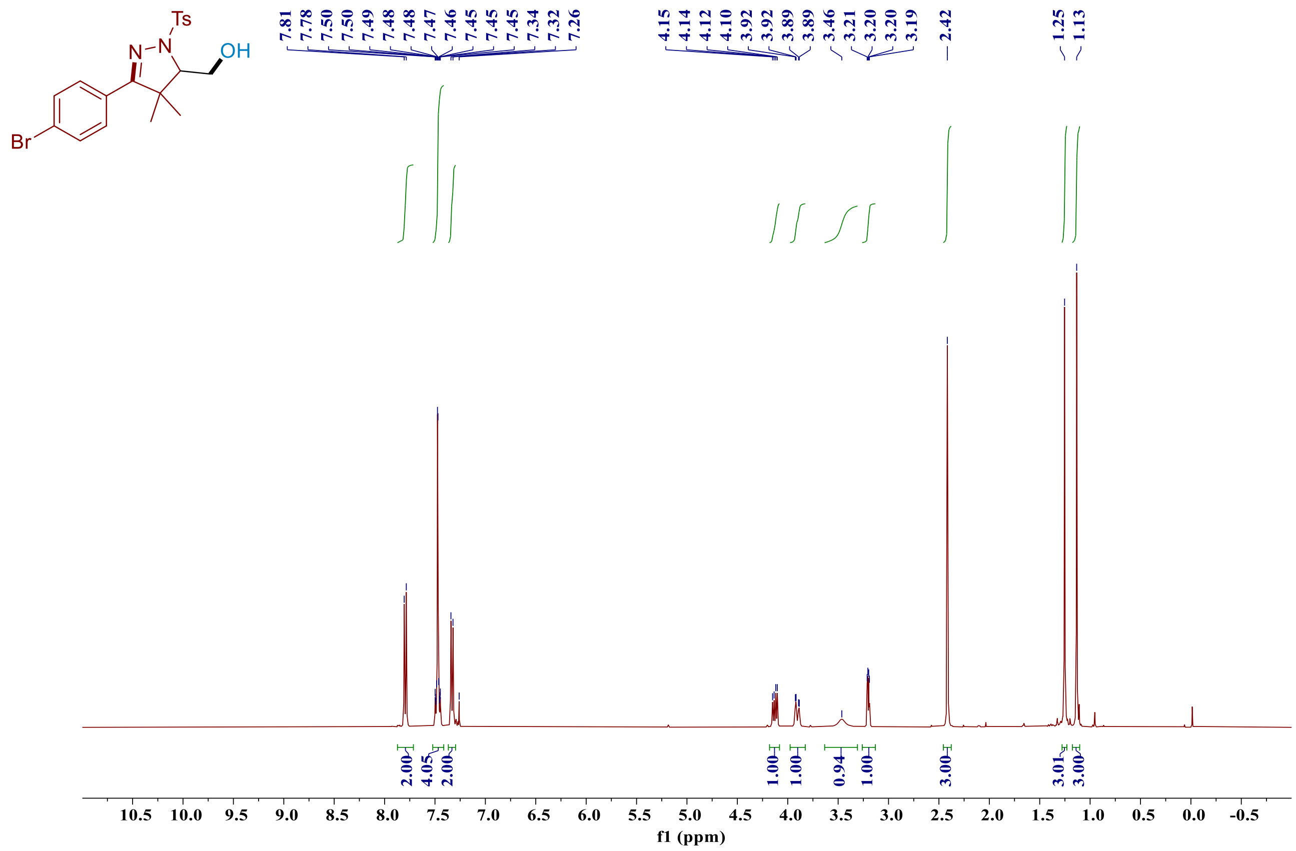

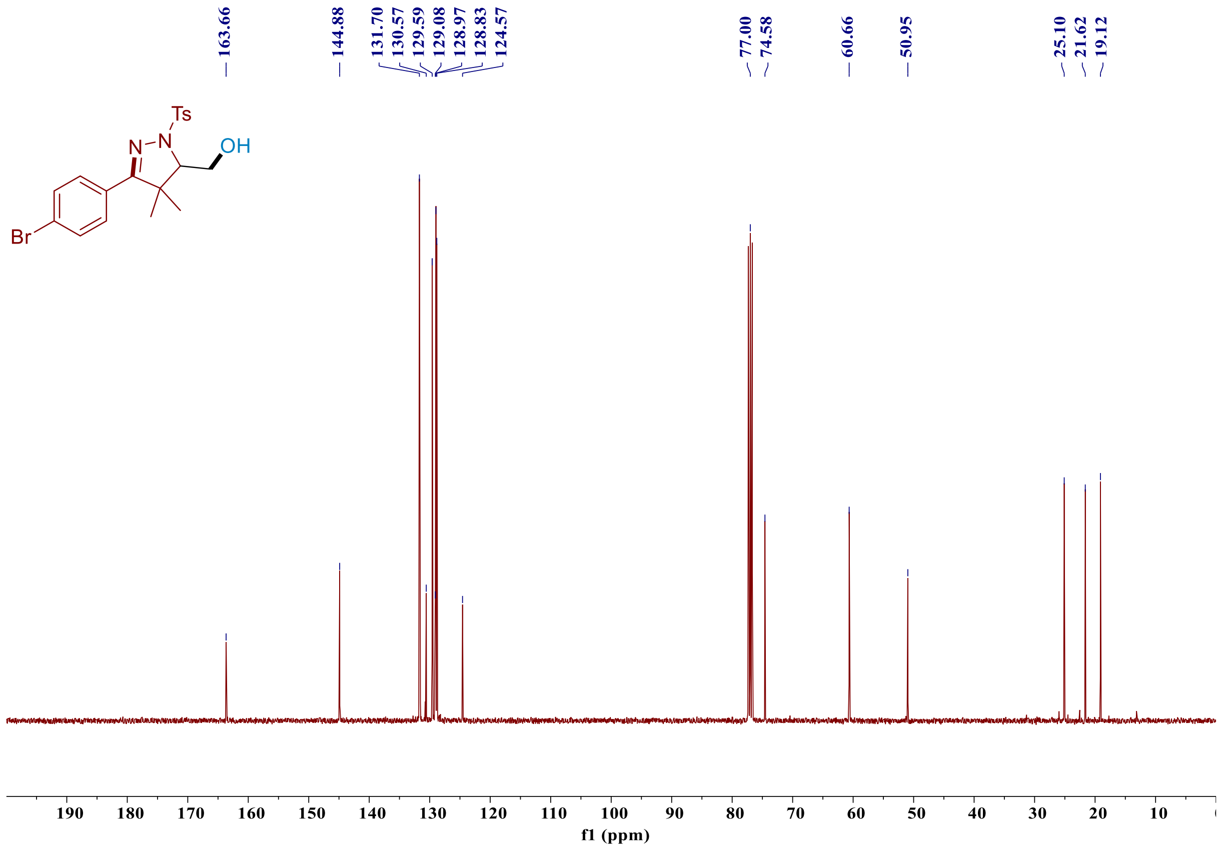

^{1}H NMR (400 MHz, CDCl$_3$), ^{13}C NMR (101 MHz, CDCl$_3$) of product 25

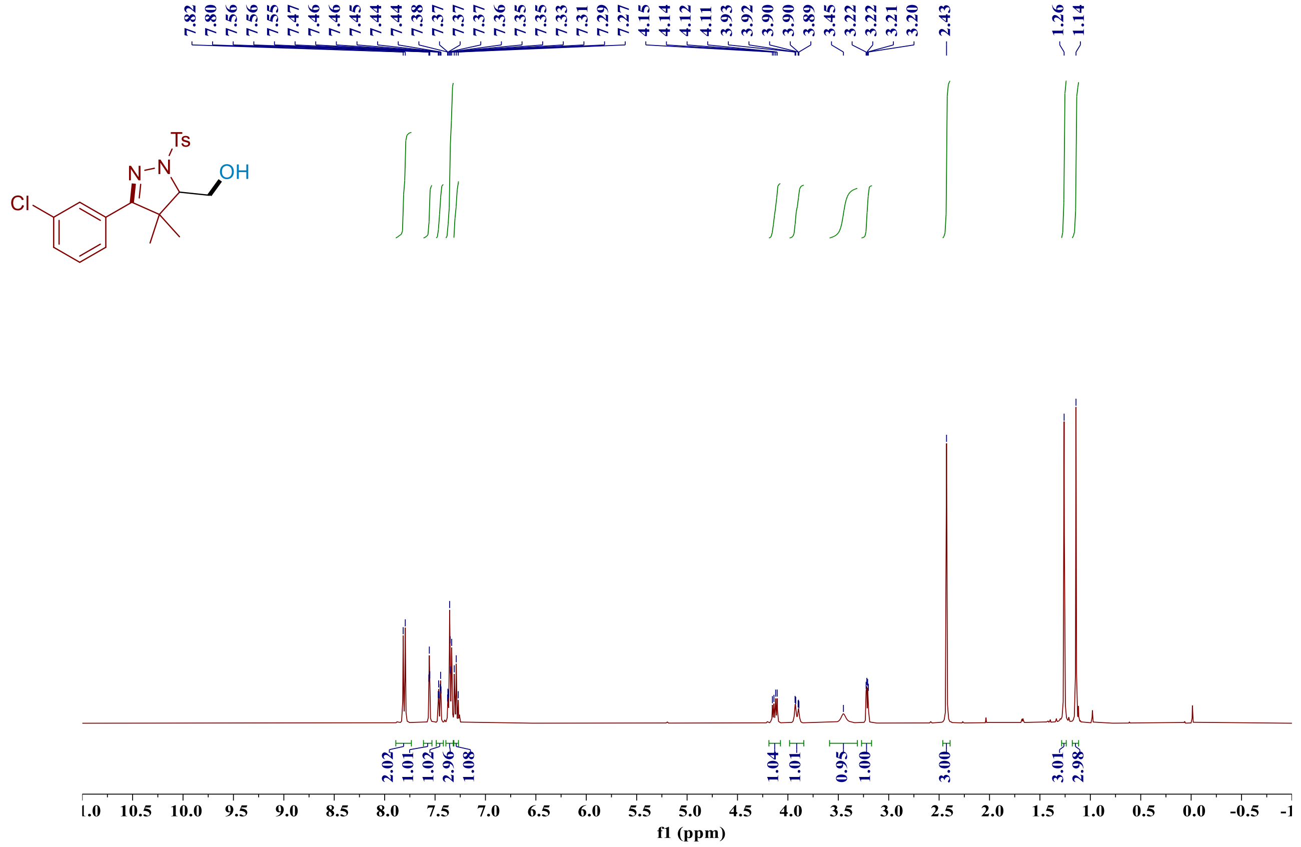

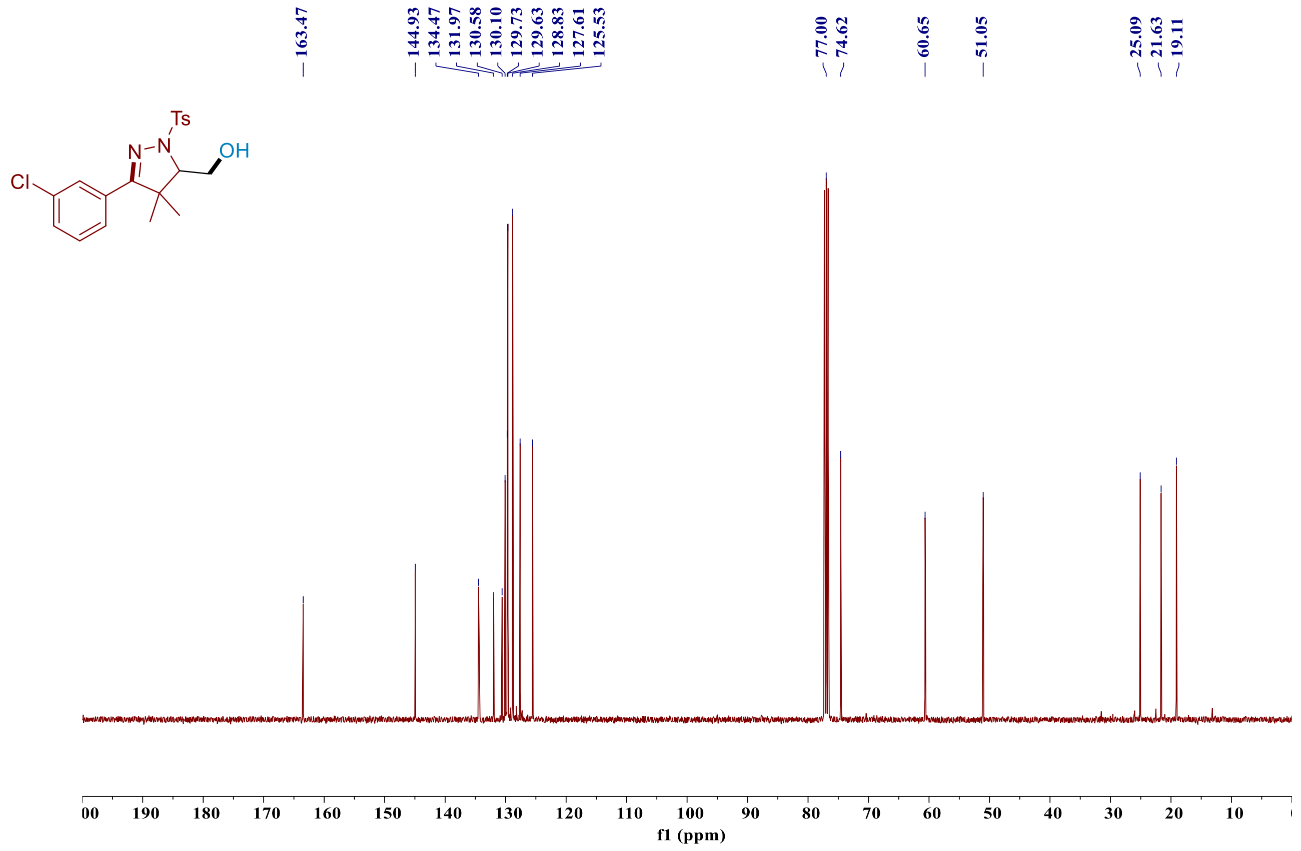

^{1}H NMR (400 MHz, $CDCl_3$), ^{13}C NMR (101 MHz, $CDCl_3$) of product 26

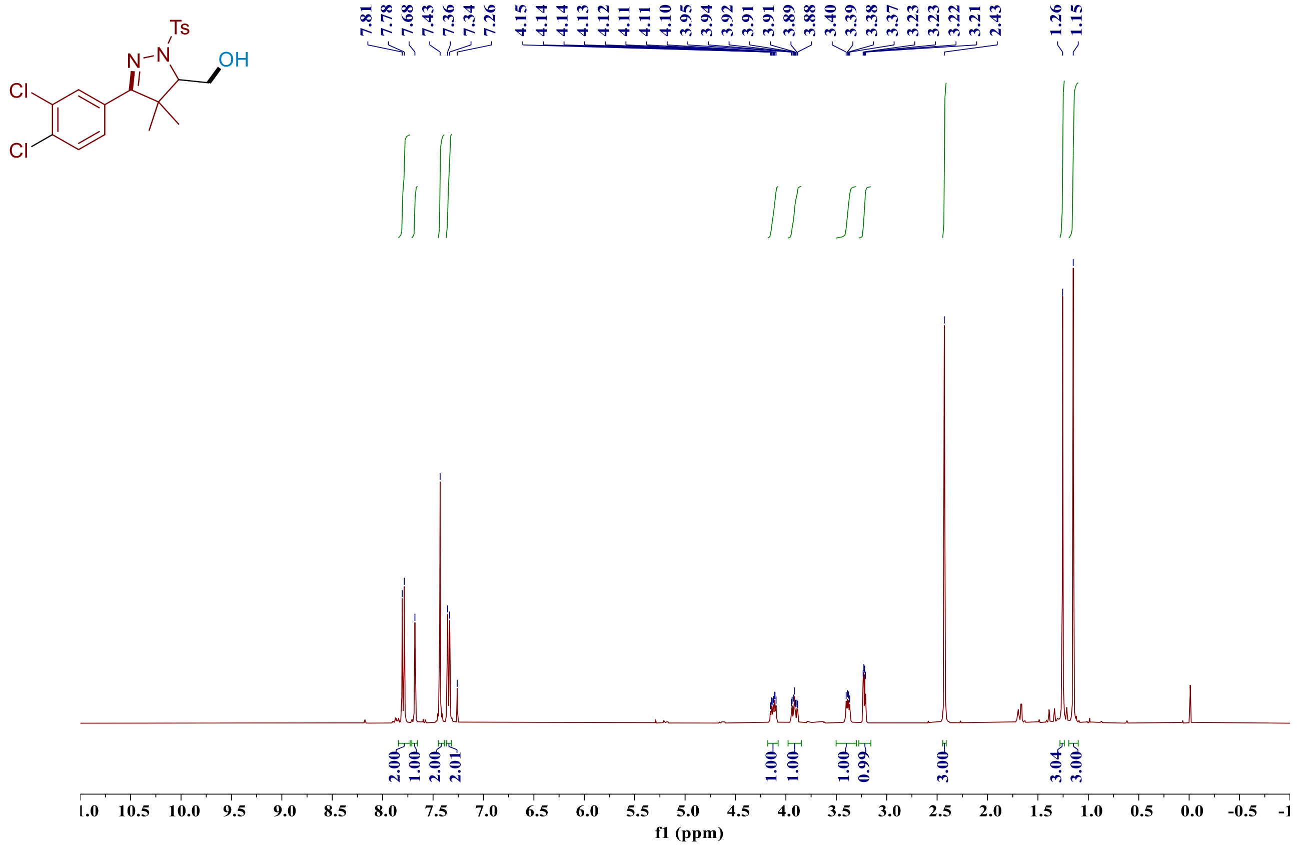

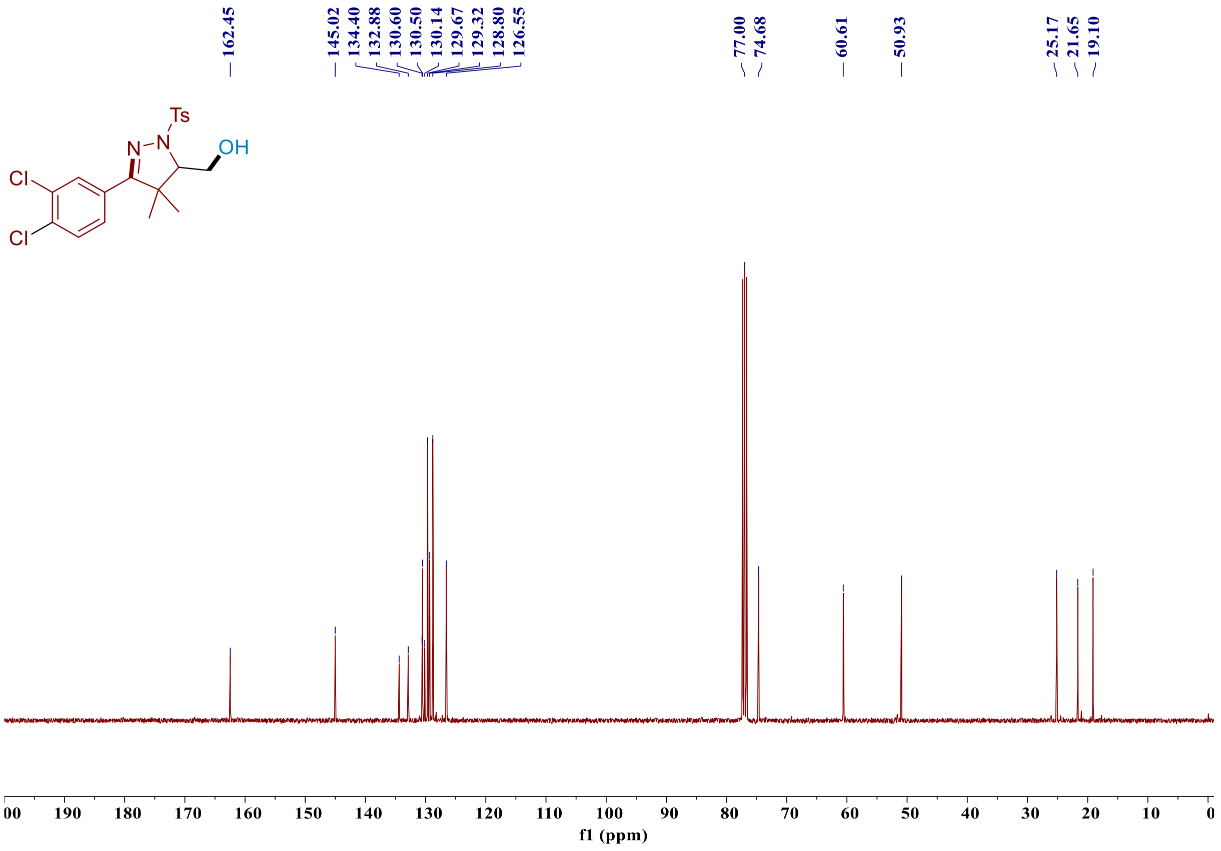

^{1}H NMR (400 MHz, CDCl$_3$), ^{13}C NMR (101 MHz, CDCl$_3$) of product 27

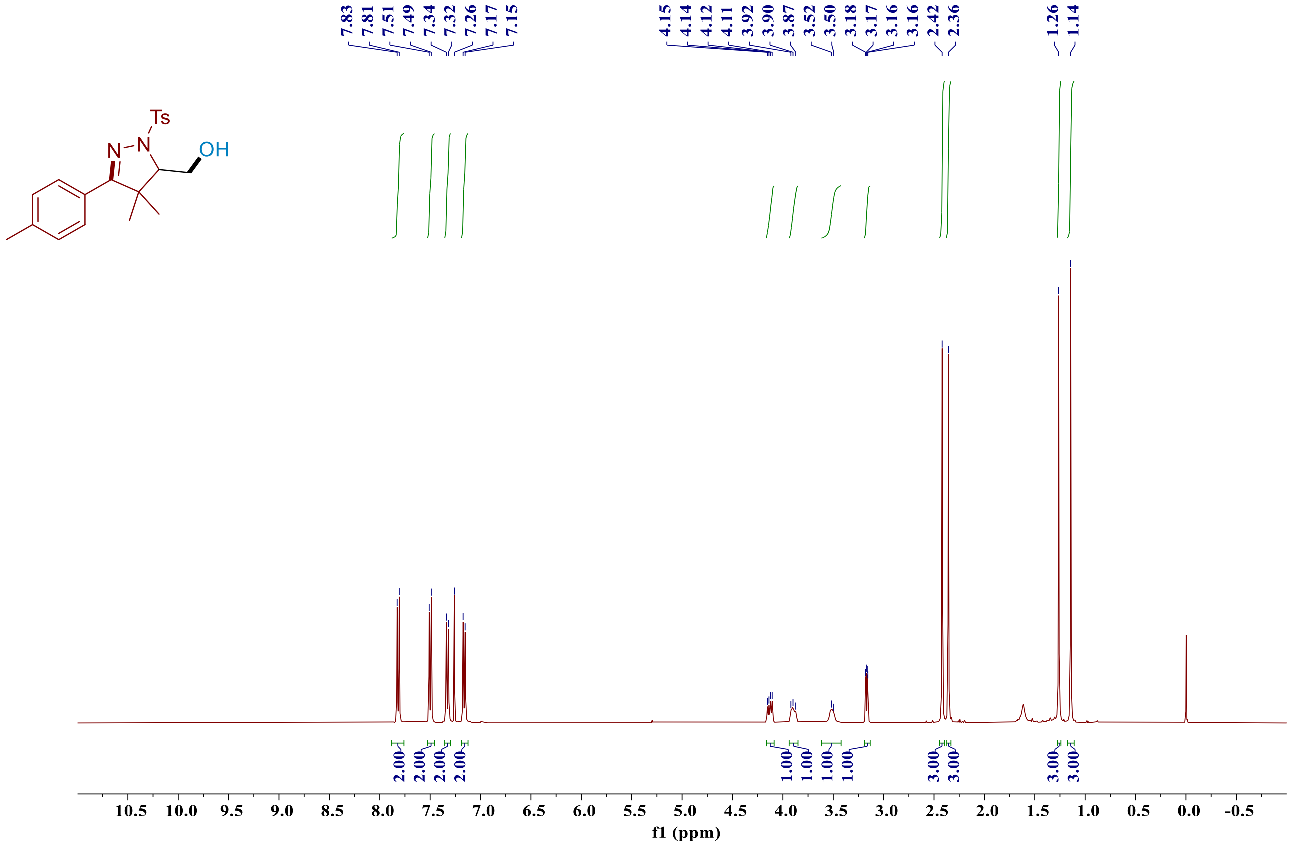

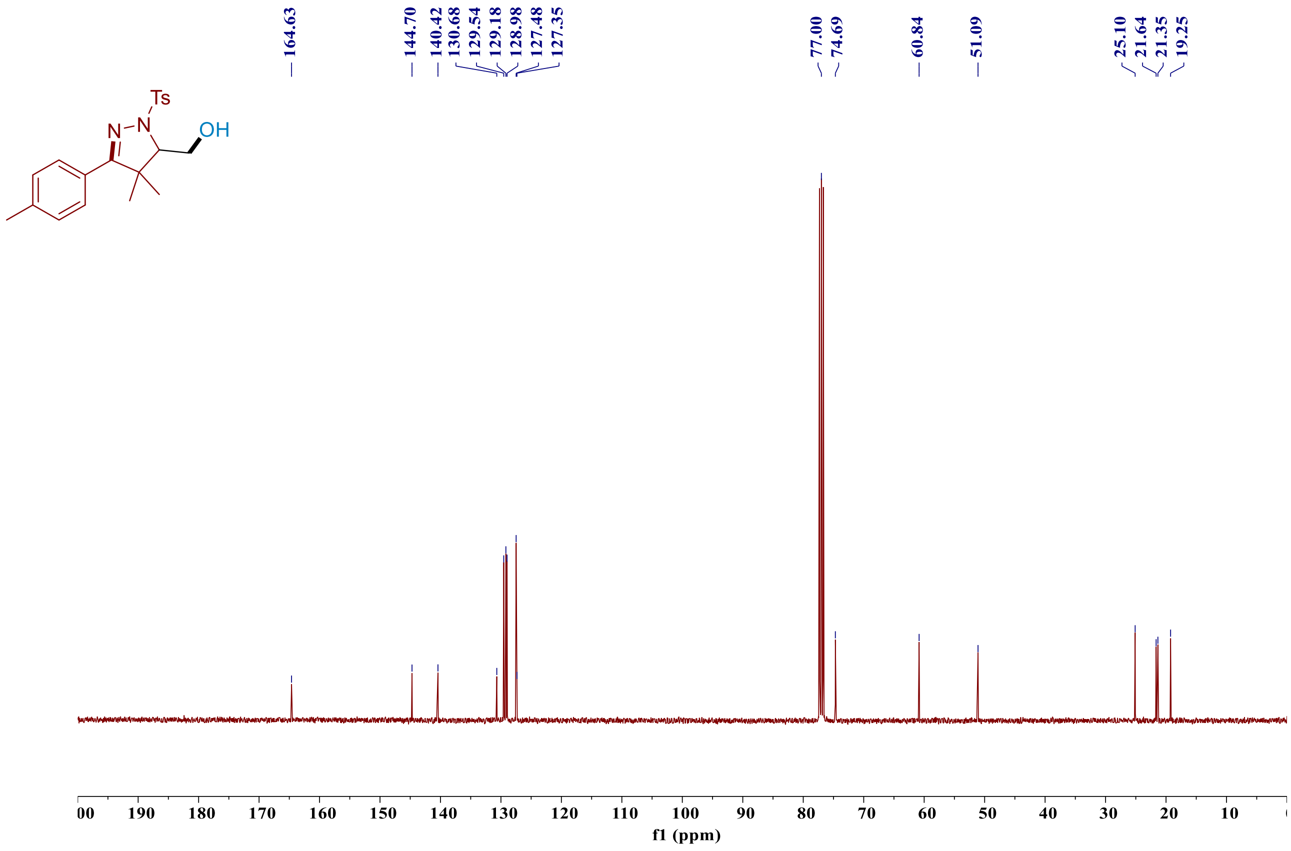

^{1}H NMR (400 MHz, CDCl$_3$), ^{13}C NMR (101 MHz, CDCl$_3$) of product 28

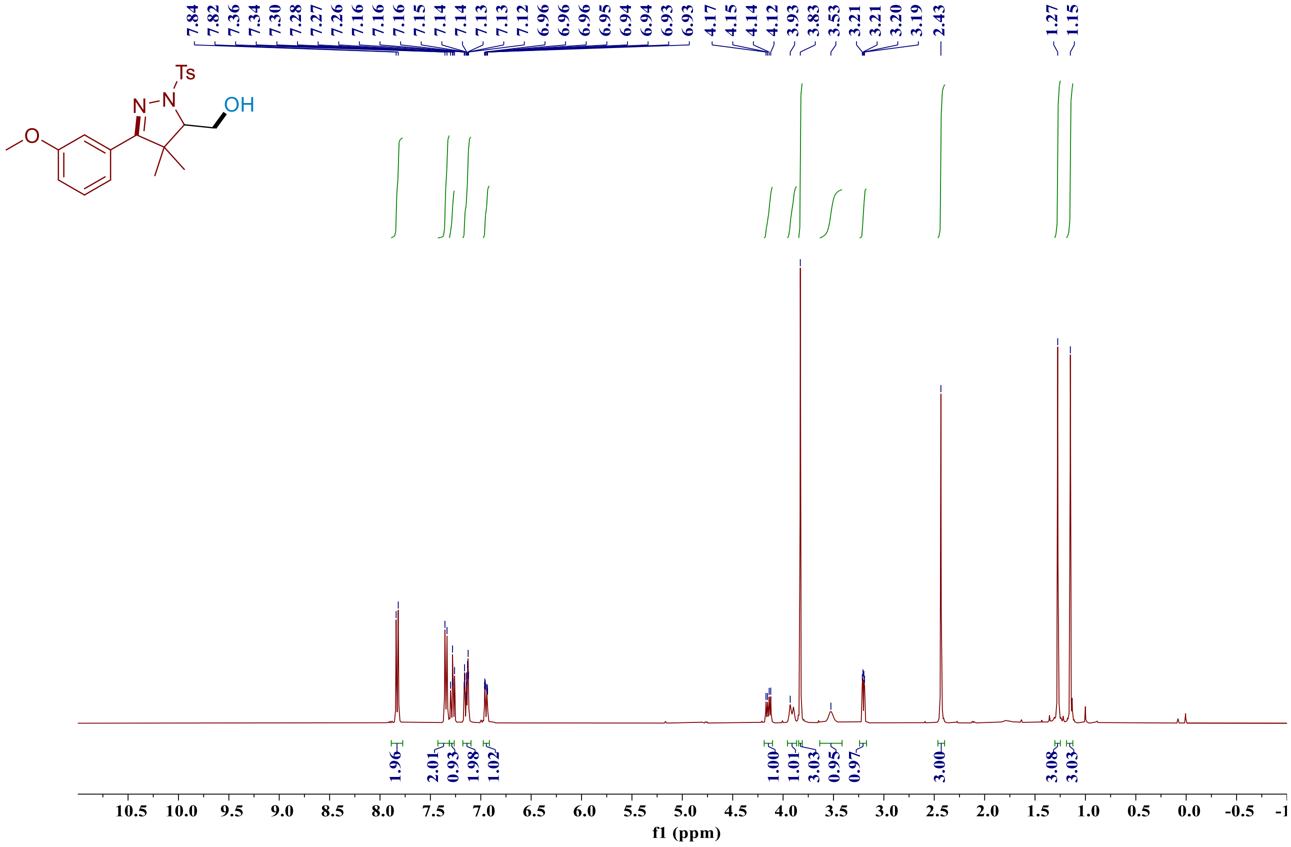

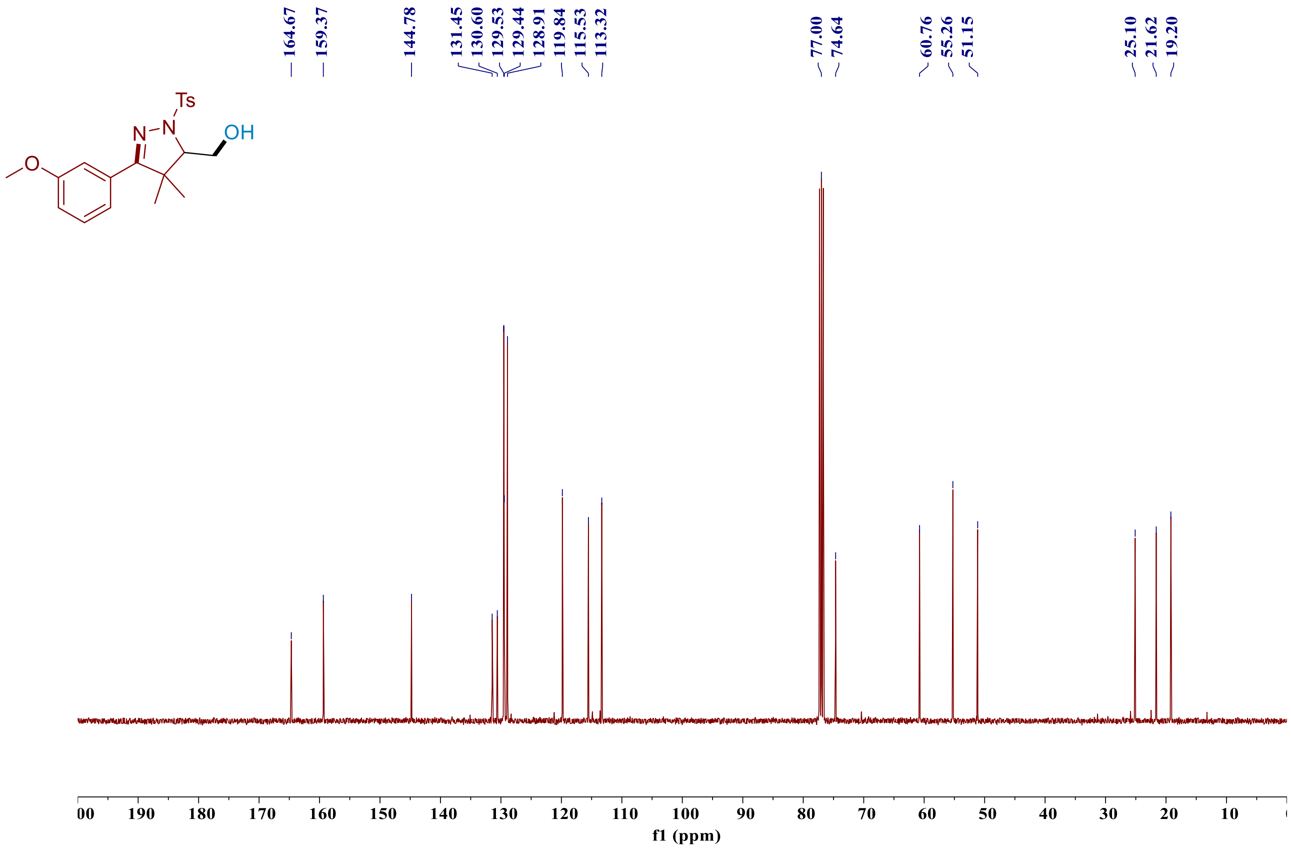

^{1}H NMR (400 MHz, CDCl$_3$), ^{13}C NMR (101 MHz, CDCl$_3$) of product 29

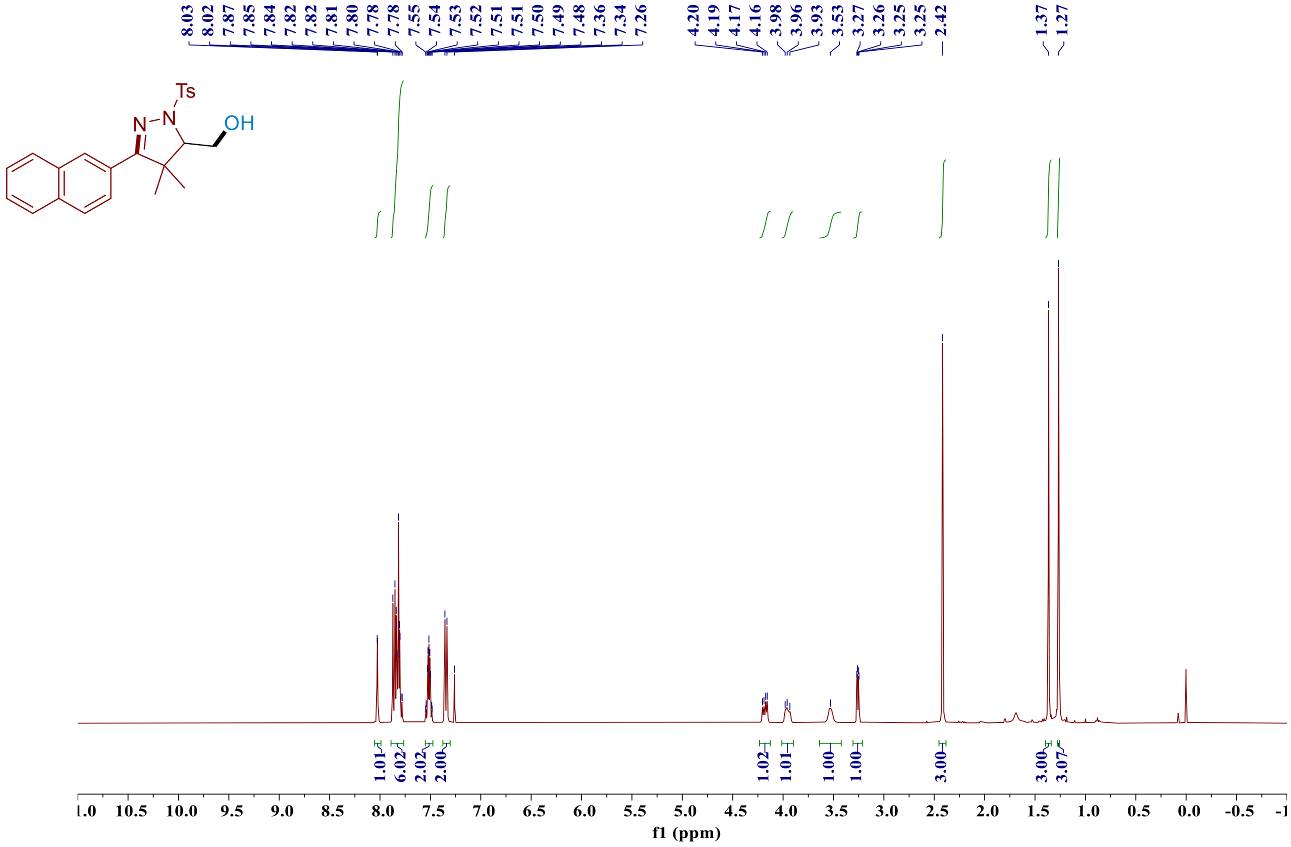

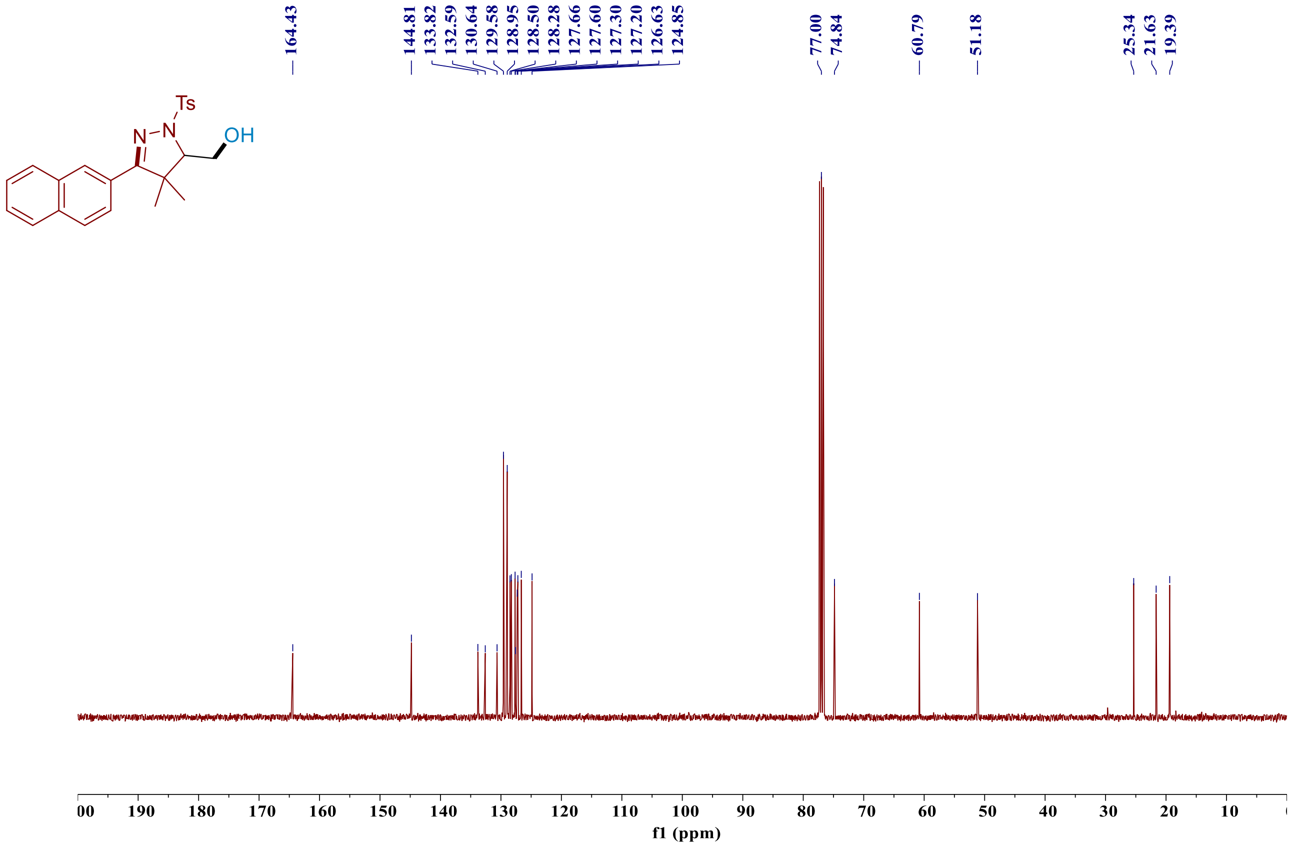

^{1}H NMR (400 MHz, $CDCl_3$), ^{13}C NMR (101 MHz, $CDCl_3$) of product 30

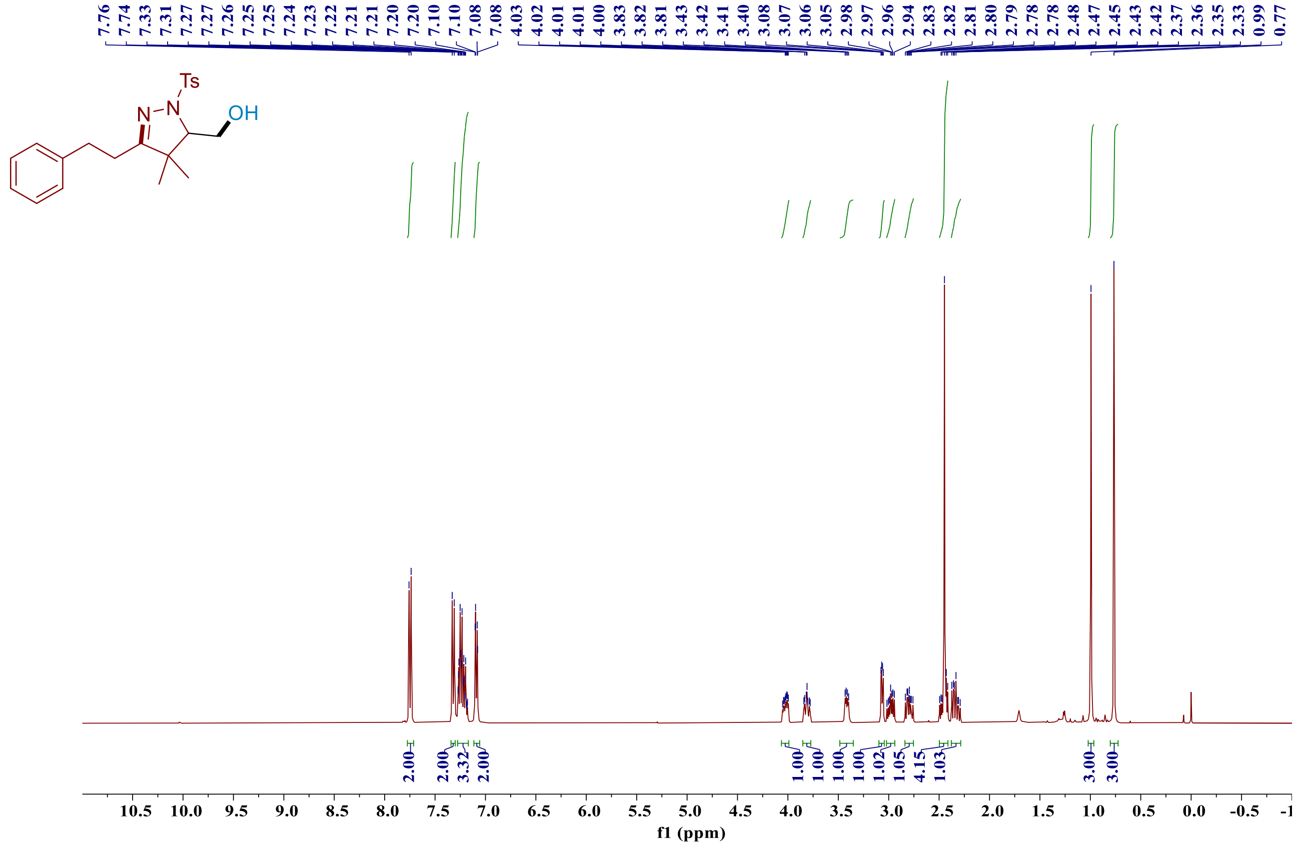

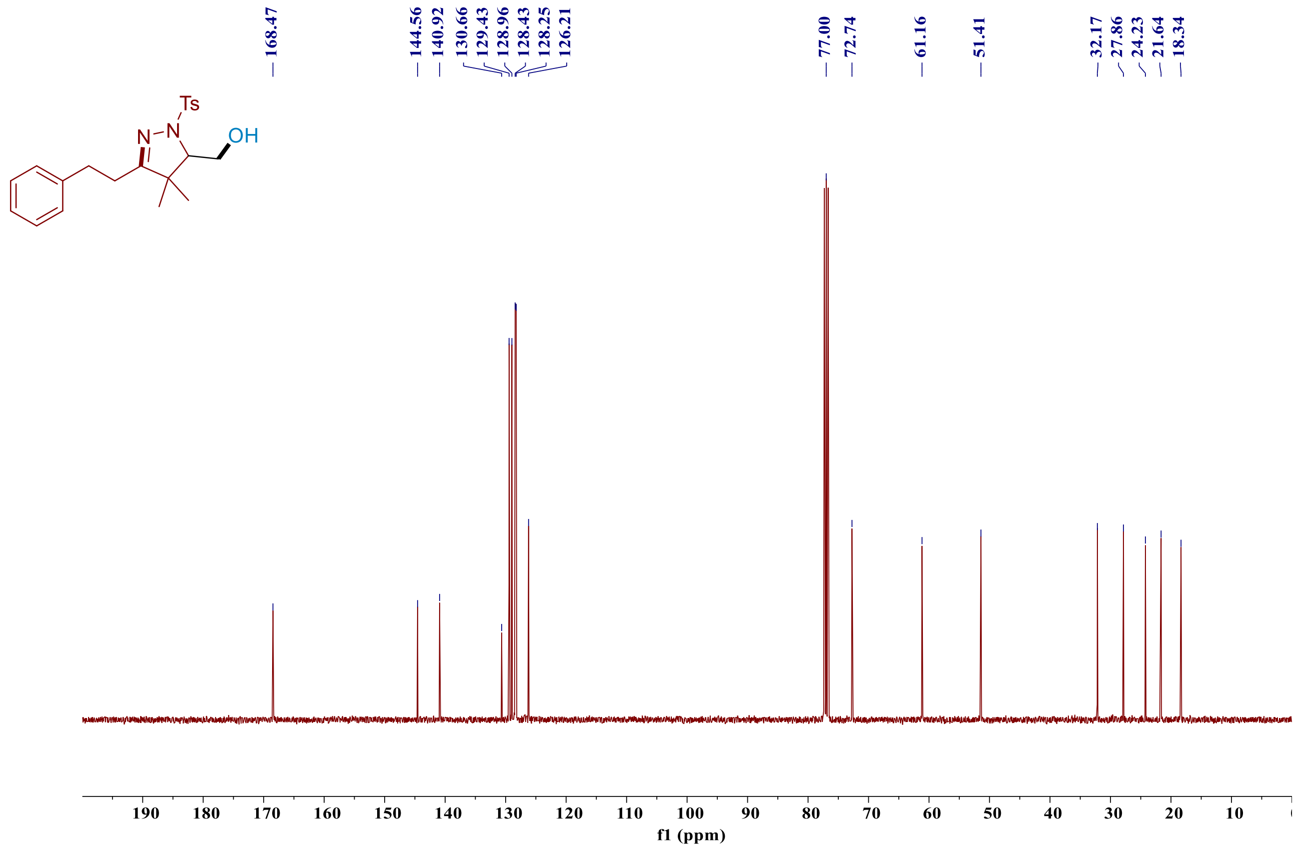

^{1}H NMR (400 MHz, $CDCl_3$), ^{13}C NMR (101 MHz, $CDCl_3$) of product 31

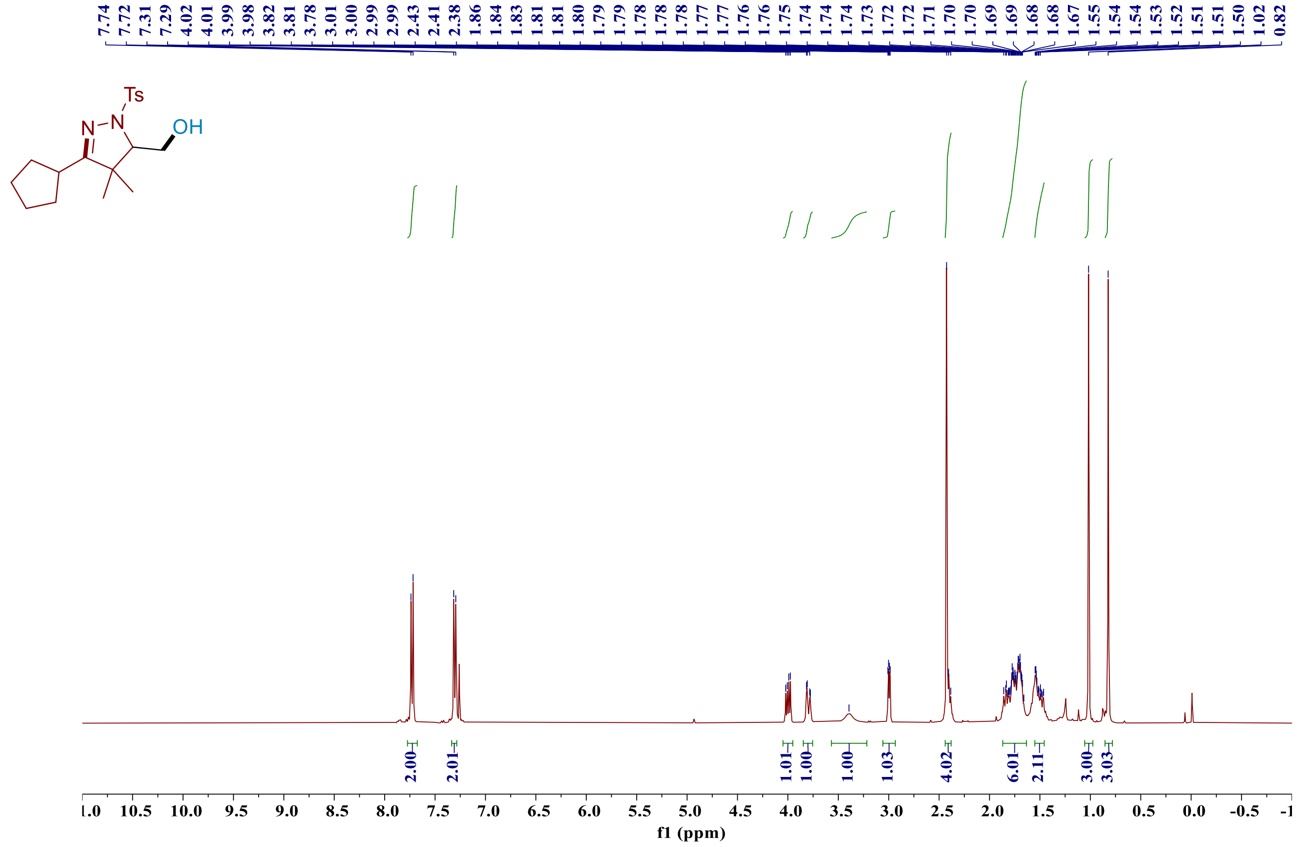

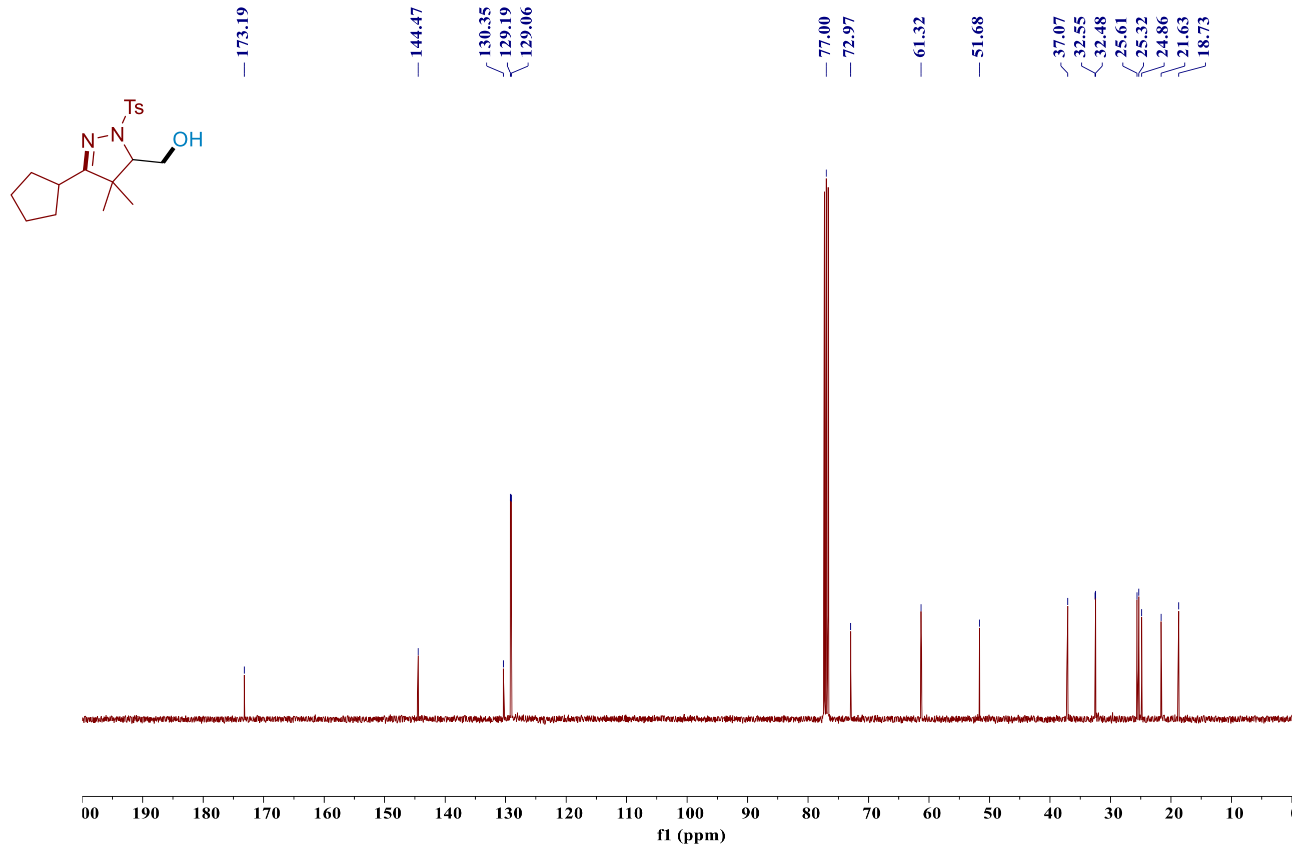

^{1}H NMR (400 MHz, $CDCl_3$), ^{13}C NMR (101 MHz, $CDCl_3$) of product 32

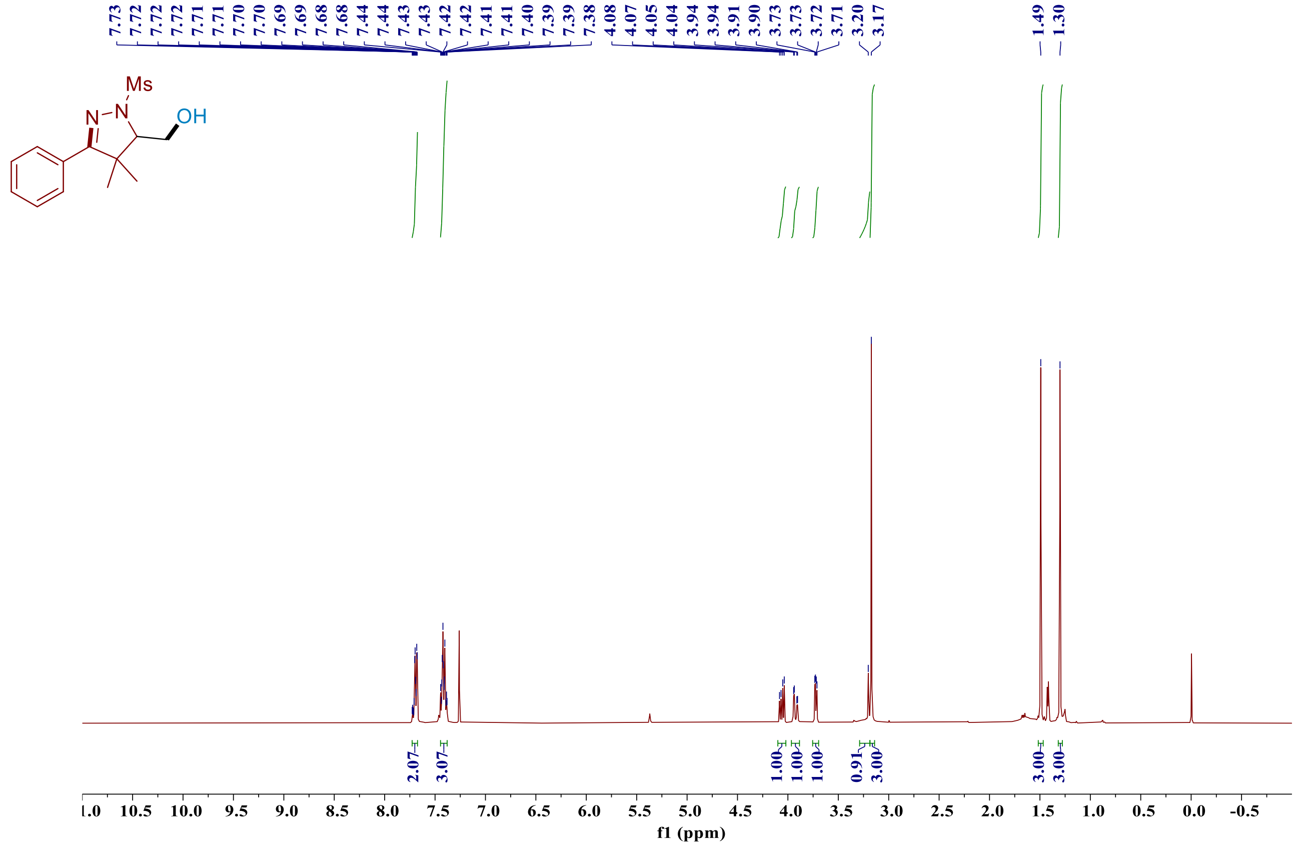

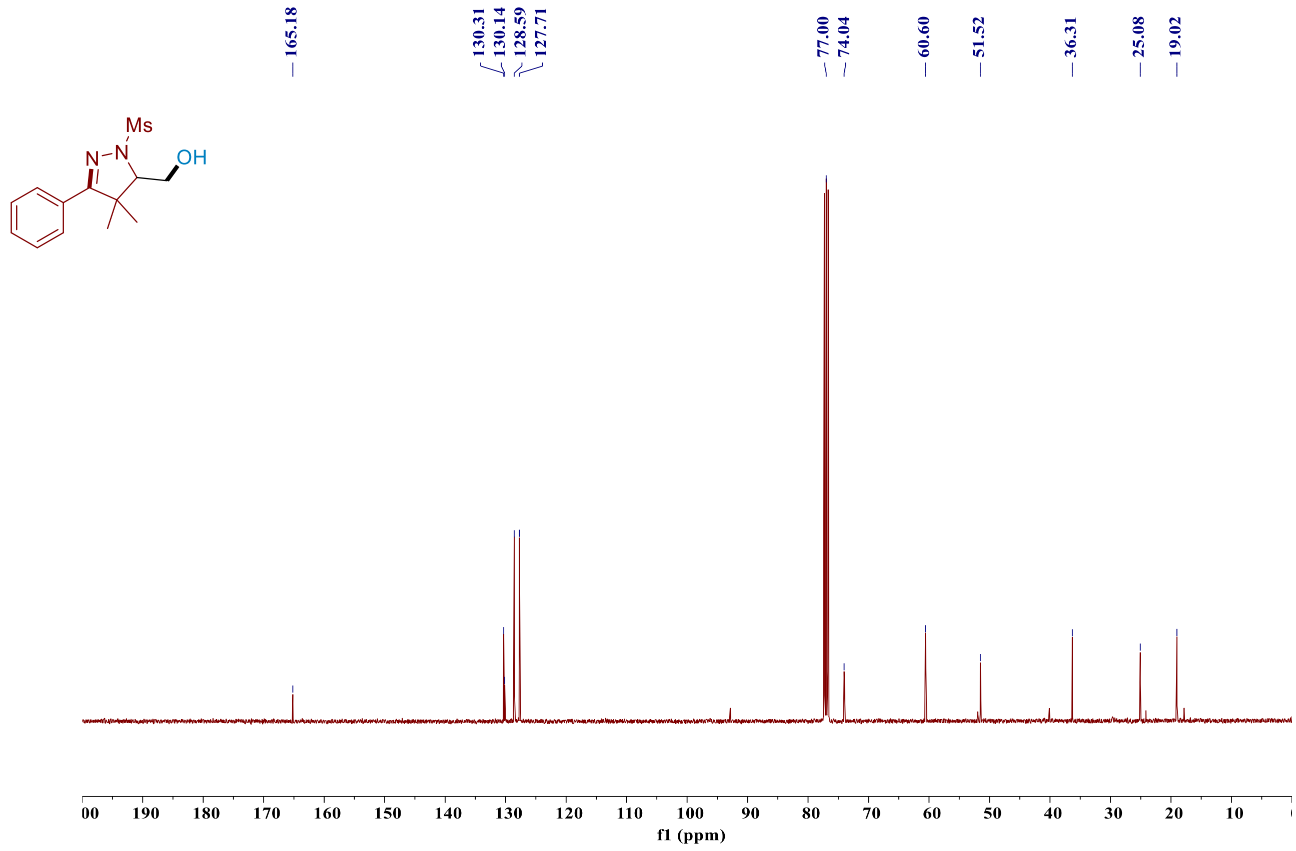

^{1}H NMR (400 MHz, CDCl$_3$), ^{13}C NMR (101 MHz, CDCl$_3$) of product 33

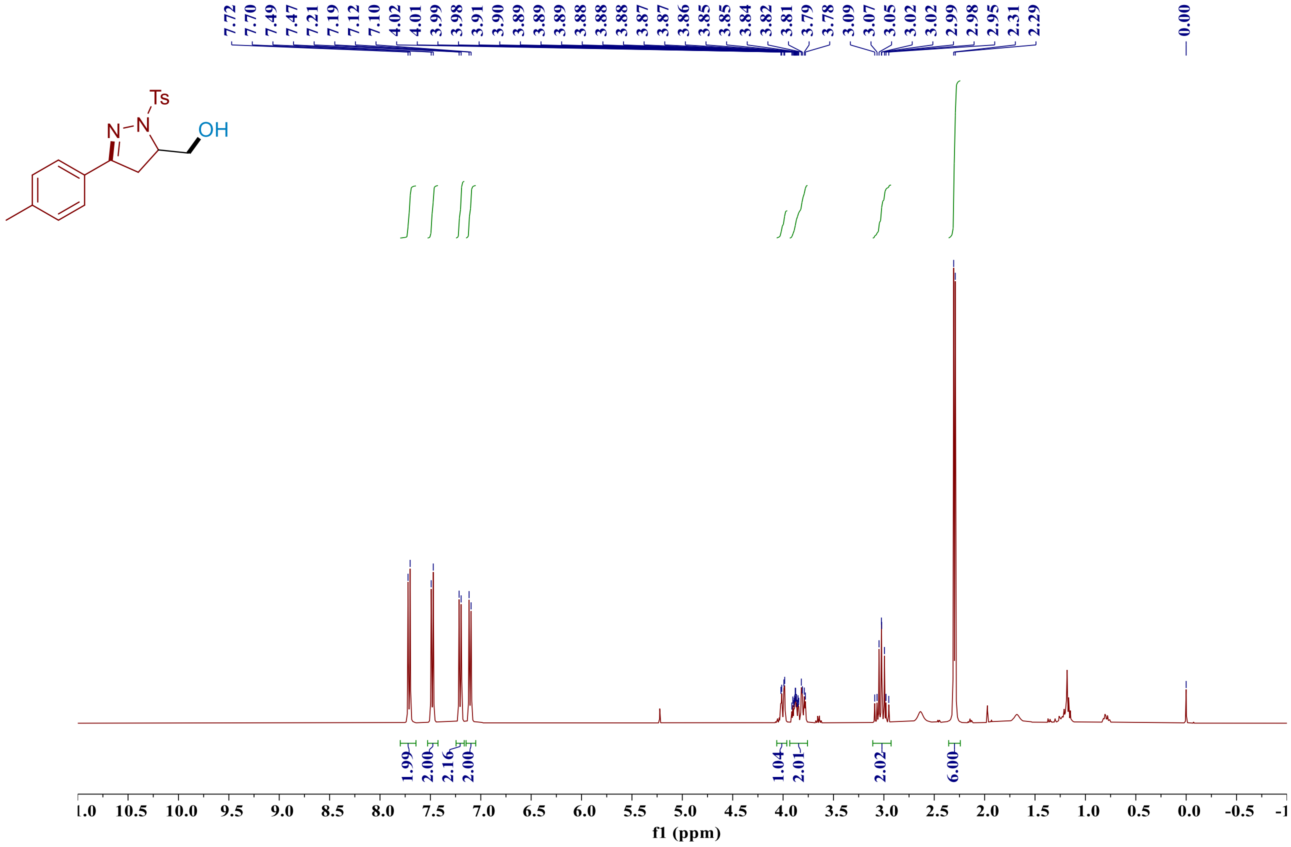

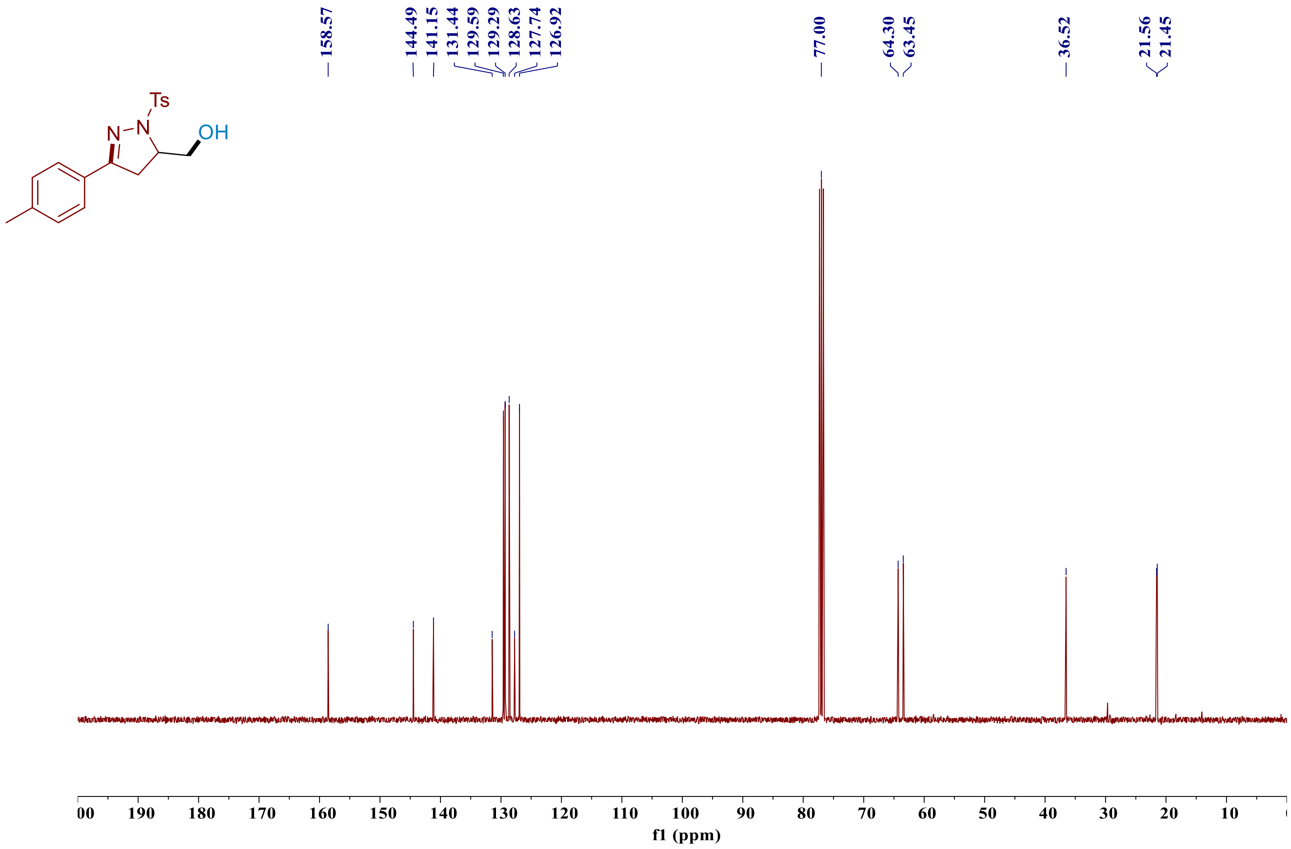

^{1}H NMR (400 MHz, $CDCl_3$), ^{13}C NMR (101 MHz, $CDCl_3$) of product 34

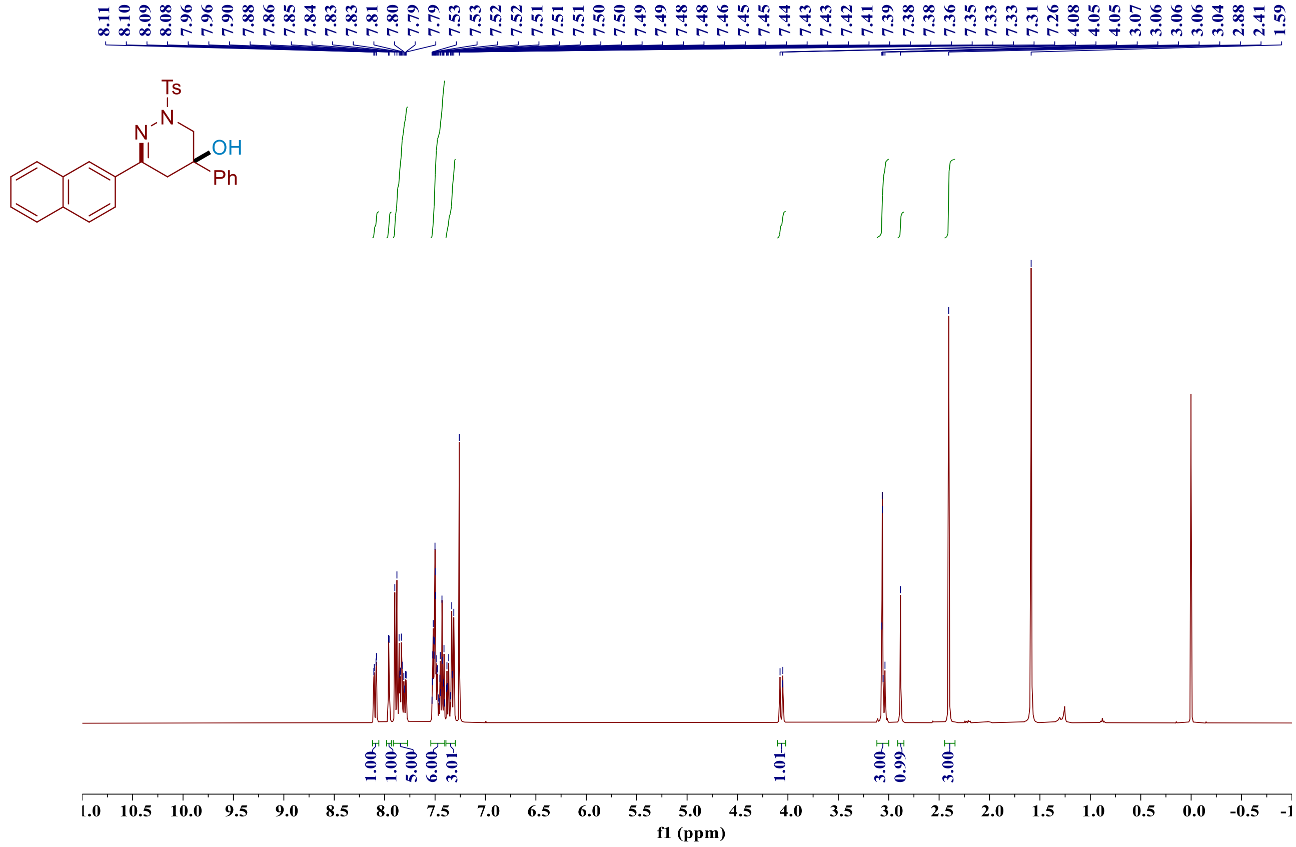

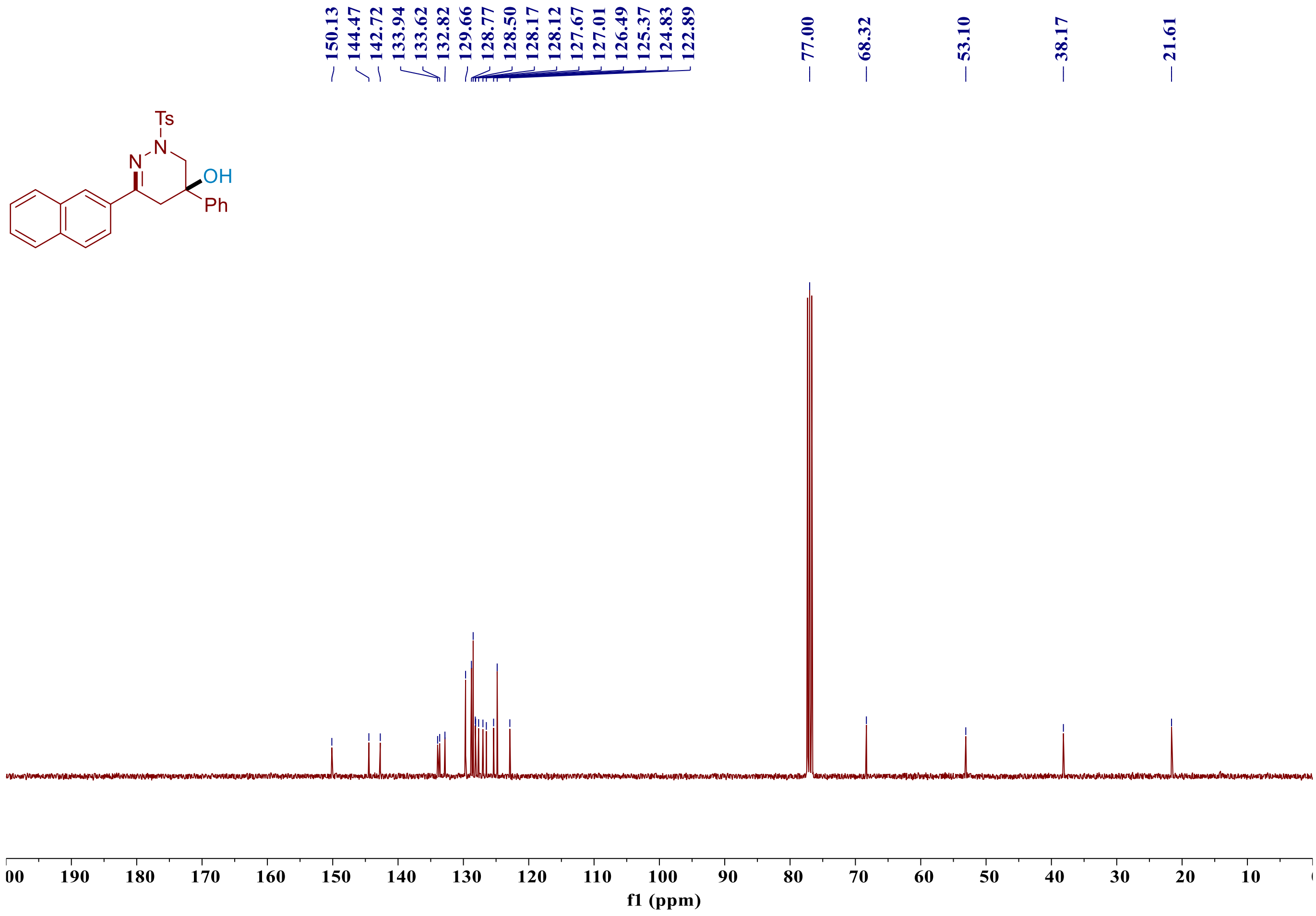

^{1}H NMR (400 MHz, $CDCl_3$), ^{13}C NMR (101 MHz, $CDCl_3$) of product 35

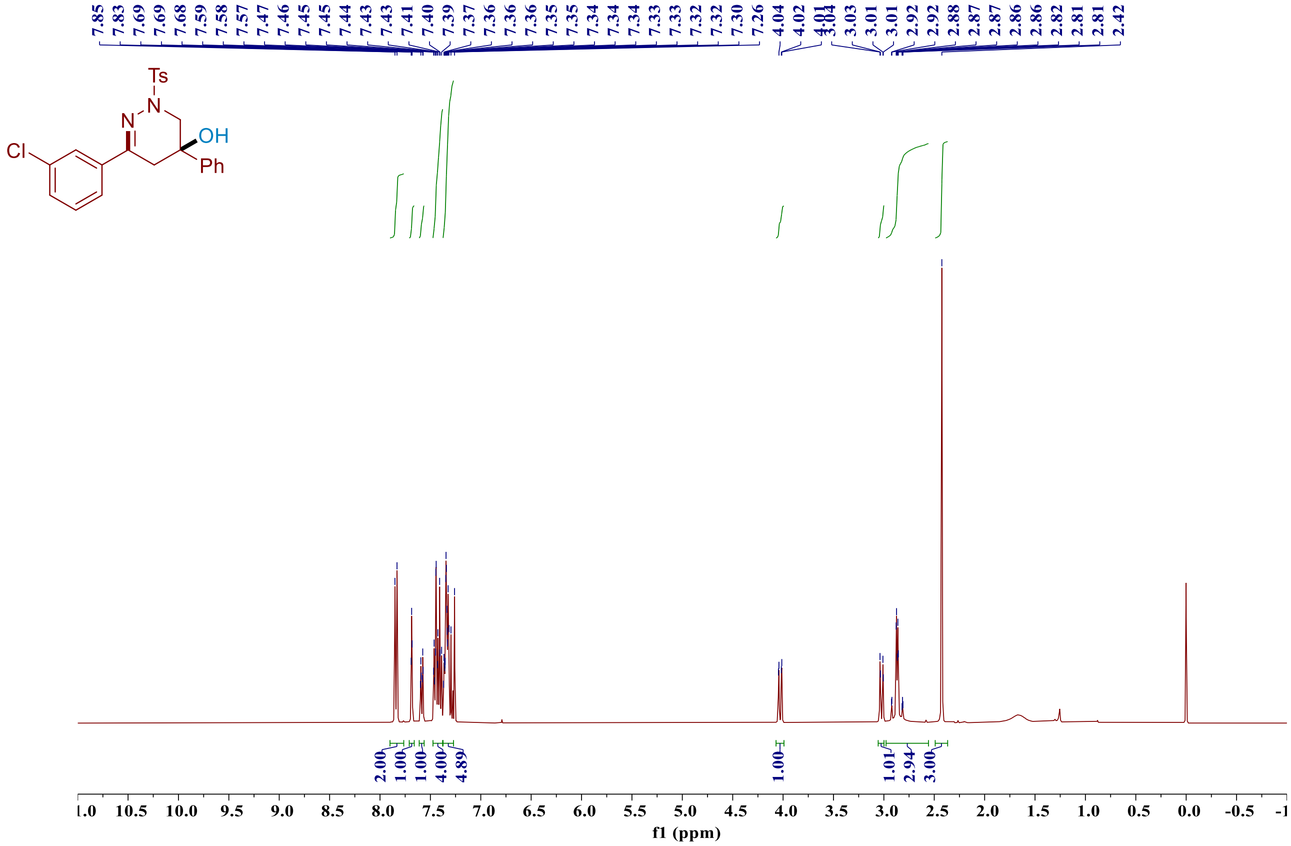

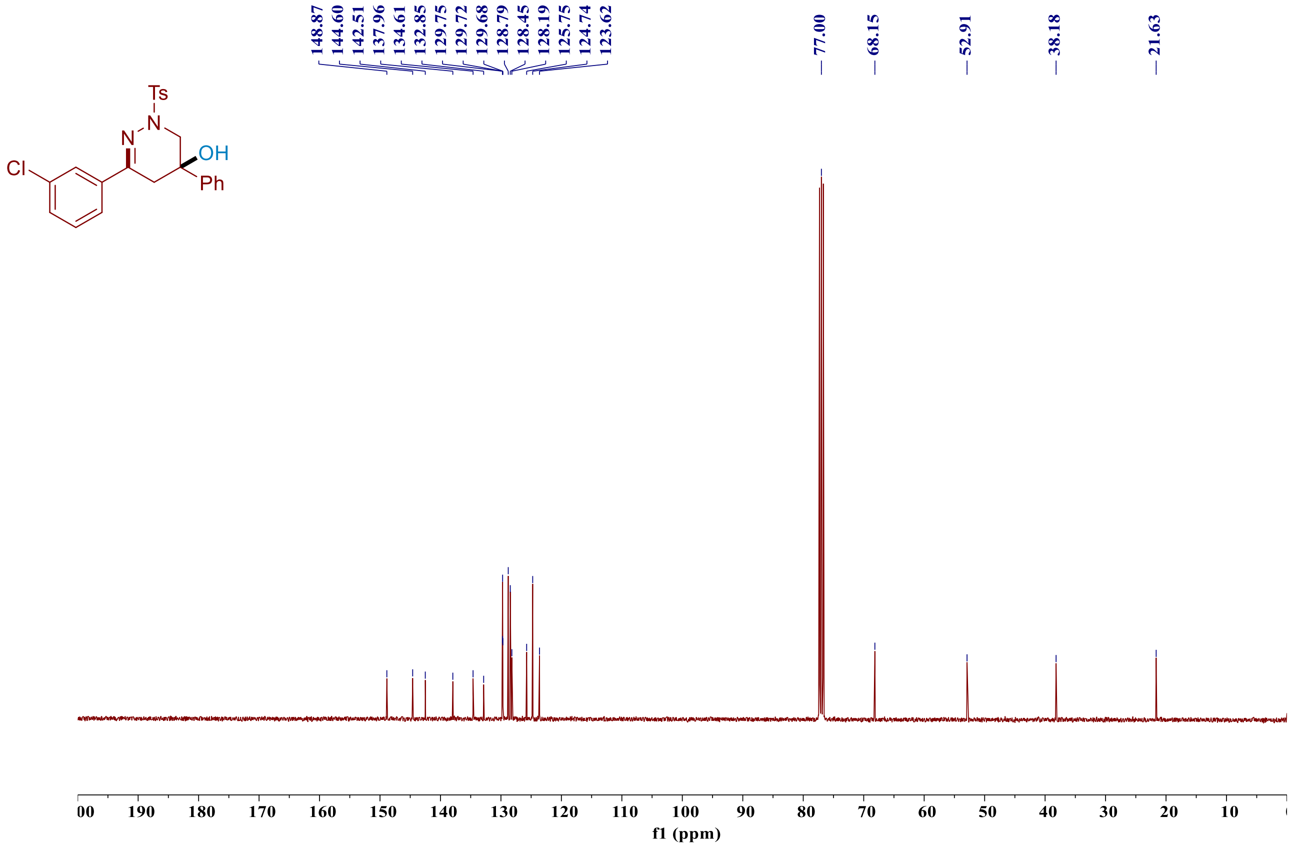

^{1}H NMR (400 MHz, $CDCl_3$), ^{13}C NMR (101 MHz, $CDCl_3$) of product 36

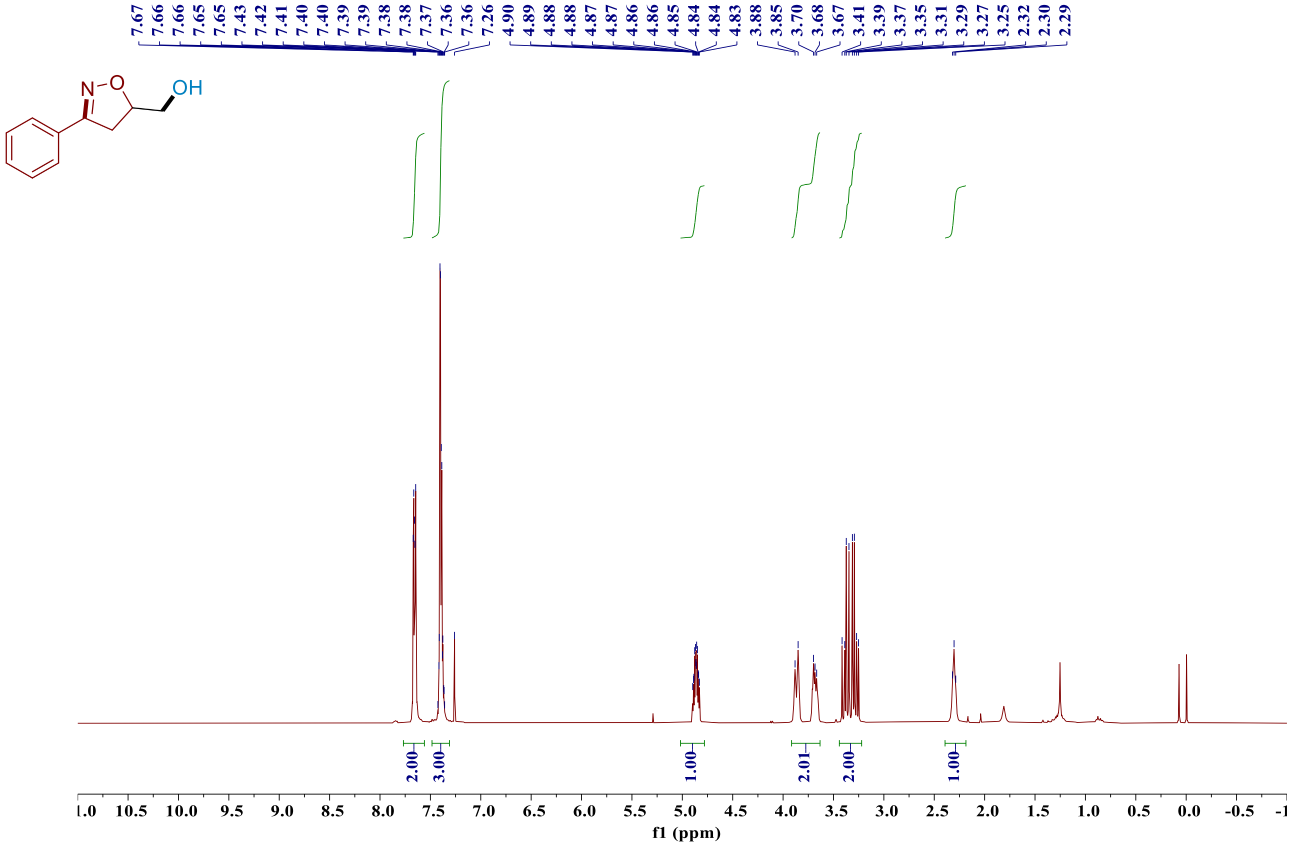

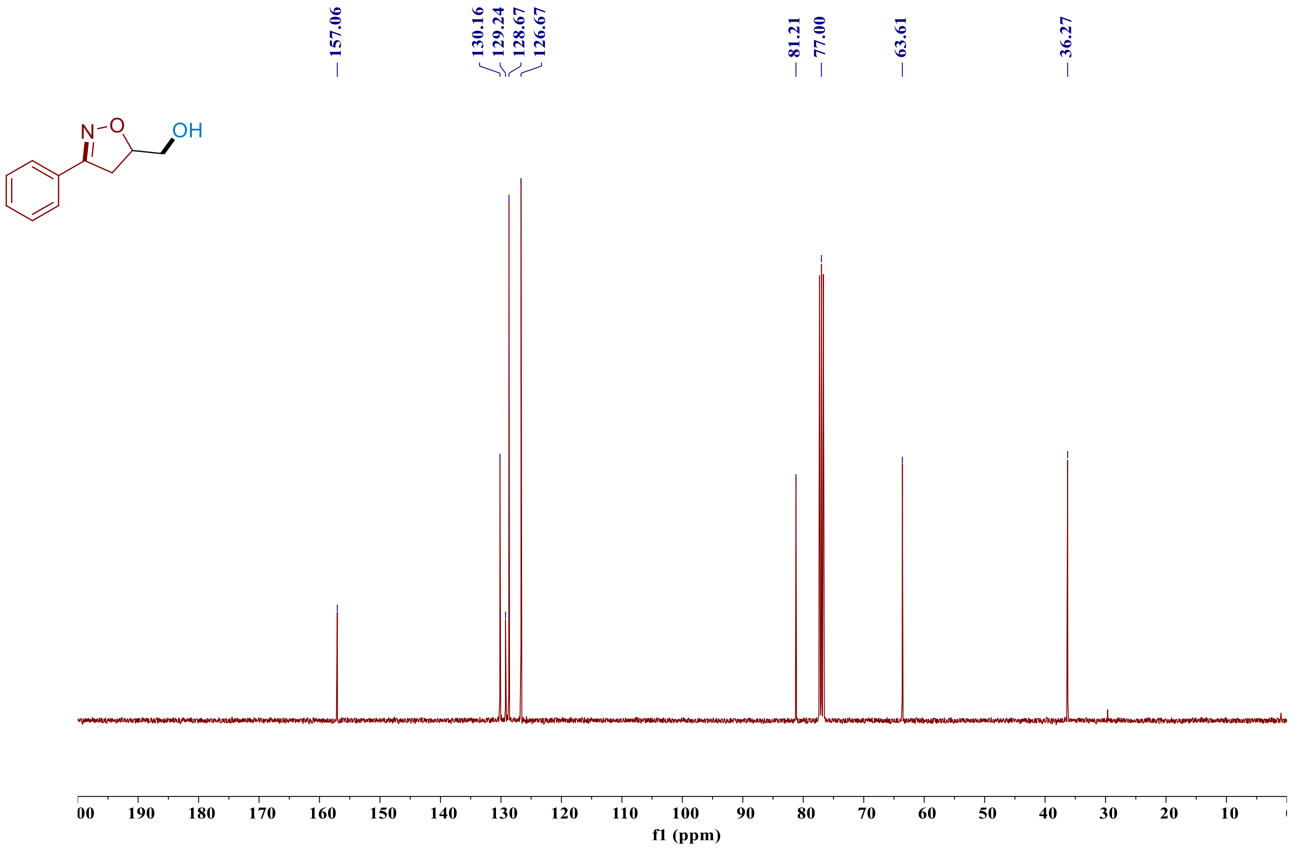

^{1}H NMR (400 MHz, CDCl$_3$), ^{13}C NMR (101 MHz, CDCl$_3$) of product 37

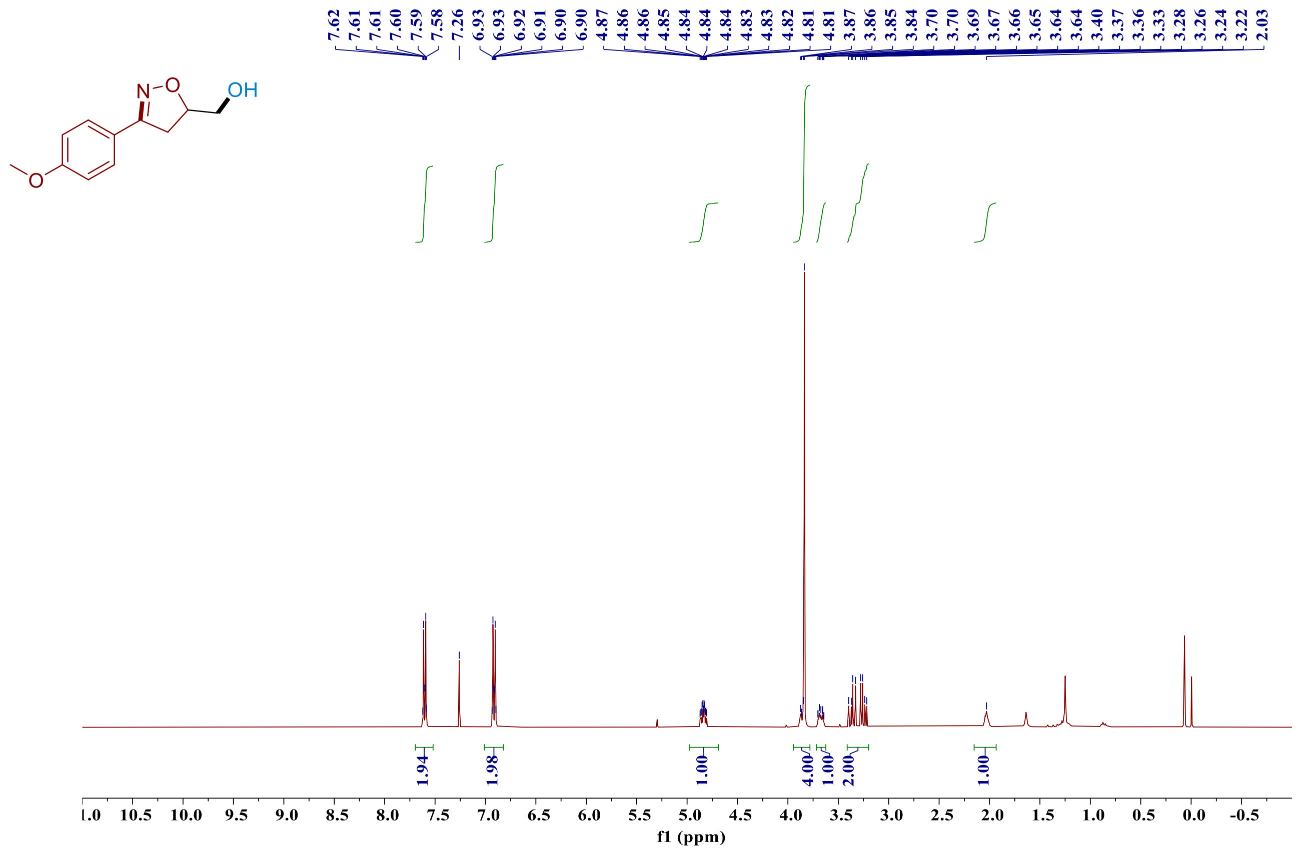

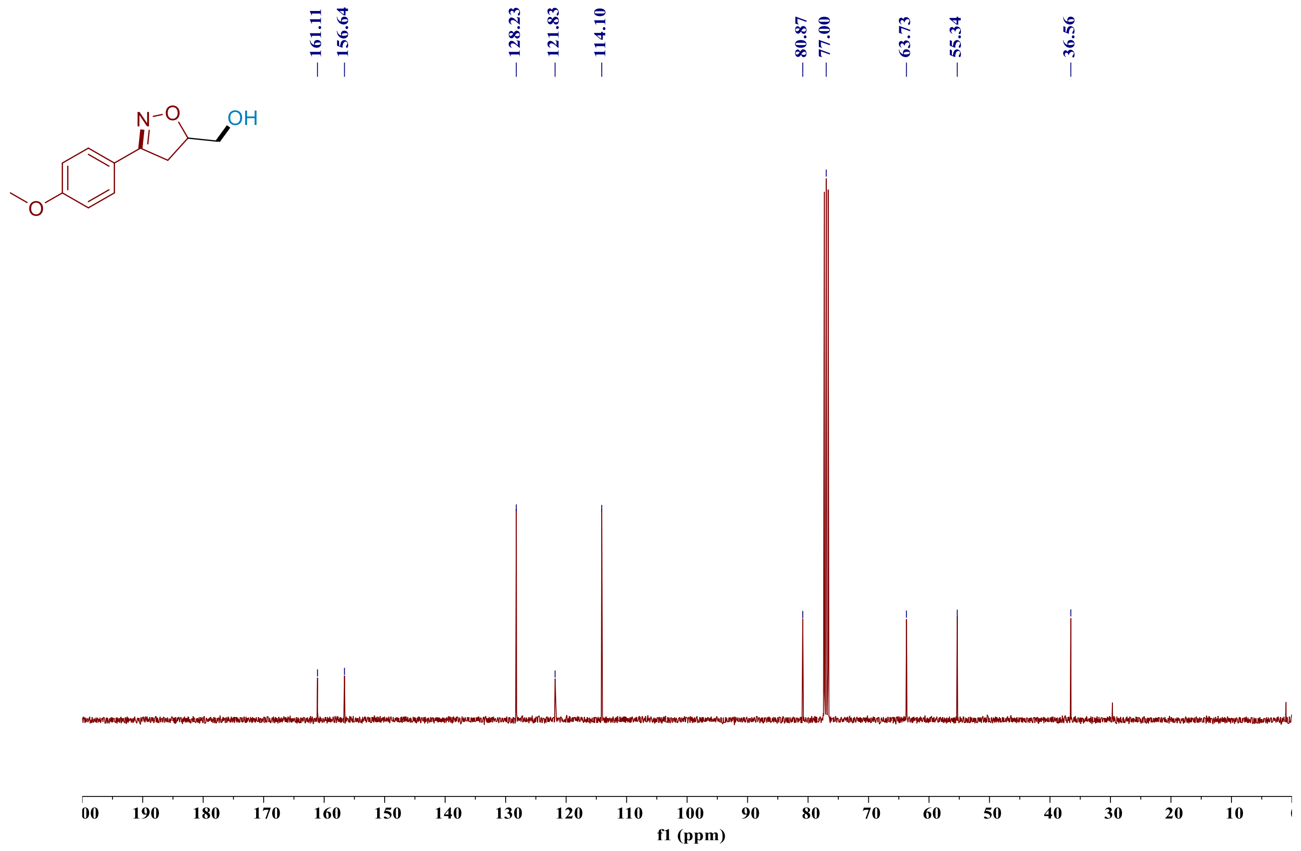

^{1}H NMR (400 MHz, $CDCl_3$), ^{13}C NMR (101 MHz, $CDCl_3$) of product 39

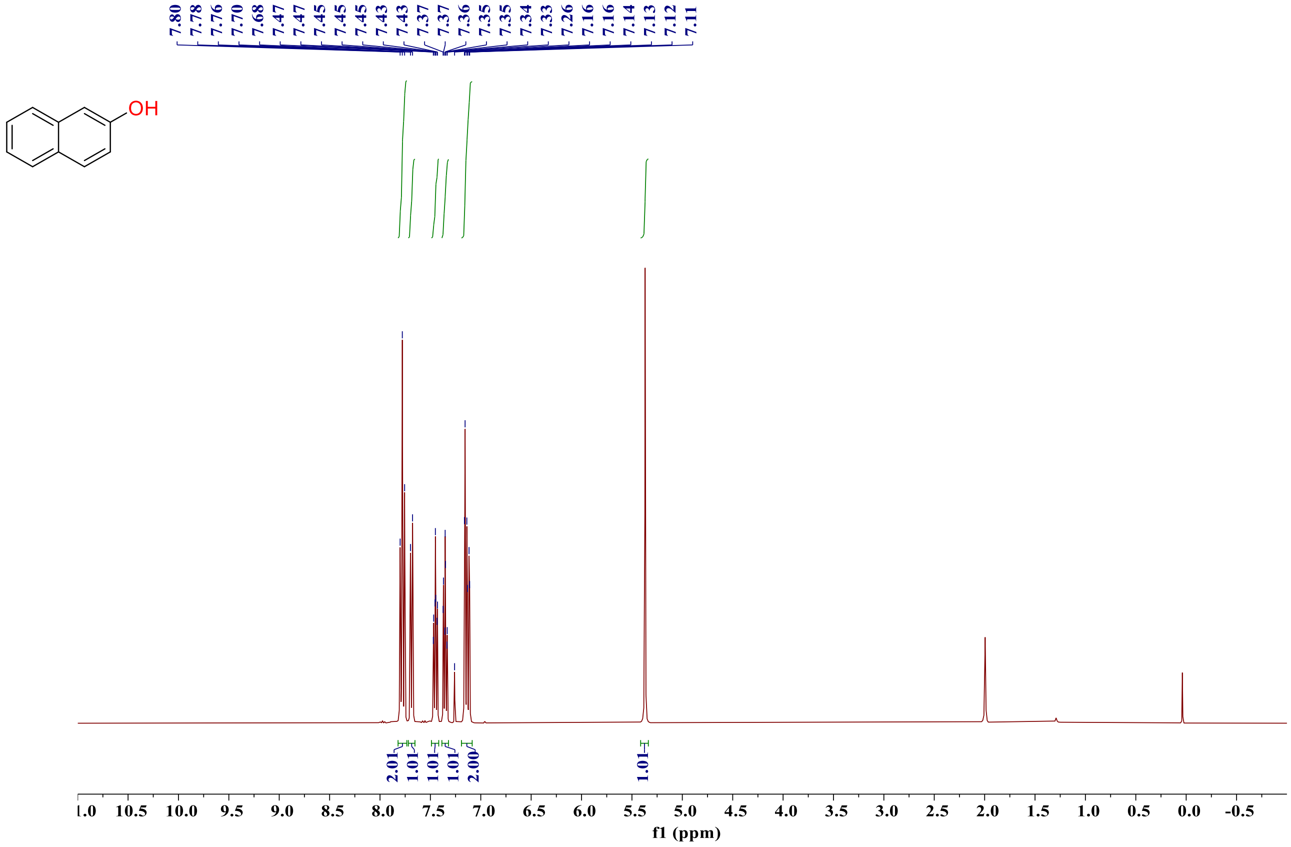

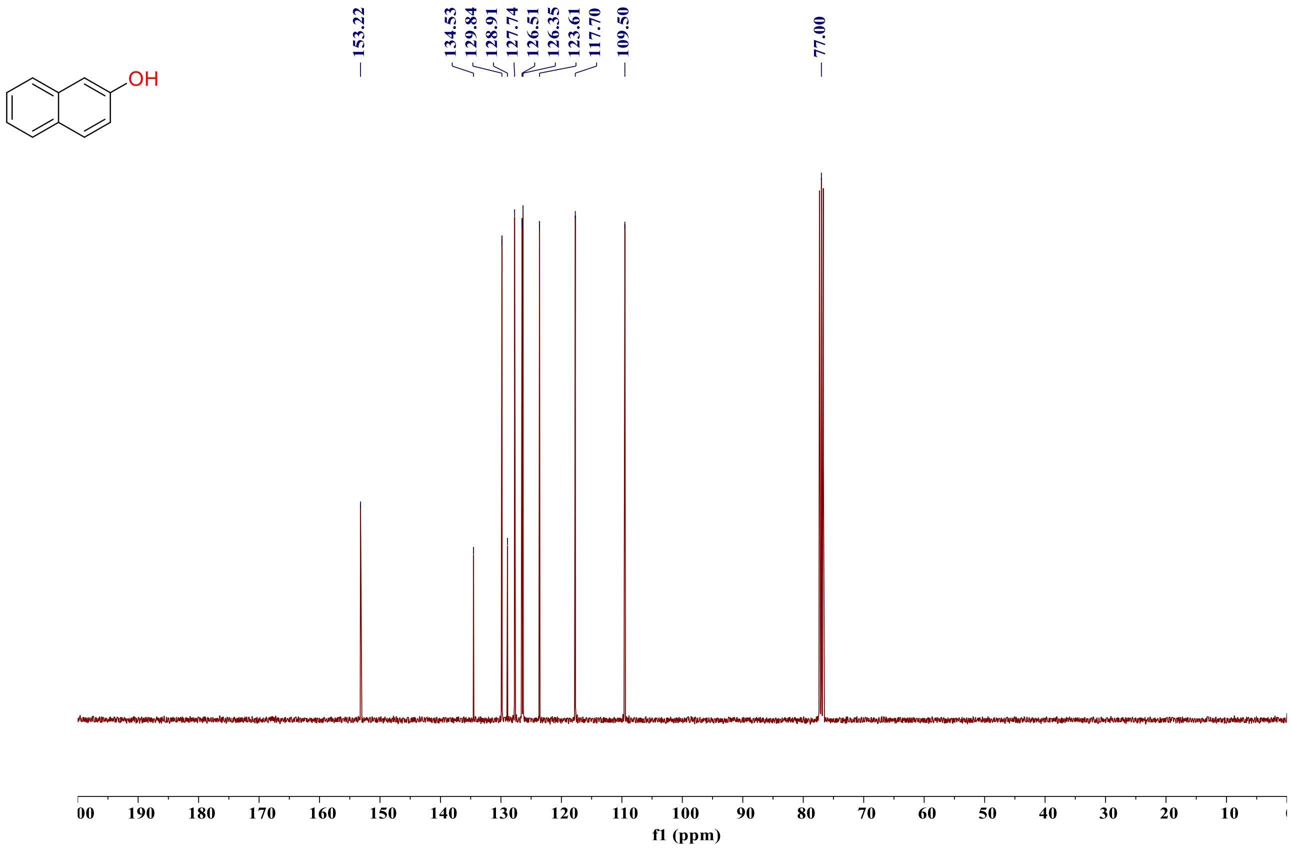

^{1}H NMR (400 MHz, $CDCl_3$), ^{13}C NMR (101 MHz, $CDCl_3$) of product 40

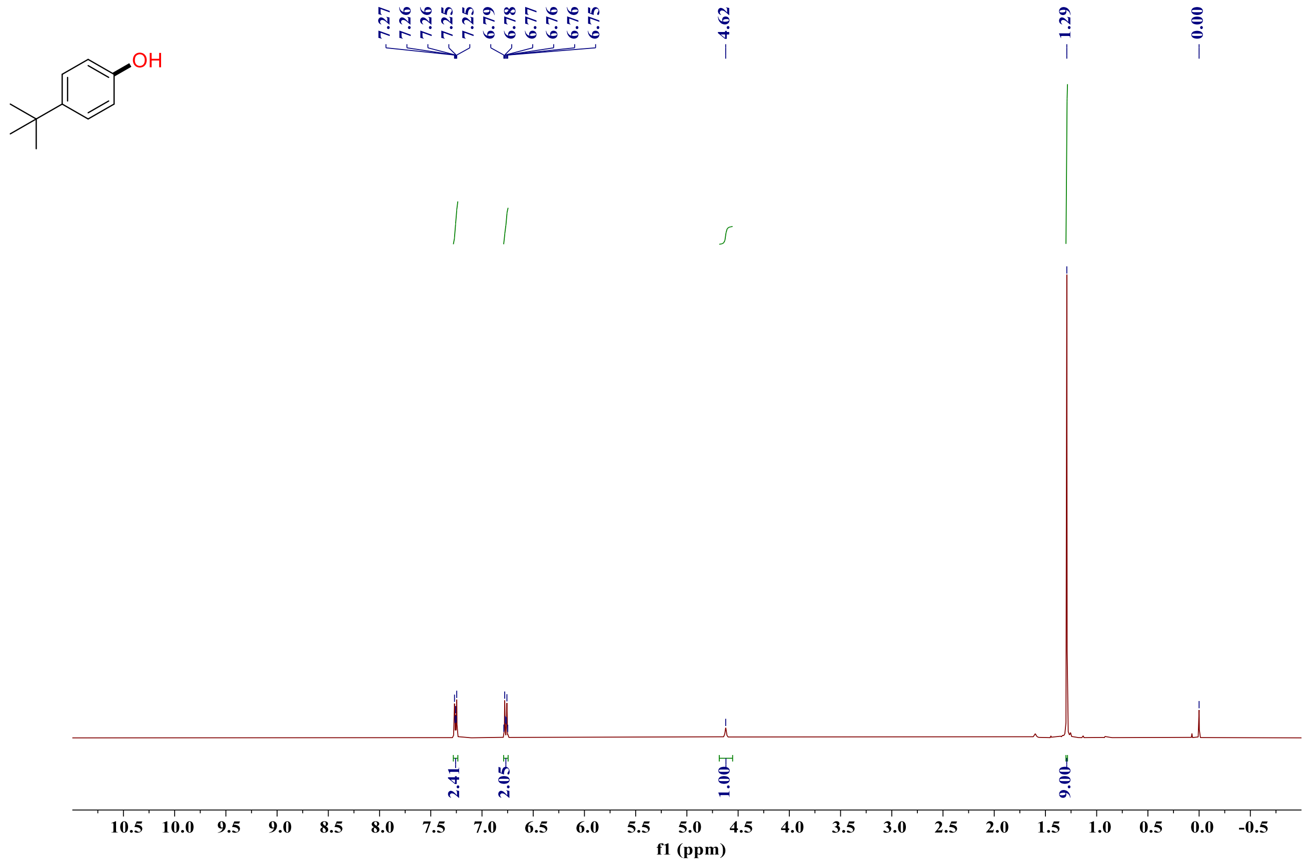

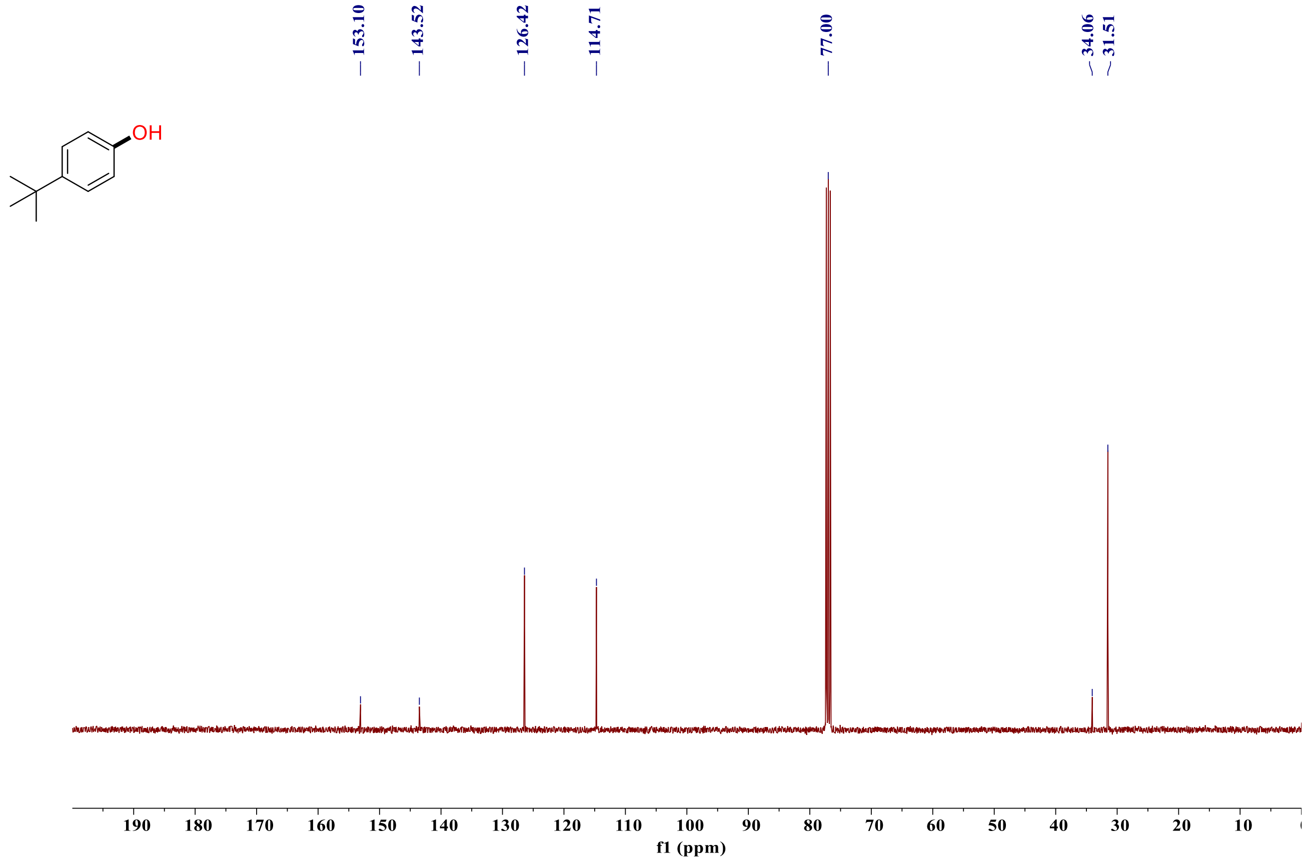

^{1}H NMR (400 MHz, $CDCl_3$), ^{13}C NMR (101 MHz, $CDCl_3$) of product 41

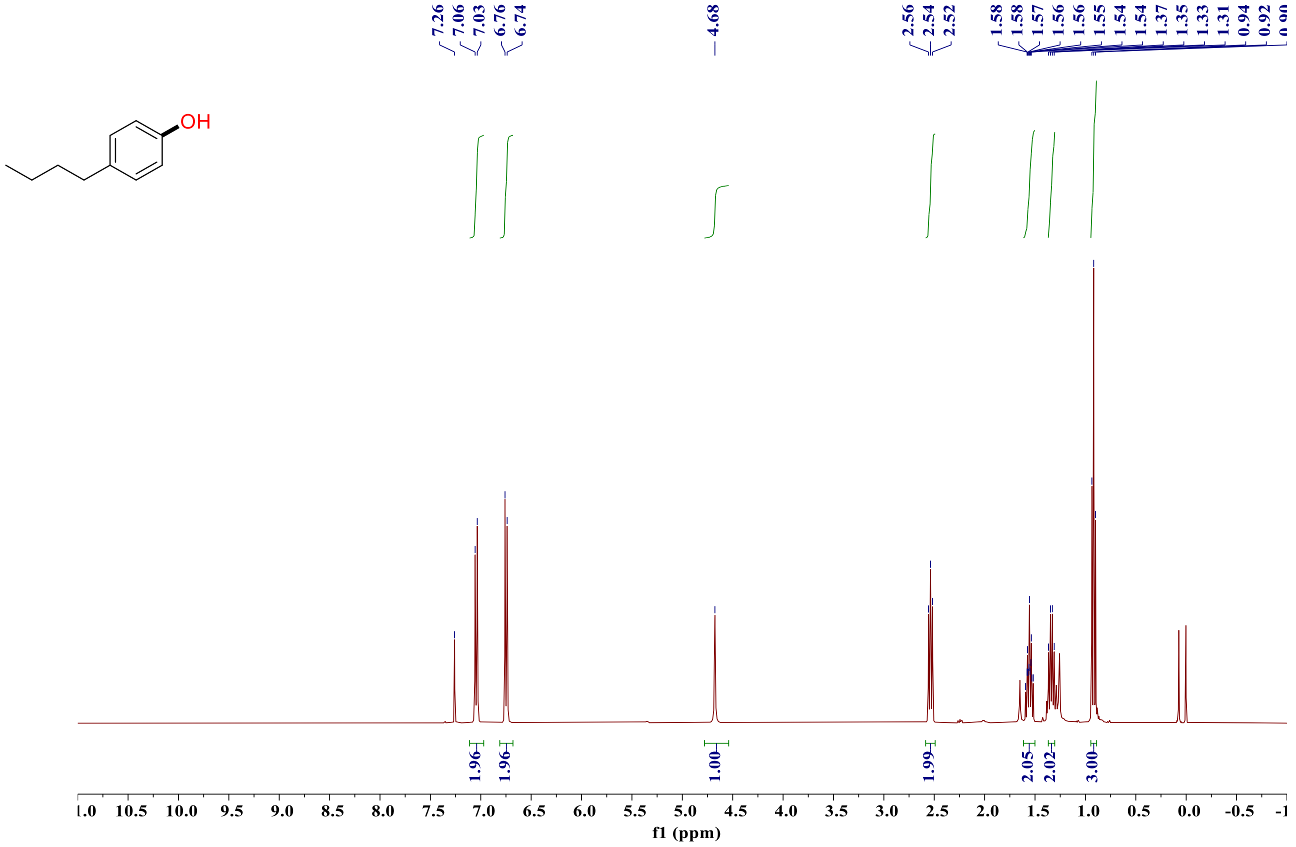

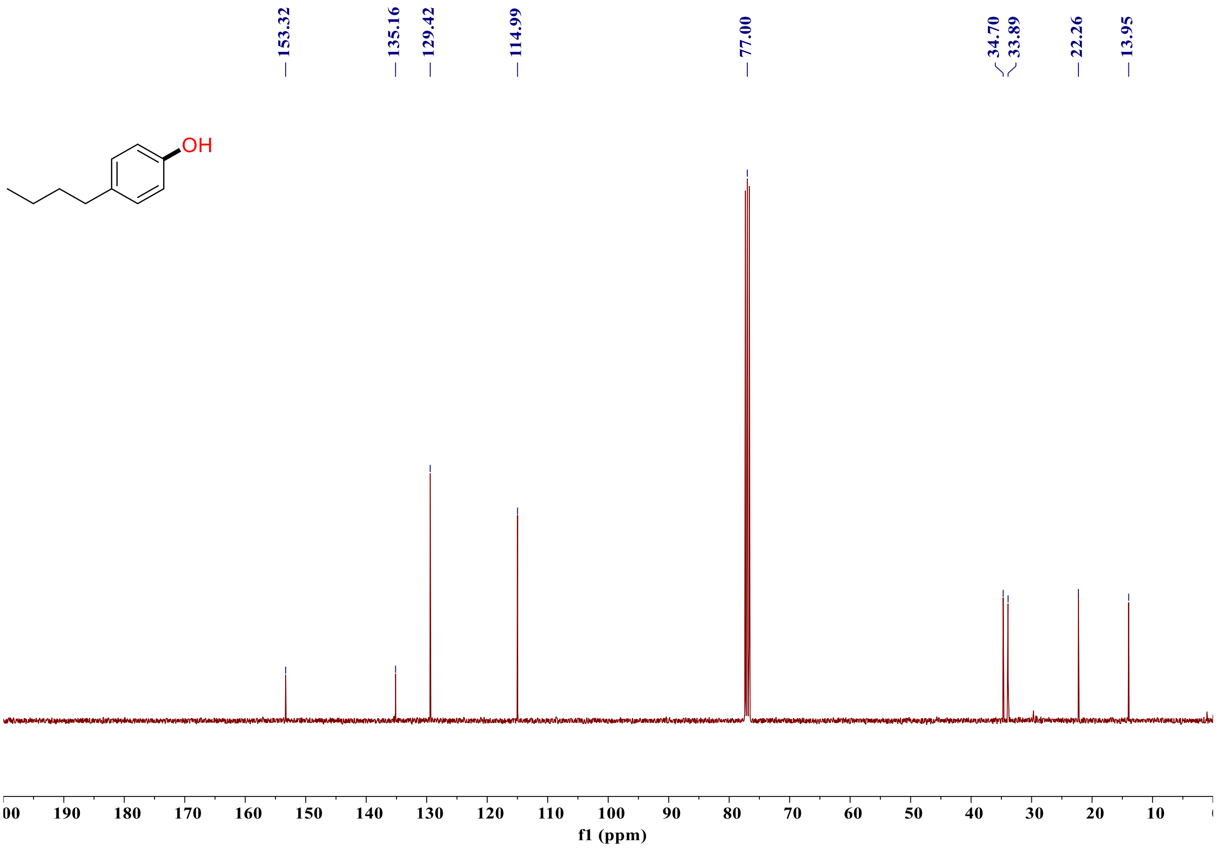

^{1}H NMR (400 MHz, CDCl$_3$), ^{13}C NMR (101 MHz, CDCl$_3$) of product 42

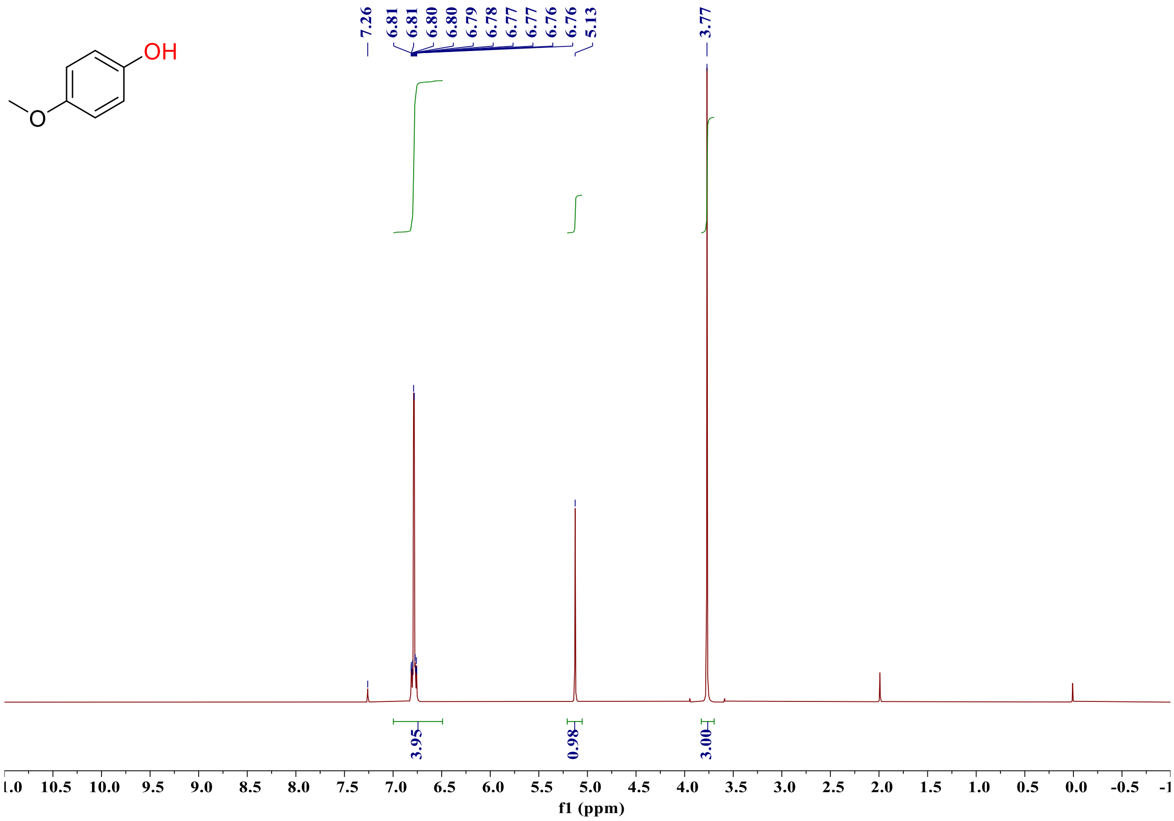

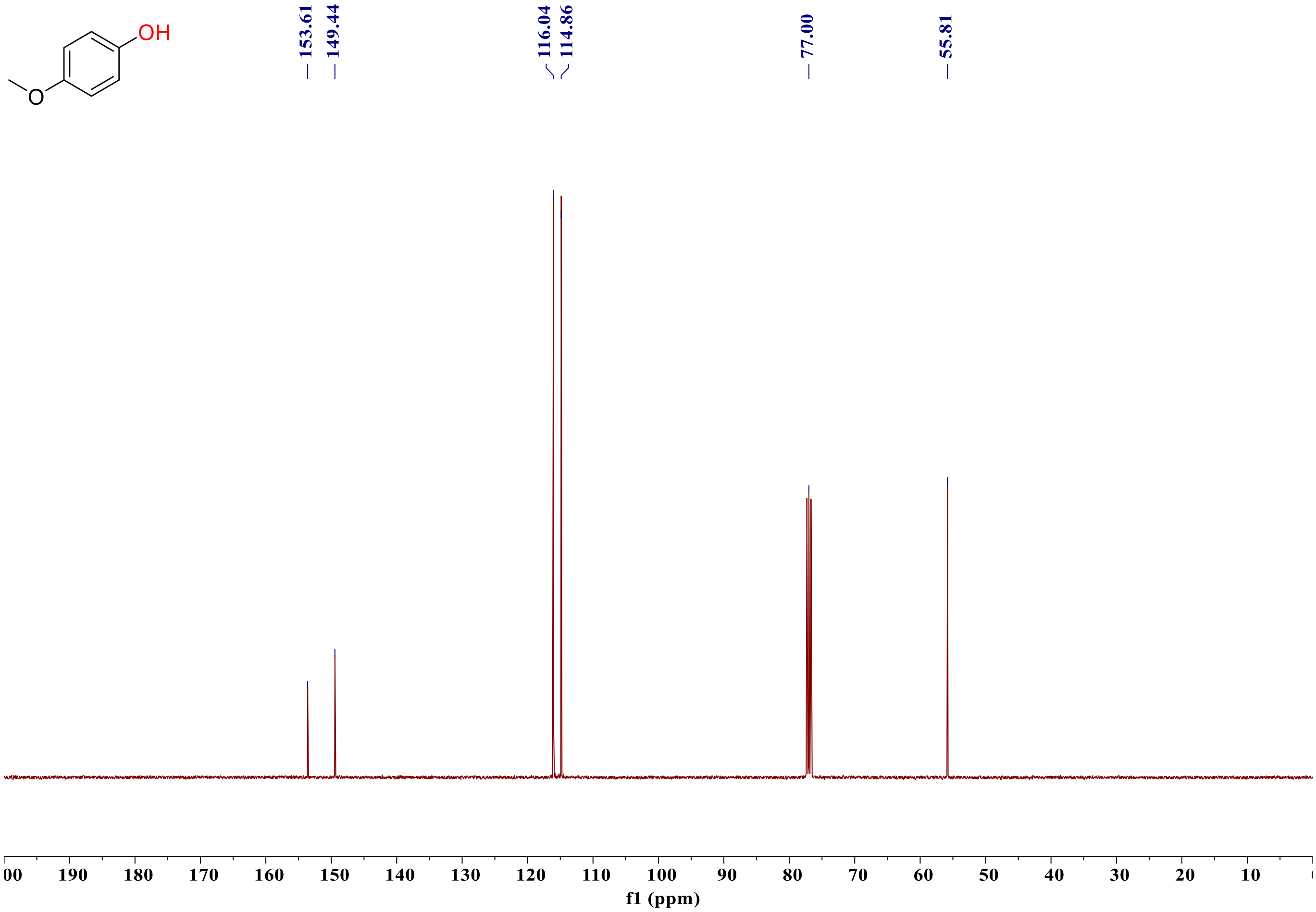

^{1}H NMR (400 MHz, $CDCl_3$), ^{13}C NMR (101 MHz, $CDCl_3$) of product 43.

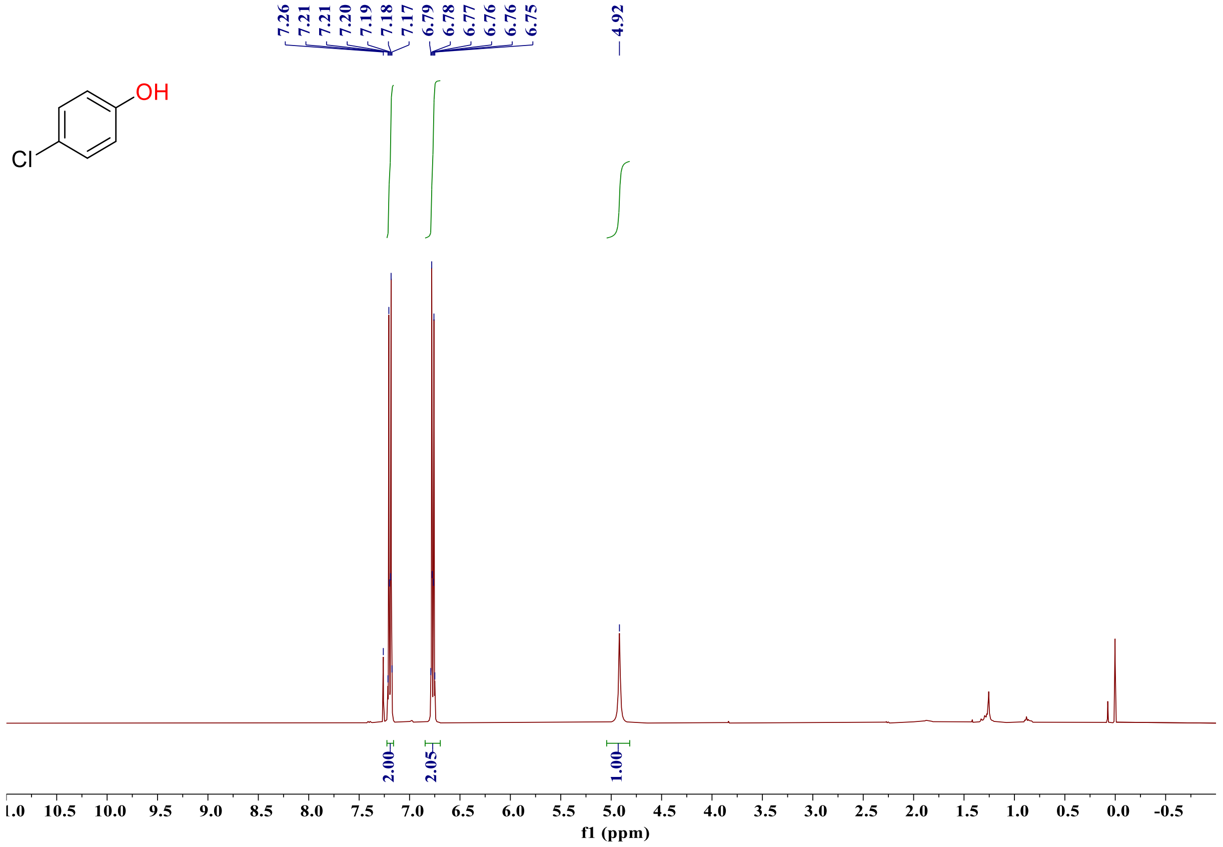

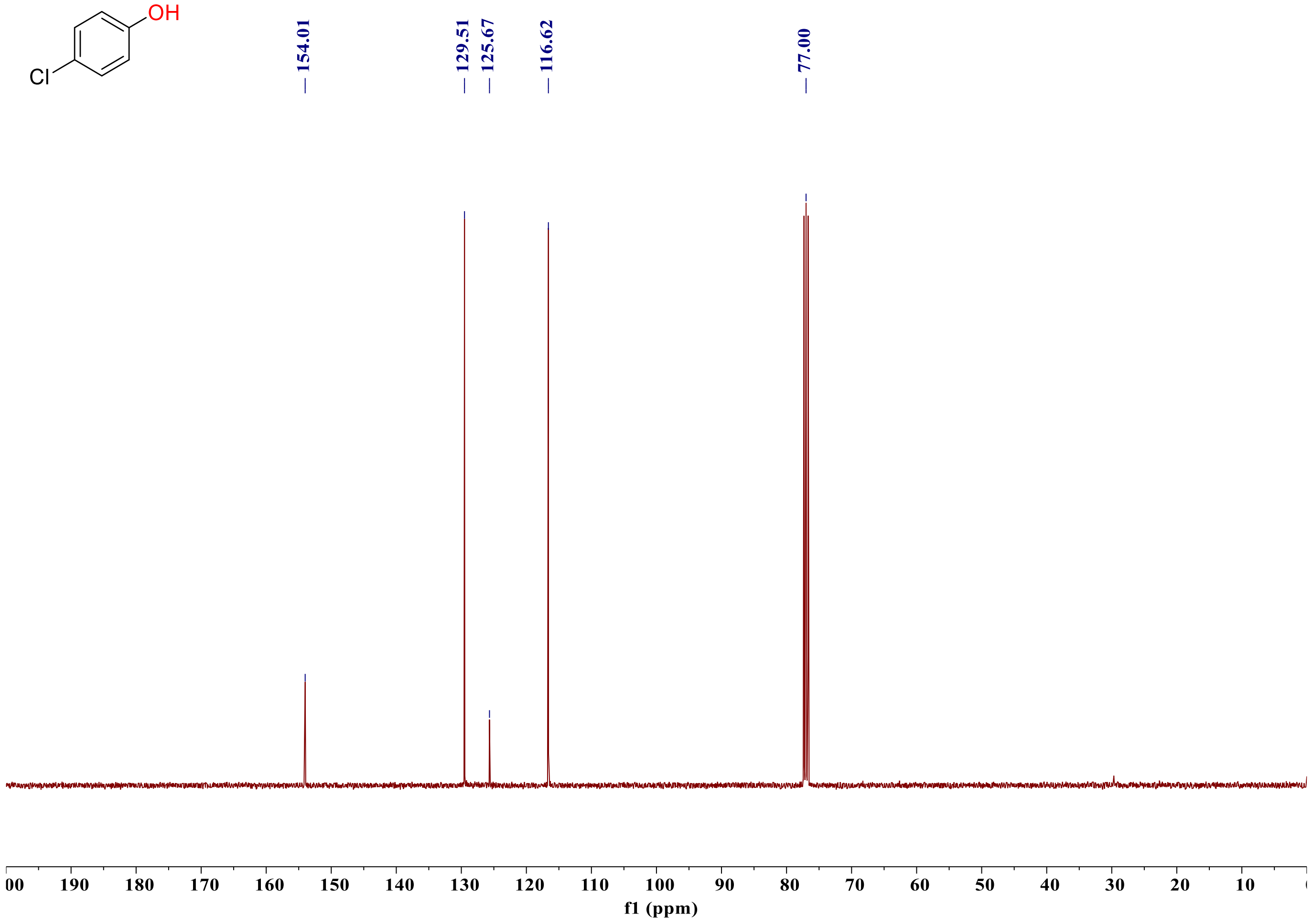

^{1}H NMR (400 MHz, $CDCl_3$), ^{13}C NMR (101 MHz, $CDCl_3$) of product 44.

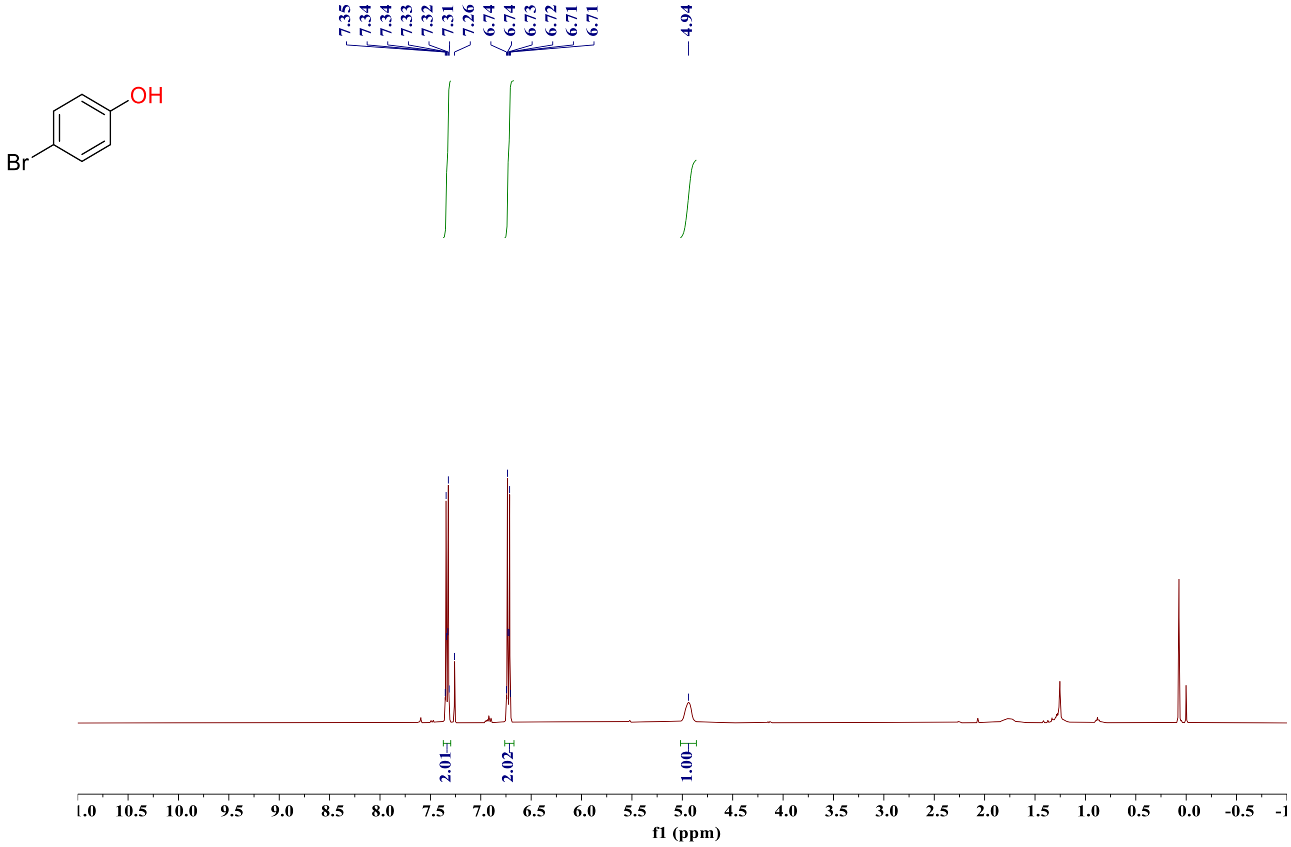

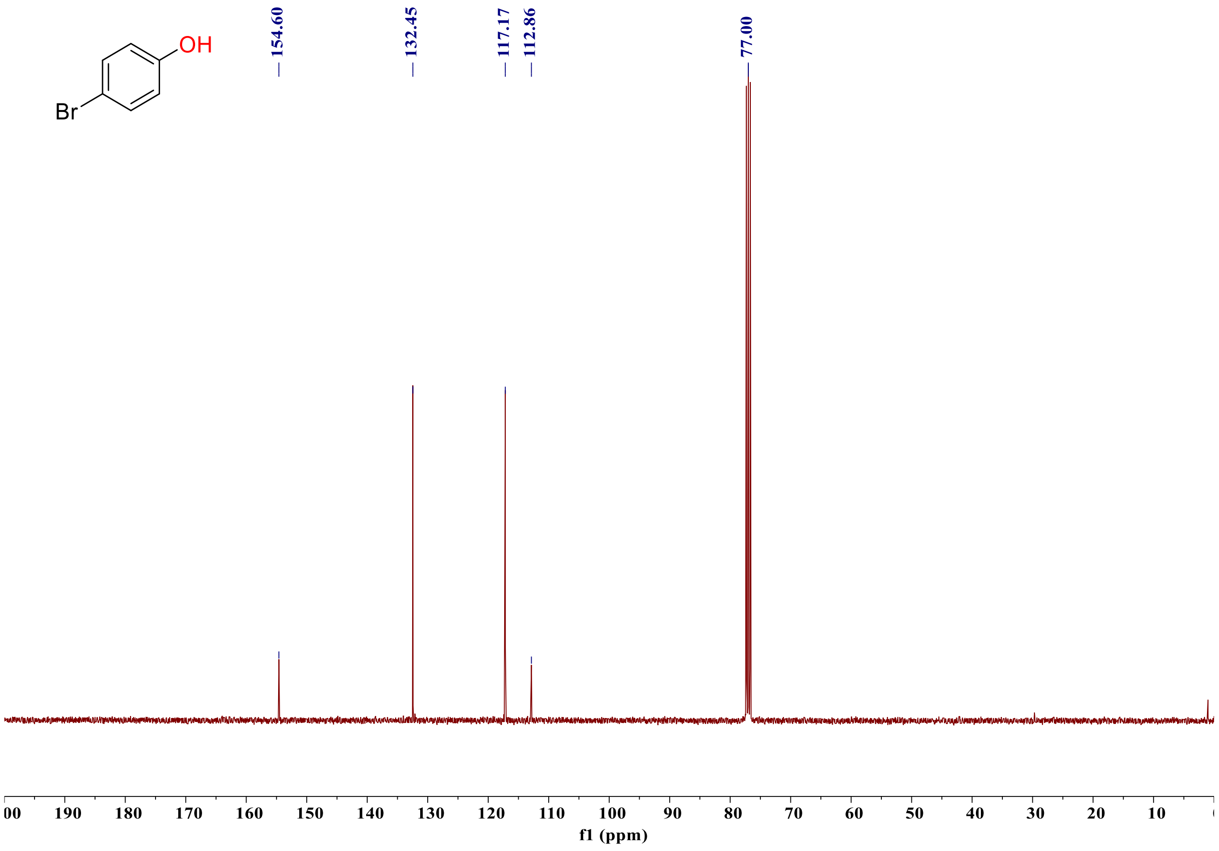

^{1}H NMR (400 MHz, $CDCl_3$), ^{13}C NMR (101 MHz, $CDCl_3$) of product 45.

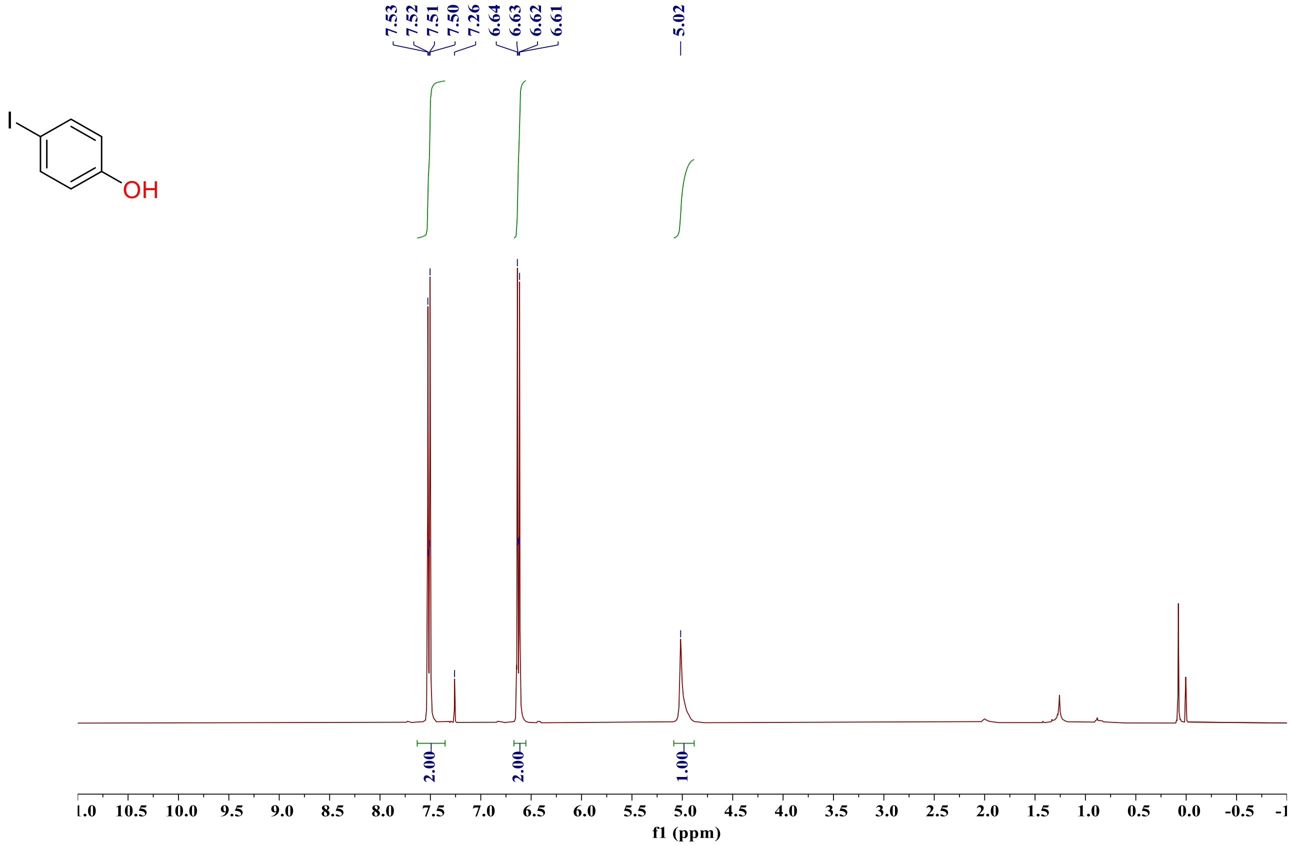

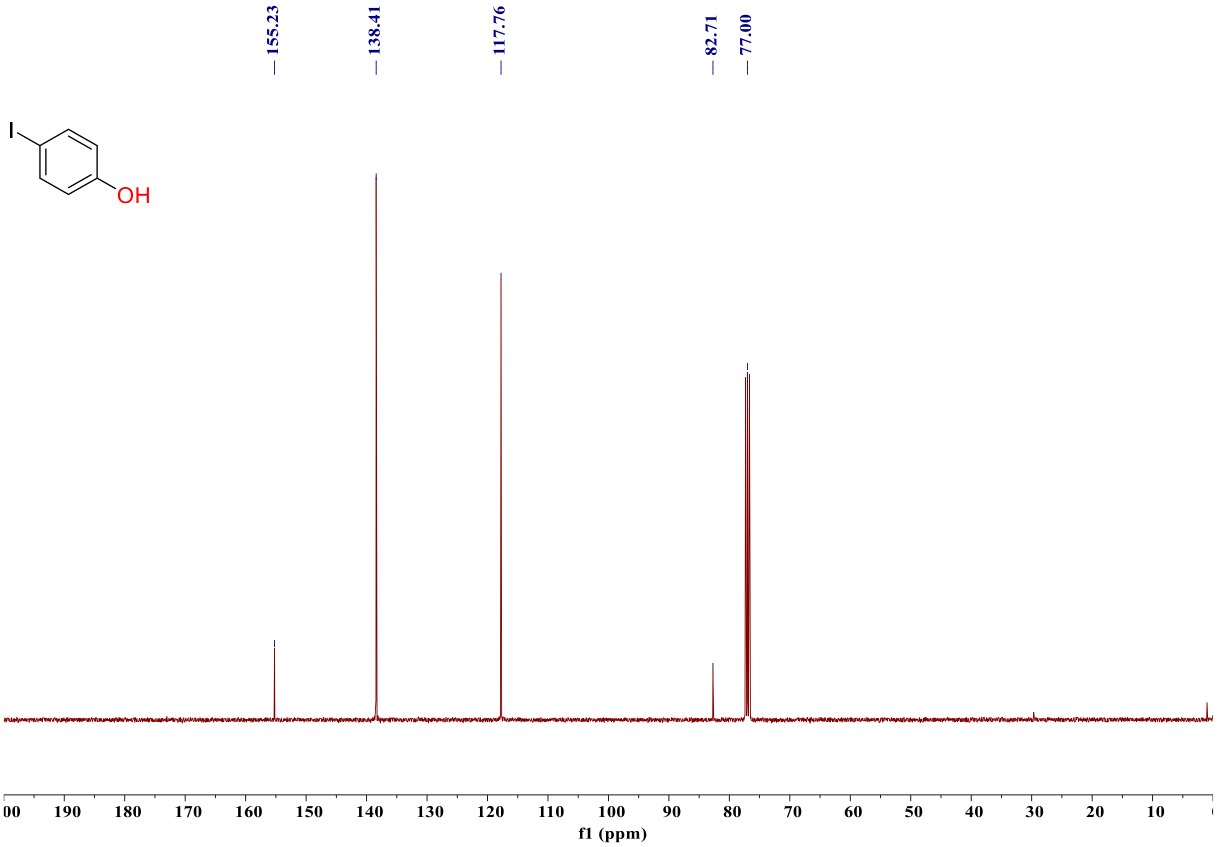

^{1}H NMR (400 MHz, $CDCl_3$), ^{13}C NMR (101 MHz, $CDCl_3$) of product 46

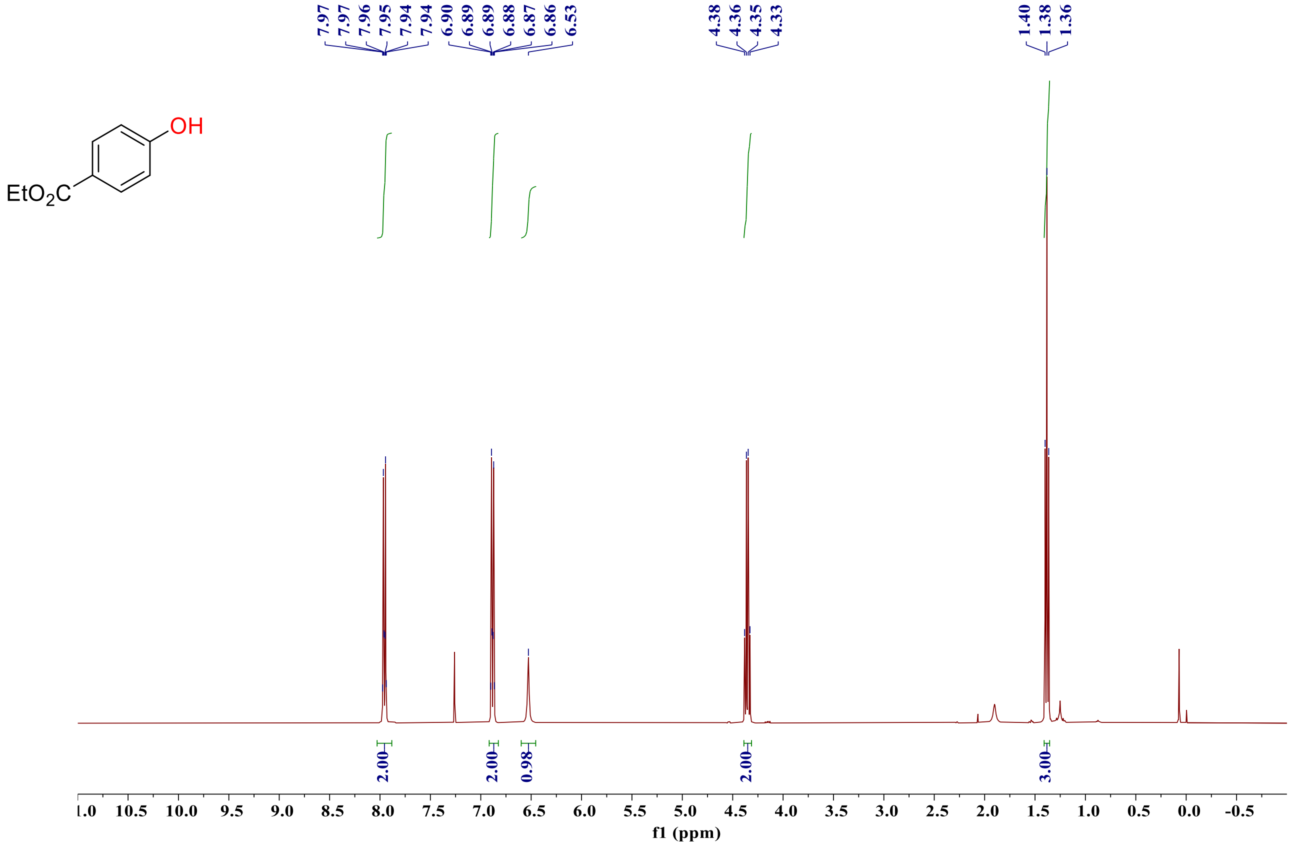

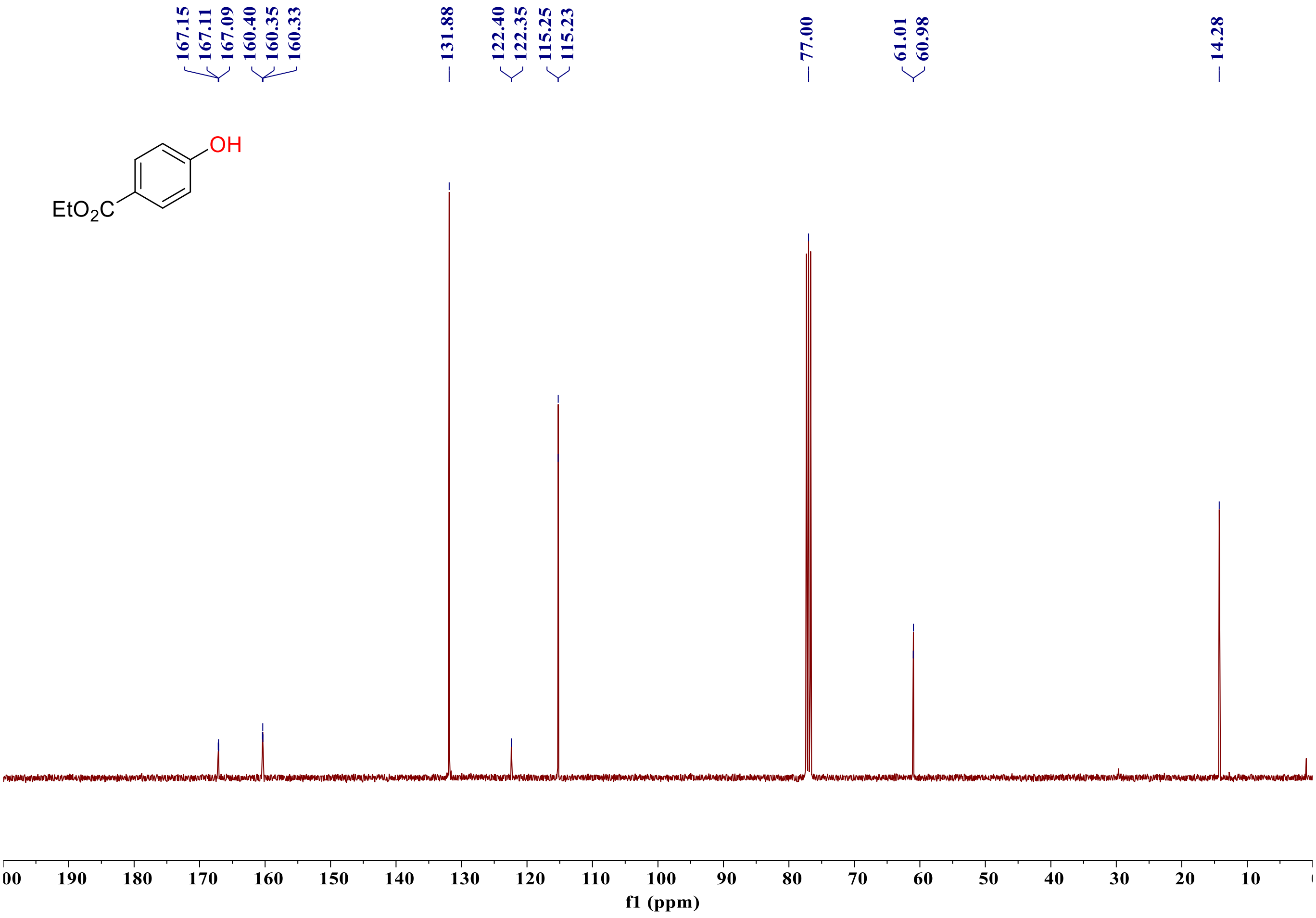

^{1}H NMR (400 MHz, CDCl$_3$), ^{13}C NMR (101 MHz, CDCl$_3$) of product 47

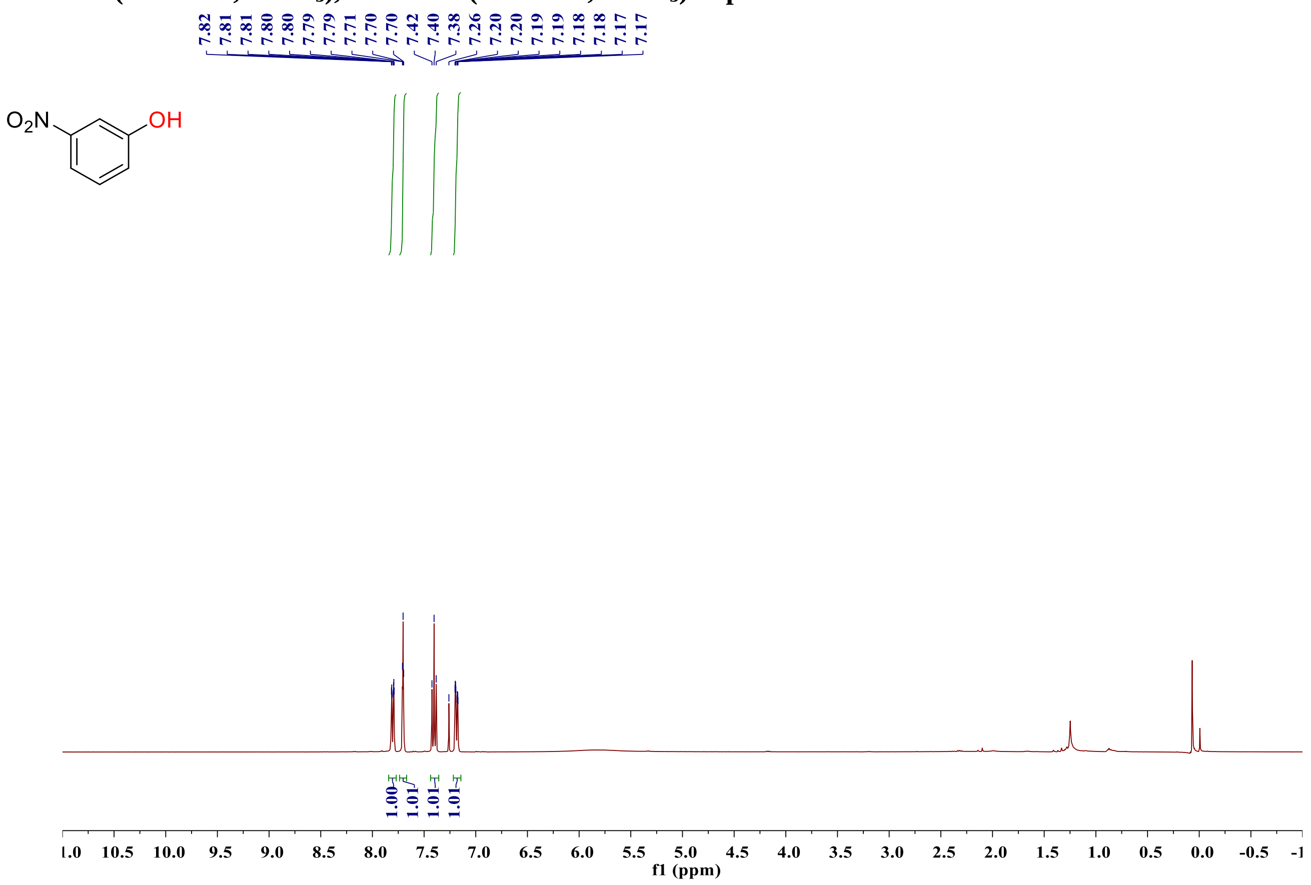

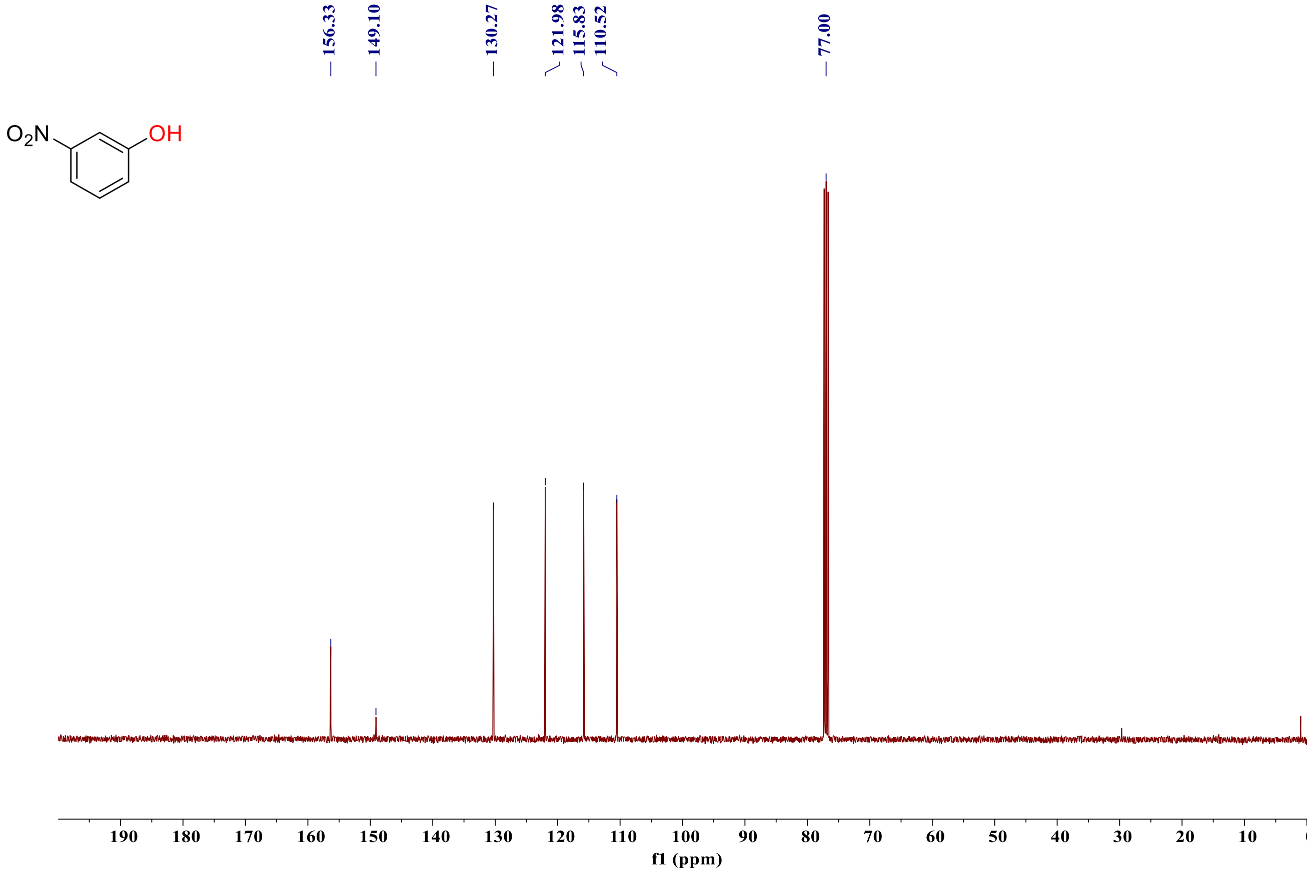

^{1}H NMR (400 MHz, CDCl$_3$), ^{13}C NMR (101 MHz, CDCl$_3$) of product 48

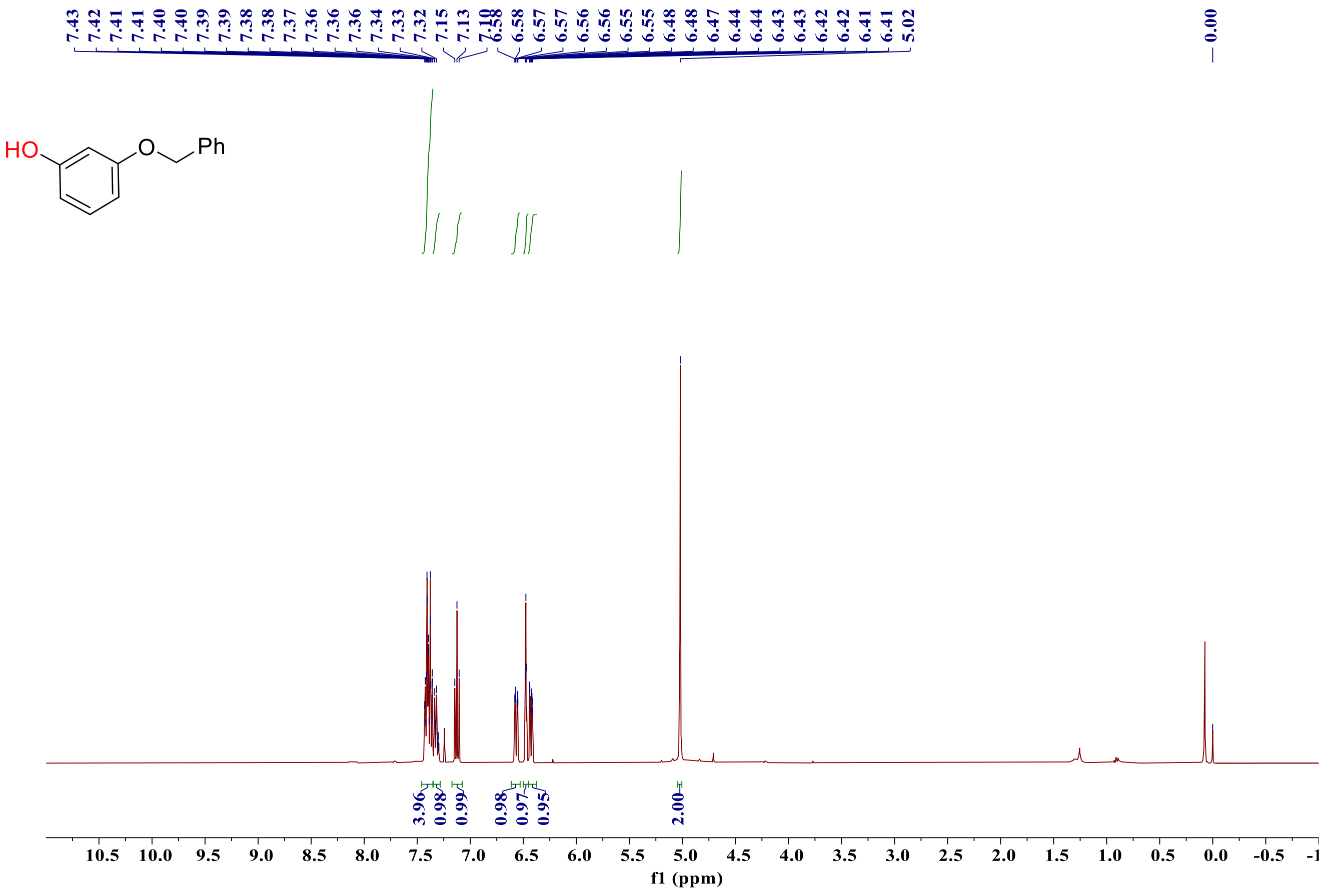

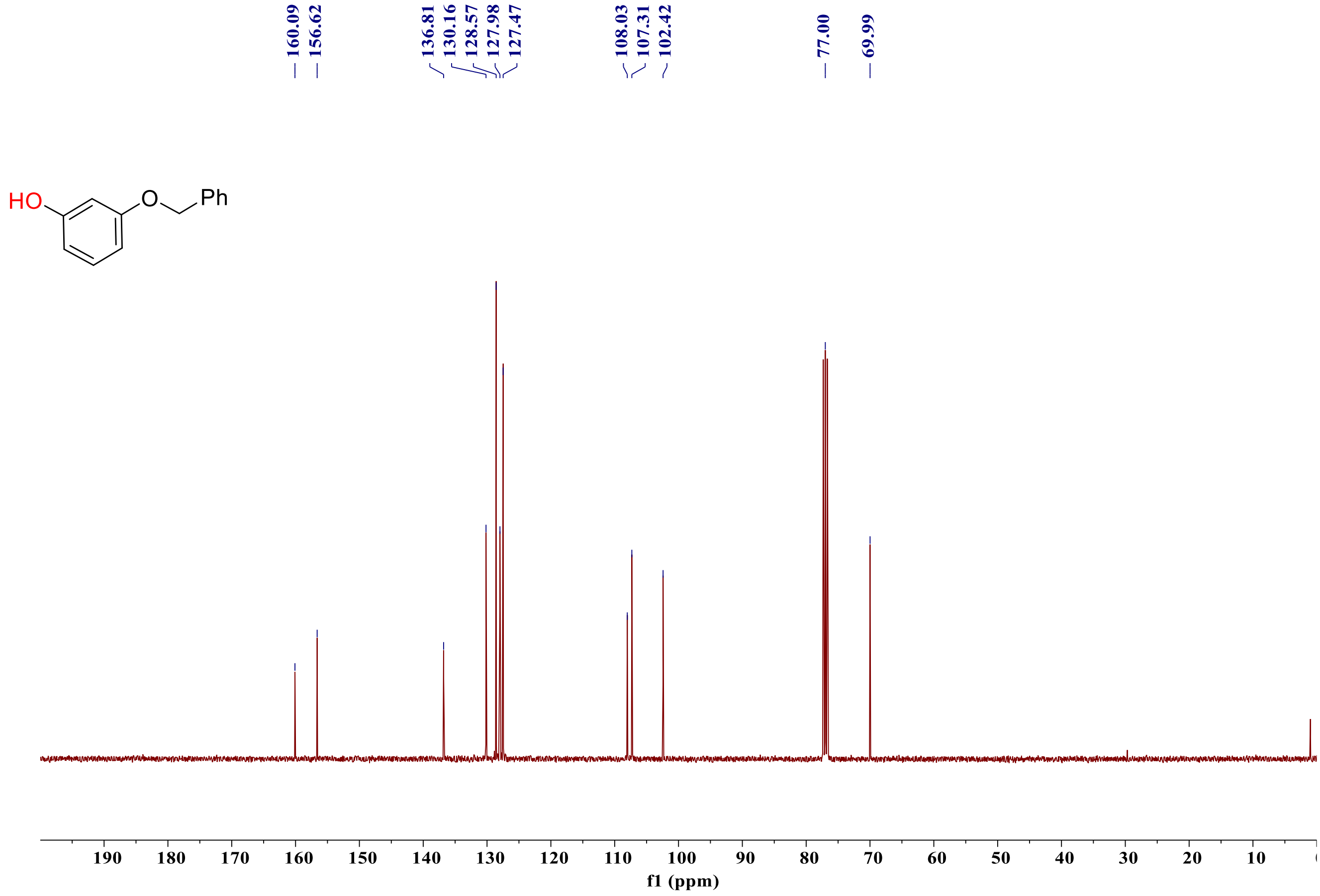

^{1}H NMR (400 MHz, $CDCl_3$), ^{13}C NMR (101 MHz, $CDCl_3$) of product 49

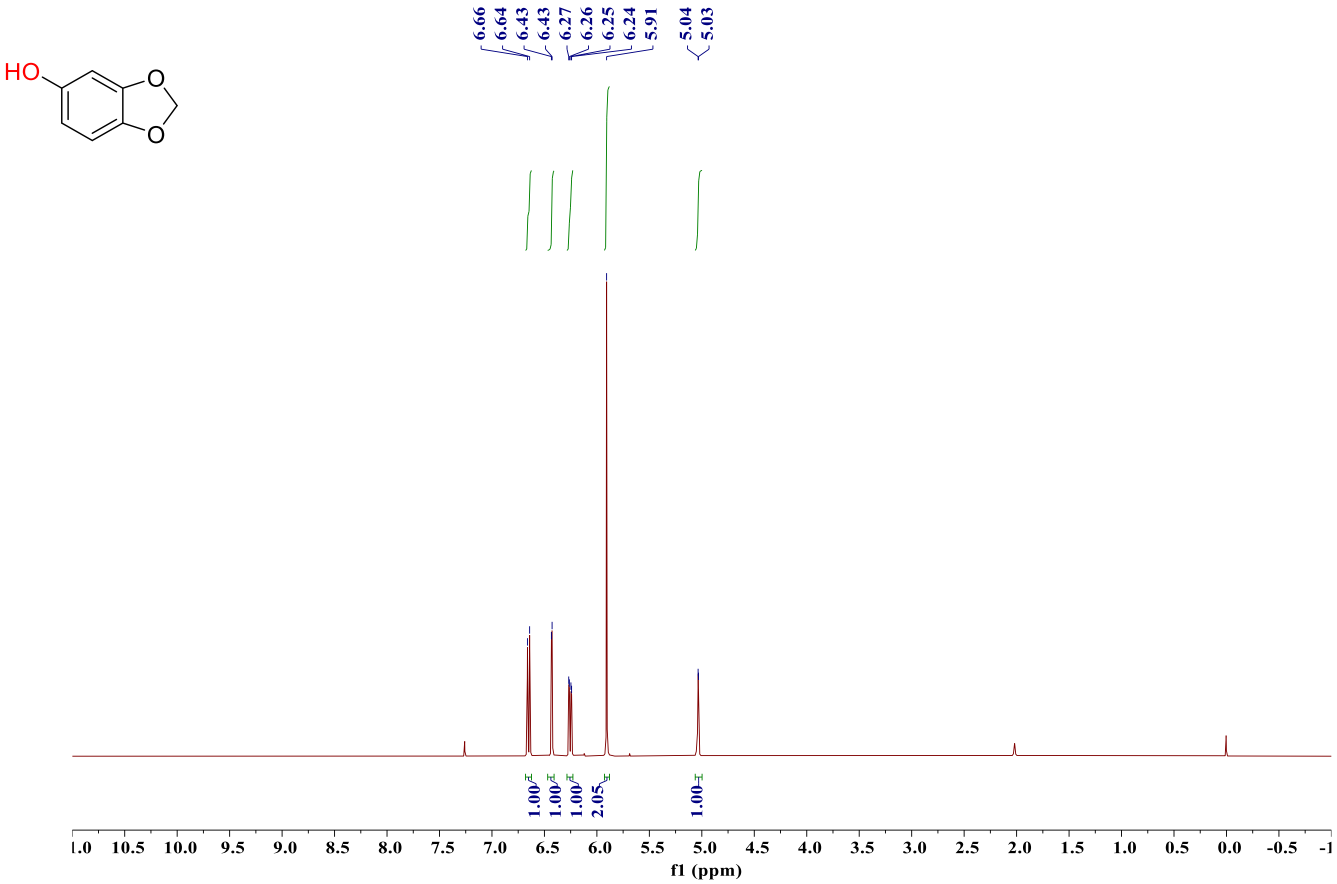

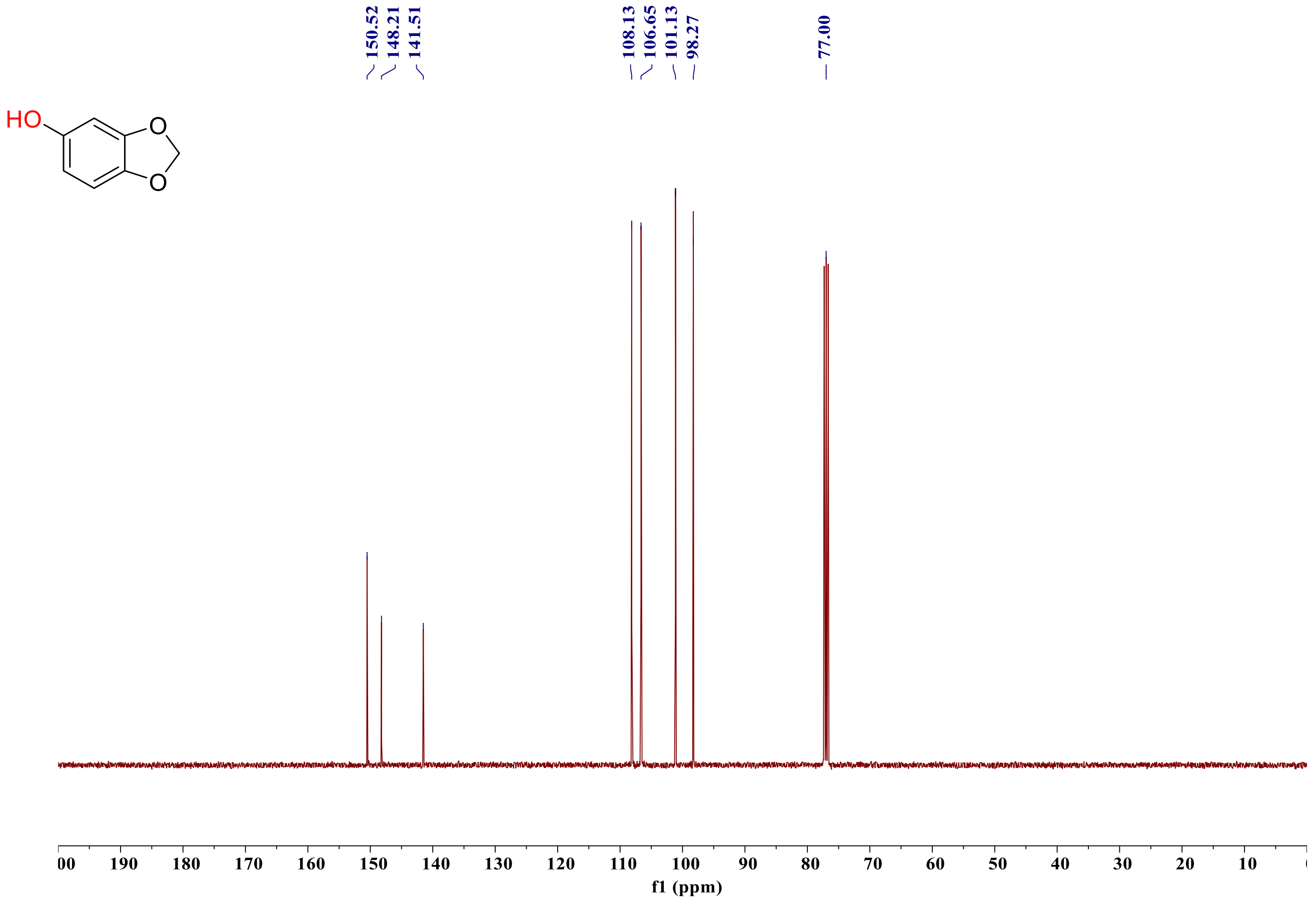

^{1}H NMR (400 MHz, CDCl$_3$), ^{13}C NMR (101 MHz, CDCl$_3$) of product 50

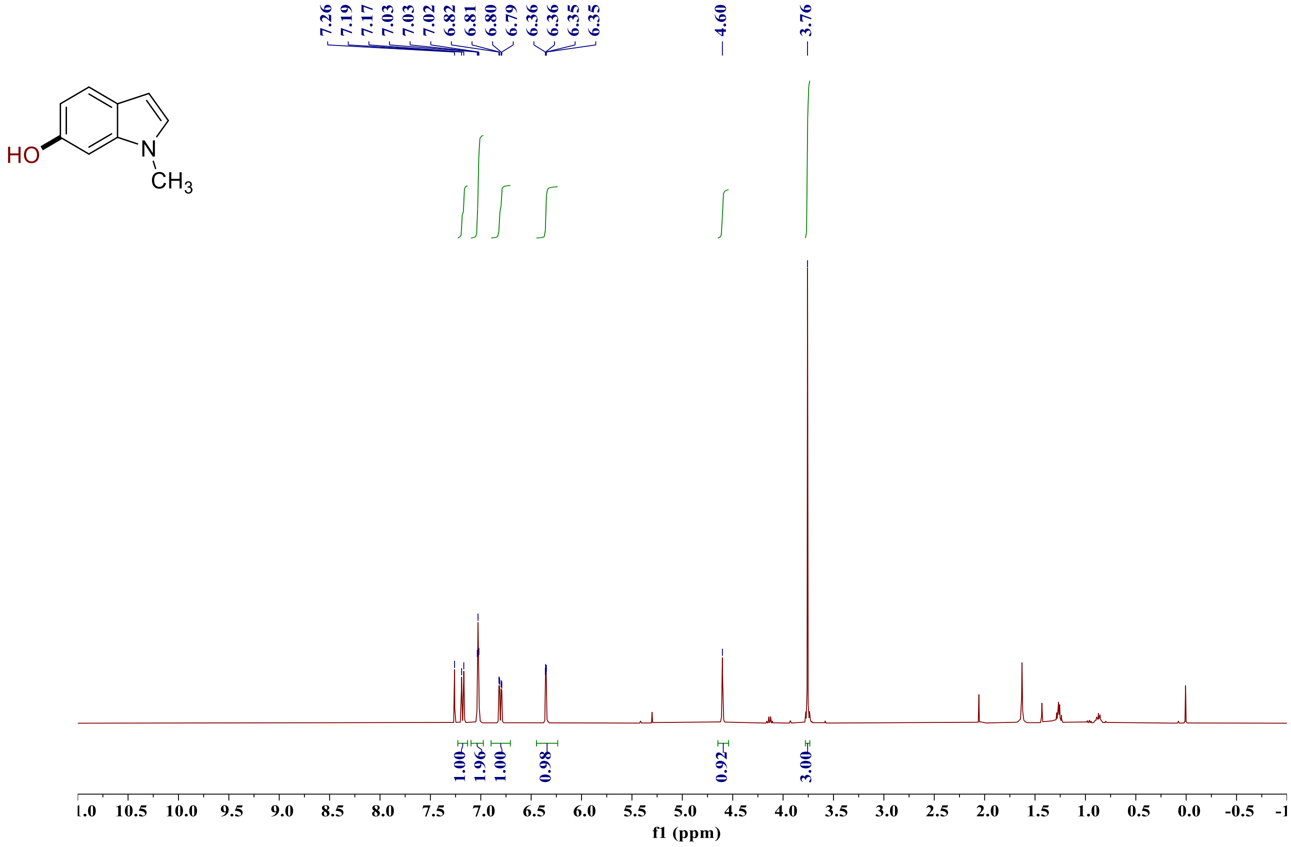

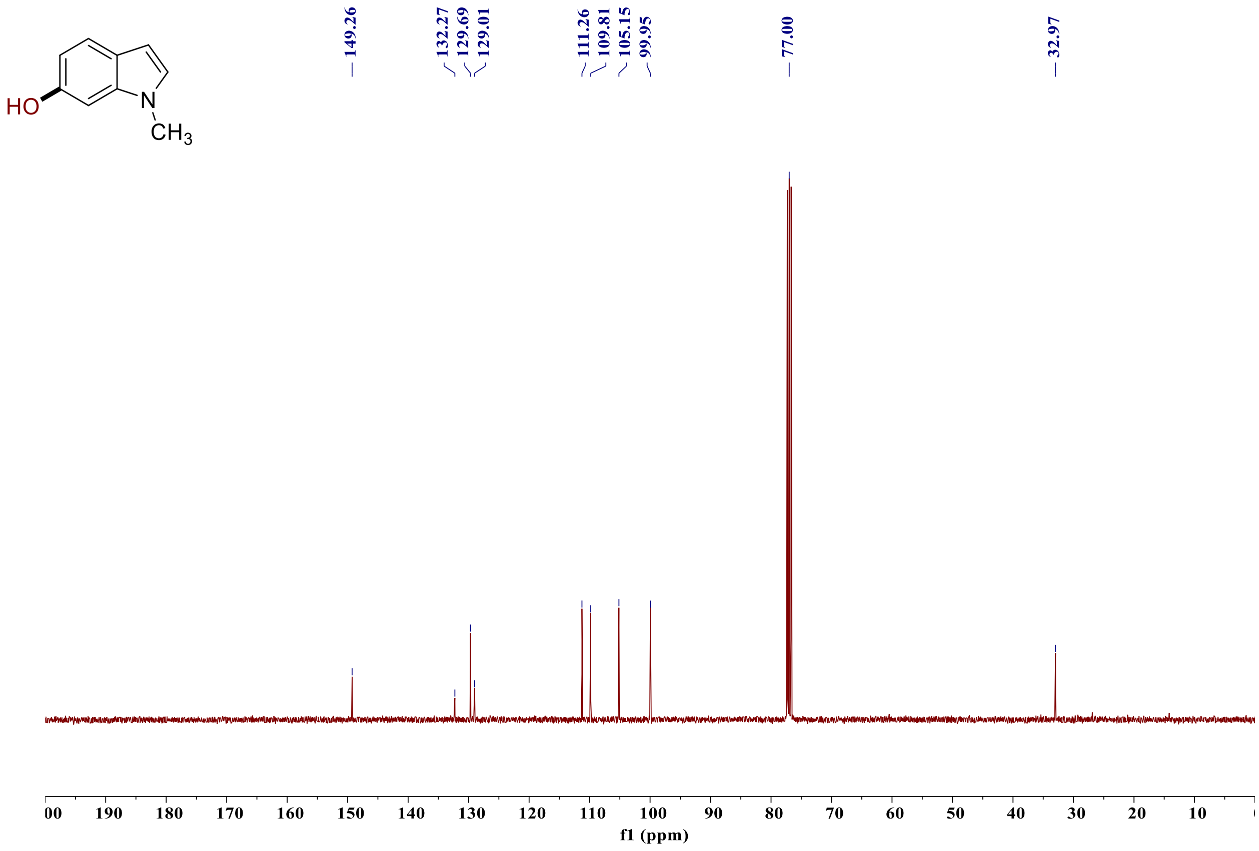

¹H NMR (400 MHz, CDCl$_3$), ¹³C NMR (101 MHz, CDCl$_3$) of product 51

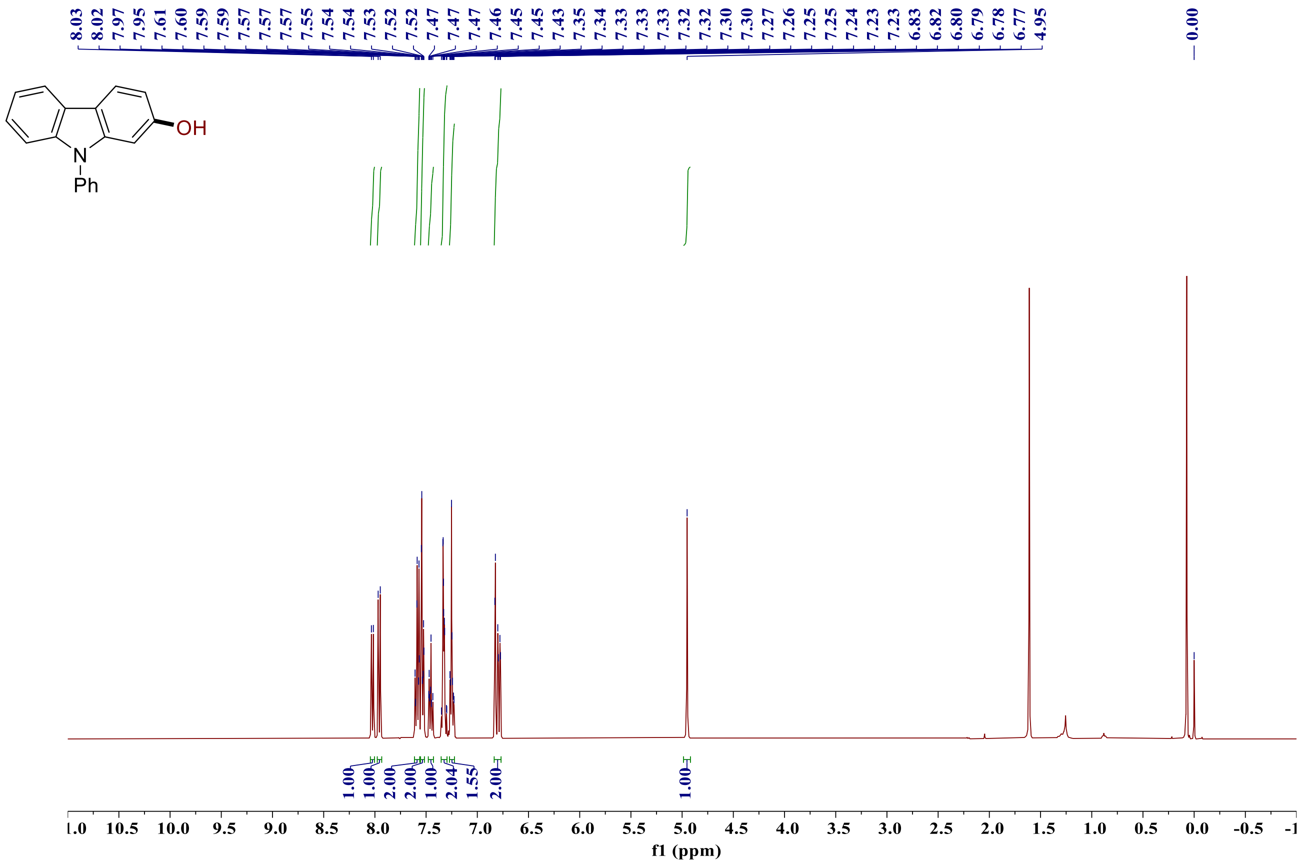

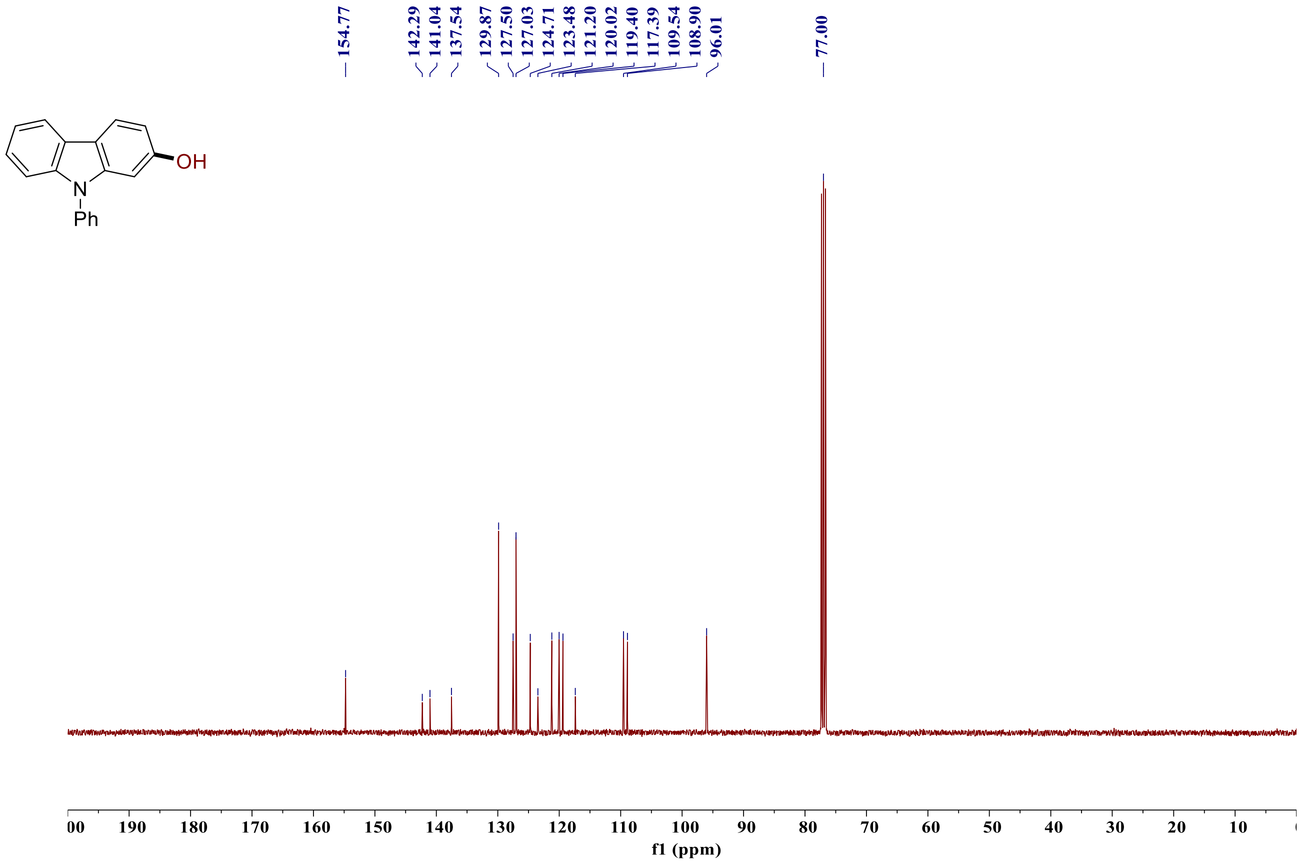

^1H NMR (400 MHz, $CDCl_3$), ^{13}C NMR (101 MHz, $CDCl_3$) of product 53

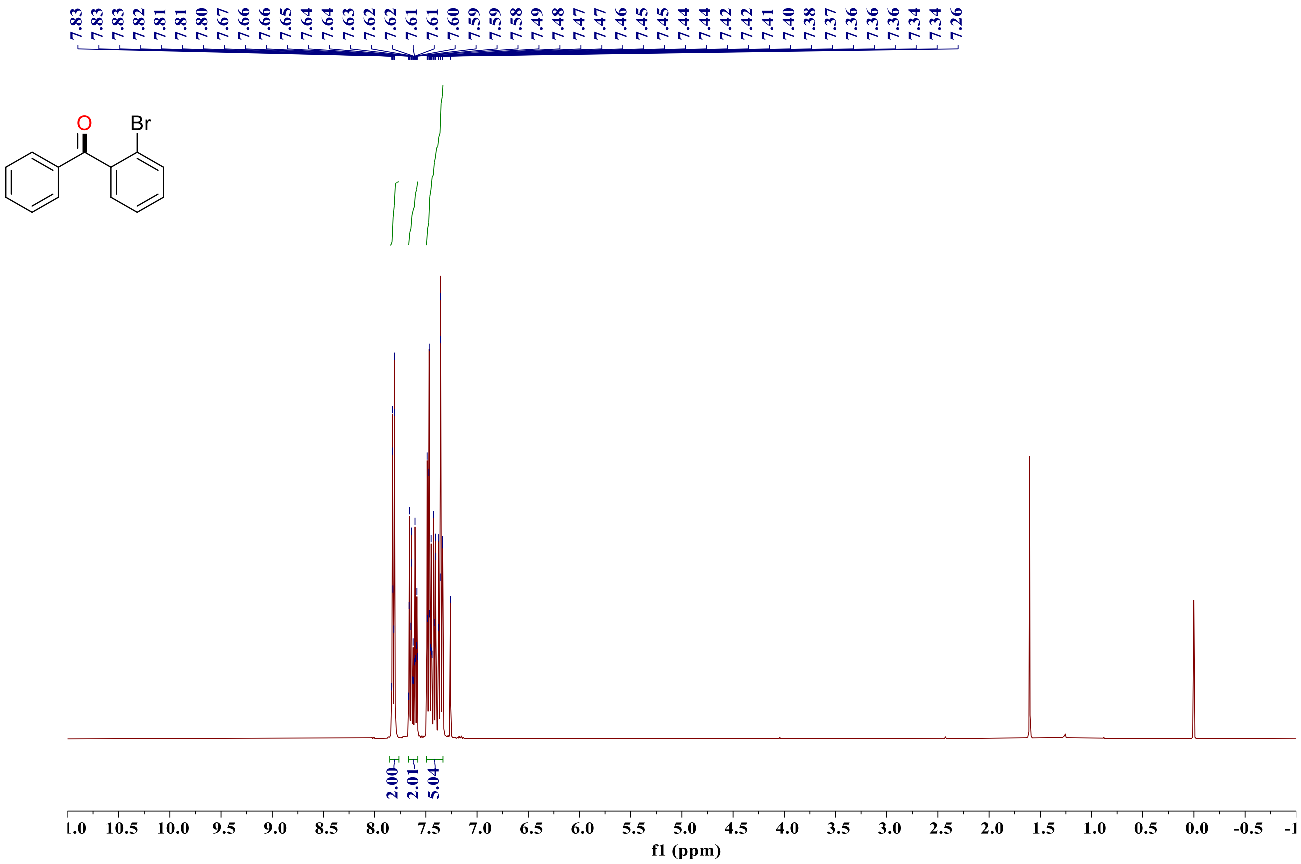

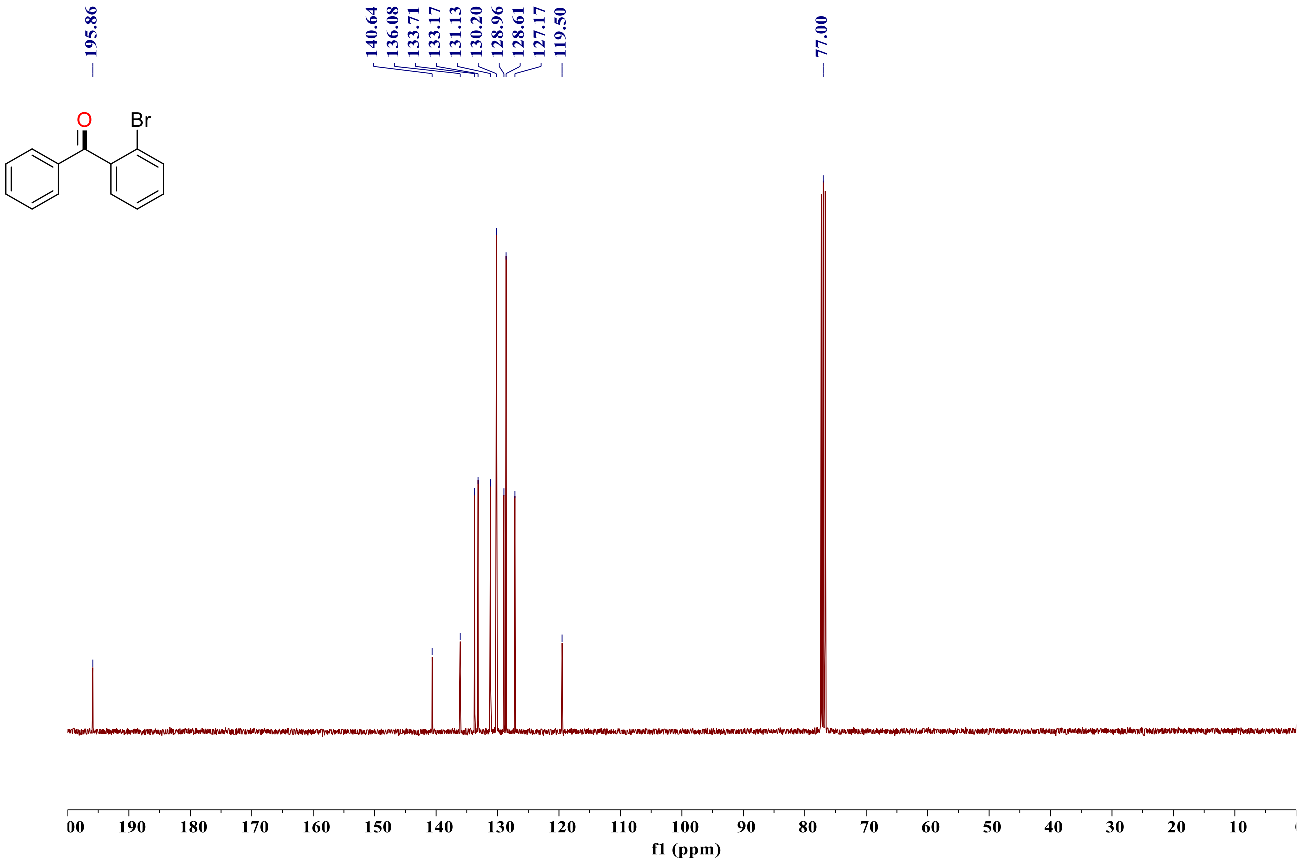

^{1}H NMR (400 MHz, $CDCl_3$), ^{13}C NMR (101 MHz, $CDCl_3$) of product 54

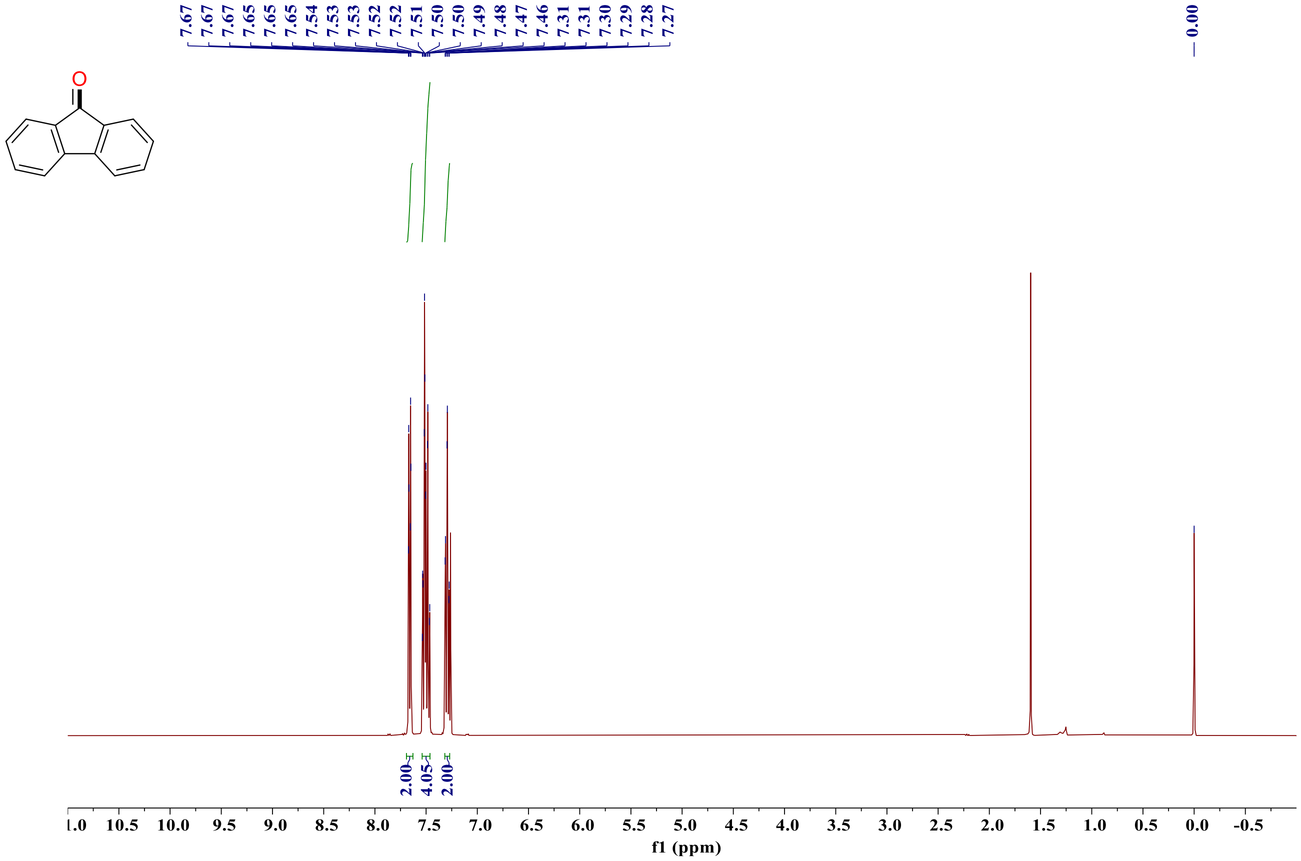

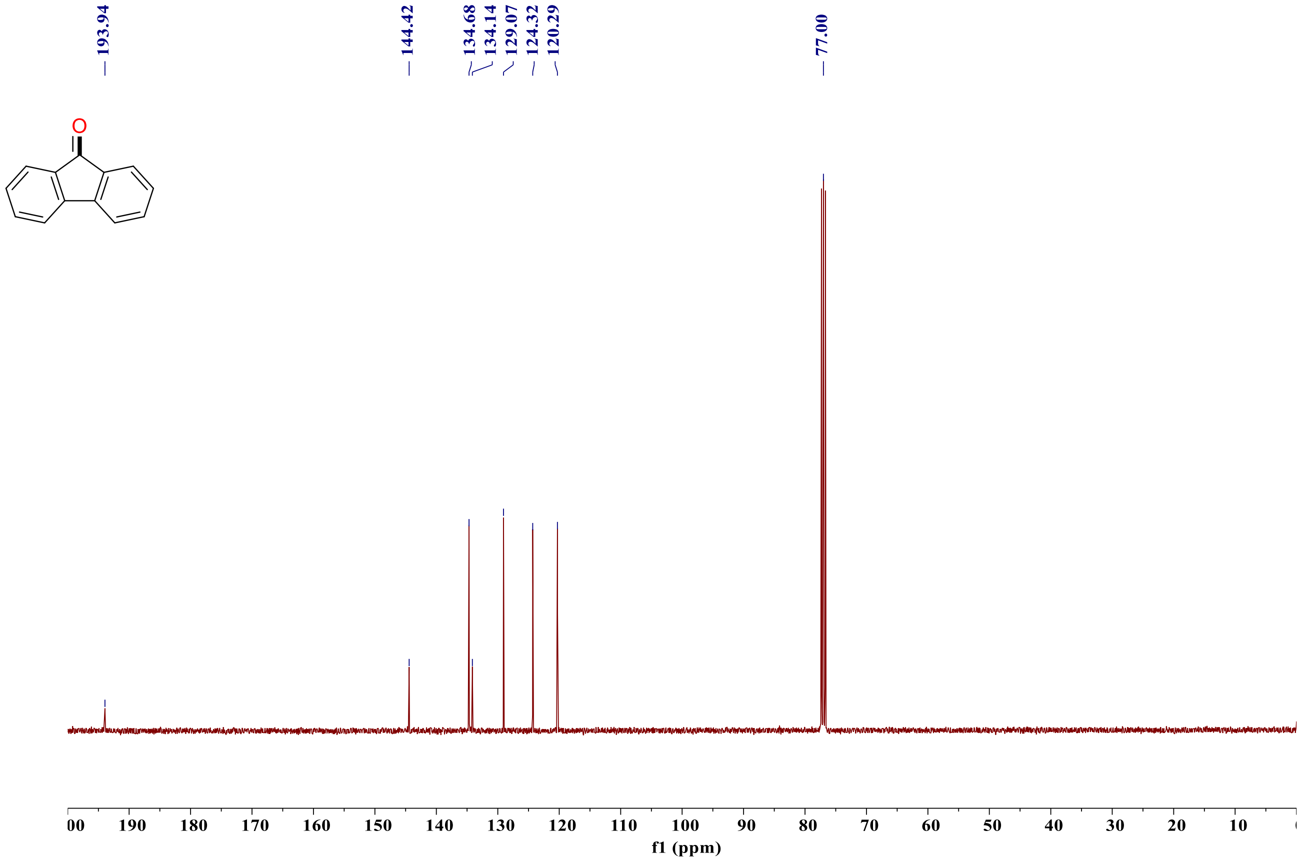

^{1}H NMR (400 MHz, $CDCl_3$), ^{13}C NMR (101 MHz, $CDCl_3$) of product 55

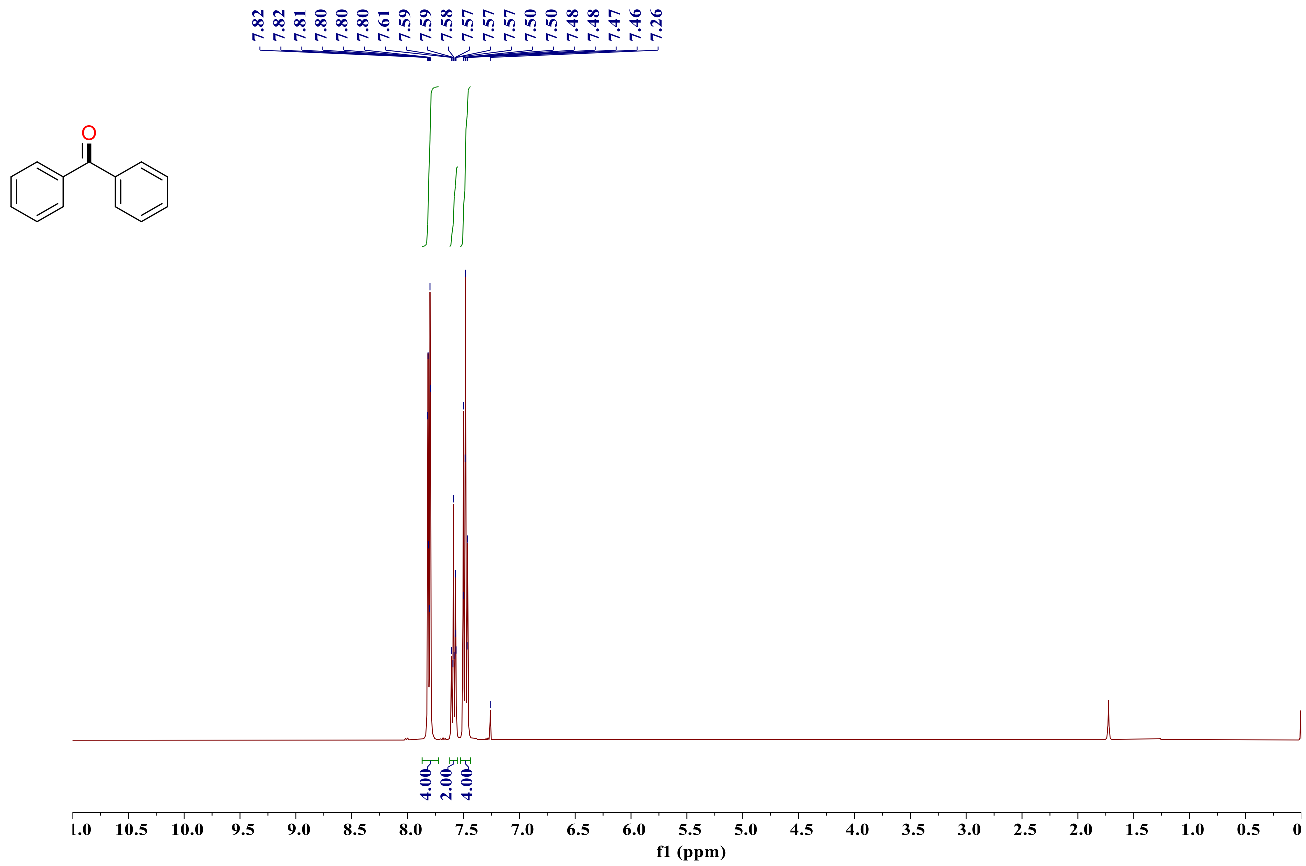

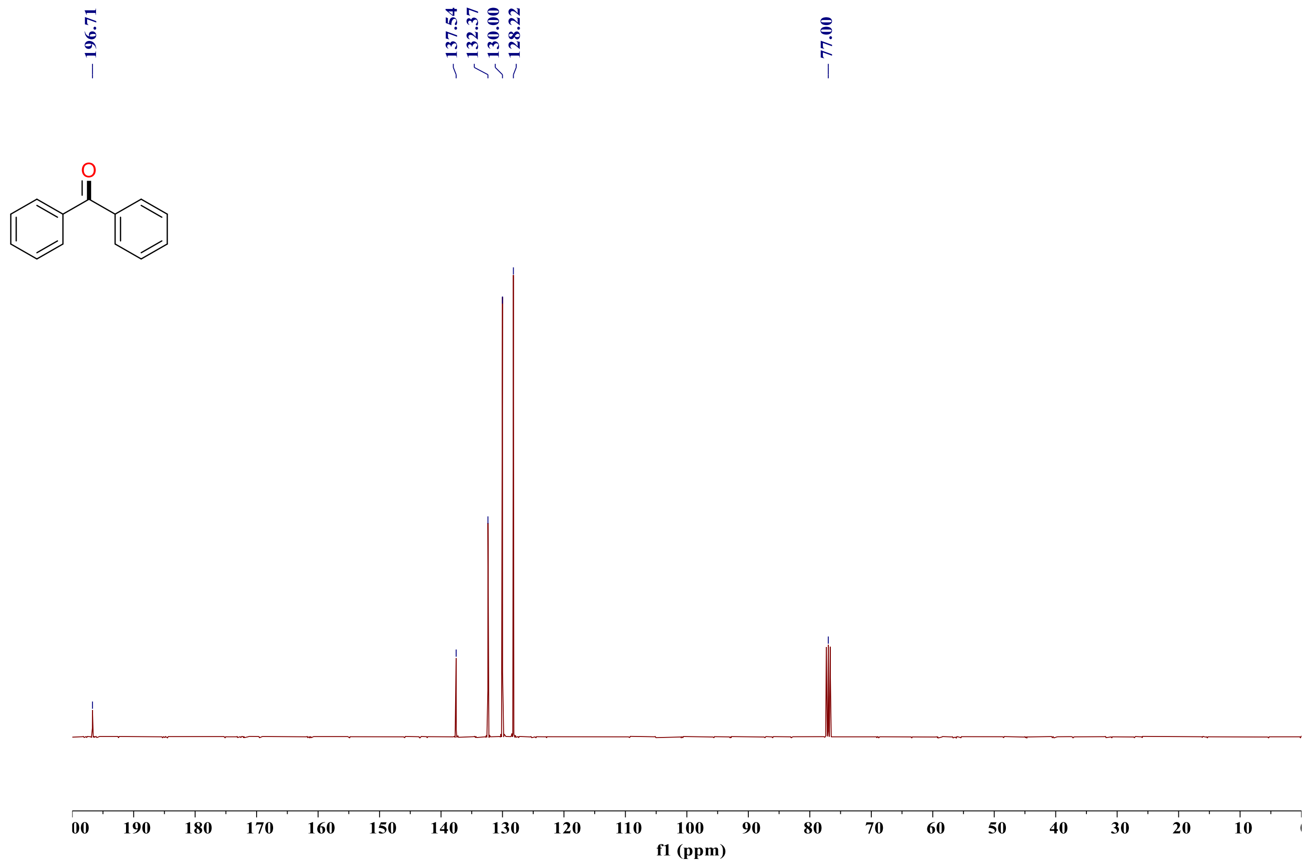

^{1}H NMR (400 MHz, $CDCl_3$), ^{13}C NMR (101 MHz, $CDCl_3$) of product 56

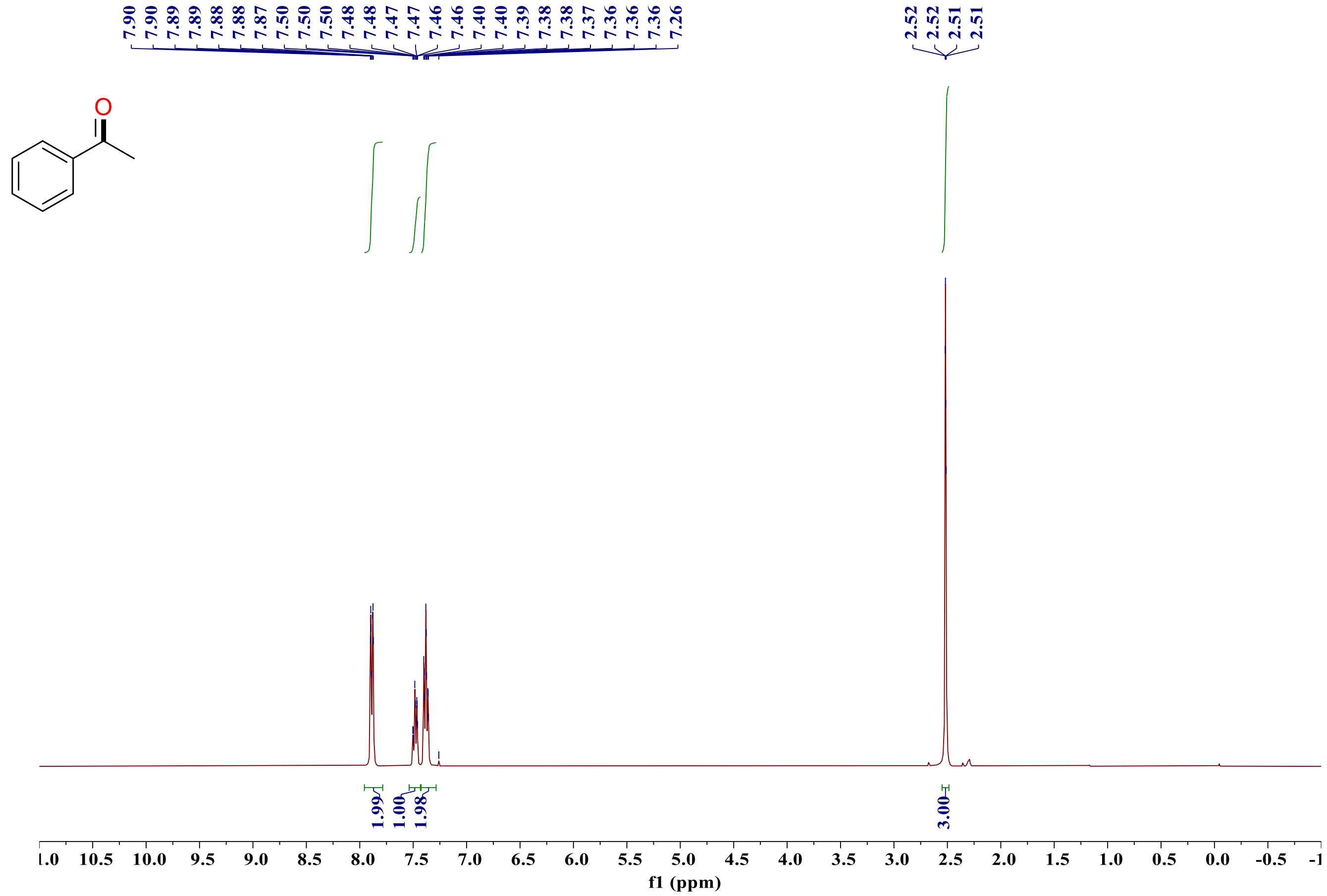

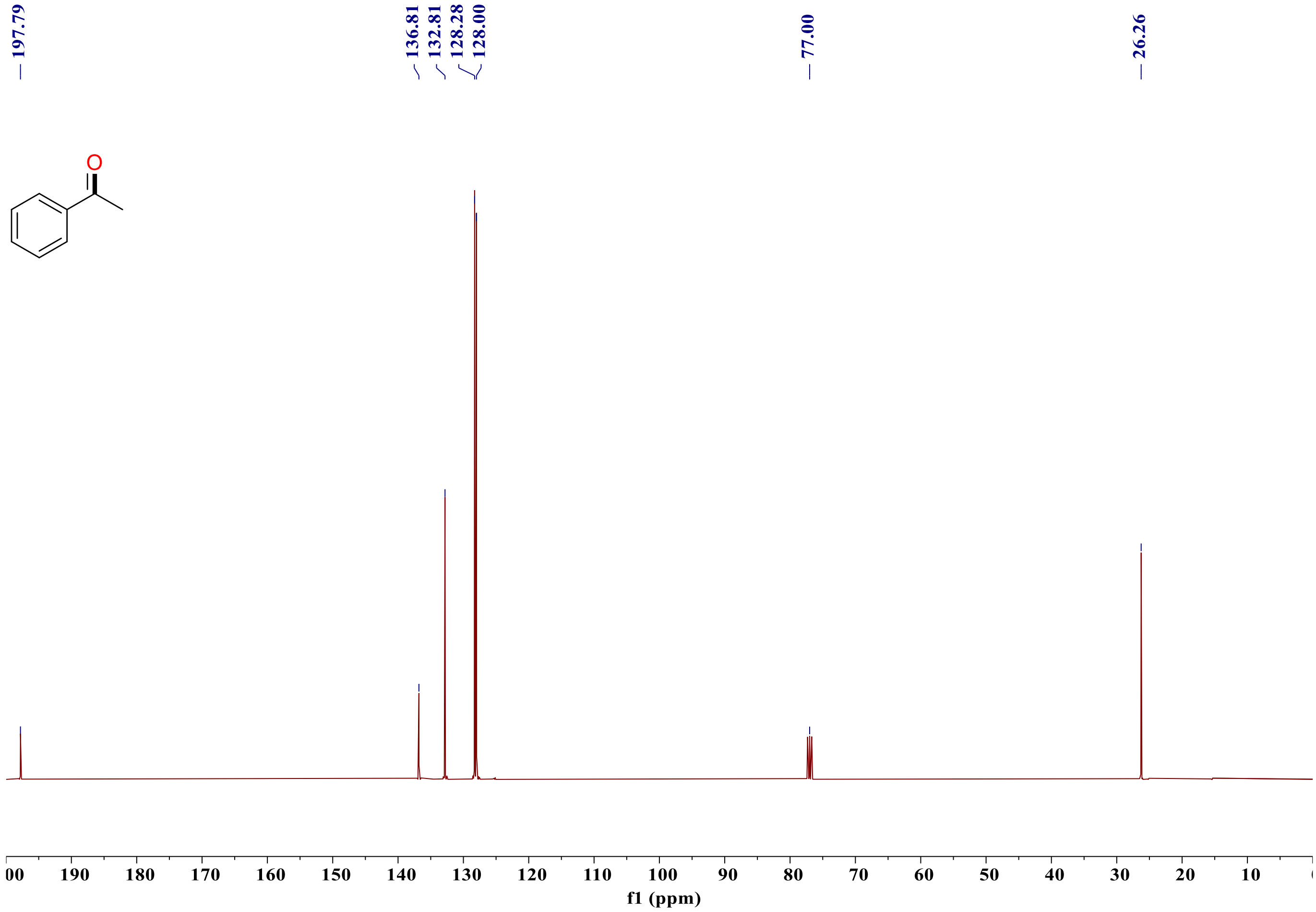

^{1}H NMR (400 MHz, $CDCl_3$), ^{13}C NMR (101 MHz, $CDCl_3$) of product 57

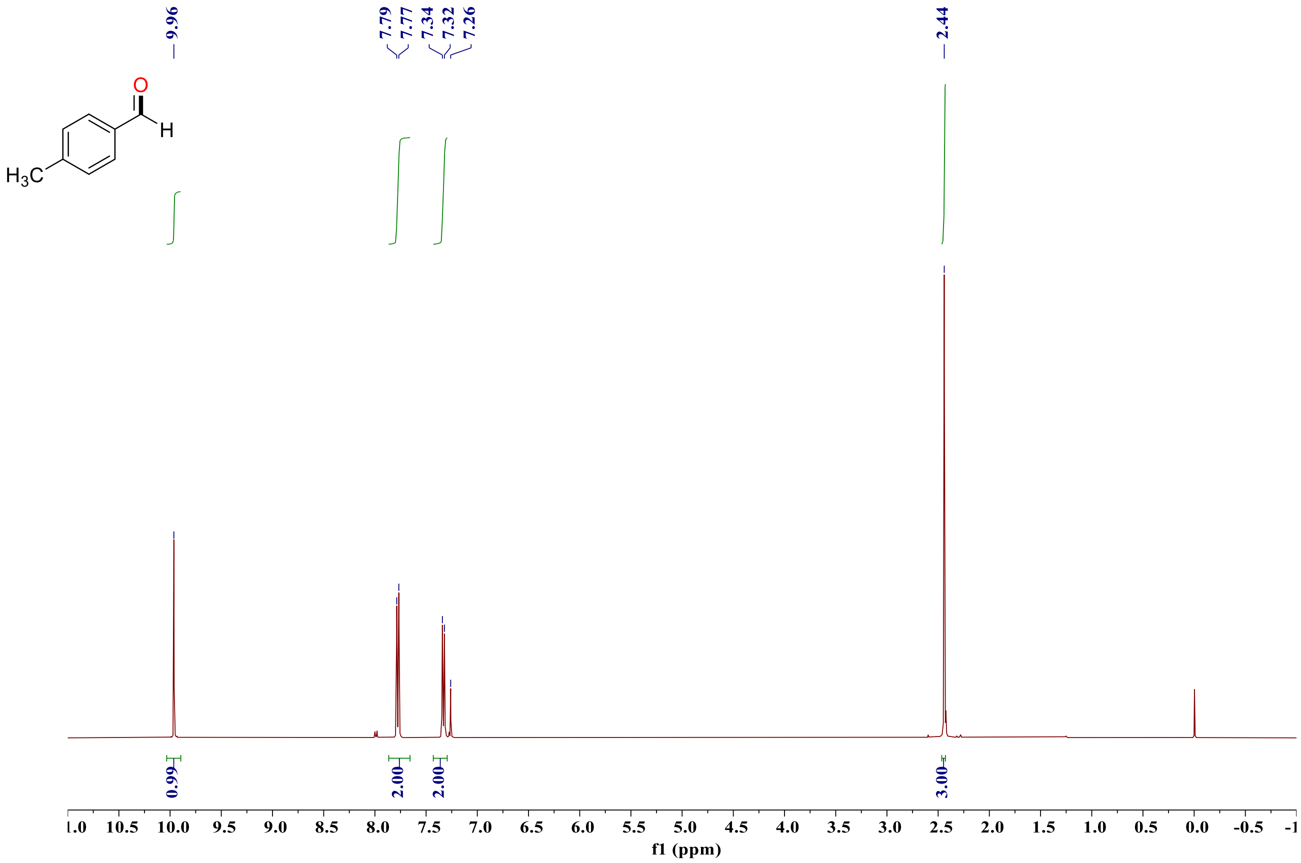

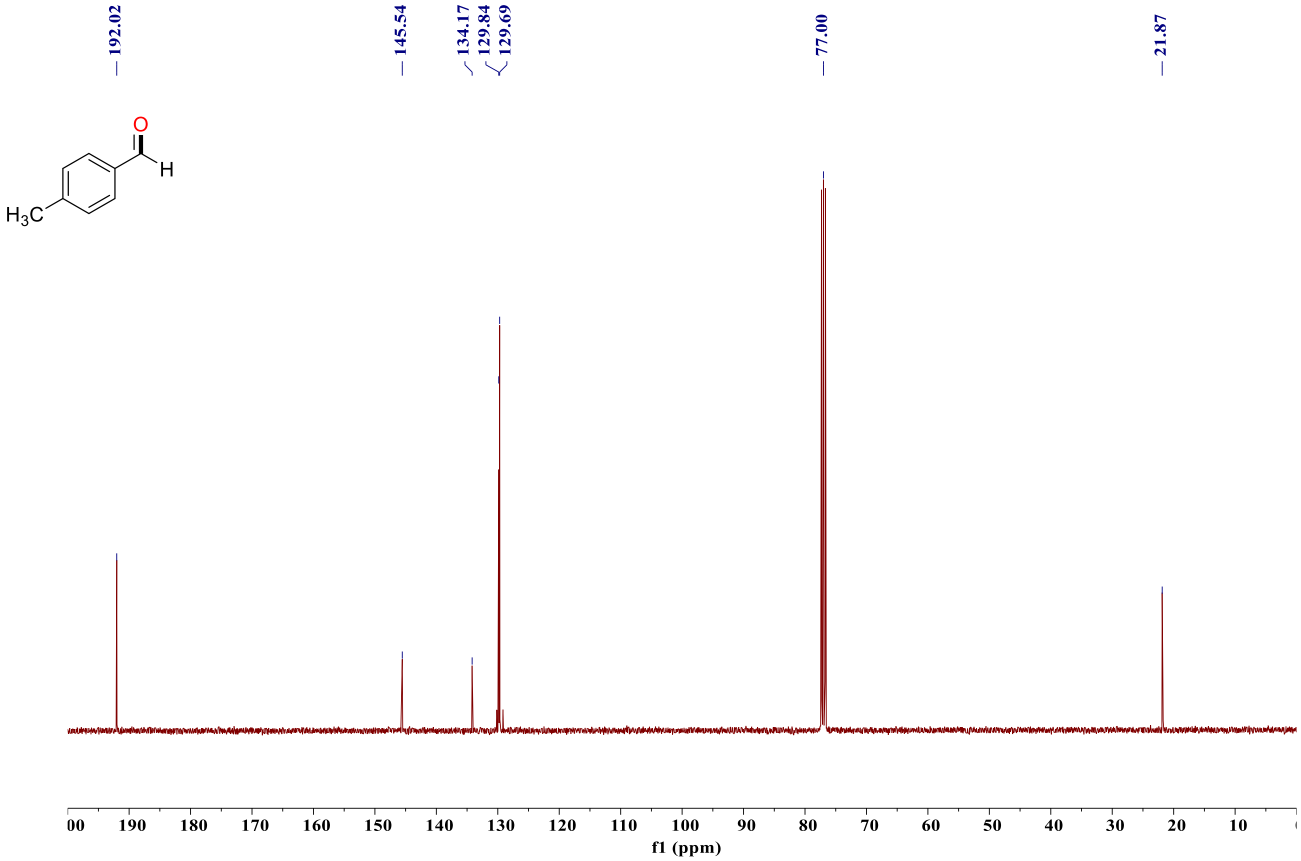

^{1}H NMR (400 MHz, $CDCl_3$), ^{13}C NMR (101 MHz, $CDCl_3$) of product 58

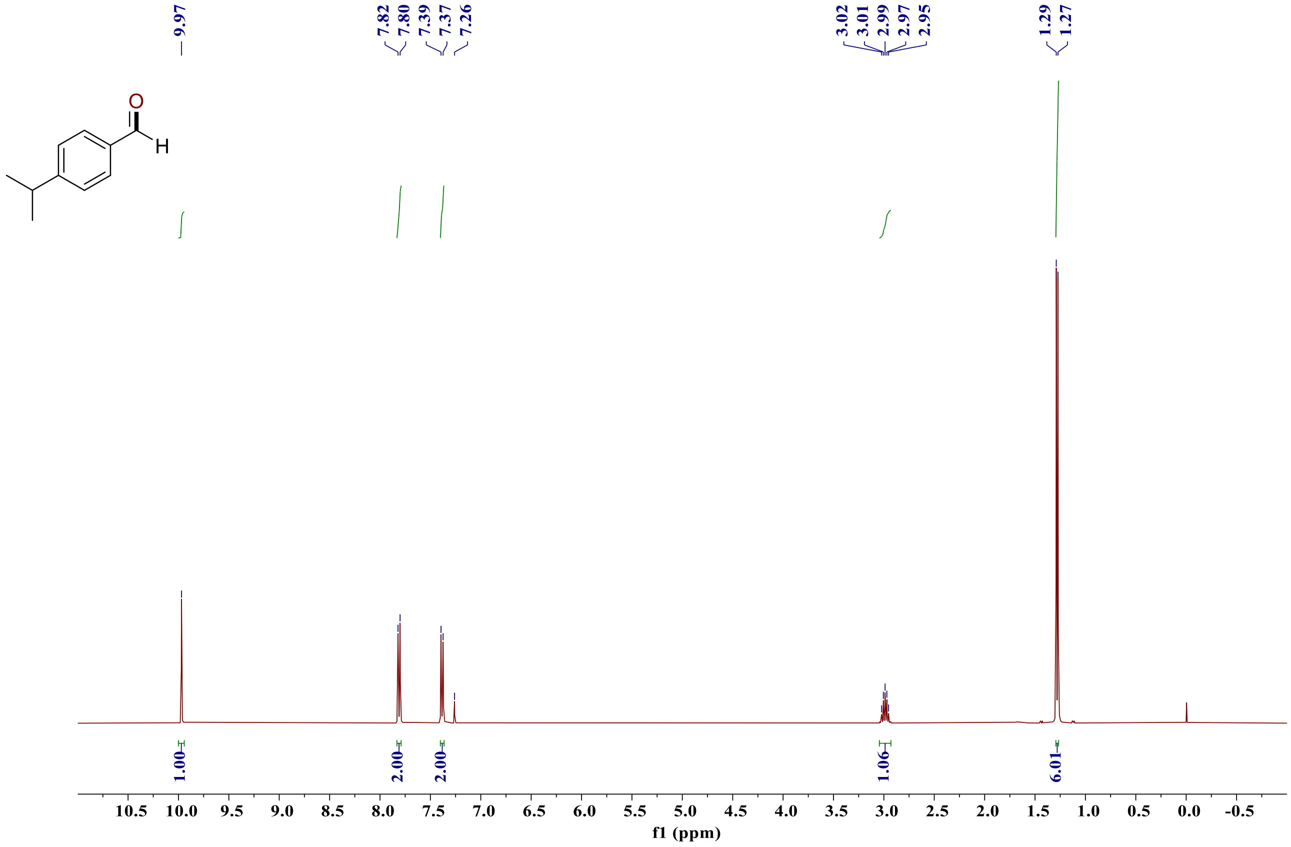

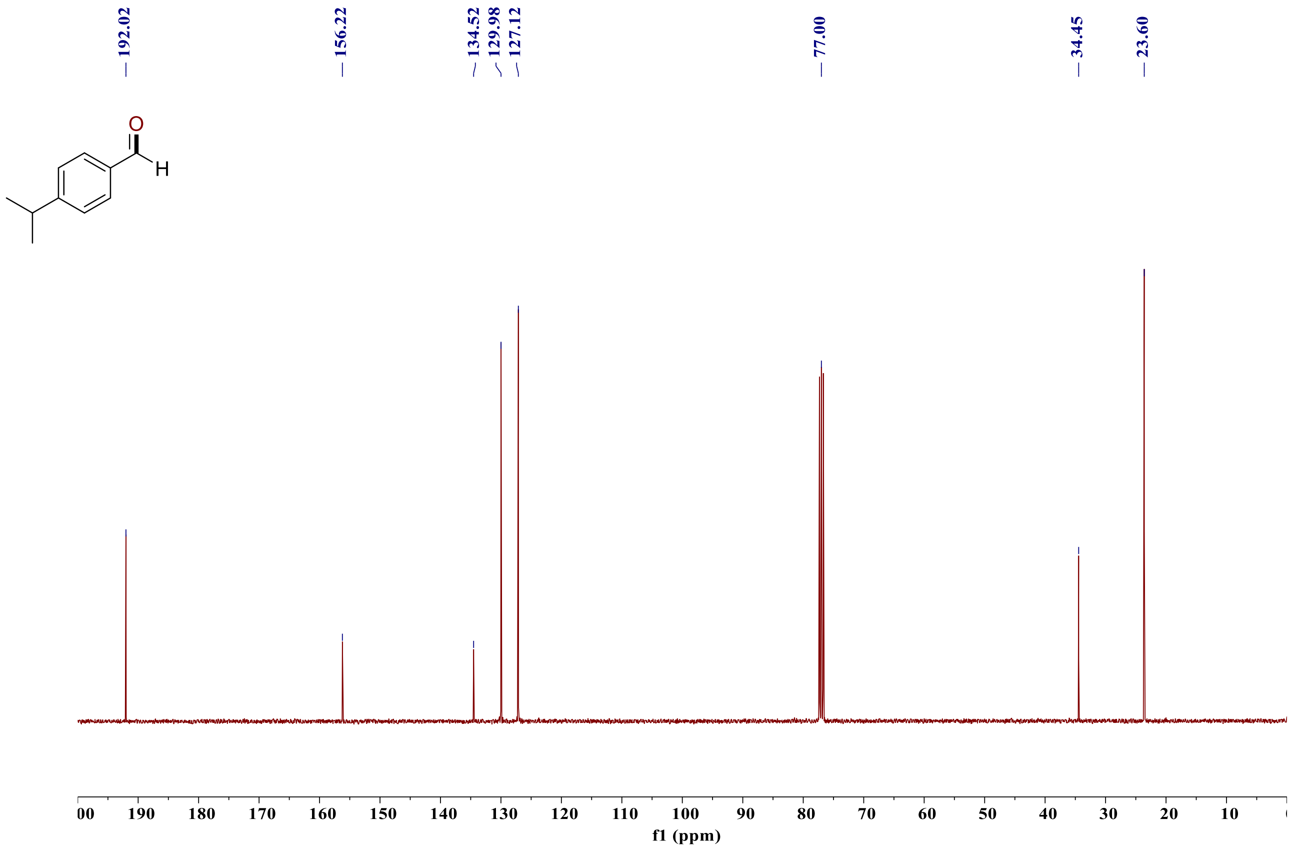

^{1}H NMR (400 MHz, $CDCl_3$), ^{13}C NMR (101 MHz, $CDCl_3$) of product 59

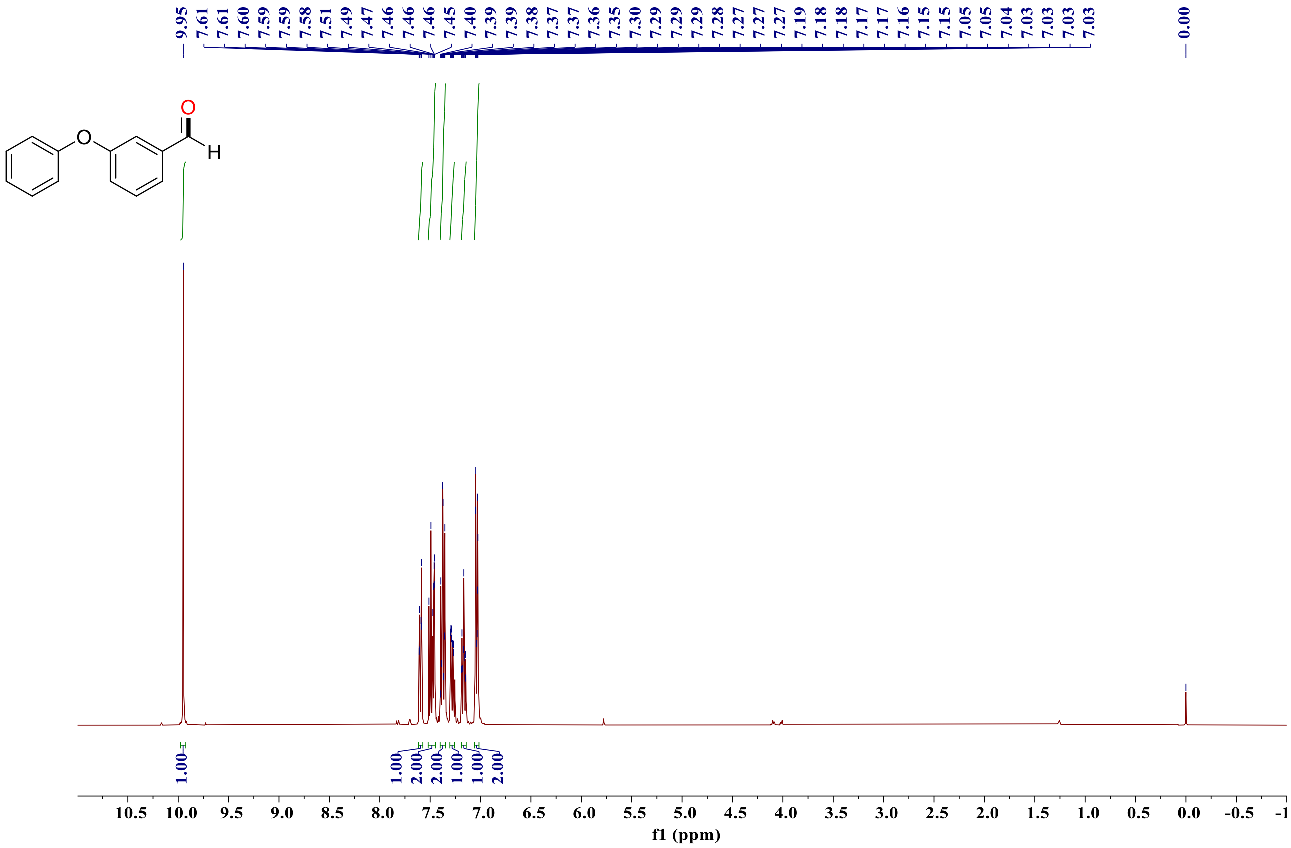

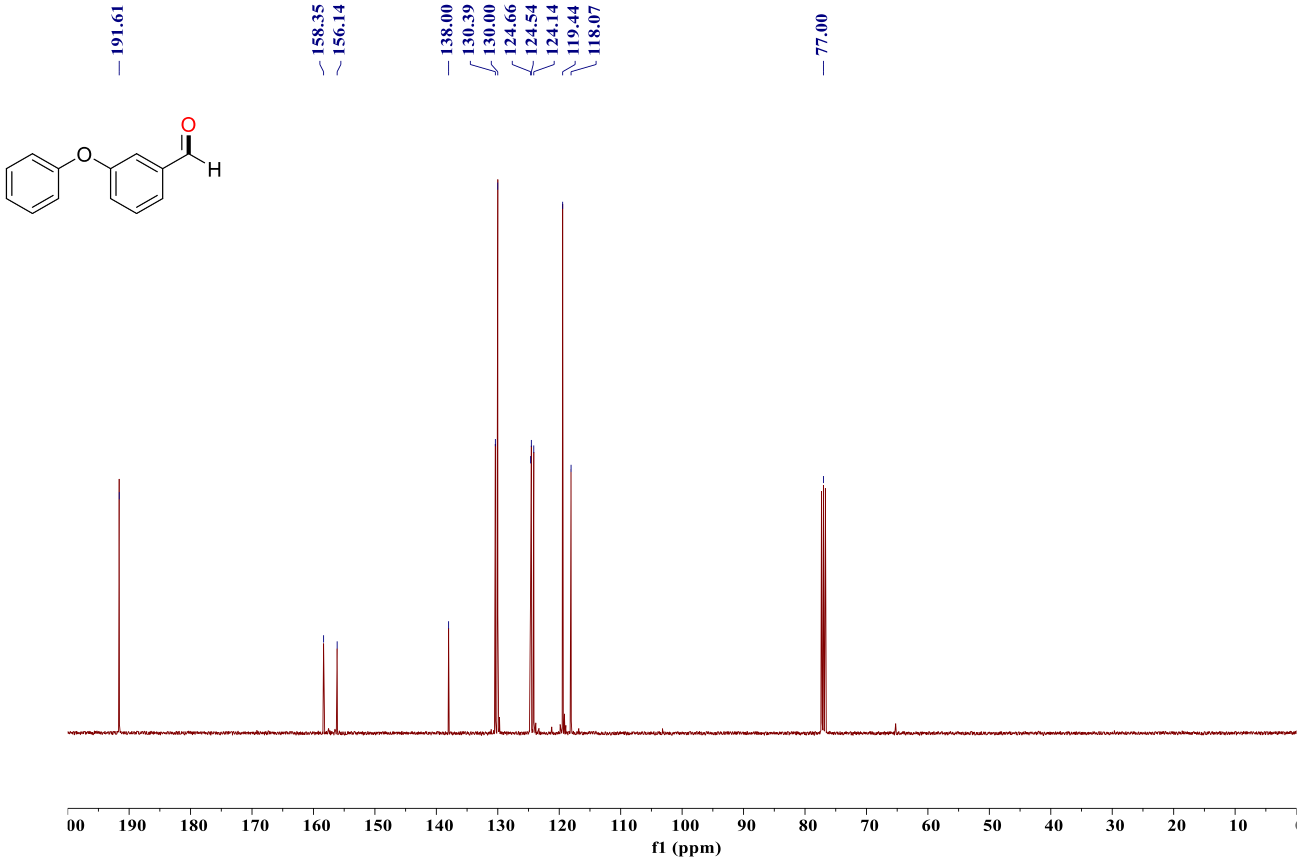

^{1}H NMR (400 MHz, $CDCl_3$), ^{13}C NMR (101 MHz, $CDCl_3$) of product 60

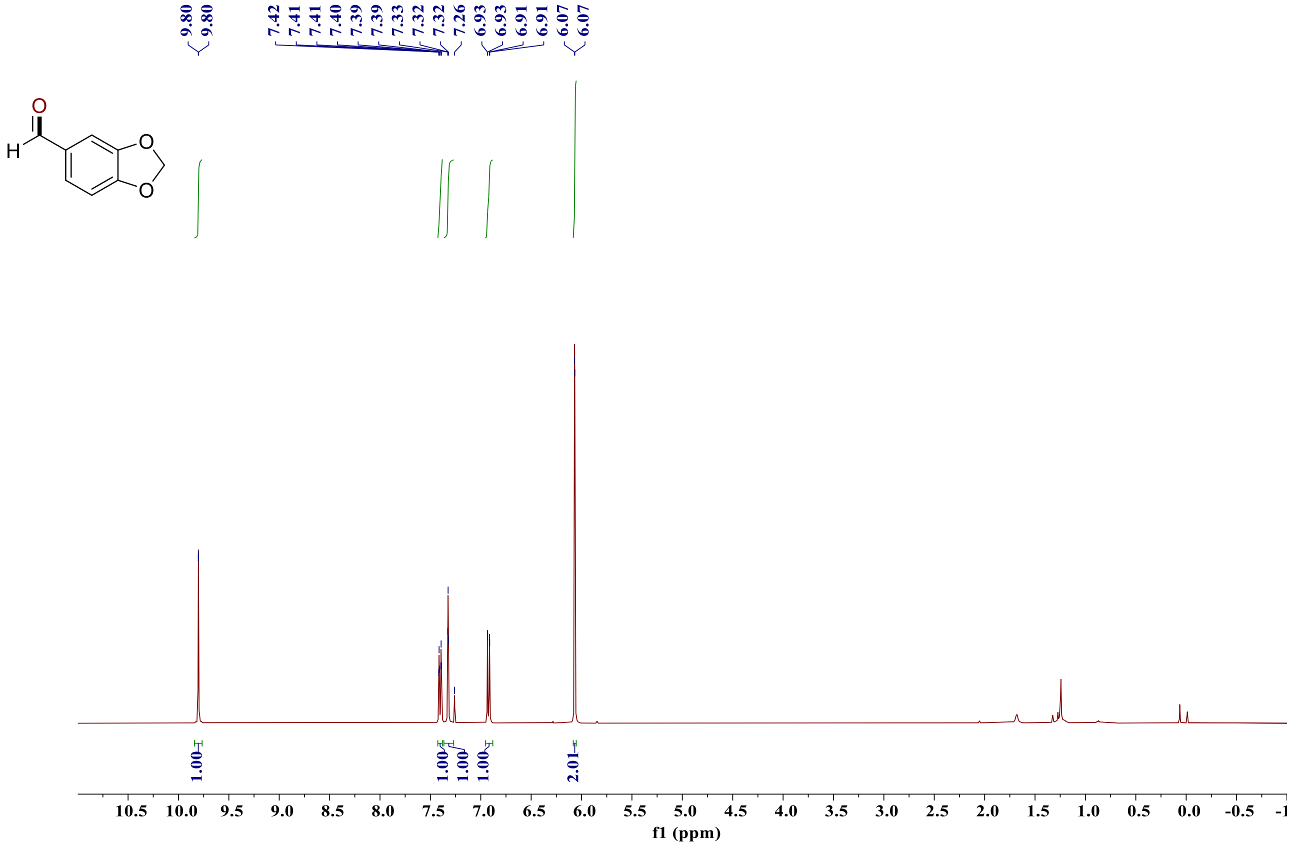

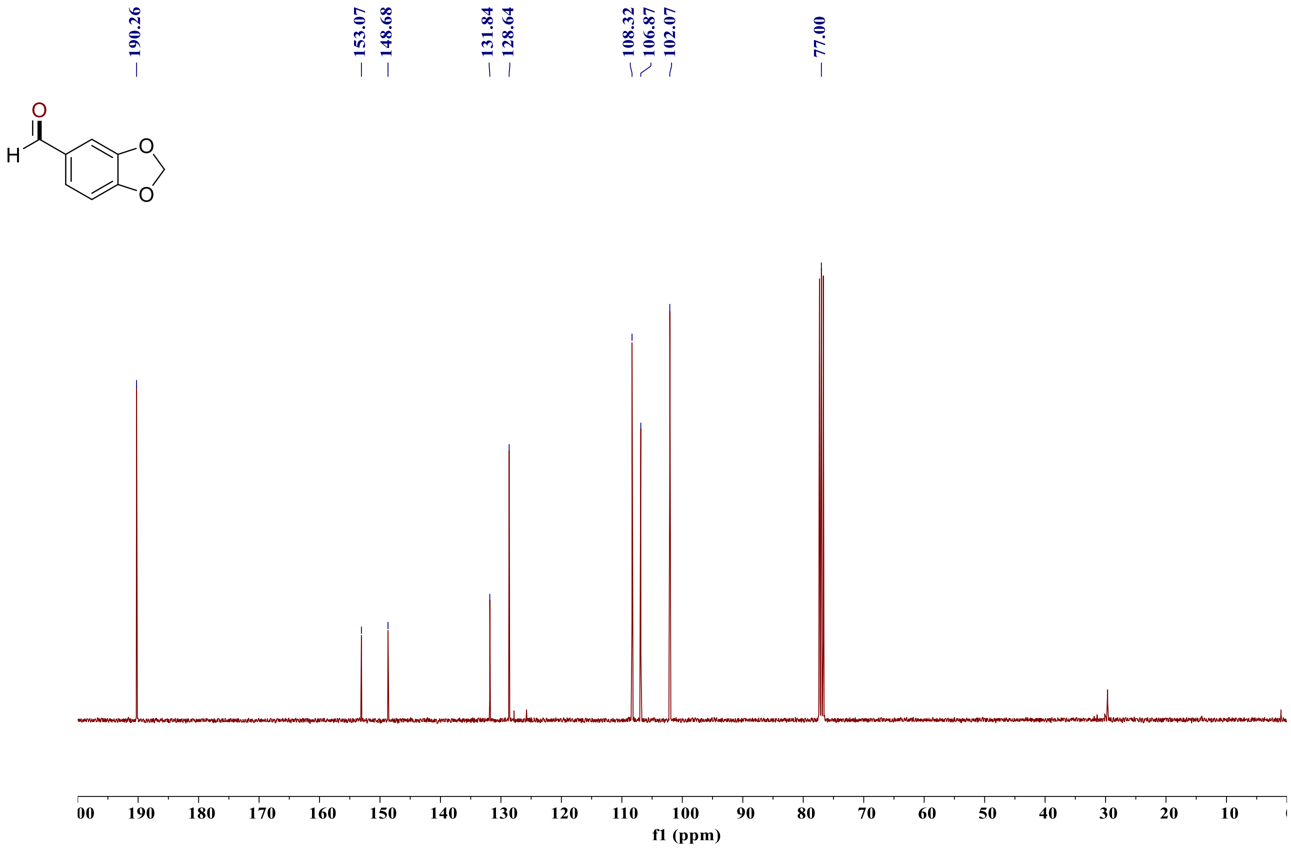

^{1}H NMR (400 MHz, $CDCl_3$), ^{13}C NMR (101 MHz, $CDCl_3$) of product 61

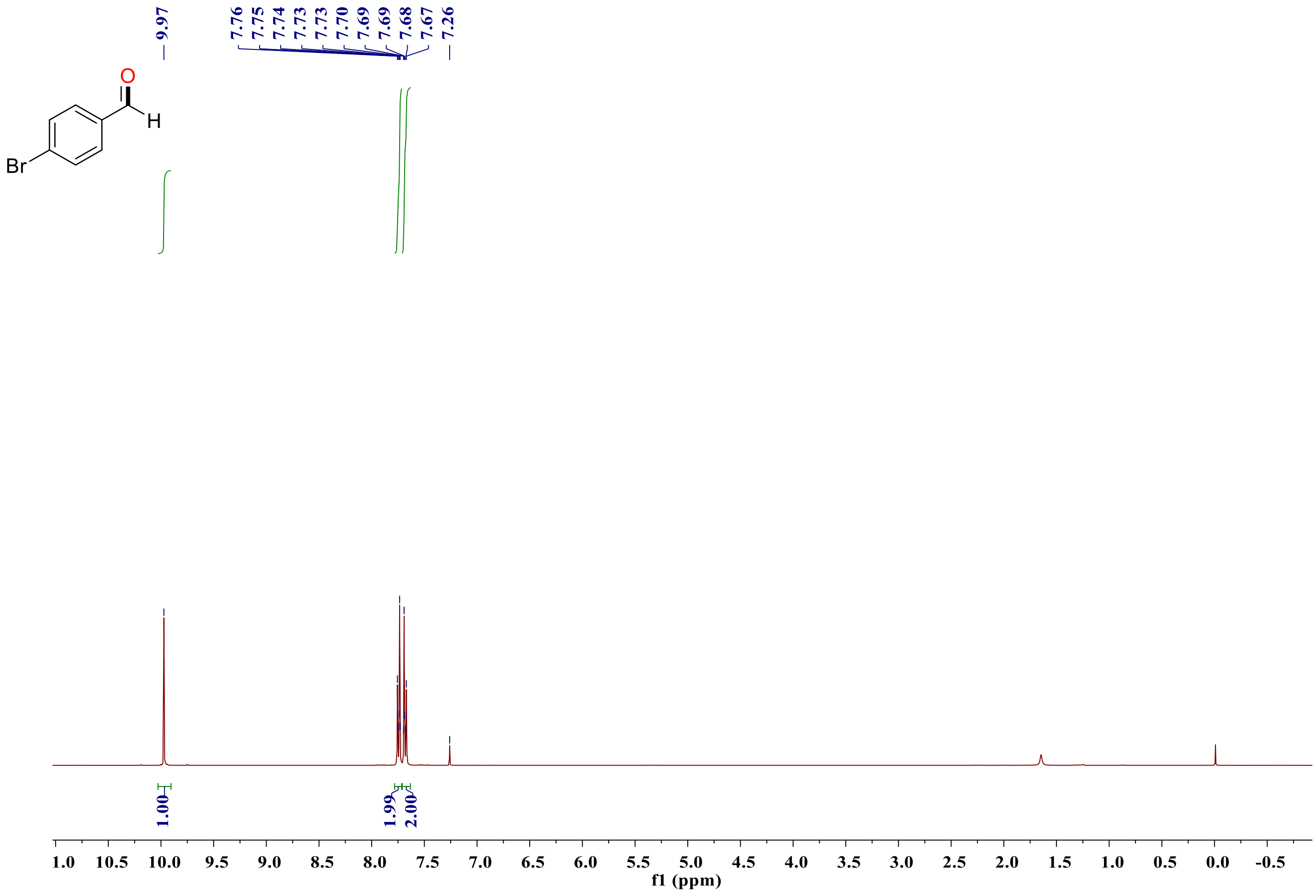

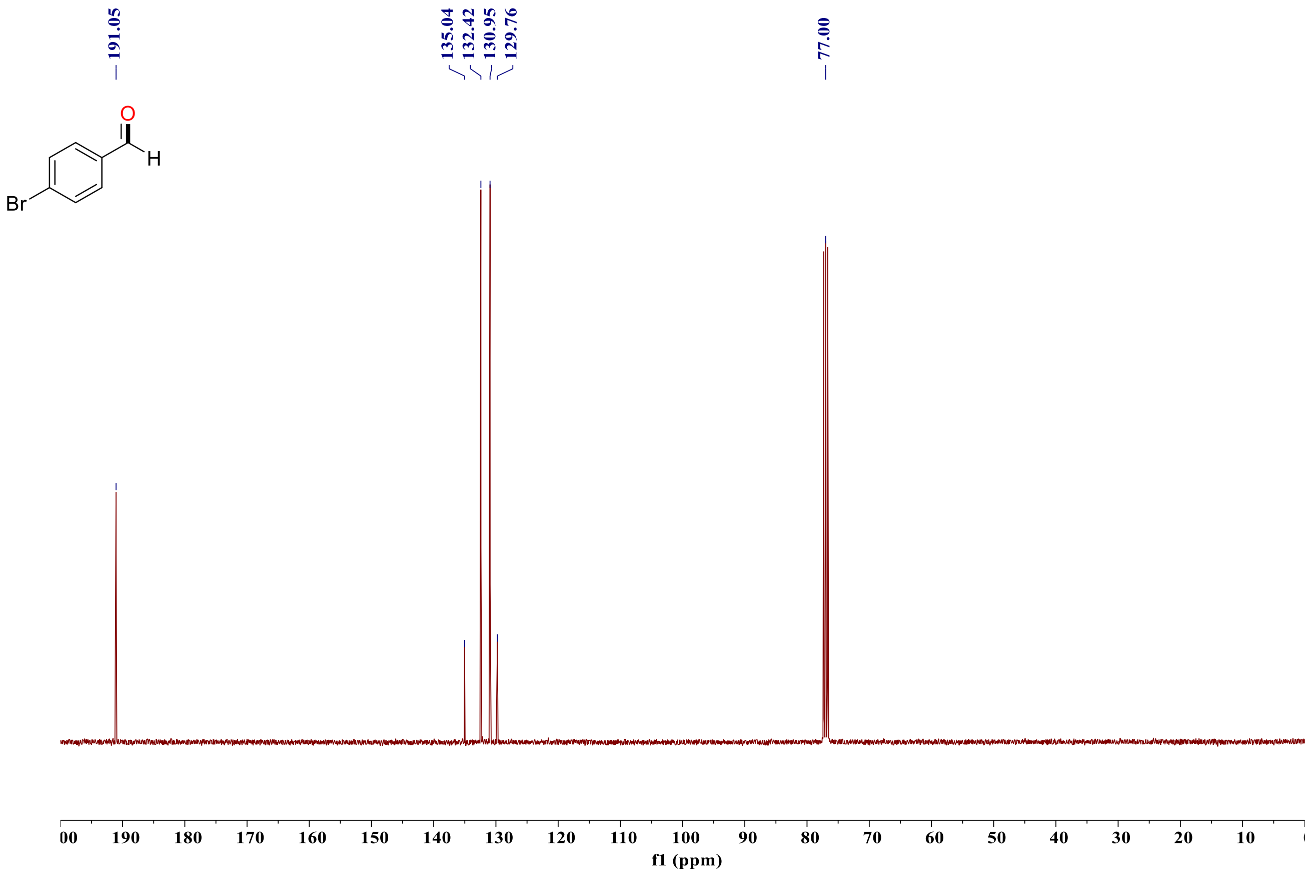

^{1}H NMR (400 MHz, $CDCl_3$), ^{13}C NMR (101 MHz, $CDCl_3$) of product 62

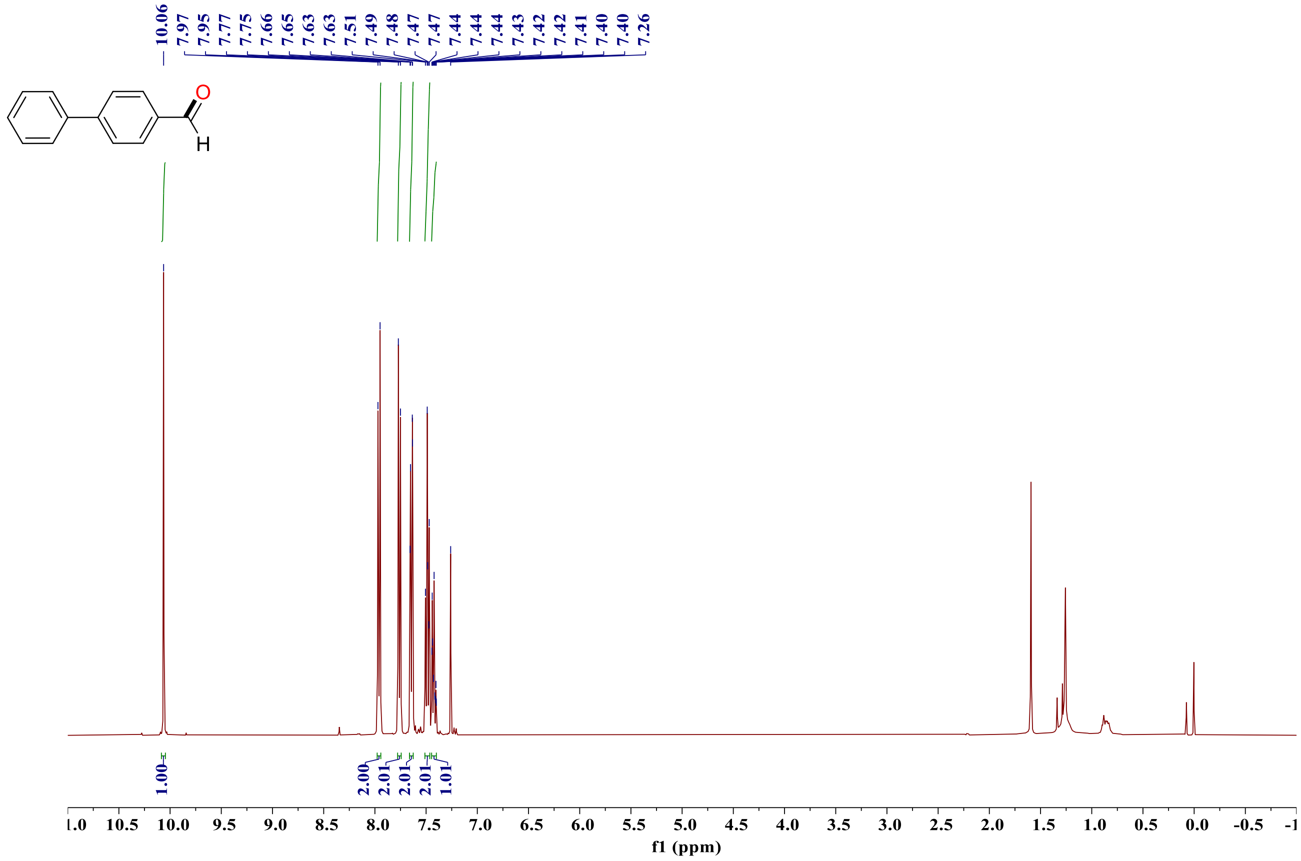

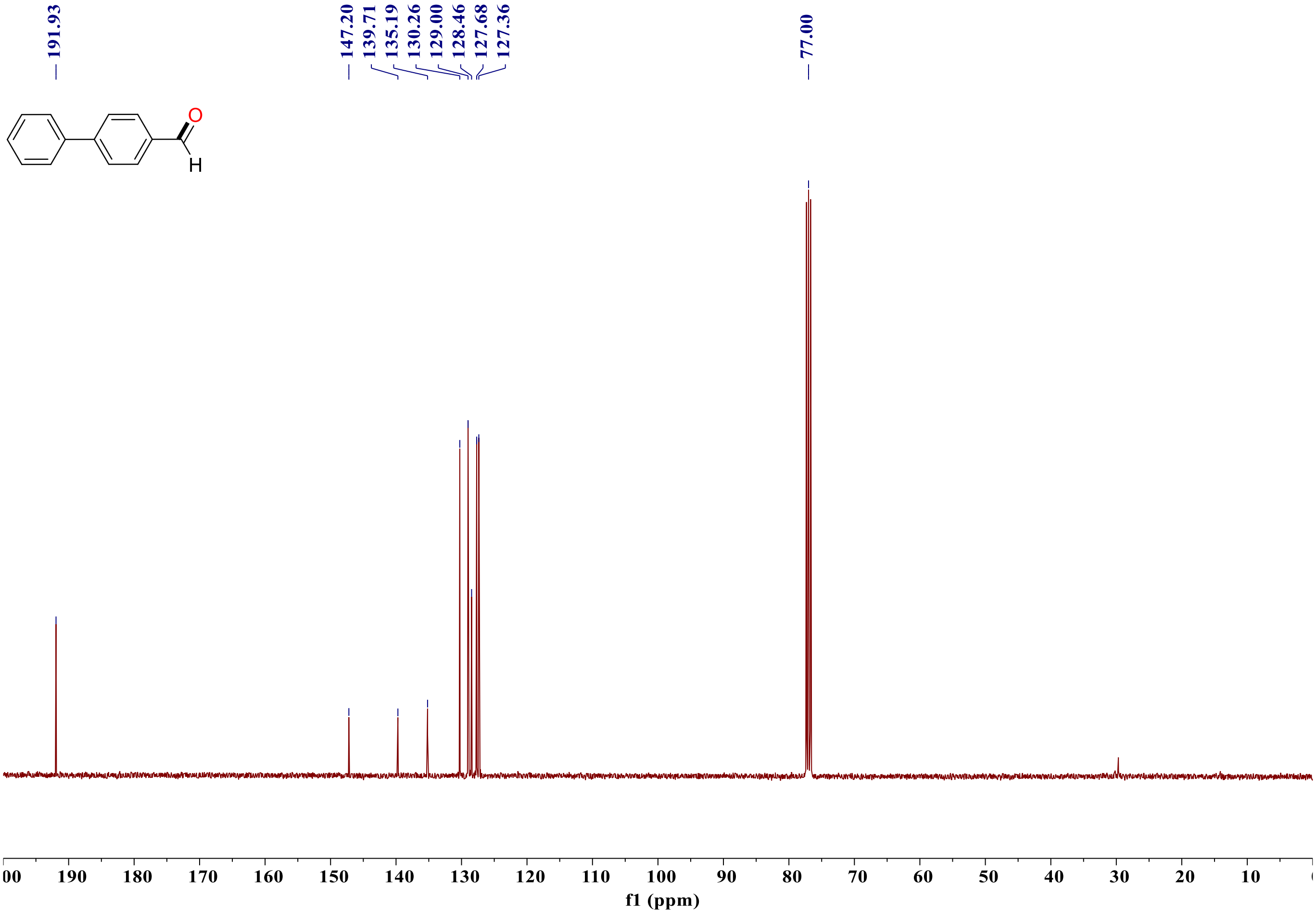

^{1}H NMR (400 MHz, $CDCl_3$), ^{13}C NMR (101 MHz, $CDCl_3$) of product 63

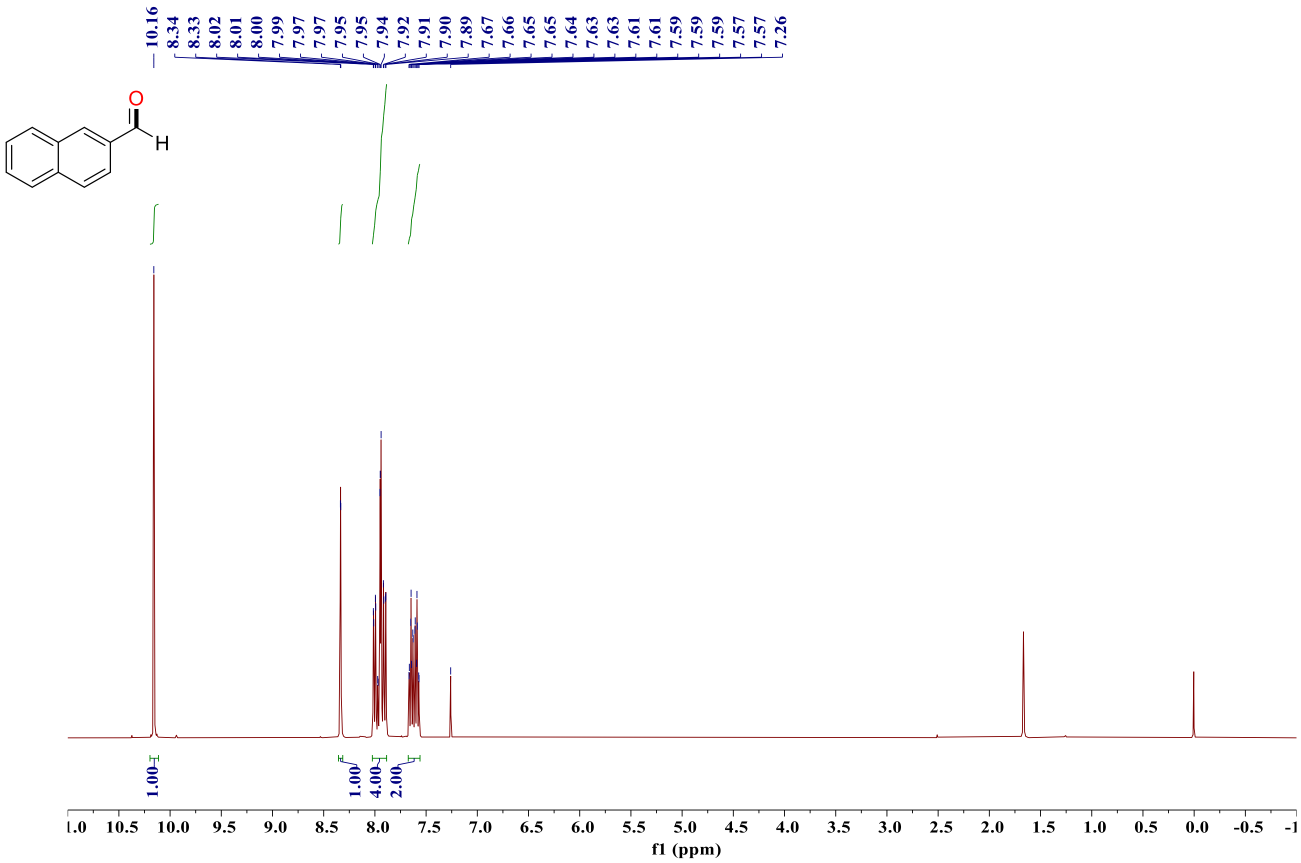

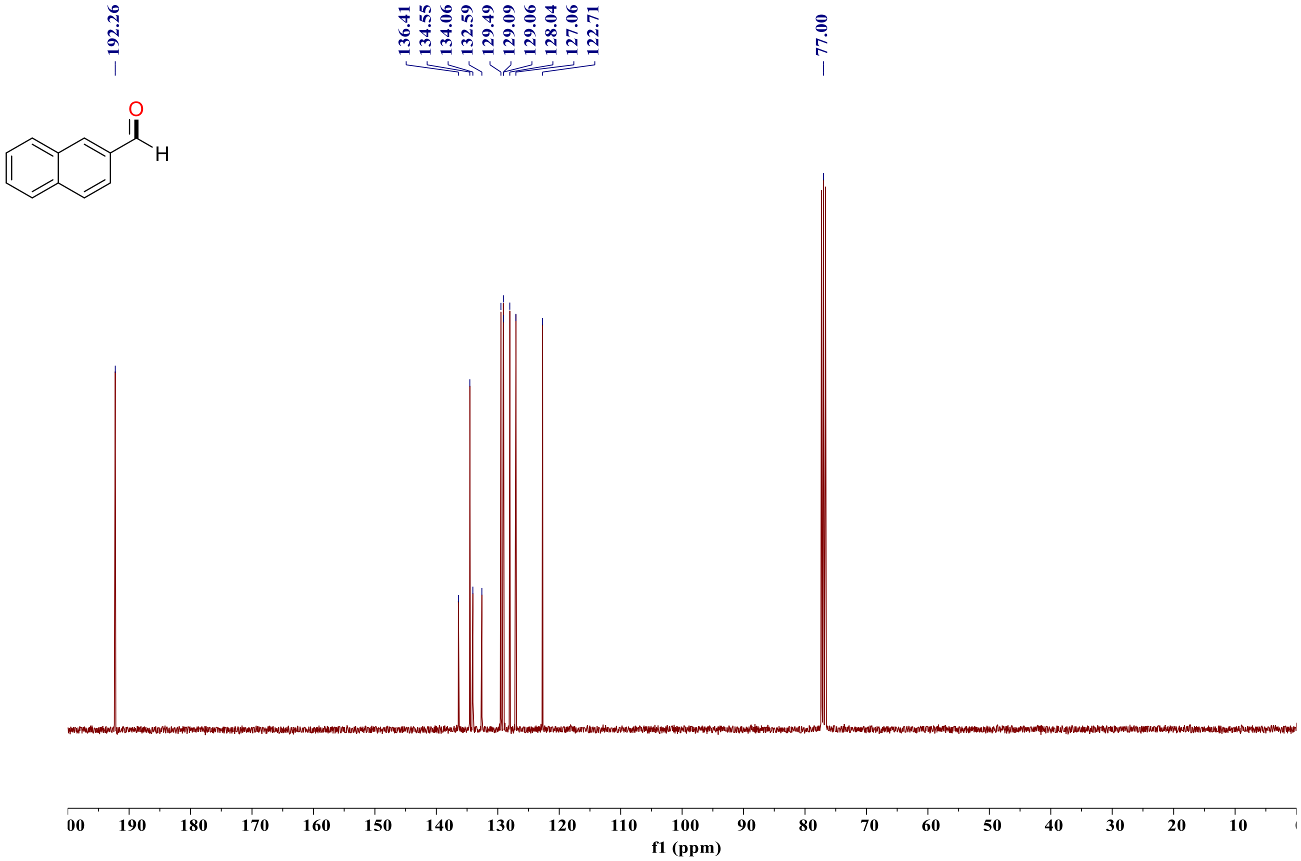

^{1}H NMR (400 MHz, $CDCl_3$), ^{13}C NMR (101 MHz, $CDCl_3$) of product 64

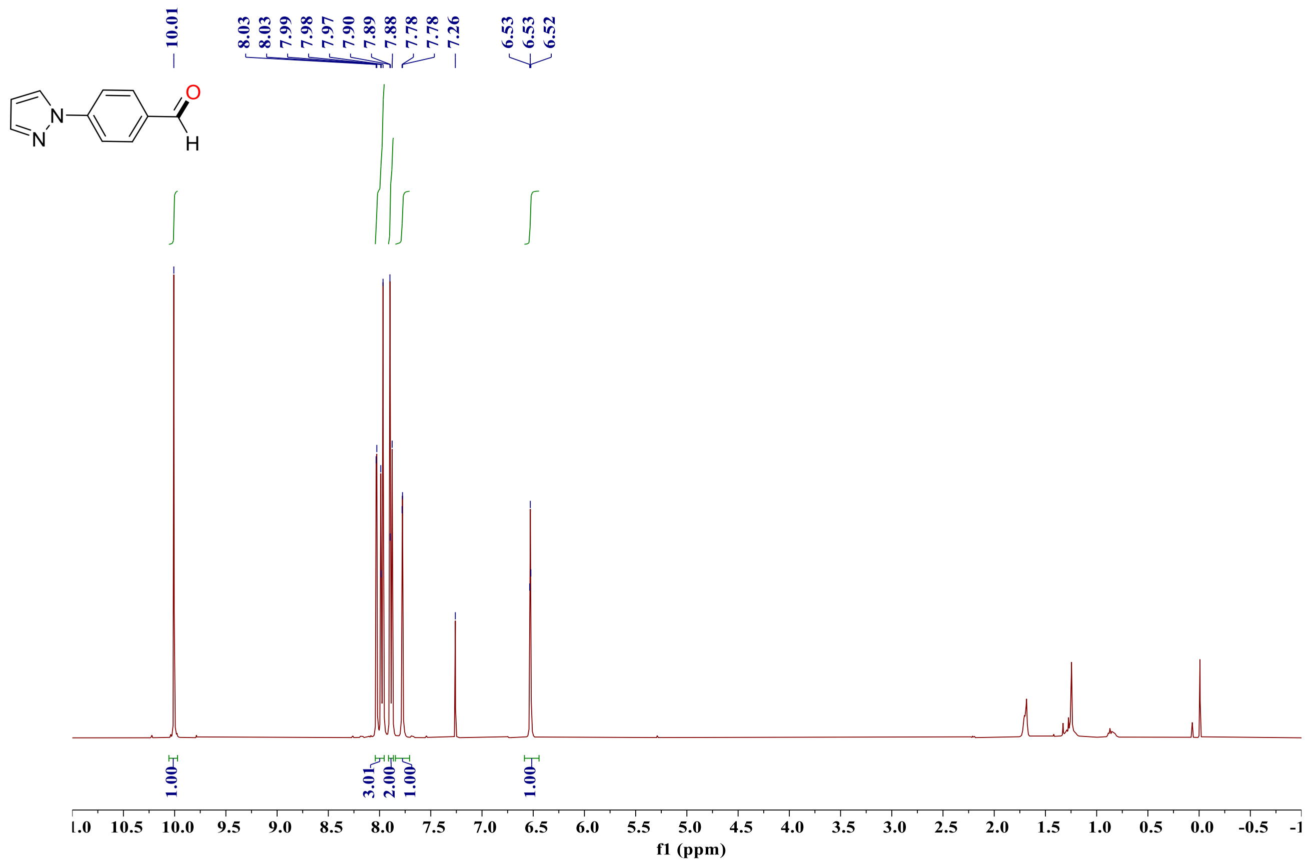

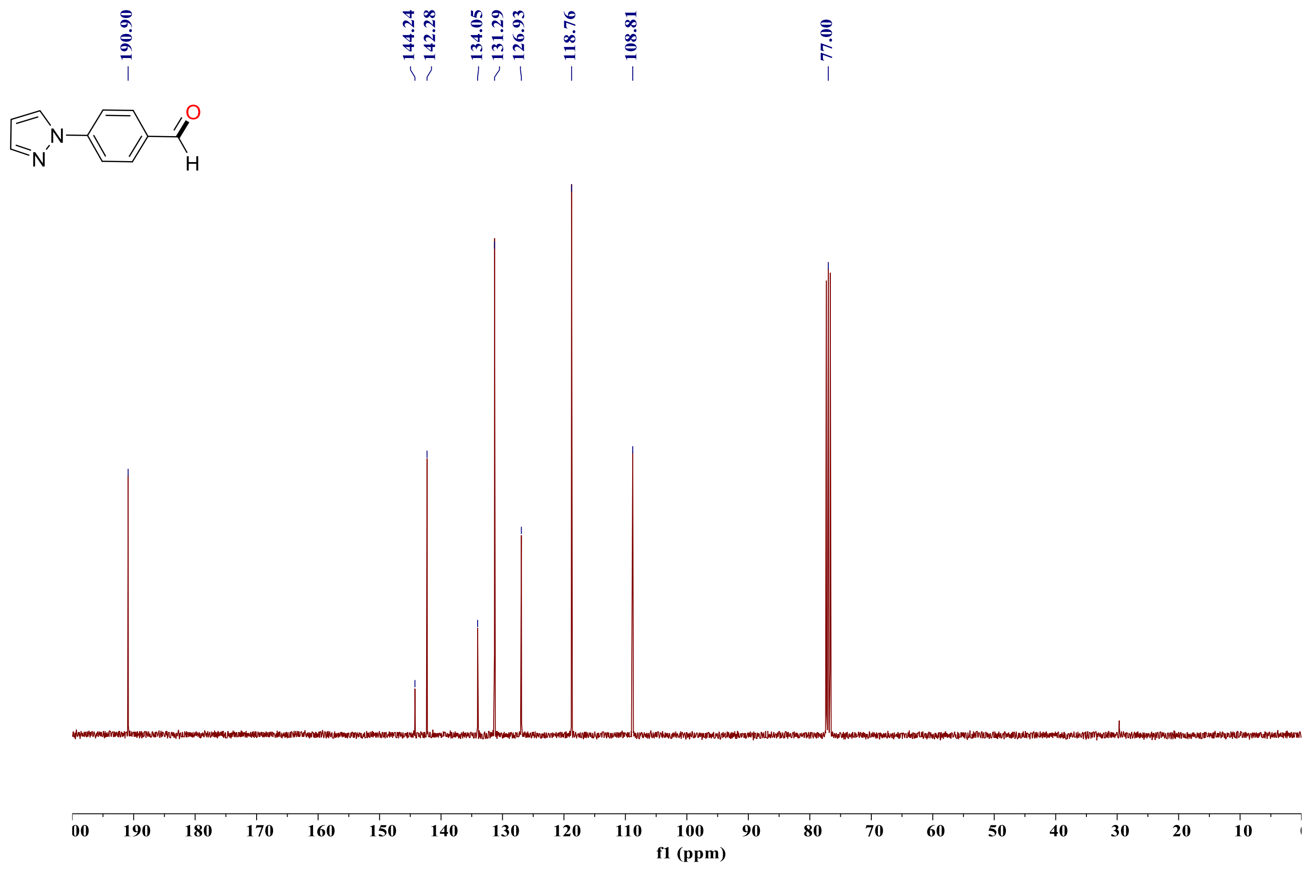

^{1}H NMR (400 MHz, CDCl$_3$), ^{13}C NMR (101 MHz, CDCl$_3$) of product 65

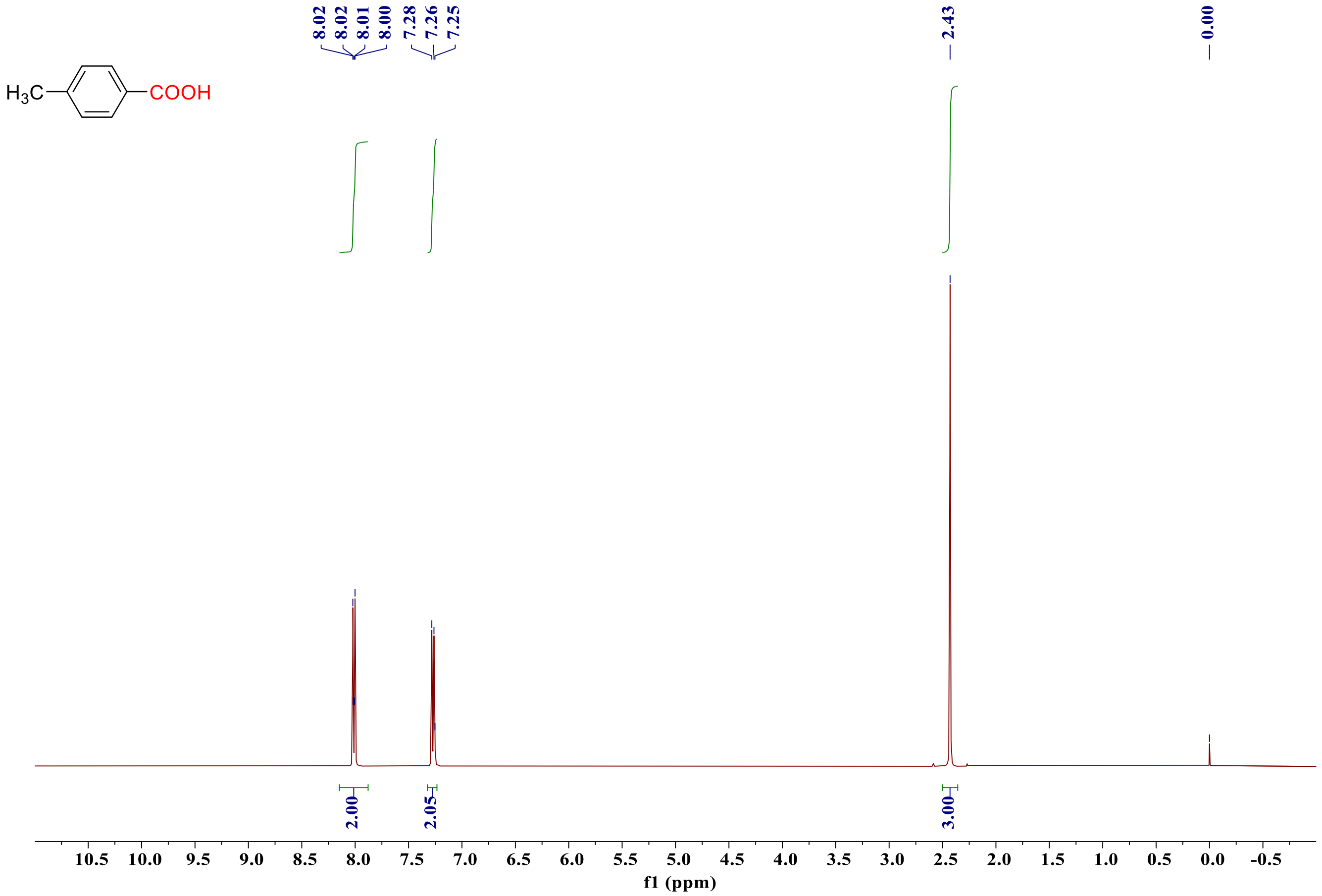

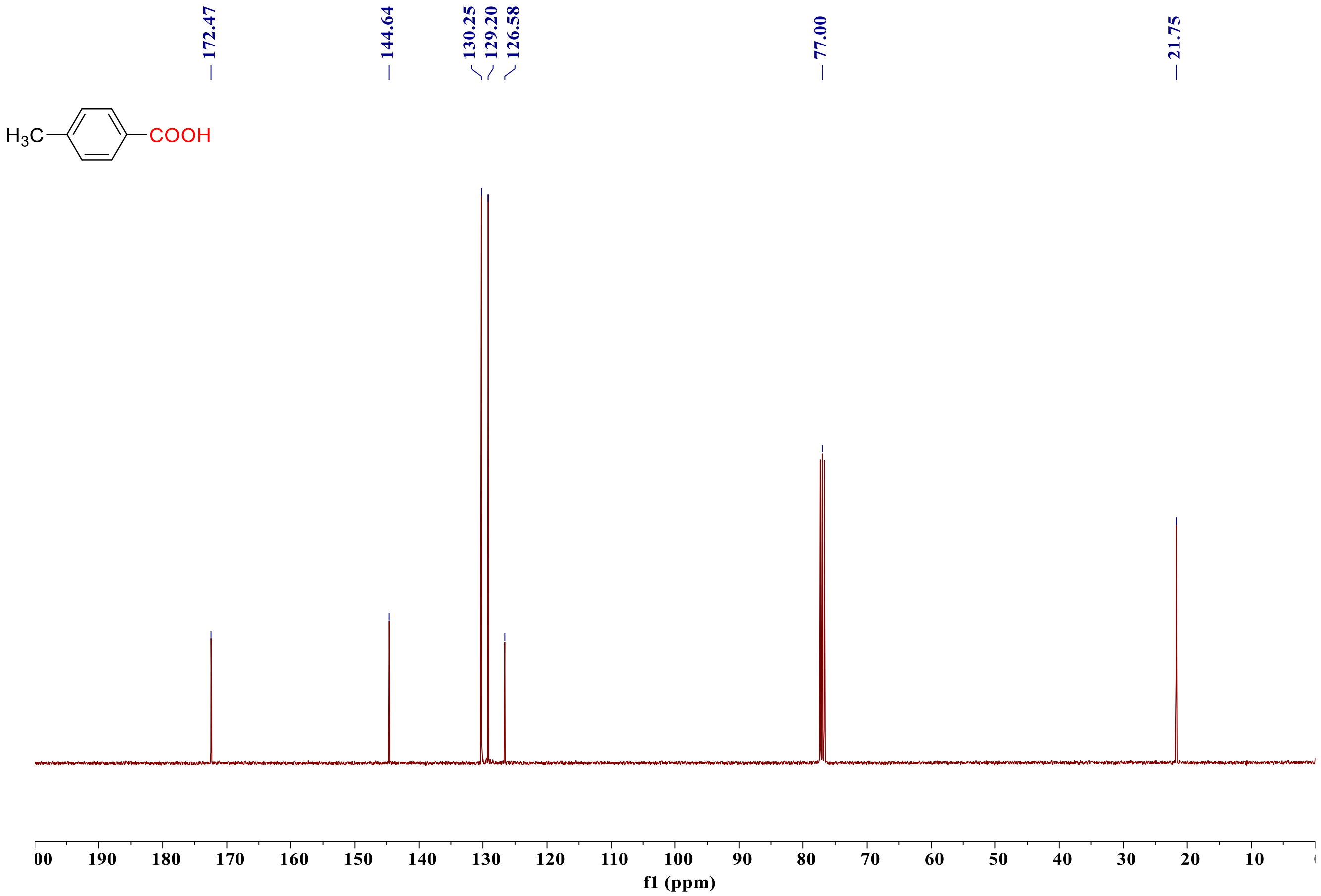

^{1}H NMR (400 MHz, $CDCl_3$), ^{13}C NMR (101 MHz, $CDCl_3$) of product 66

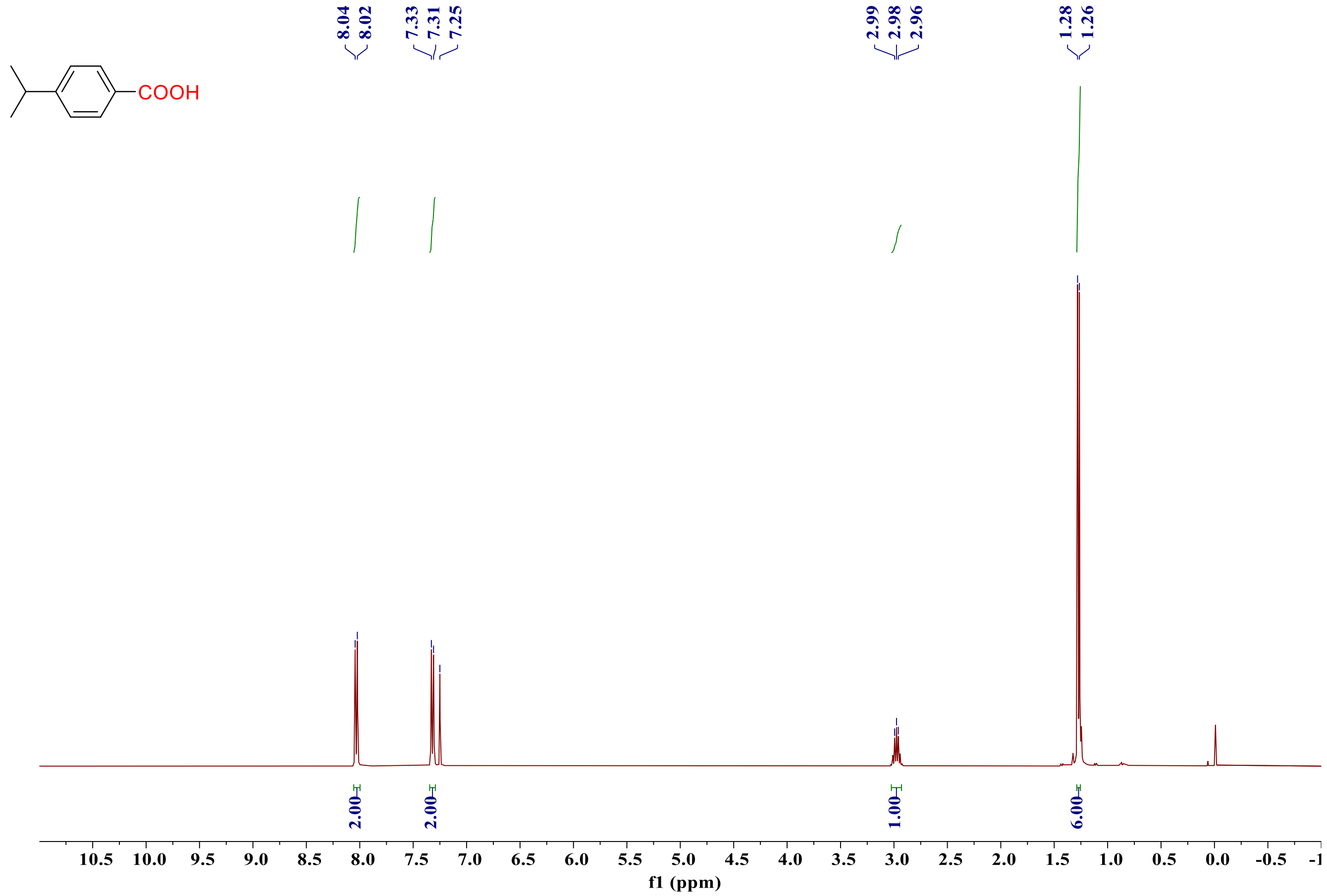

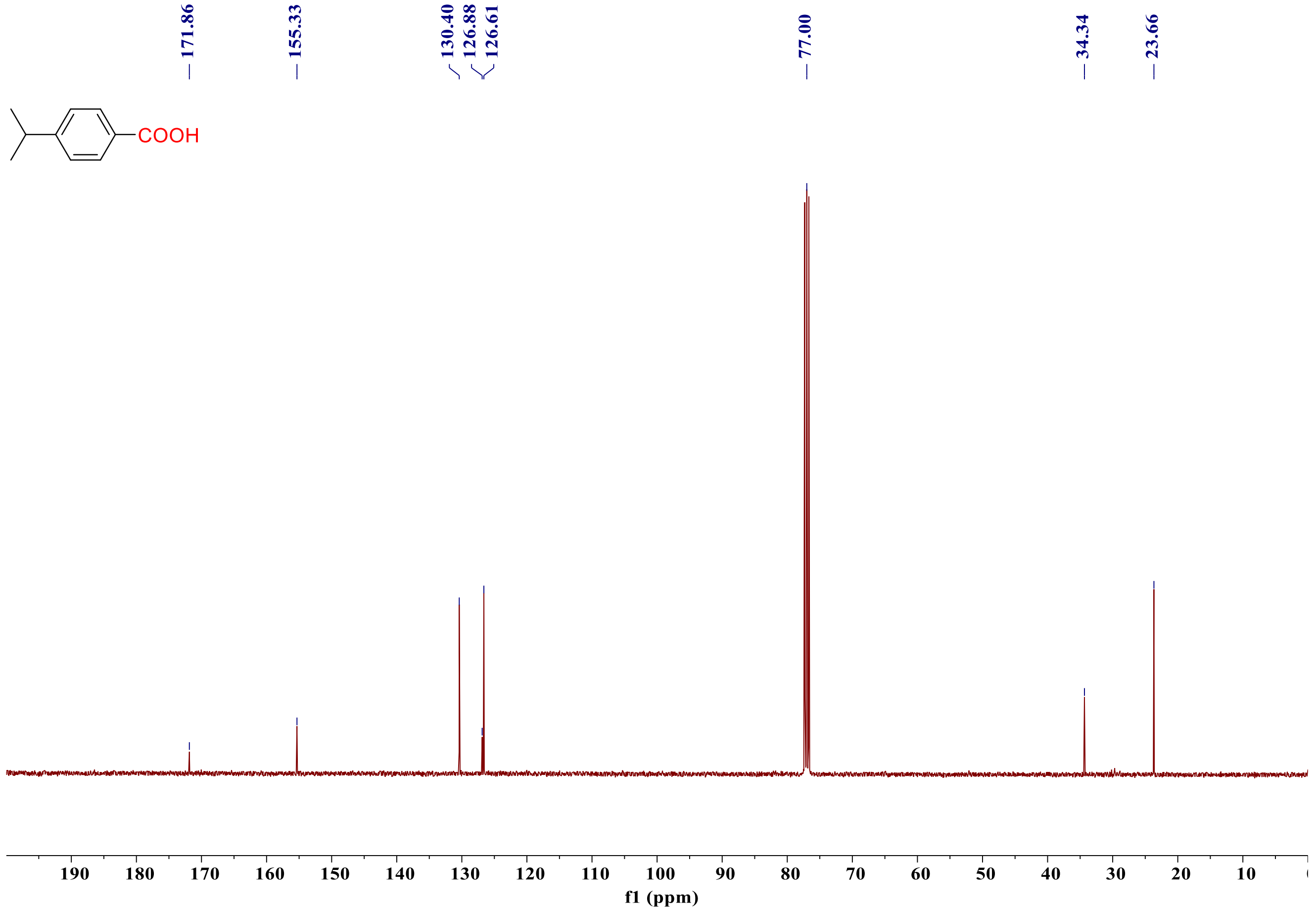

^{1}H NMR (400 MHz, $CDCl_3$), ^{13}C NMR (101 MHz, $CDCl_3$) of product 67

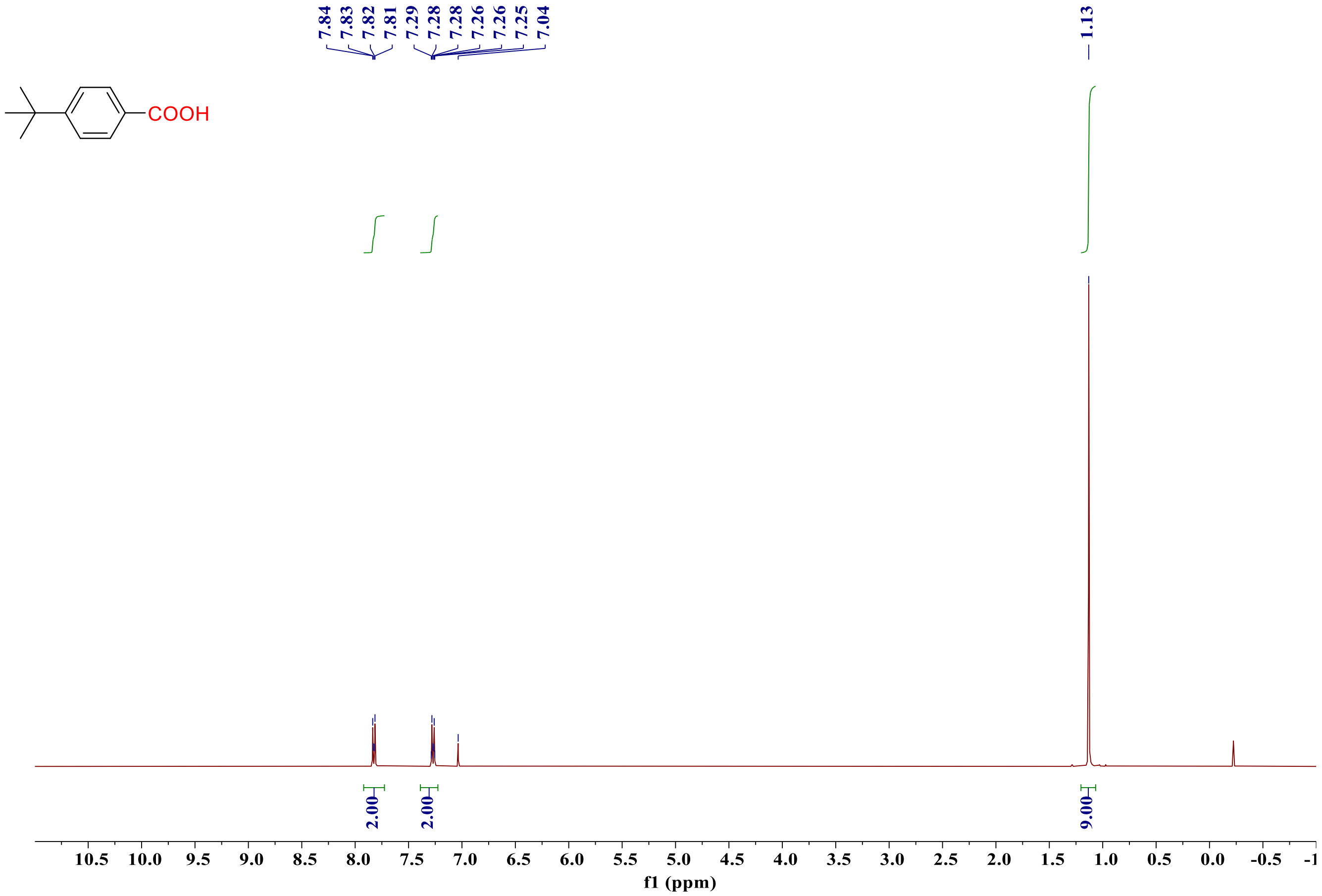

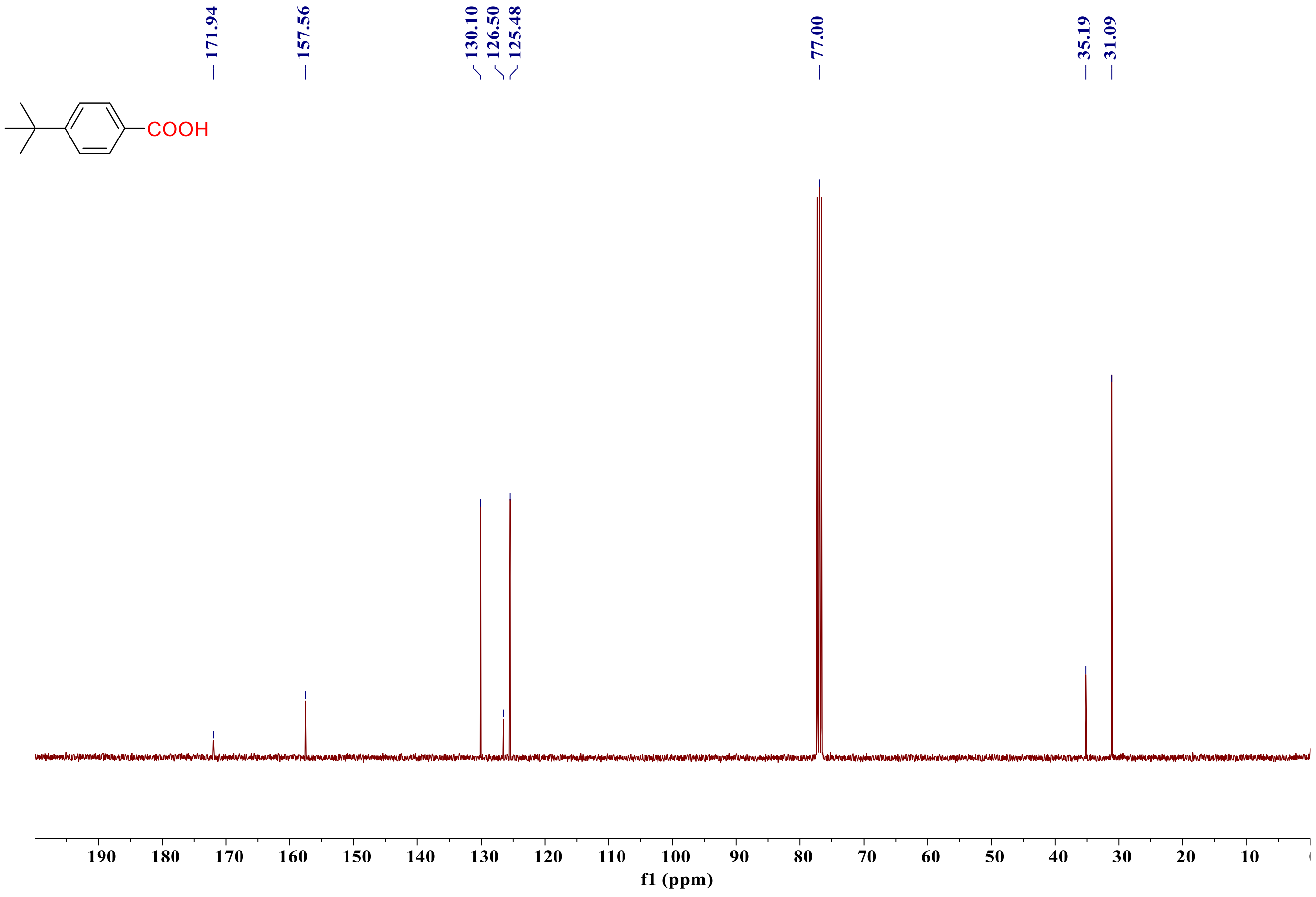

^{1}H NMR (400 MHz, $CDCl_3$), ^{13}C NMR (101 MHz, DMSO-D_6) of product 68

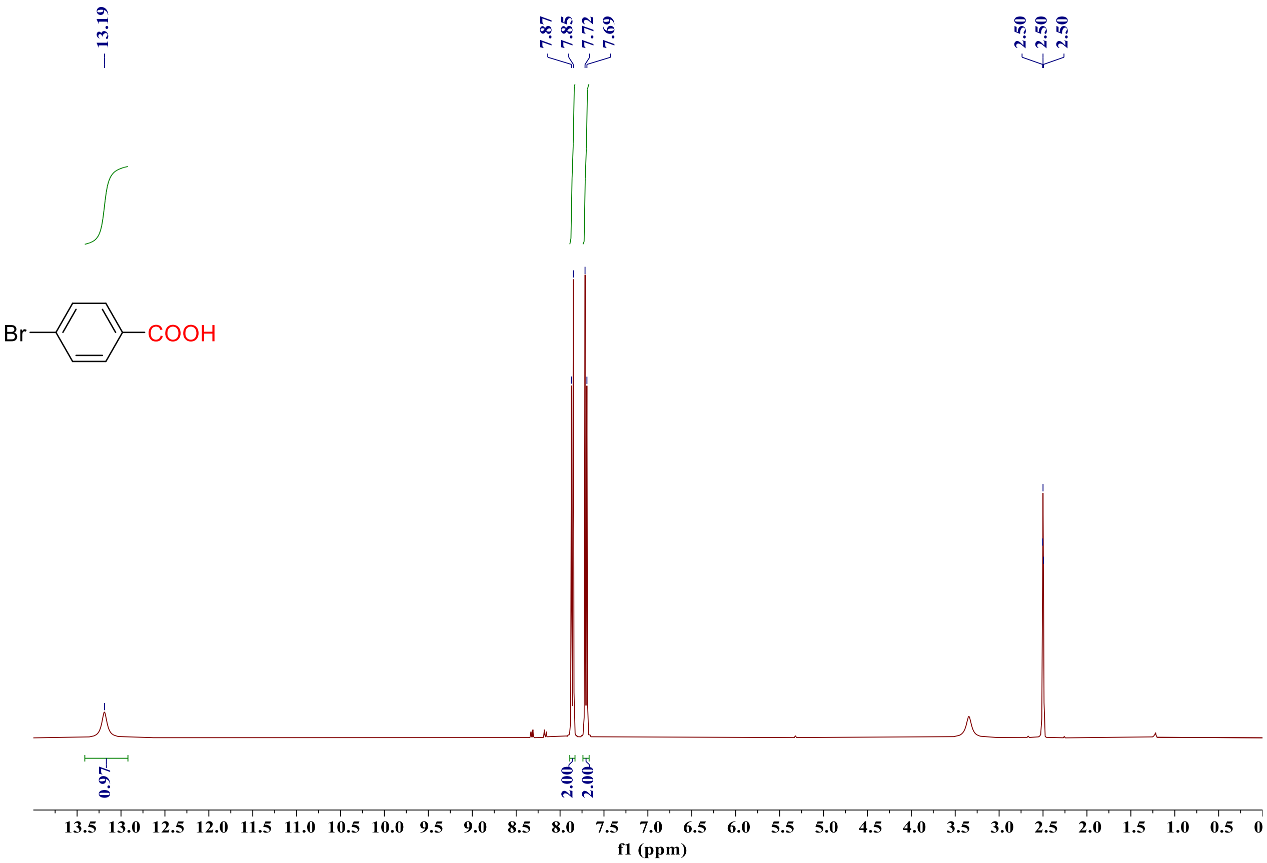

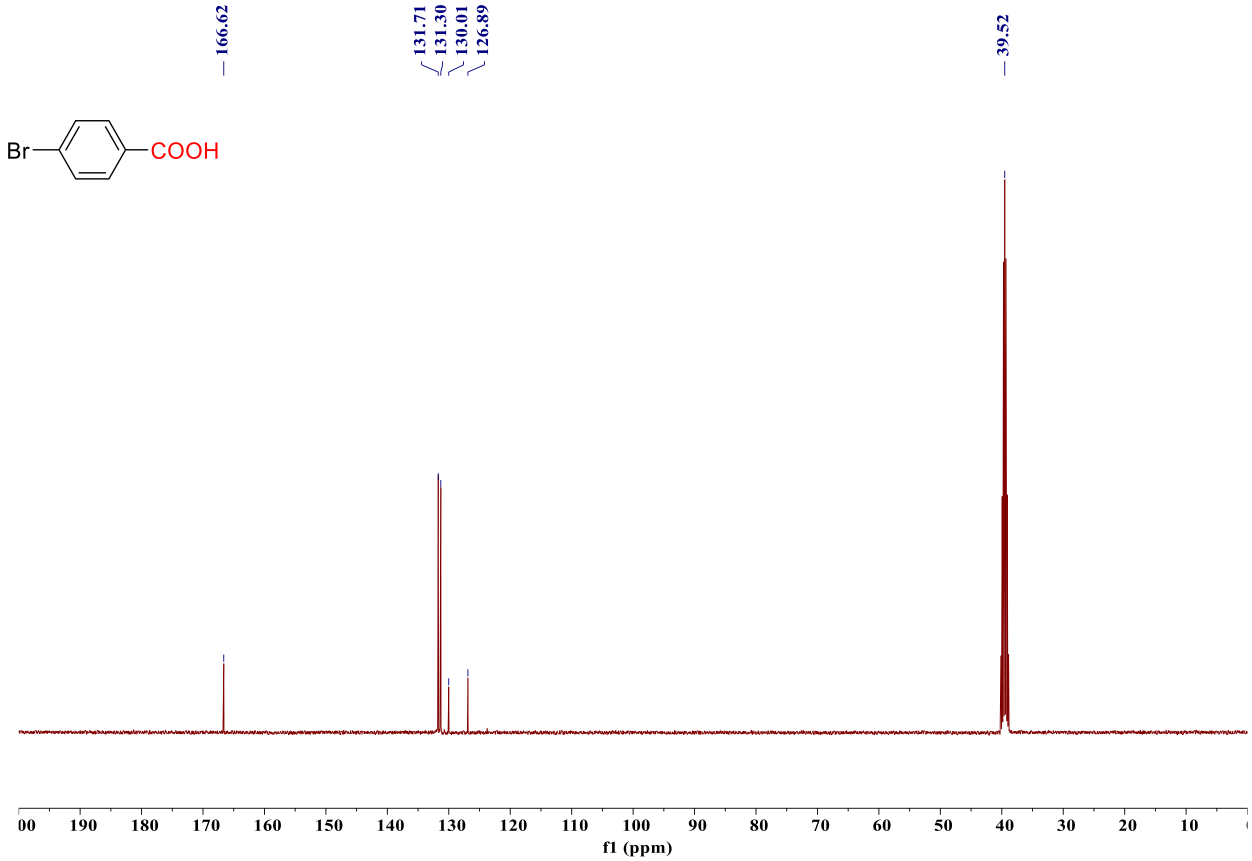

^{1}H NMR (400 MHz, $CDCl_3$), ^{13}C NMR (101 MHz, DMSO-D_6) of product 69

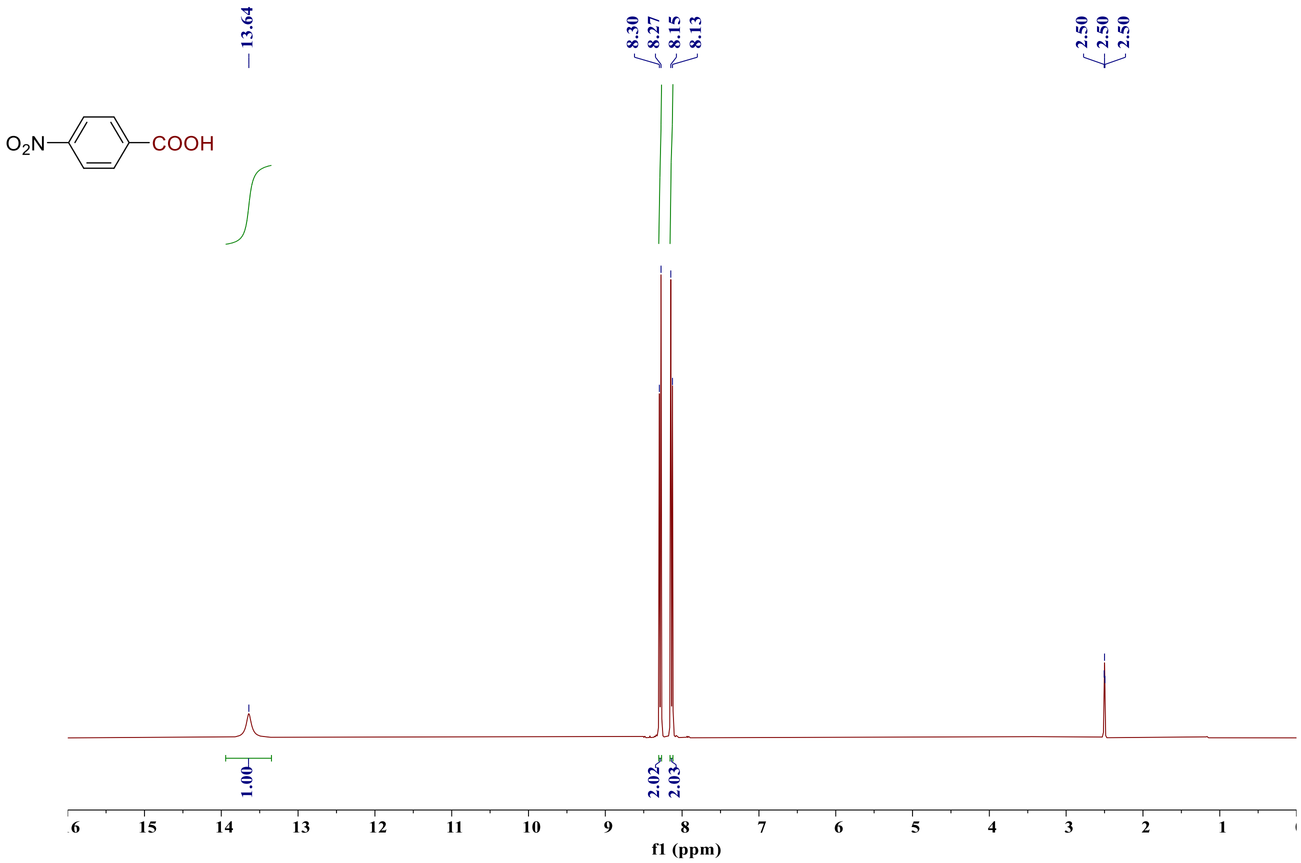

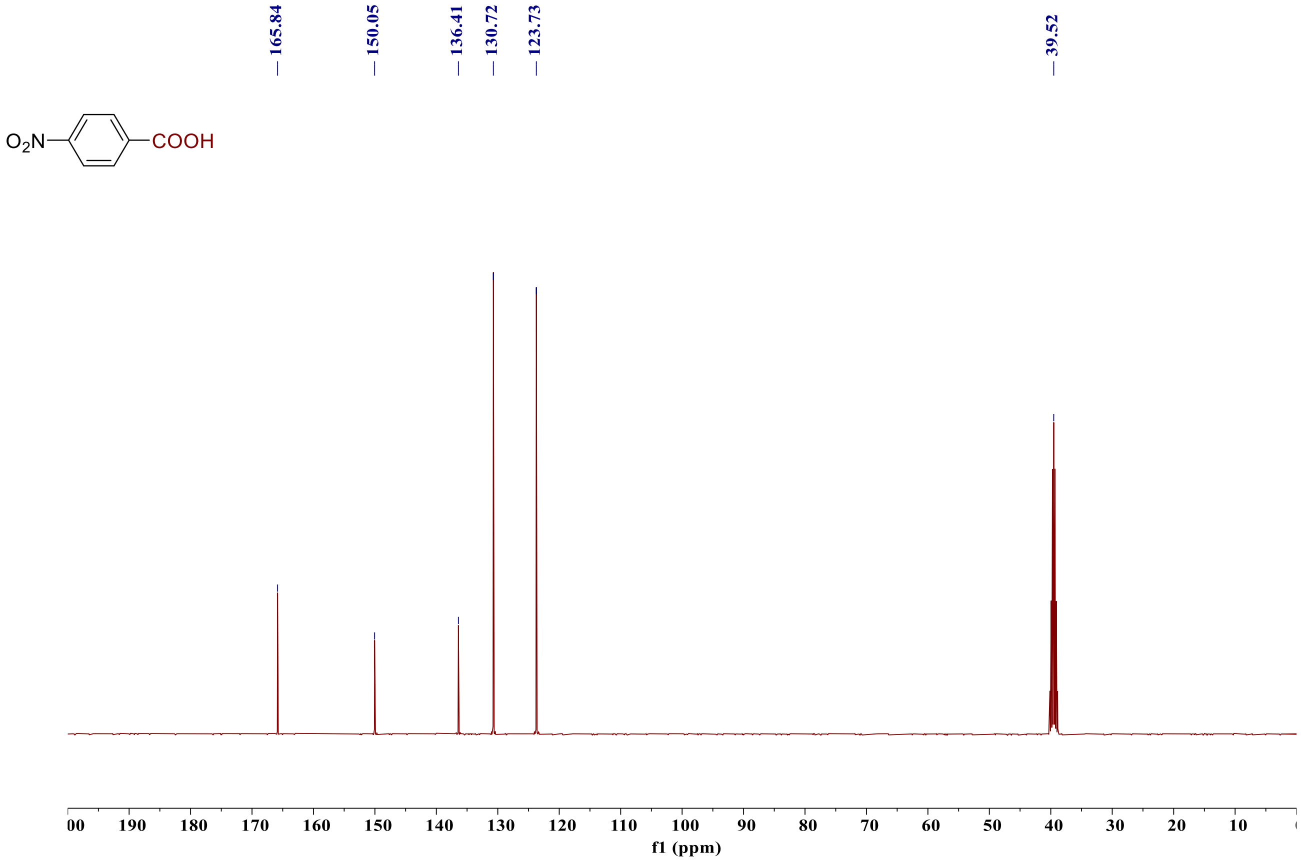

^{1}H NMR (400 MHz, $CDCl_3$), ^{13}C NMR (101 MHz, $CDCl_3$) of product 70

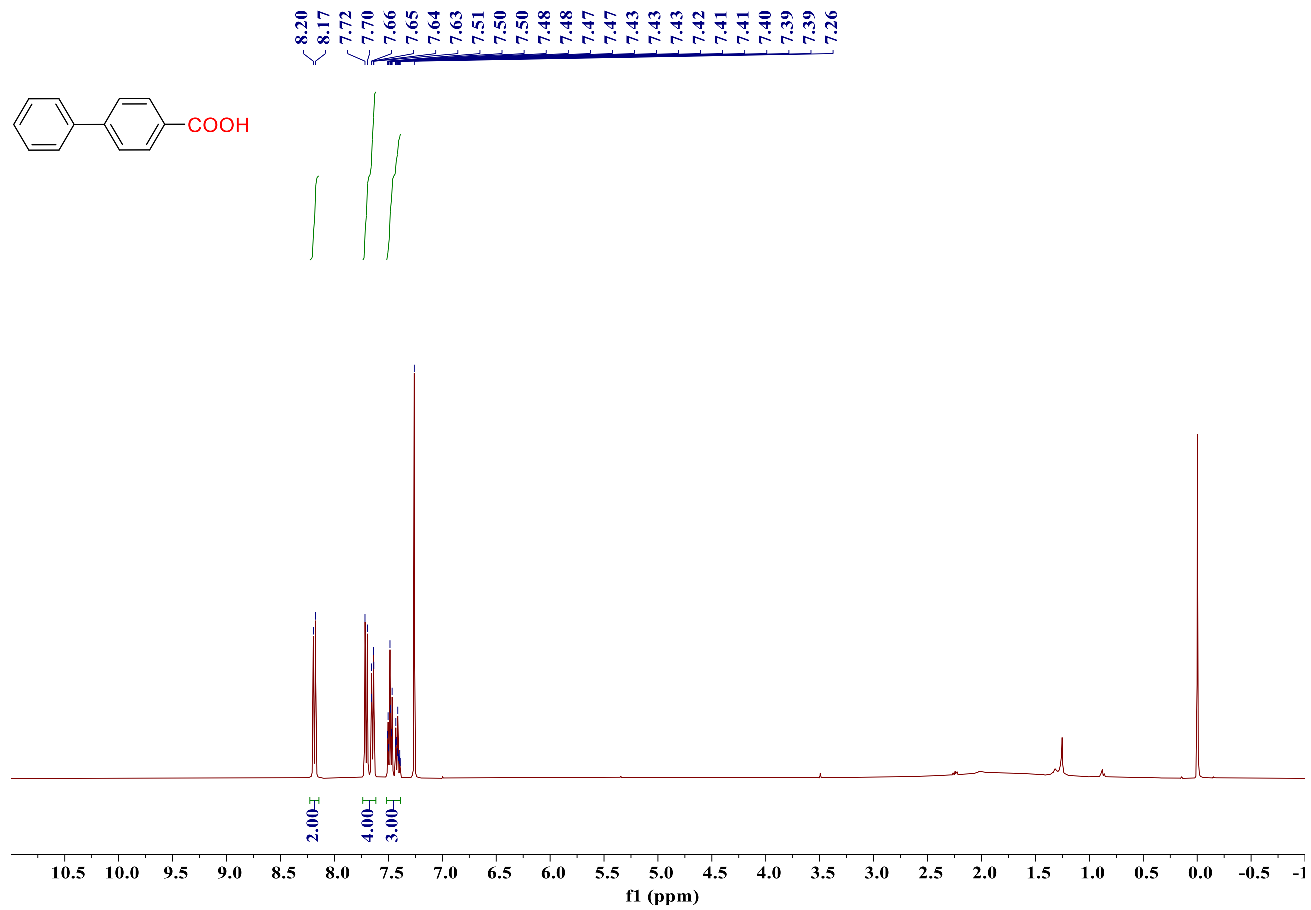

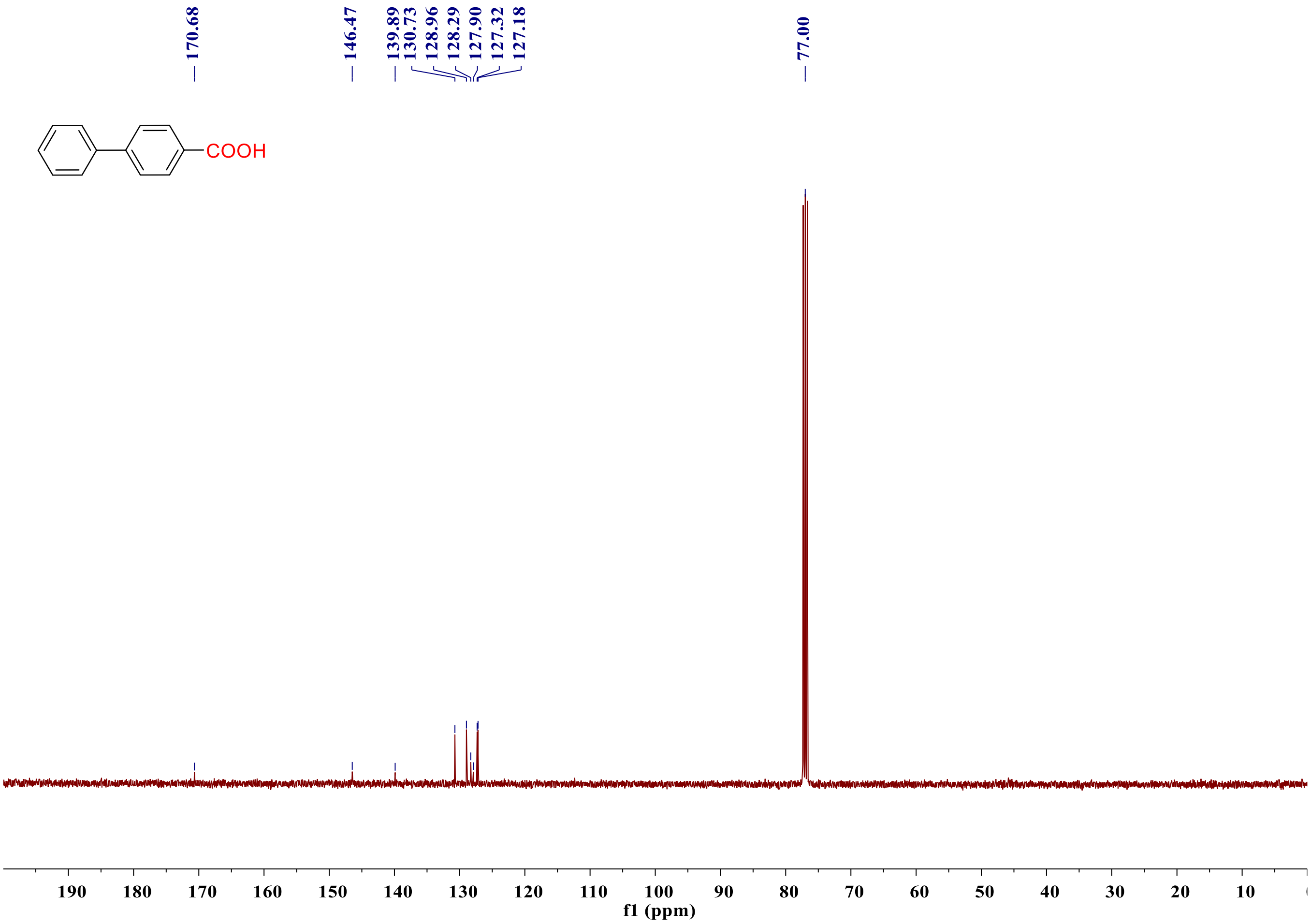

^{1}H NMR (400 MHz, $CDCl_3$), ^{13}C NMR (101 MHz, $CDCl_3$) of product 71

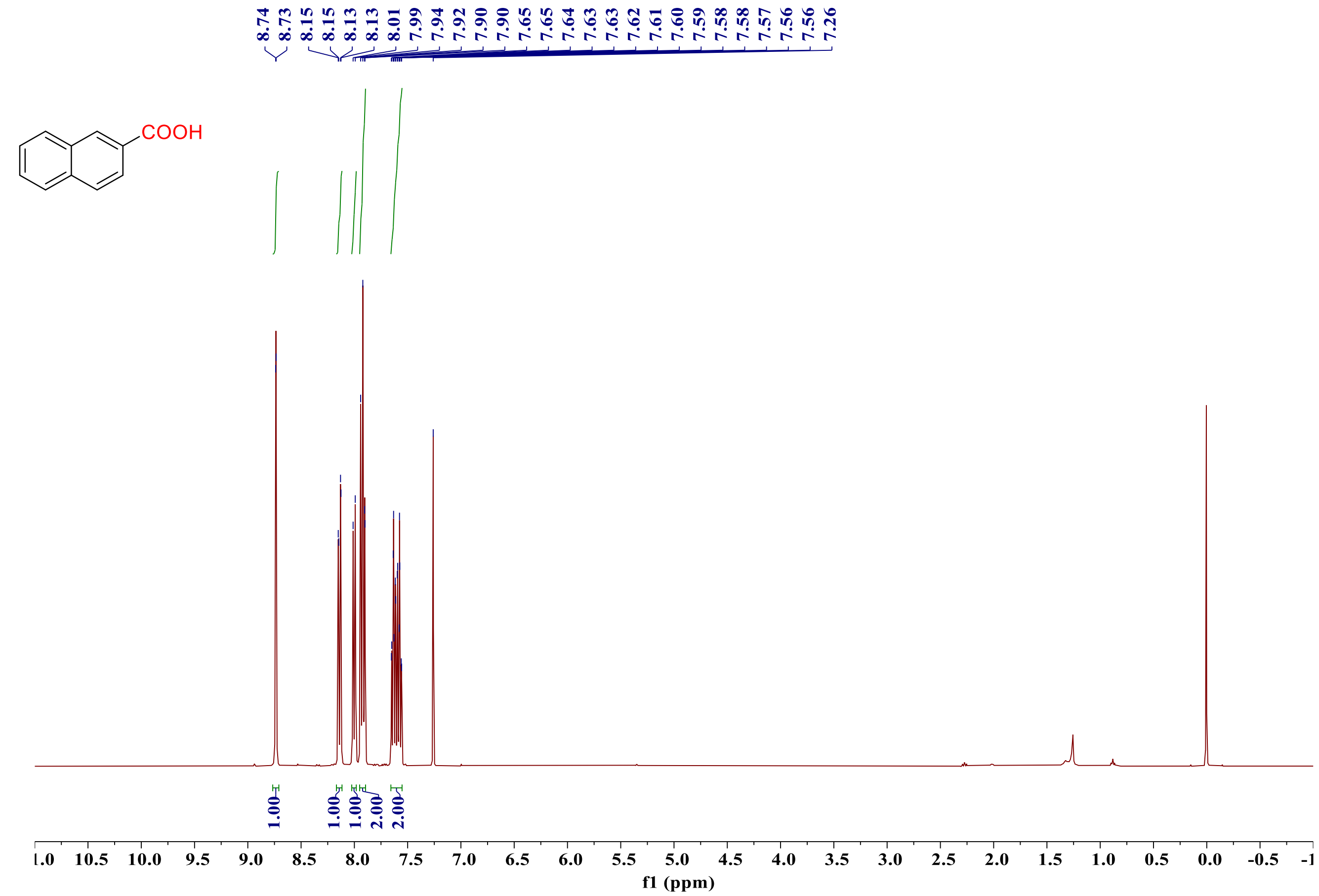

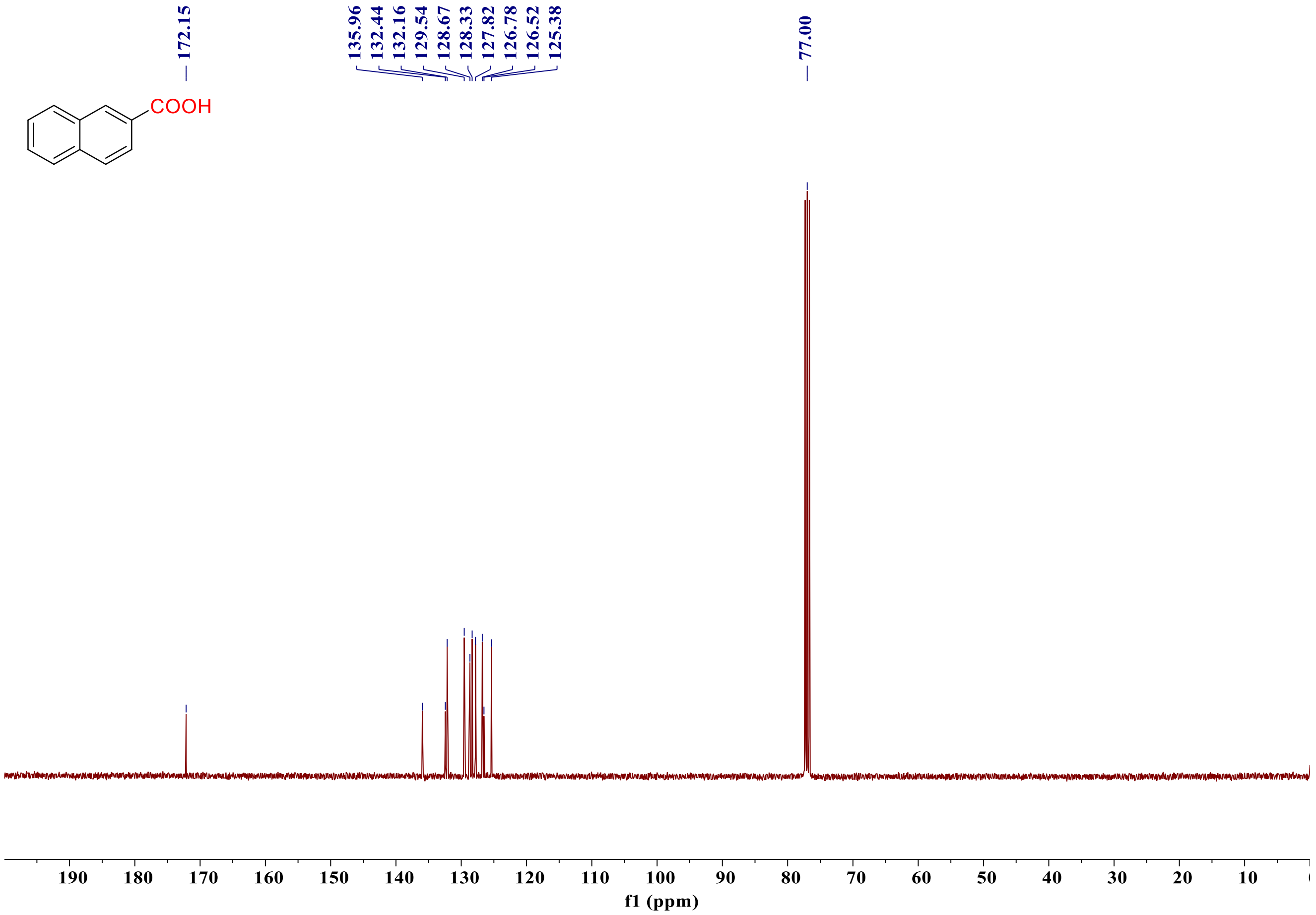

^{1}H NMR (400 MHz, DMSO-d_6), ^{13}C NMR (101 MHz, DMSO) of product 72

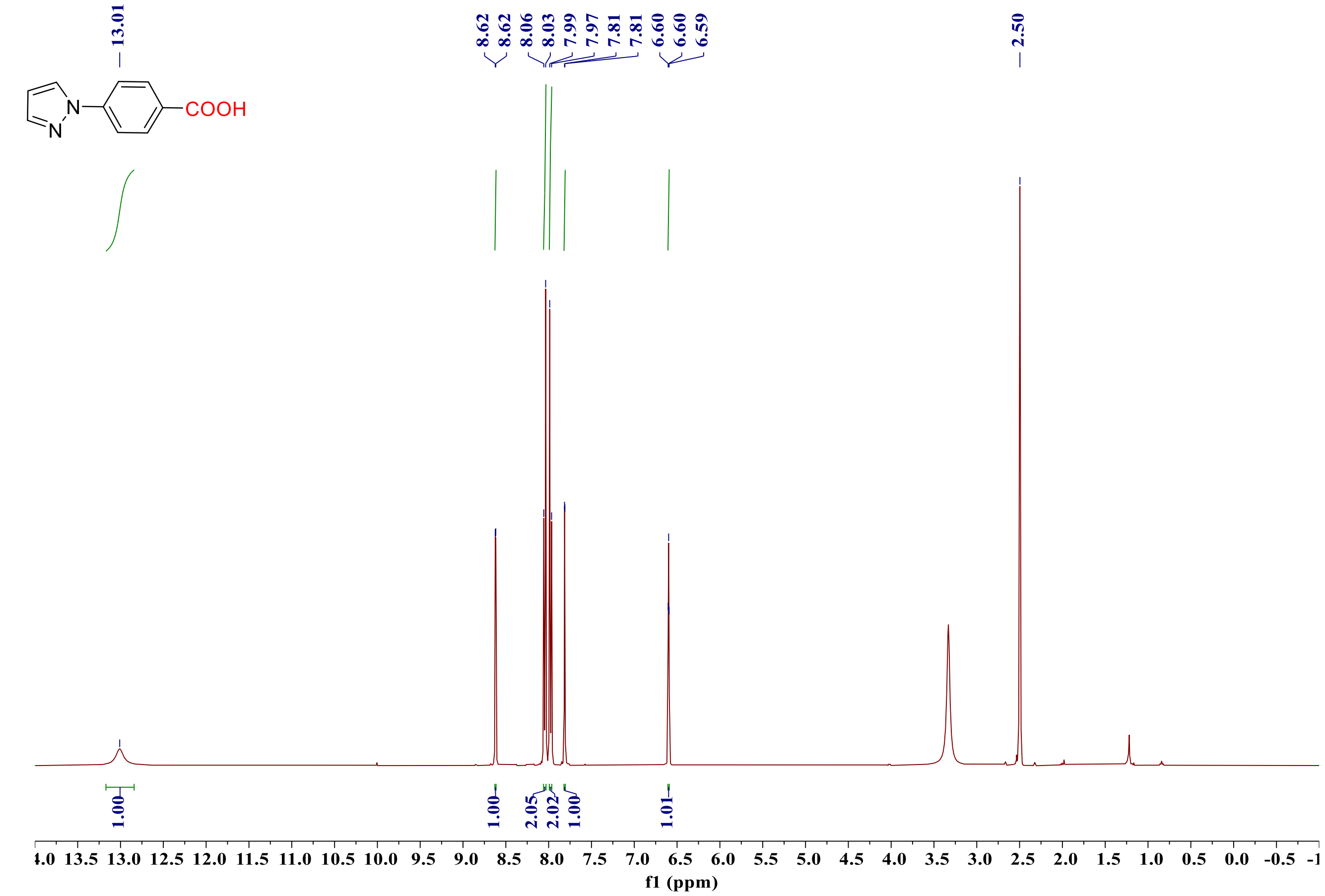

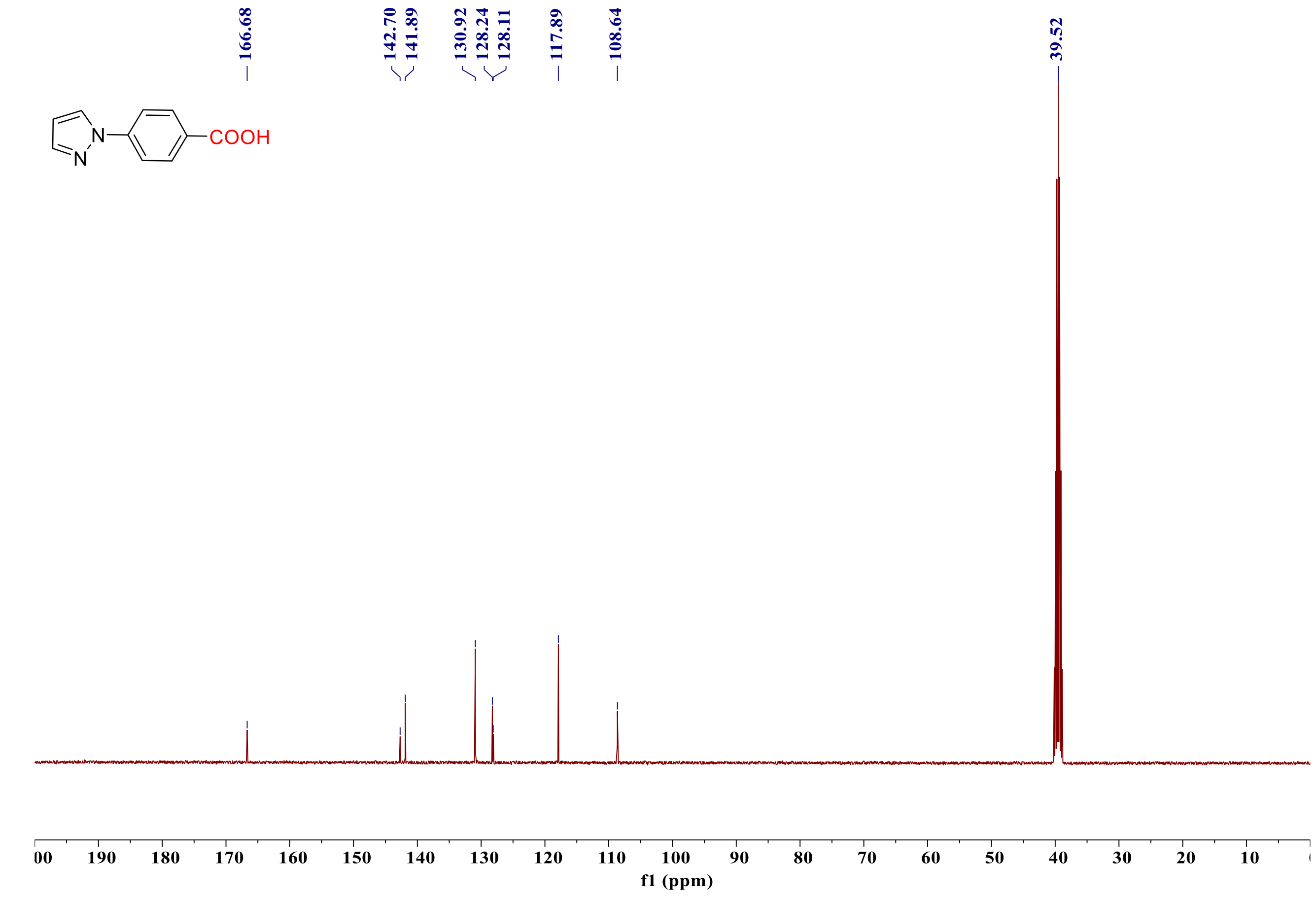

^{1}H NMR (400 MHz, $CDCl_3$), ^{13}C NMR (101 MHz, $CDCl_3$) of product 76

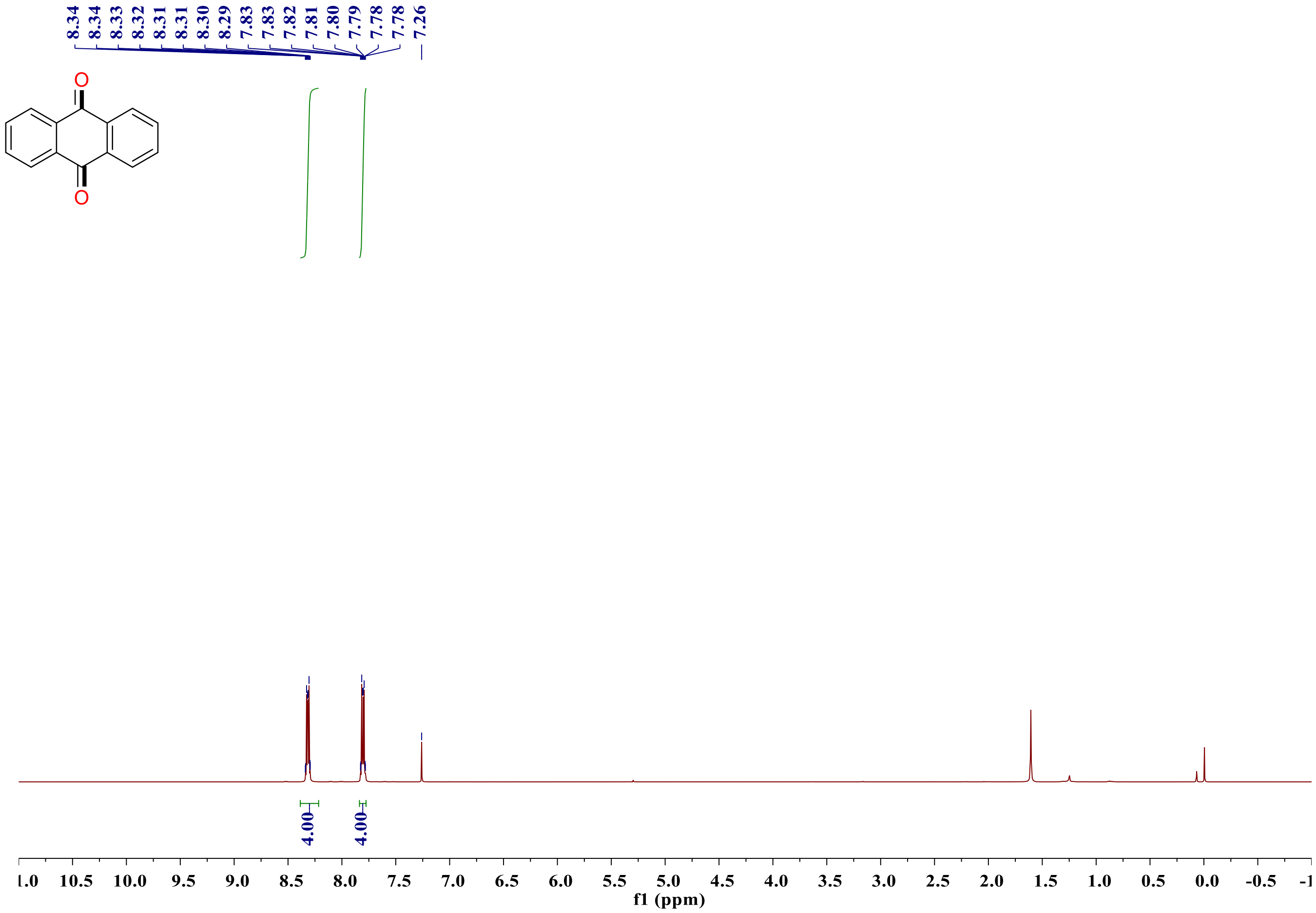

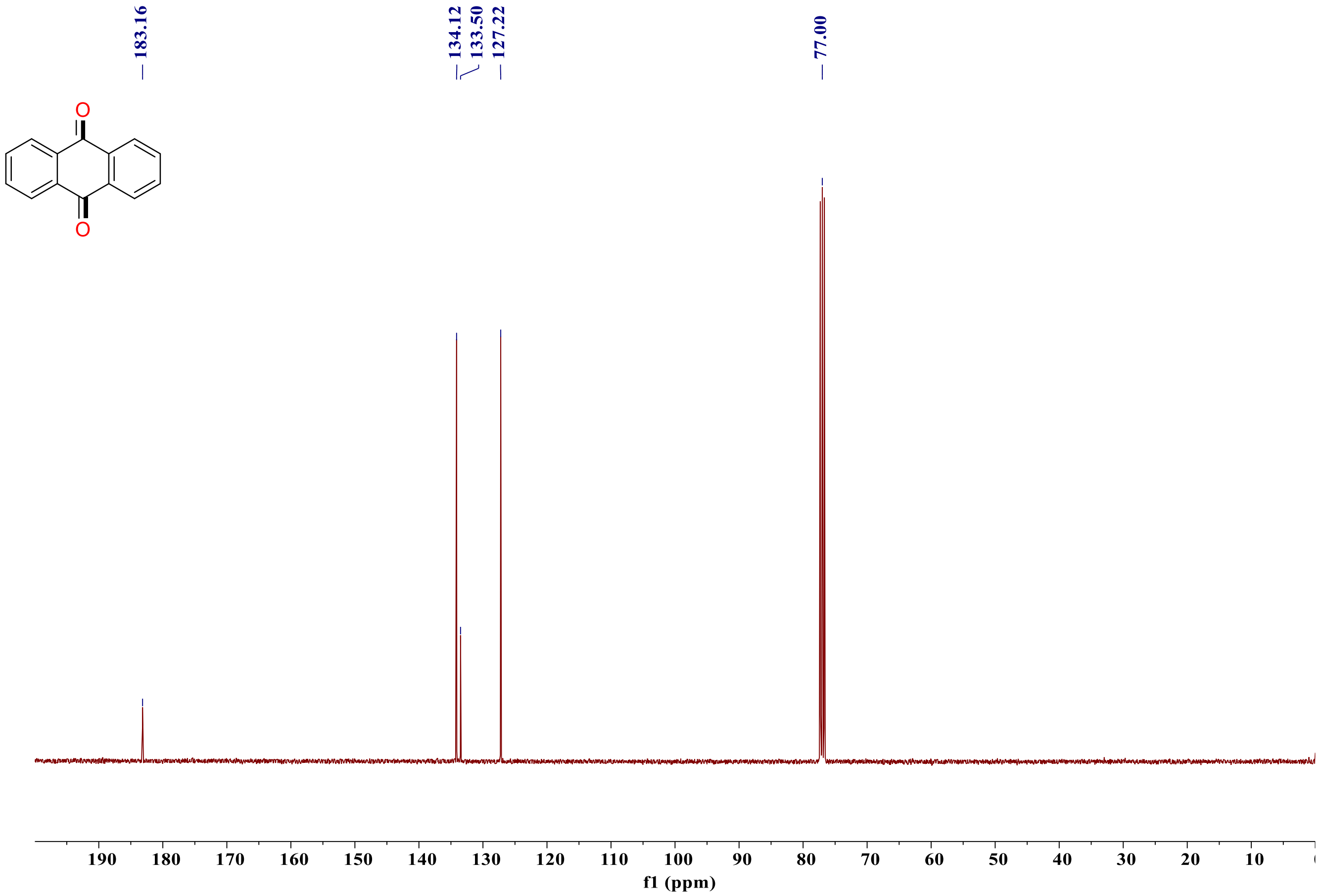

Supplement: Supplementary file 1 — Supporting Information [file ADVS-12-2417752-s001.pdf]
